# Supplementary material for: Discovery of Cancer Driver Long Noncoding RNAs across 1112 Tumour Genomes: New Candidates and Distinguishing Features
Source: Sci Rep. 2017 Jan 27;7:41544. doi: 10.1038/srep41544 (PMC5269722; doi:10.1038/srep41544)

Discovery of Cancer Driver Long Noncoding RNAs across 1112 Tumour Genomes: New Candidates and Distinguishing Features.

Authors

Andrés Lanzós[1,2,3], Joana Carlevaro-Fita[1,2,3], Loris Mularoni[4], Ferran Reverter[1,2,3], Emilio Palumbo[1,2,3], Roderic Guigó[1,2,3], Rory Johnson[1,2,3,5]*

1. Centre for Genomic Regulation (CRG), The Barcelona Institute of Science and Technology, Dr. Aiguader 88, Barcelona 08003, Spain

2. Universitat Pompeu Fabra (UPF), Barcelona, Spain.

3. Institut Hospital del Mar d'Investigacions Mèdiques (IMIM), 08003 Barcelona, Spain.

4. Research Unit on Biomedical Informatics, Department of Experimental and Health Sciences, Universitat Pompeu Fabra, Dr. Aiguader 88, Barcelona, Spain

5. Present address: Department of Clinical Research, University of Bern, Murtenstrasse 35, 3010 Bern, Switzerland.

* Correspondence to rory.johnson@dkf.unibe.ch

Supplementary Figure Legends

Supplementary Figure S1: Graphical representation of the ExInAtor workflow. The objective of this algorithm is the creation of a local background region with identical trinucleotide content to the exons of a given gene. As an example we take a hypothetical single-exon lncRNA gene. (A) Upstream and downstream regions within the window size are defined as background regions. All the trinucleotides in the exon region are counted. The mutations (indicated by *) in the central nucleotide positions of each trinucleotide are counted. Colours indicate the four nucleotides in the exon. Background region nucleotides are not coloured. For brevity, only four trinucleotide combinations are shown. (B) Next, an iterative sampling is performed. A set of trinucleotides, identical to the exonic trinucleotide set, are randomly sampled from the background region. This operation is repeated, without replacement of the central nucleotide (red "X"), until it is no longer possible to extract the appropriate set of trinucleotides. (C) The total number of nucleotide positions and mutations are counted for the exon region and the sampled background region. These values are used for the contingency table analysis shown in Figure 1.

Supplementary Figure S2: Ratio of exonic to background mutation density.

Supplementary Figure S3: Quantile-quantile ("QQ") plots for all analyses. Grey dots represent the P values obtained in the two simulations. The black and coloured dots correspond to the P values calculated in the real dataset.

Supplementary Figure S4: Histogram of predicted candidates in Breast across the 100 iterations of shuffling analysis. First, all lncRNAs randomly placed in a new position within the whole genome, while maintaining exon-intron structure. Then ExInAtor was run, with same settings as described, using real mutations from Breast. Shown are the number of predicted candidates at a cutoff of FDR<0.1. The number of predicted candidates using real gene locations is 3, greater than 97% of simulations.

Supplementary Figure S5: Quantile-quantile ("QQ") plot of "intronic gene" simulation in Breast. Artificial "intronic" gene models were constructed, where the exons of every gene were replaced by equally-sized, randomly-selected fragments of introns from the same gene. These genes were used as the input for ExInAtor and run (with same settings as for real data) on Breast samples.

Supplementary Figure S6: Expression of SAMMSON in Stomach (in which is detected by ExInAtor), and skin, sun-exposed or not (in which has been previously implicated in Melanoma).

Supplementary Figure S7: Exon-level mutational density of all lncRNAs candidates against the background regions.

Supplementary Figure S8: Intersection between ExInAtor, MiTranscriptome and Du et al candidates.

Supplementary Figure S9: Performance of ExInAtor compared MutSig, OncodriveFM and OncodriveClust in the Pancancer Alexandrov dataset. (A) Percentage of CGC candidates amongst predicted drivers for each method across different cutoffs (-log10 Q value) in all the Alexandrov cancer files. (B) As for (A), but displayed by ranked number of top predicted drivers.

Supplementary Figure S10: The overlap of predicted protein-coding drivers between methods. The percent of predicted drivers of each method on Pancancer Alexander mutations that are also predicted by at least one of the other methods displayed by (A) cutoff or by (B) ranked number of candidates.

Supplementary Figure S11: QQ plots of each method on the Pancancer Alexandrov dataset. Grey dots represent the P values obtained in a simulation. The black and coloured dots correspond to the P values calculated in the real dataset.

Supplementary Figure S12: Tumour specificity of lncRNA and protein coding genes. The figure shows the percent of genes of each biotype that are mutated in one or more distinct tumour samples.

Supplementary Figure S13: Screenshots of the 12 candidate's mutations analysed with IGV.

Supplementary Figure S14: Replication timing of cancer lncRNAs, ExInAtor predictions and other lncRNAs. Cumulative distribution are shown for S50 values in HeLa cells assigned to lncRNA gene sets. S50 ratio is defined as the fraction of the S phase at which 50% of the DNA is replicated (see Materials and Methods). Low ratios indicate early replication timing. Genes are grouped by: literature-reported cancer CRL lncRNAs; all ExInAtor candidates (both CRL and not); non-CRL novel ExInAtor candidates; non-CRL non-candidates, being all other GENCODE lincRNAs. Candidates here were defined at a threshold of $Q \leq 0.2$. Dashed vertical lines indicate the mean value of each group.

Supplementary File Legends

Supplementary File S1: CRL lncRNAs.

Supplementary File S2: Non-CRL lncRNAs.

Supplementary File S3: The full set of lncRNA drivers predicted by ExInAtor in CRL.

Supplementary File S4: The full set of lncRNA drivers predicted by ExInAtor in non-CRL.

Supplementary File S5: The full set of protein-coding drivers predicted by ExInAtor.

Supplementary File S6: The full set of results for lncRNAs and protein-coding genes described in this study.

Supplementary File S7: Candidates identified by Du et al. that are also considered in our study.

Supplementary File S8: Candidates identified by MiTranscriptome that are also considered in our study.

Supplementary File S9: The full set of CGC drivers analysed in this study.

Supplementary File S10: The set of IDs for protein-coding genes used in this study.

Supplementary File S11: Protein-coding overlap with CGC across all cancers and both datasets.

Supplementary File S12: Effects on ExInAtor predictions of various filtering schemes.

Supplementary Figure S1

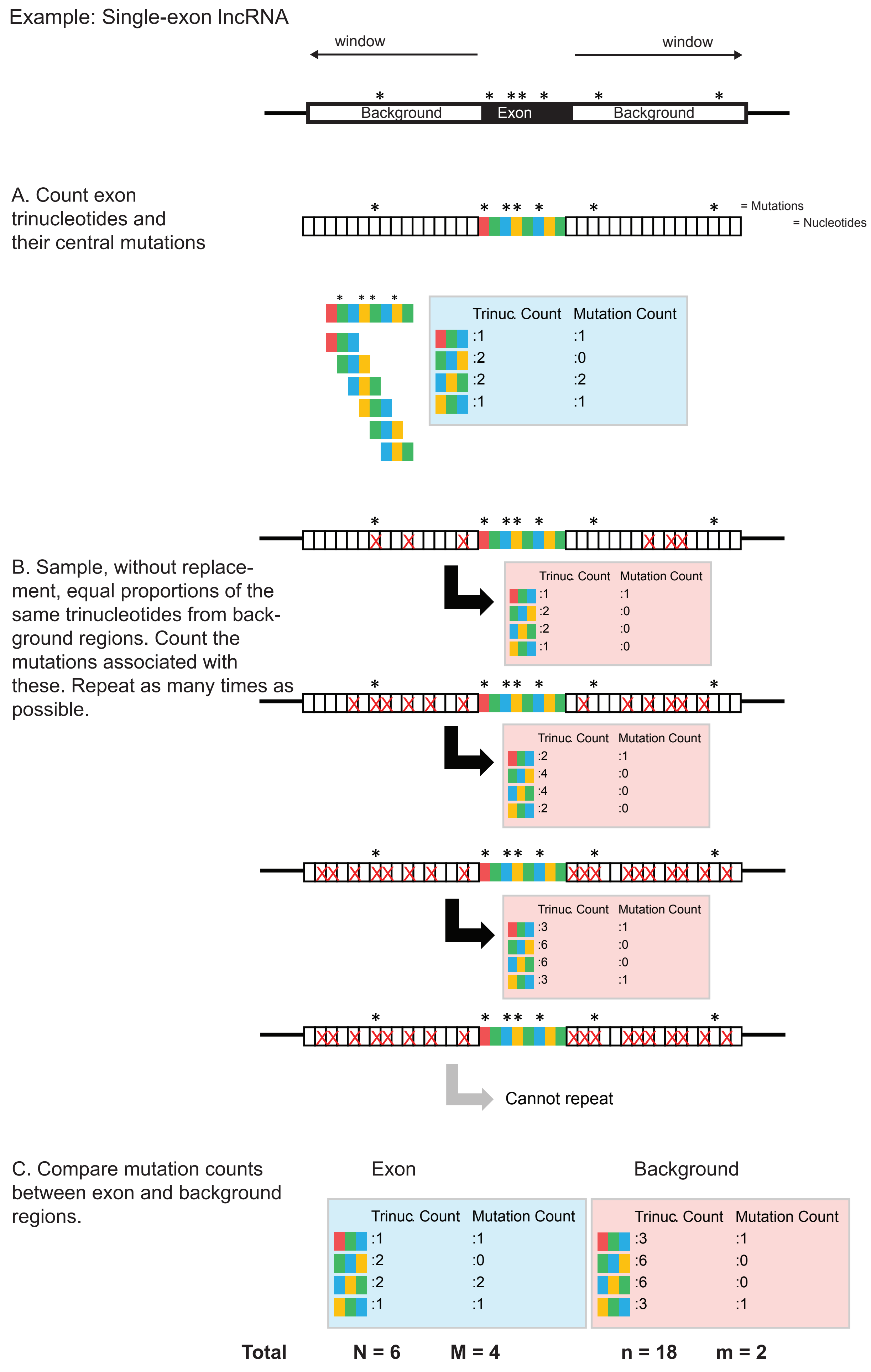

Supplementary Figure S2

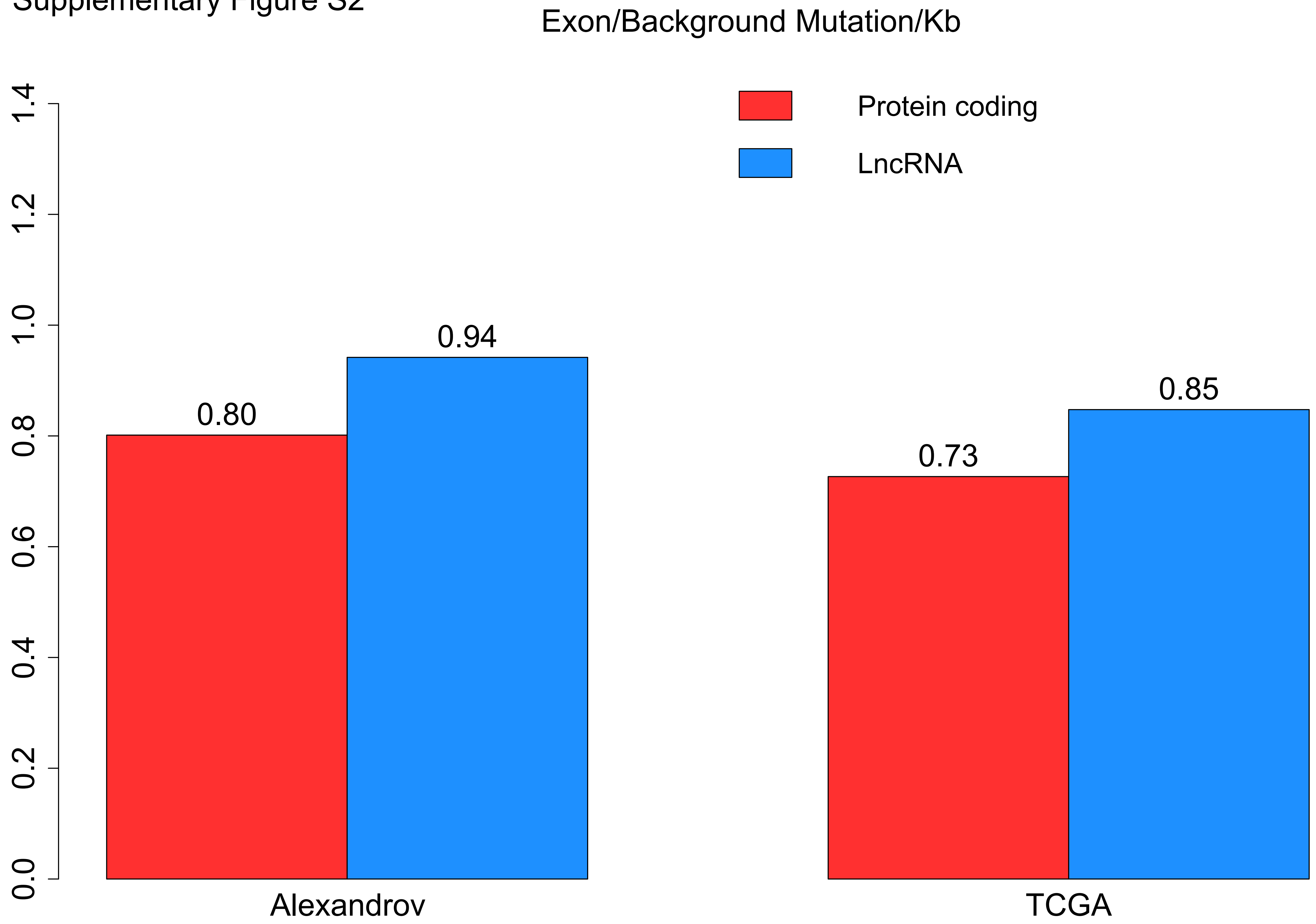

Supplementary Figure S3

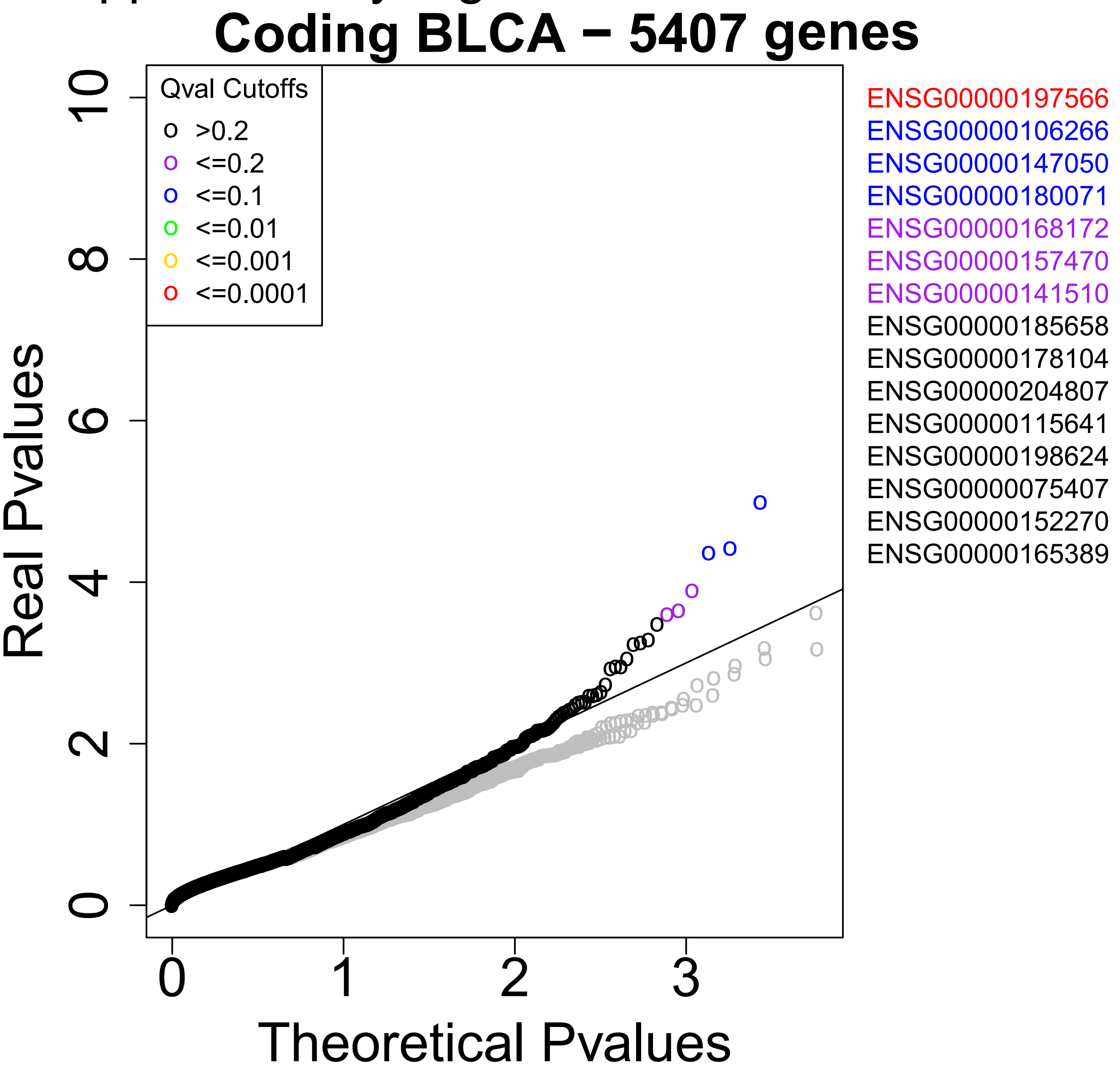

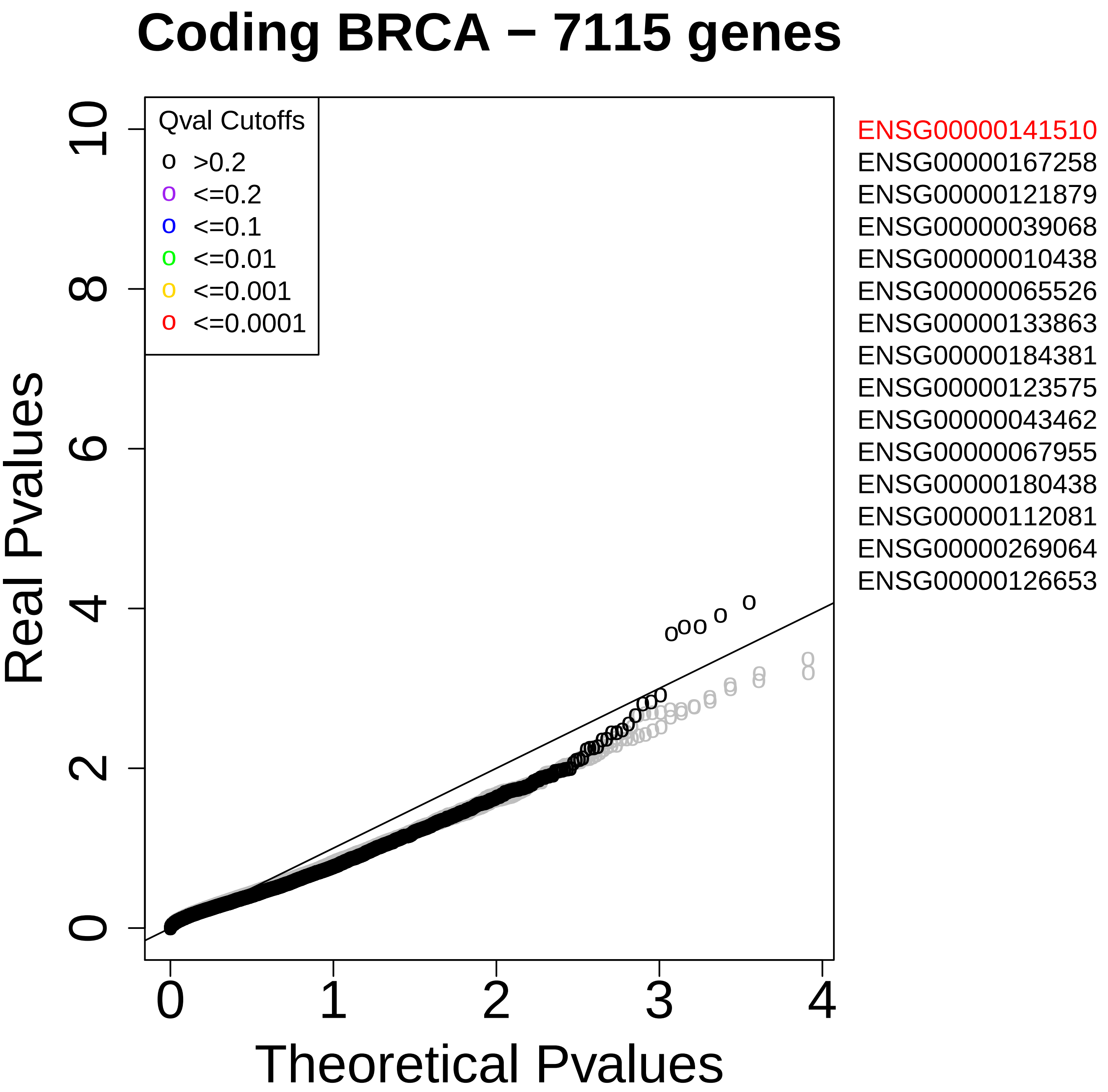
Coding BRCA – 7115 genes
Qval Cutoffs
o >0.2
o <=0.2
o <=0.1
o <=0.01
o <=0.001
o <=0.0001
Real Pvalues
Theoretical Pvalues
0
2
4
6
8
10
0
1
2
3
4
ENSG00000141510
ENSG00000167258
ENSG00000121879
ENSG00000039068
ENSG00000010438
ENSG00000065526
ENSG00000133863
ENSG00000184381
ENSG00000123575
ENSG00000043462
ENSG00000067955
ENSG00000180438
ENSG00000112081
ENSG00000269064
ENSG00000126653

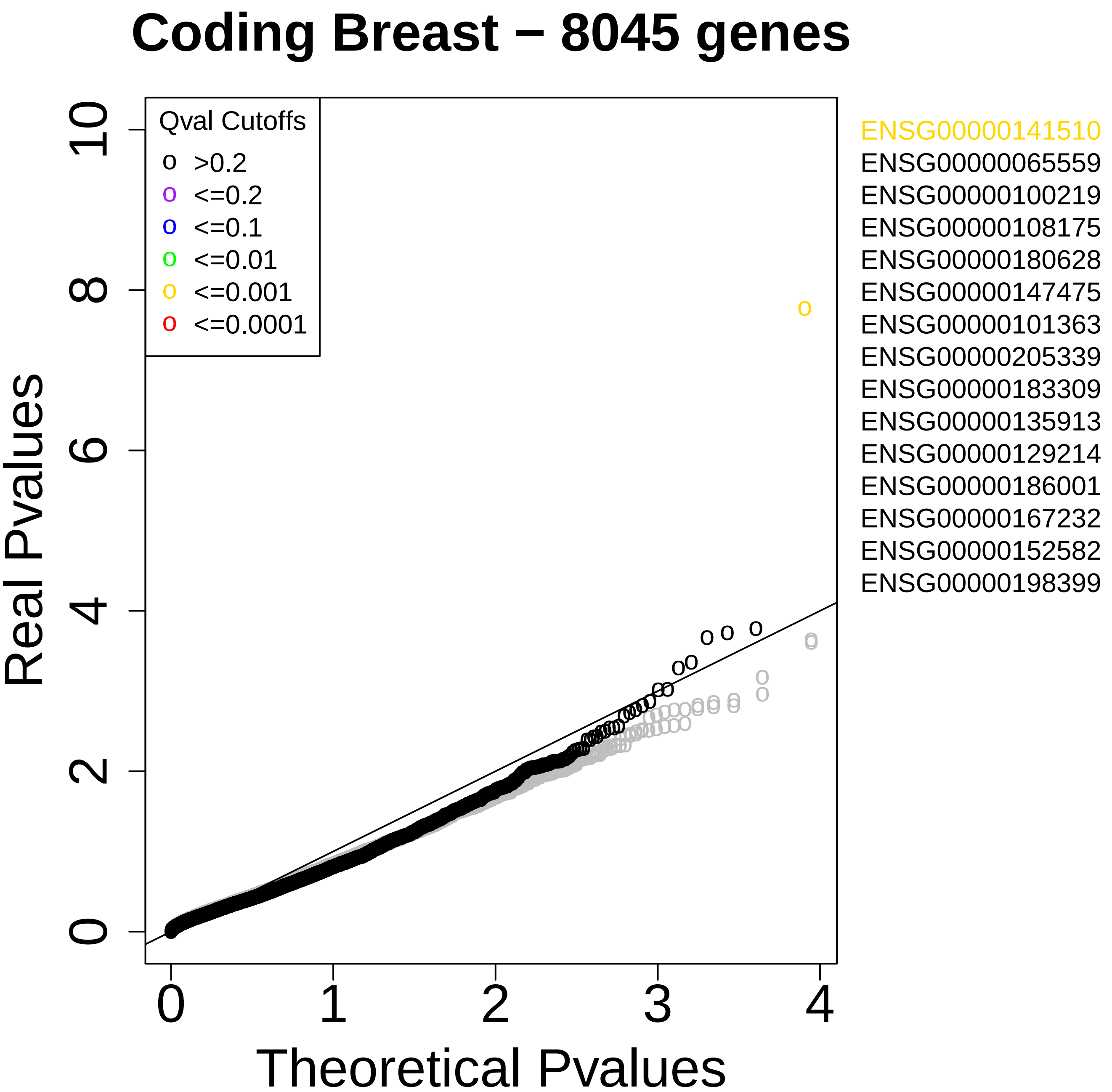
Coding Breast − 8045 genes
Qval Cutoffs
o >0.2
o <=0.2
o <=0.1
o <=0.01
o <=0.001
o <=0.0001
Real Pvalues
Theoretical Pvalues
0
2
4
6
8
10
0
1
2
3
4
ENSG00000141510
ENSG00000065559
ENSG00000100219
ENSG00000108175
ENSG00000180628
ENSG00000147475
ENSG00000101363
ENSG00000205339
ENSG00000183309
ENSG00000135913
ENSG00000129214
ENSG00000186001
ENSG00000167232
ENSG00000152582
ENSG00000198399

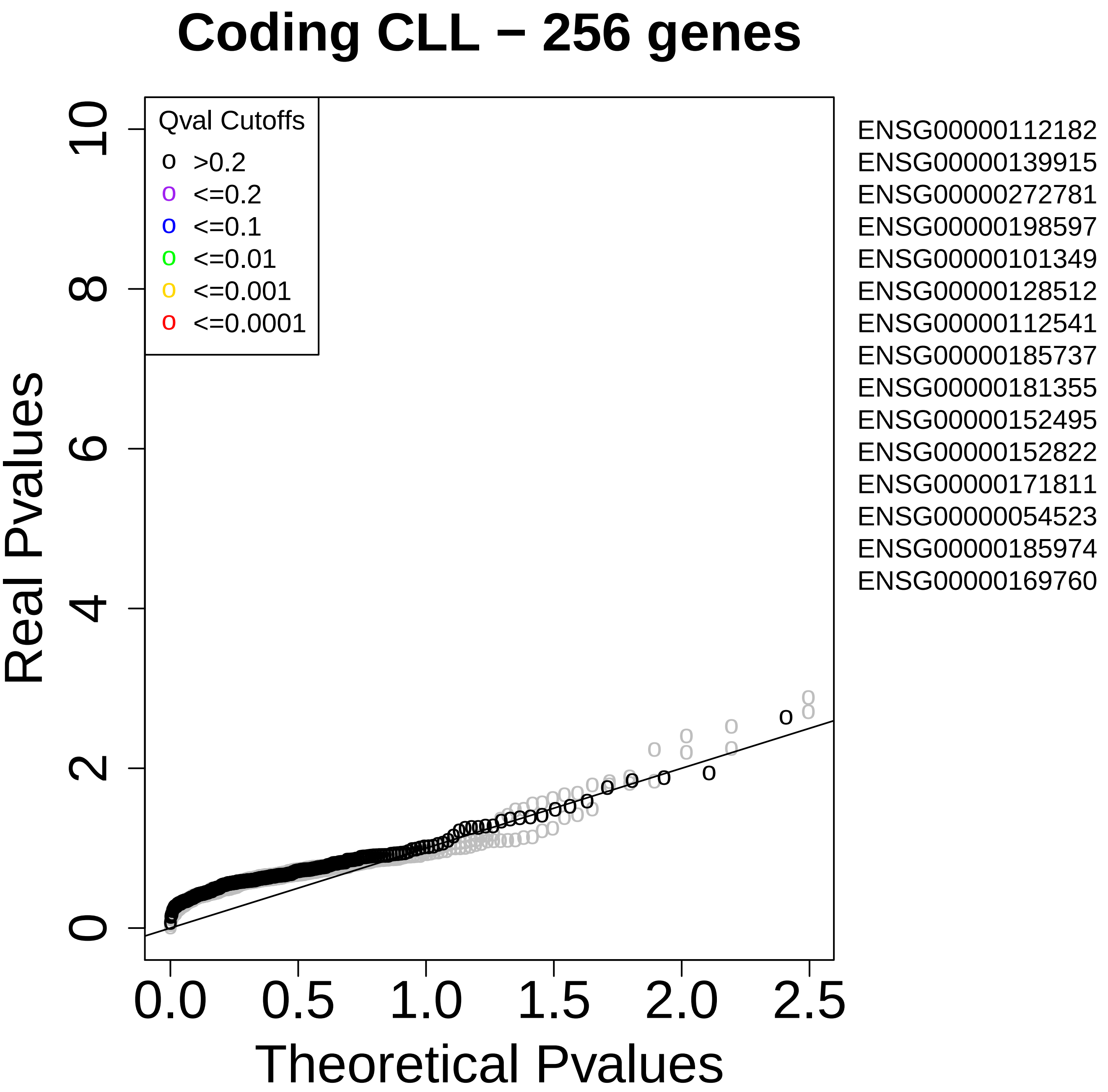
Coding CLL − 256 genes
Qval Cutoffs
o >0.2
o <=0.2
o <=0.1
o <=0.01
o <=0.001
o <=0.0001
Real Pvalues
Theoretical Pvalues
0
2
4
6
8
10
0.0
0.5
1.0
1.5
2.0
2.5
ENSG00000112182
ENSG00000139915
ENSG00000272781
ENSG00000198597
ENSG00000101349
ENSG00000128512
ENSG00000112541
ENSG00000185737
ENSG00000181355
ENSG00000152495
ENSG00000152822
ENSG00000171811
ENSG00000054523
ENSG00000185974
ENSG00000169760

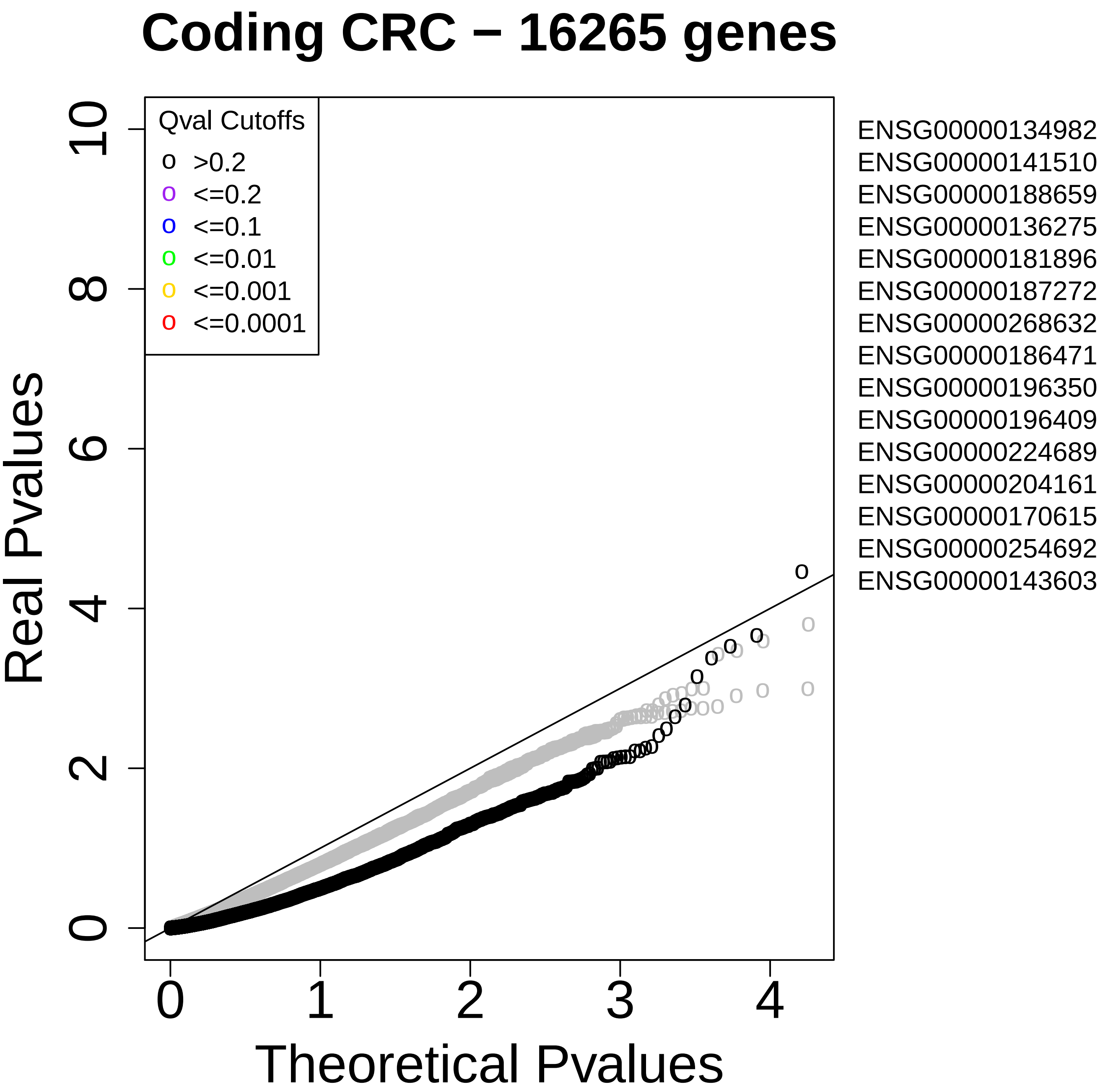
Coding CRC – 16265 genes
Qval Cutoffs
o >0.2
o <=0.2
o <=0.1
o <=0.01
o <=0.001
o <=0.0001
Real Pvalues
Theoretical Pvalues
0
2
4
6
8
10
0
1
2
3
4
ENSG00000134982
ENSG00000141510
ENSG00000188659
ENSG00000136275
ENSG00000181896
ENSG00000187272
ENSG00000268632
ENSG00000186471
ENSG00000196350
ENSG00000196409
ENSG00000224689
ENSG00000204161
ENSG00000170615
ENSG00000254692
ENSG00000143603

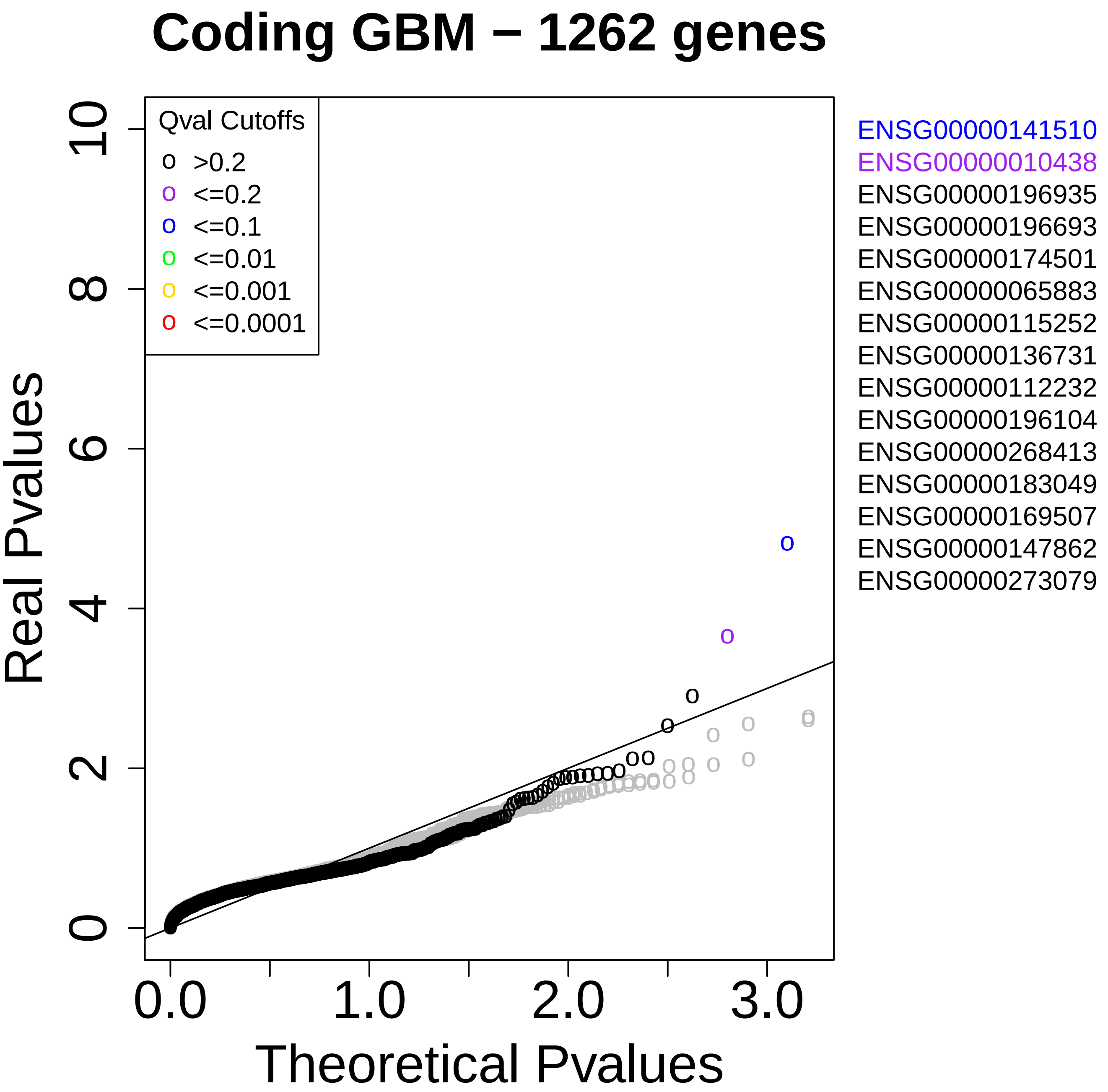
Coding GBM – 1262 genes
Qval Cutoffs
o >0.2
o <=0.2
o <=0.1
o <=0.01
o <=0.001
o <=0.0001
Real Pvalues
Theoretical Pvalues
0
2
4
6
8
10
0.0
1.0
2.0
3.0
ENSG00000141510
ENSG00000010438
ENSG00000196935
ENSG00000196693
ENSG00000174501
ENSG00000065883
ENSG00000115252
ENSG00000136731
ENSG00000112232
ENSG00000196104
ENSG00000268413
ENSG00000183049
ENSG00000169507
ENSG00000147862
ENSG00000273079

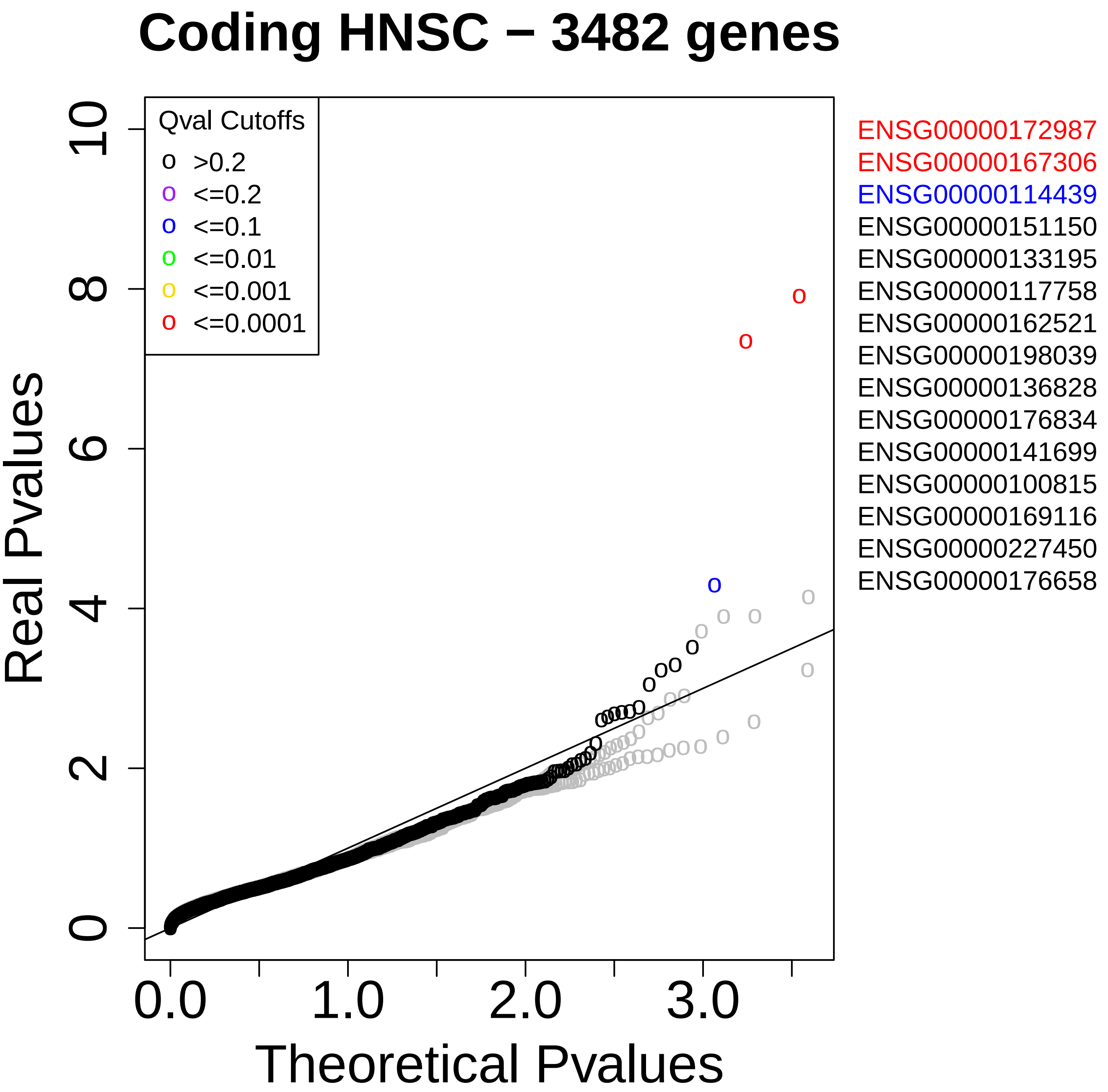

Coding HNSC − 3482 genes
Qval Cutoffs
o >0.2
o <=0.2
o <=0.1
o <=0.01
o <=0.001
o <=0.0001
Real Pvalues
Theoretical Pvalues
0
2
4
6
8
10
0.0
1.0
2.0
3.0
ENSG00000172987
ENSG00000167306
ENSG00000114439
ENSG00000151150
ENSG00000133195
ENSG00000117758
ENSG00000162521
ENSG00000198039
ENSG00000136828
ENSG00000176834
ENSG00000141699
ENSG00000100815
ENSG00000169116
ENSG00000227450
ENSG00000176658

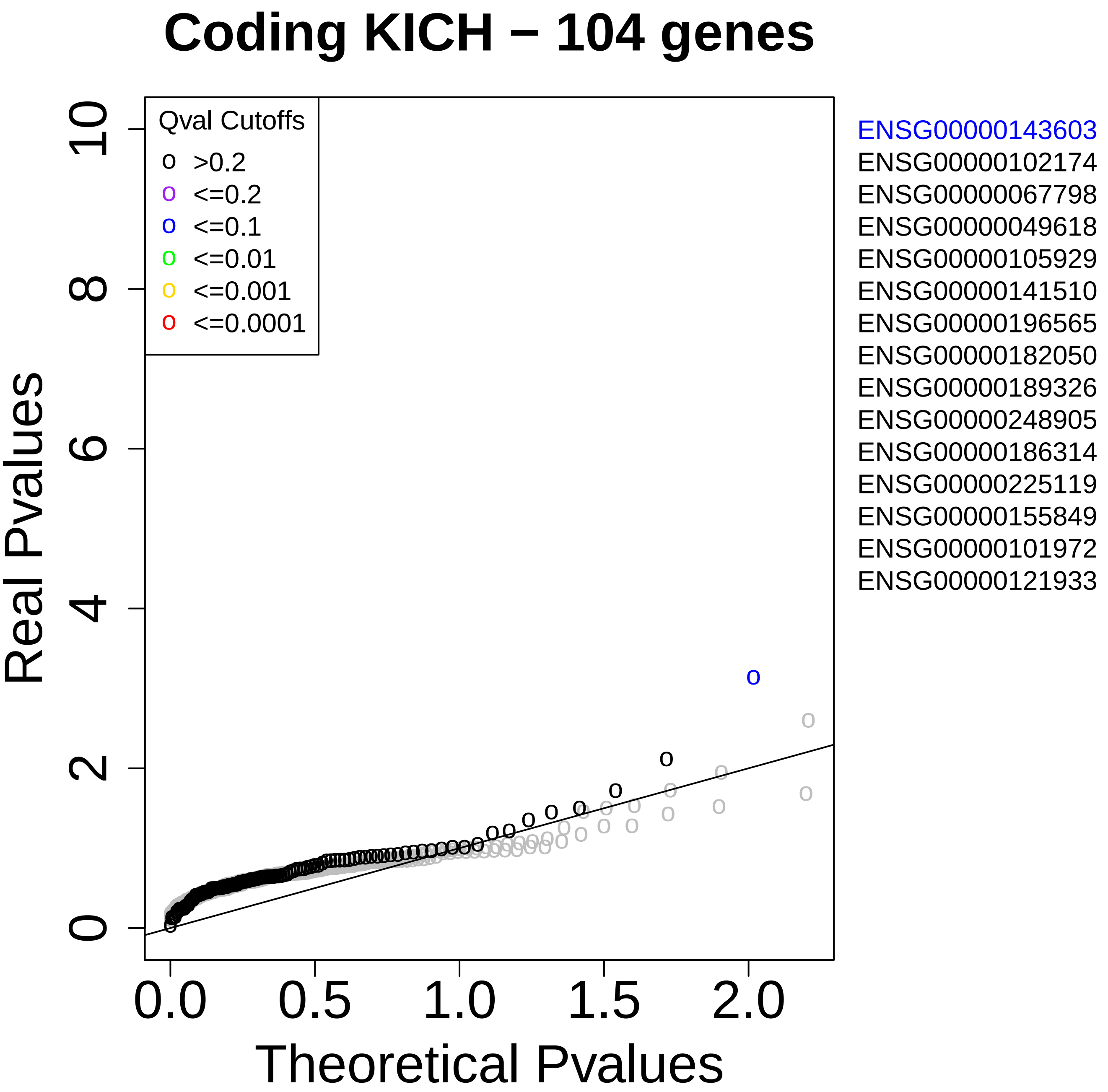

Coding KICH – 104 genes
Qval Cutoffs
o >0.2
o <=0.2
o <=0.1
o <=0.01
o <=0.001
o <=0.0001
Real Pvalues
Theoretical Pvalues
0
2
4
6
8
10
0.0
0.5
1.0
1.5
2.0
ENSG00000143603
ENSG00000102174
ENSG00000067798
ENSG00000049618
ENSG00000105929
ENSG00000141510
ENSG00000196565
ENSG00000182050
ENSG00000189326
ENSG00000248905
ENSG00000186314
ENSG00000225119
ENSG00000155849
ENSG00000101972
ENSG00000121933

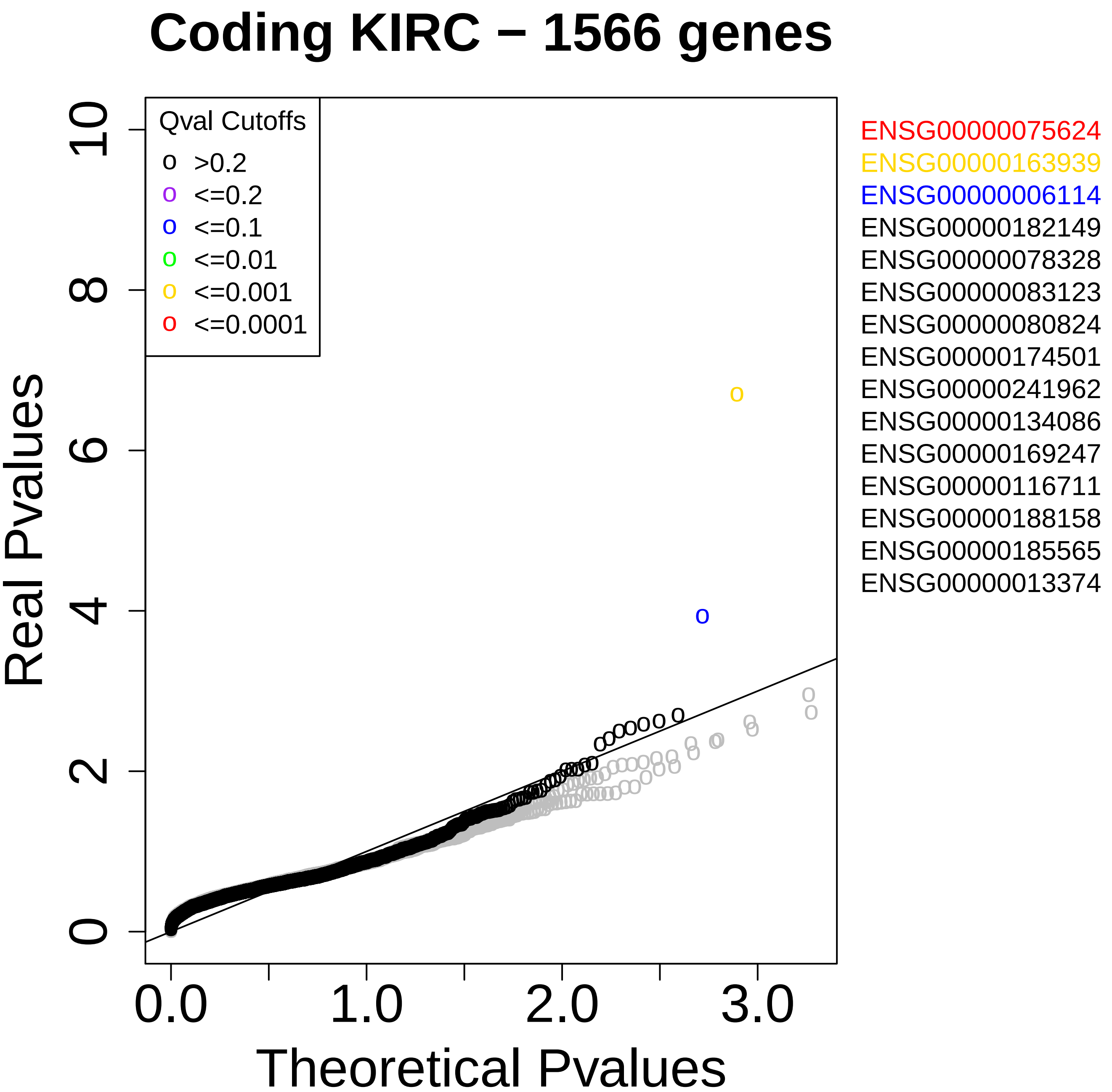

Coding KIRC – 1566 genes
Qval Cutoffs
o >0.2
o <=0.2
o <=0.1
o <=0.01
o <=0.001
o <=0.0001
ENSG00000075624
ENSG00000163939
ENSG00000006114
ENSG00000182149
ENSG00000078328
ENSG00000083123
ENSG00000080824
ENSG00000174501
ENSG00000241962
ENSG00000134086
ENSG00000169247
ENSG00000116711
ENSG00000188158
ENSG00000185565
ENSG00000013374
Real Pvalues
0
2
4
6
8
10
Theoretical Pvalues
0.0
1.0
2.0
3.0

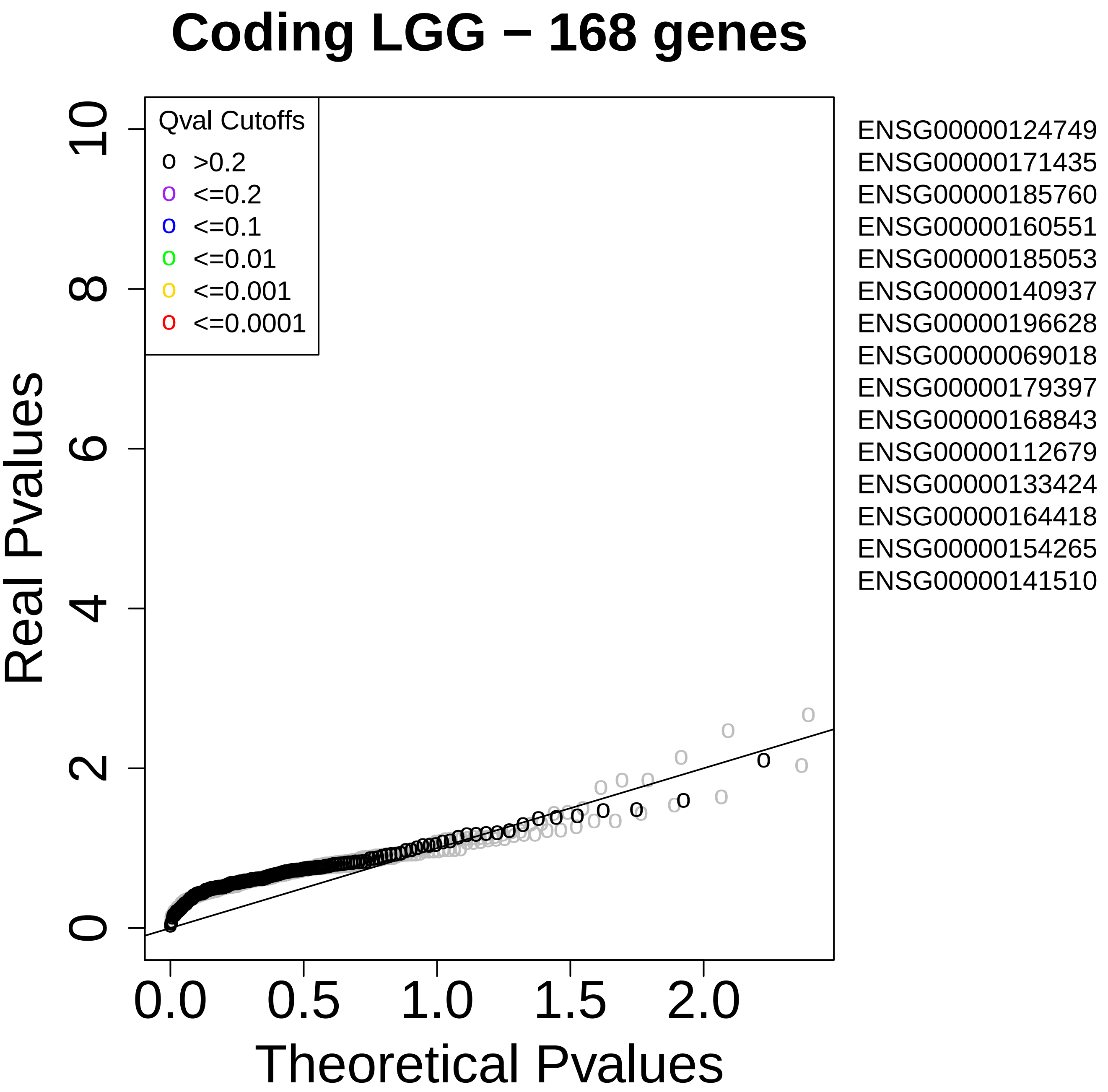
Coding LGG – 168 genes
Qval Cutoffs
o >0.2
o <=0.2
o <=0.1
o <=0.01
o <=0.001
o <=0.0001
Real Pvalues
0
2
4
6
8
10
0.0
0.5
1.0
1.5
2.0
Theoretical Pvalues
ENSG00000124749
ENSG00000171435
ENSG00000185760
ENSG00000160551
ENSG00000185053
ENSG00000140937
ENSG00000196628
ENSG00000069018
ENSG00000179397
ENSG00000168843
ENSG00000112679
ENSG00000133424
ENSG00000164418
ENSG00000154265
ENSG00000141510

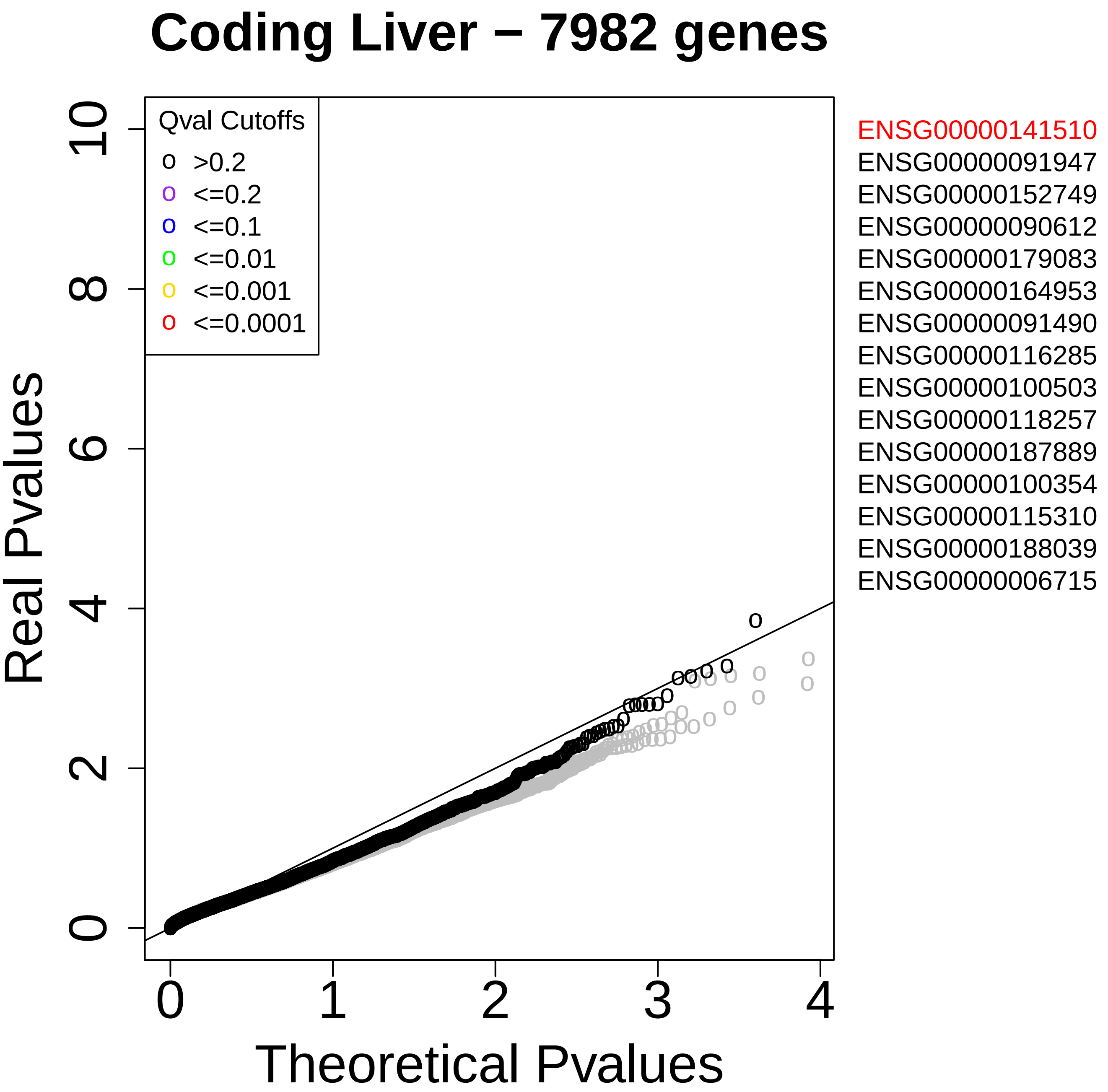
Coding Liver − 7982 genes
Qval Cutoffs
o >0.2
o <=0.2
o <=0.1
o <=0.01
o <=0.001
o <=0.0001
Real Pvalues
Theoretical Pvalues
0
2
4
6
8
10
0
1
2
3
4
ENSG00000141510
ENSG00000091947
ENSG00000152749
ENSG00000090612
ENSG00000179083
ENSG00000164953
ENSG00000091490
ENSG00000116285
ENSG00000100503
ENSG00000118257
ENSG00000187889
ENSG00000100354
ENSG00000115310
ENSG00000188039
ENSG00000006715

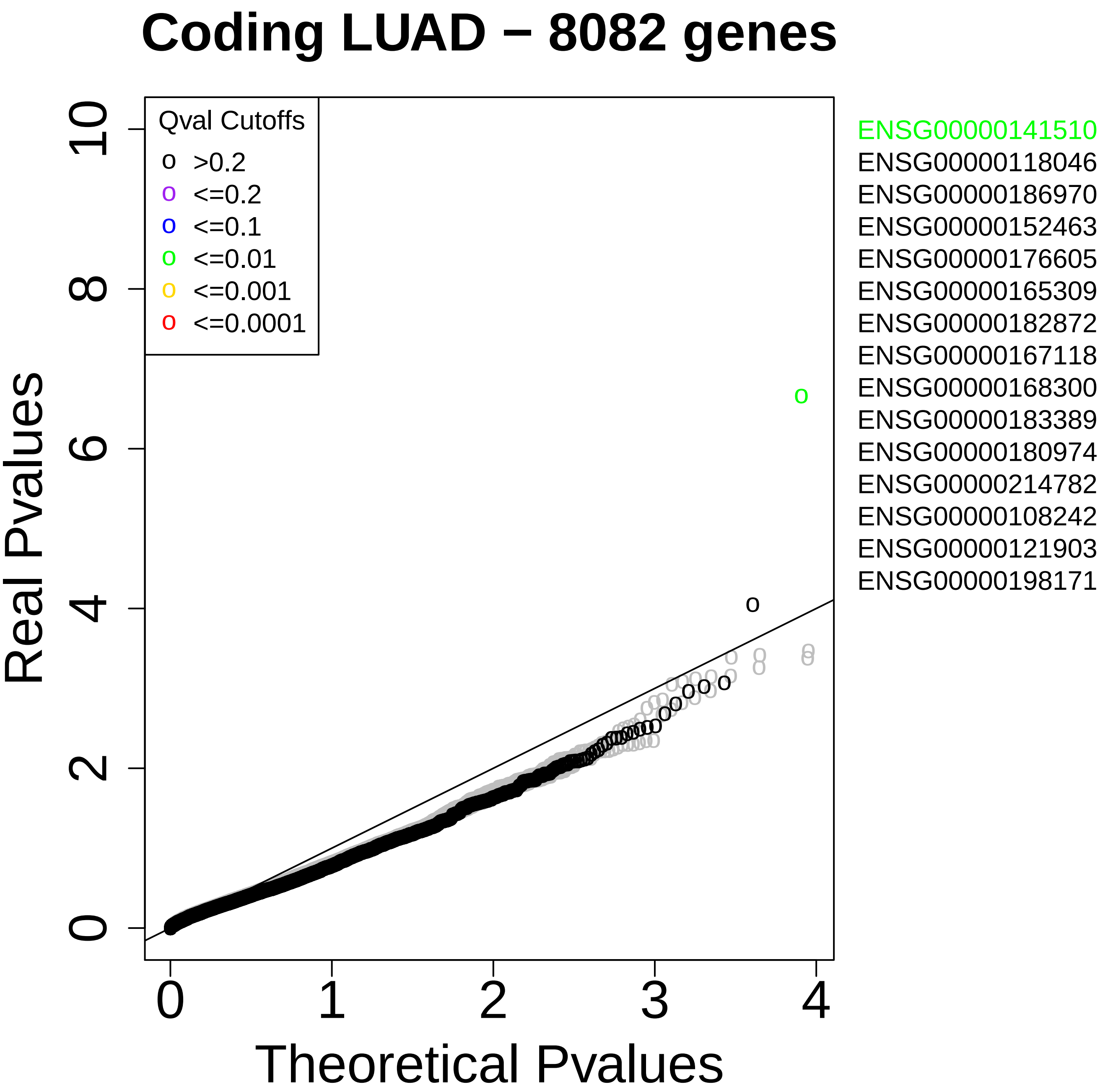
Coding LUAD – 8082 genes
Qval Cutoffs
o >0.2
o <=0.2
o <=0.1
o <=0.01
o <=0.001
o <=0.0001
Real Pvalues
Theoretical Pvalues
0
2
4
6
8
10
0
1
2
3
4
ENSG00000141510
ENSG00000118046
ENSG00000186970
ENSG00000152463
ENSG00000176605
ENSG00000165309
ENSG00000182872
ENSG00000167118
ENSG00000168300
ENSG00000183389
ENSG00000180974
ENSG00000214782
ENSG00000108242
ENSG00000121903
ENSG00000198171

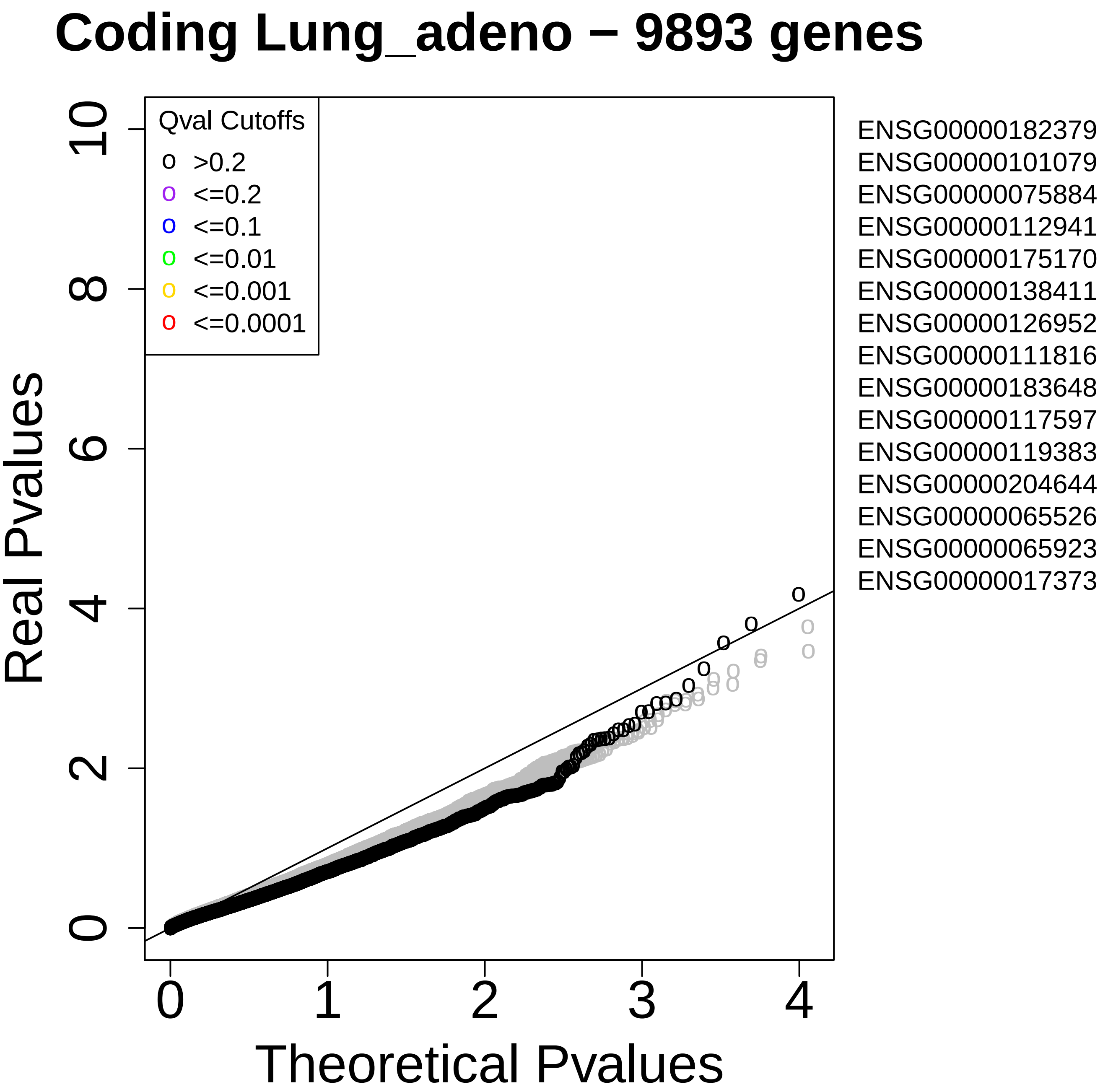

Coding Lung_adeno − 9893 genes
Qval Cutoffs
o >0.2
o <=0.2
o <=0.1
o <=0.01
o <=0.001
o <=0.0001
Real Pvalues
Theoretical Pvalues
0
2
4
6
8
10
0
1
2
3
4
ENSG00000182379
ENSG00000101079
ENSG00000075884
ENSG00000112941
ENSG00000175170
ENSG00000138411
ENSG00000126952
ENSG00000111816
ENSG00000183648
ENSG00000117597
ENSG00000119383
ENSG00000204644
ENSG00000065526
ENSG00000065923
ENSG00000017373

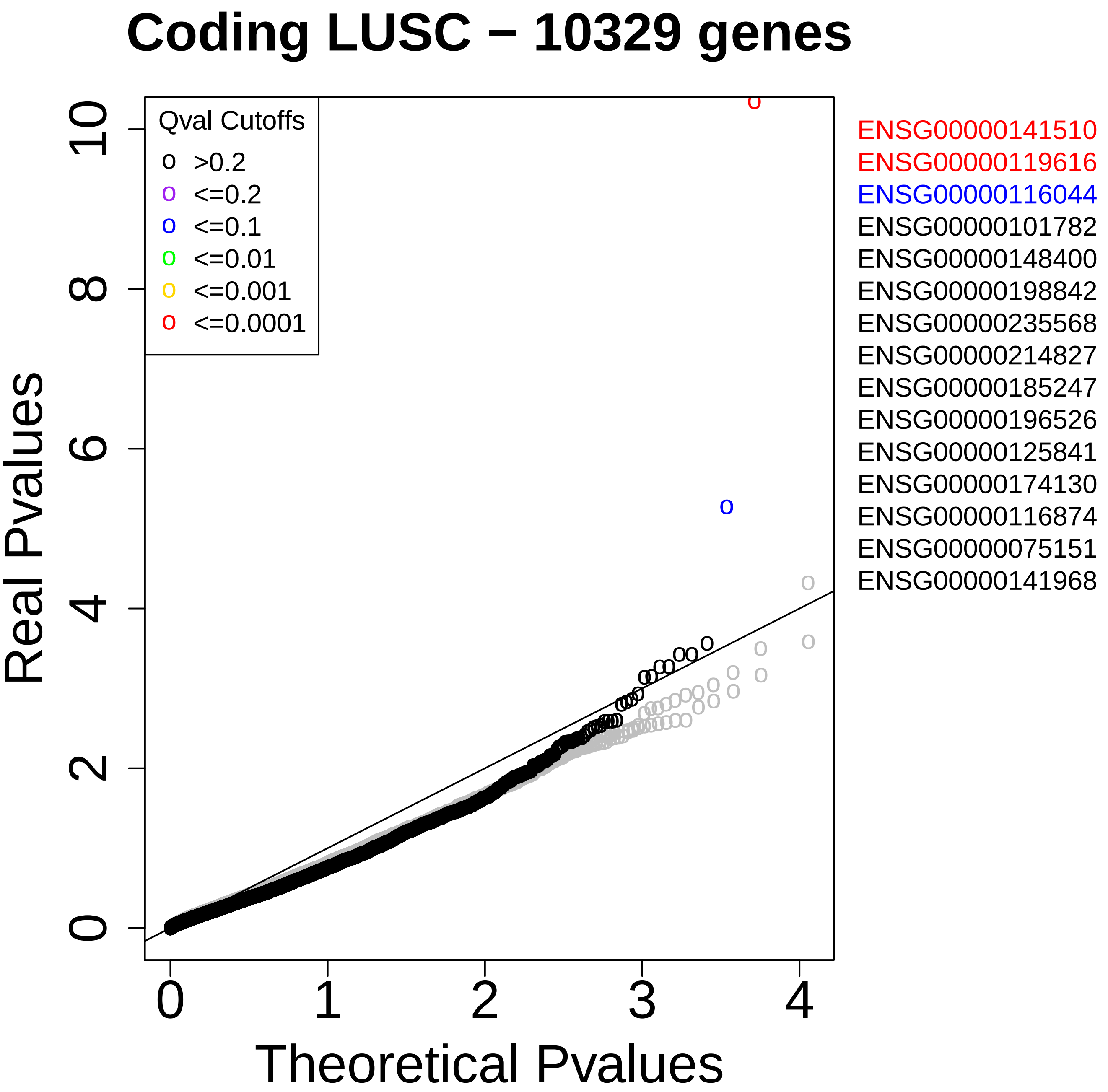
Coding LUSC − 10329 genes
Qval Cutoffs
o >0.2
o <=0.2
o <=0.1
o <=0.01
o <=0.001
o <=0.0001
Real Pvalues
Theoretical Pvalues
0
2
4
6
8
10
0
1
2
3
4
ENSG00000141510
ENSG00000119616
ENSG00000116044
ENSG00000101782
ENSG00000148400
ENSG00000198842
ENSG00000235568
ENSG00000214827
ENSG00000185247
ENSG00000196526
ENSG00000125841
ENSG00000174130
ENSG00000116874
ENSG00000075151
ENSG00000141968

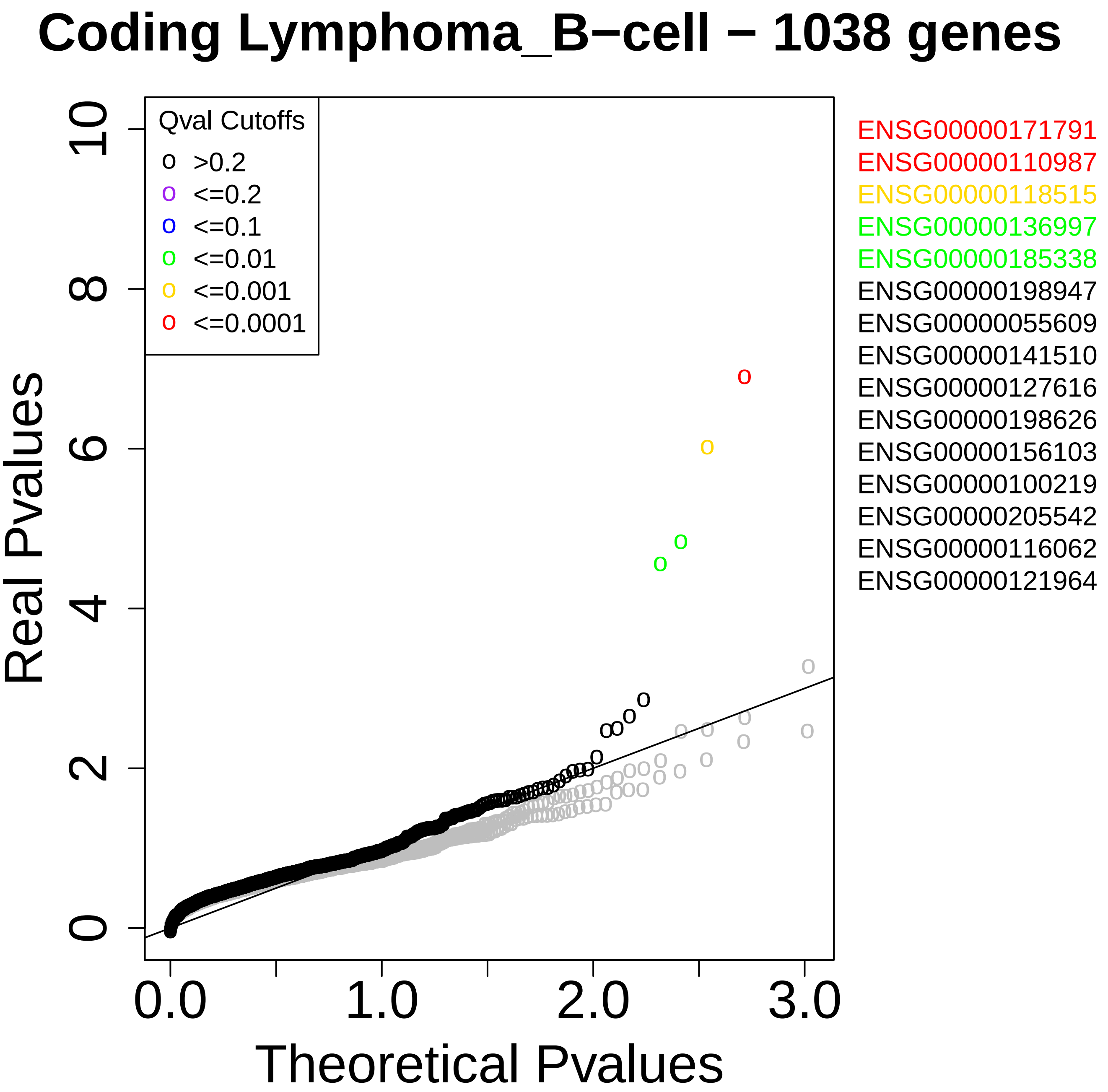
Coding Lymphoma_B−cell − 1038 genes
Qval Cutoffs
>0.2
<=0.2
<=0.1
<=0.01
<=0.001
<=0.0001
Real Pvalues
Theoretical Pvalues
0
2
4
6
8
10
0.0
1.0
2.0
3.0
ENSG00000171791
ENSG00000110987
ENSG00000118515
ENSG00000136997
ENSG00000185338
ENSG00000198947
ENSG00000055609
ENSG00000141510
ENSG00000127616
ENSG00000198626
ENSG00000156103
ENSG00000100219
ENSG00000205542
ENSG00000116062
ENSG00000121964

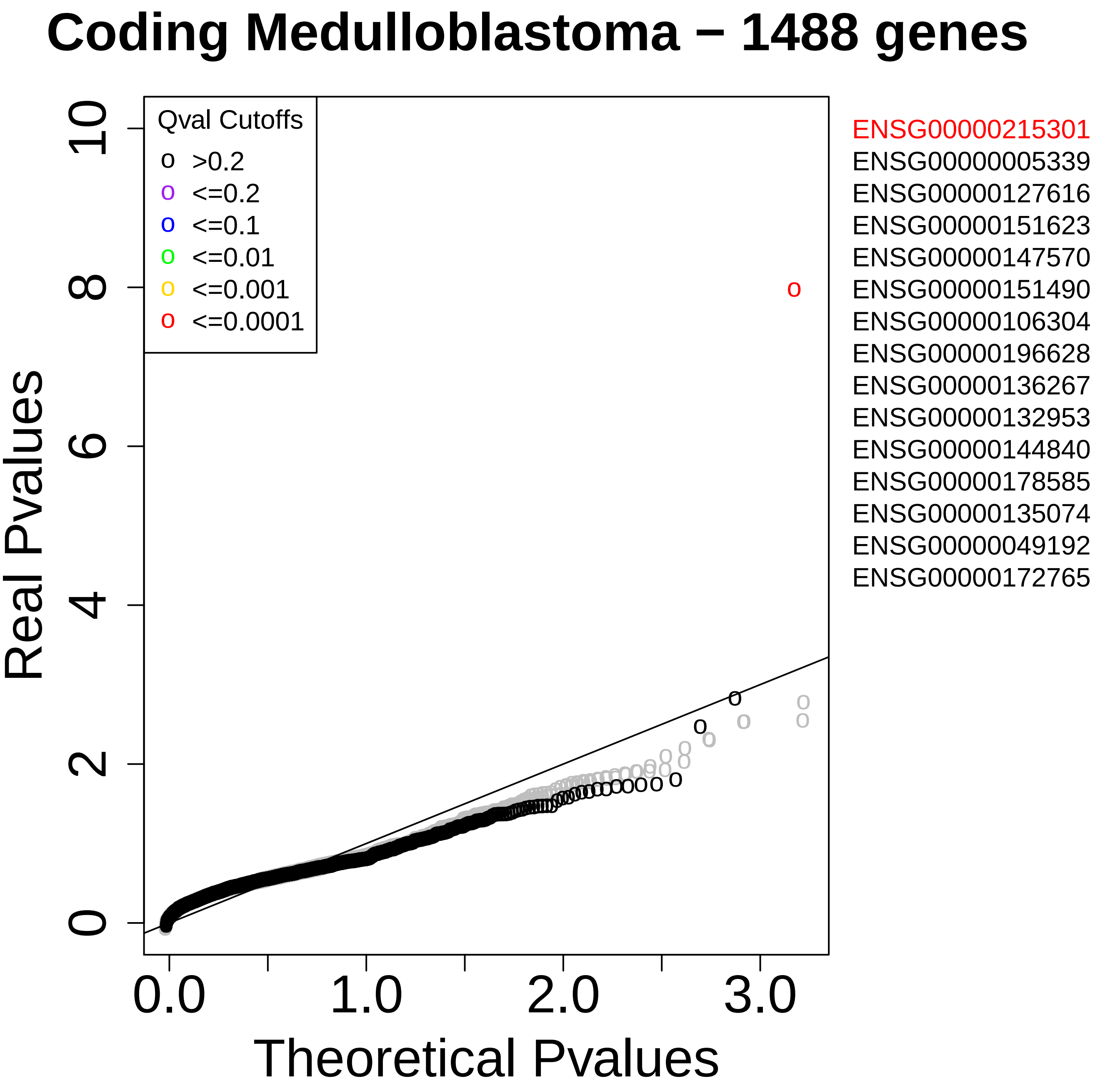
Coding Medulloblastoma − 1488 genes
Qval Cutoffs
o >0.2
o <=0.2
o <=0.1
o <=0.01
o <=0.001
o <=0.0001
Real Pvalues
Theoretical Pvalues
0
2
4
6
8
10
0.0
1.0
2.0
3.0
ENSG00000215301
ENSG00000005339
ENSG00000127616
ENSG00000151623
ENSG00000147570
ENSG00000151490
ENSG00000106304
ENSG00000196628
ENSG00000136267
ENSG00000132953
ENSG00000144840
ENSG00000178585
ENSG00000135074
ENSG00000049192
ENSG00000172765

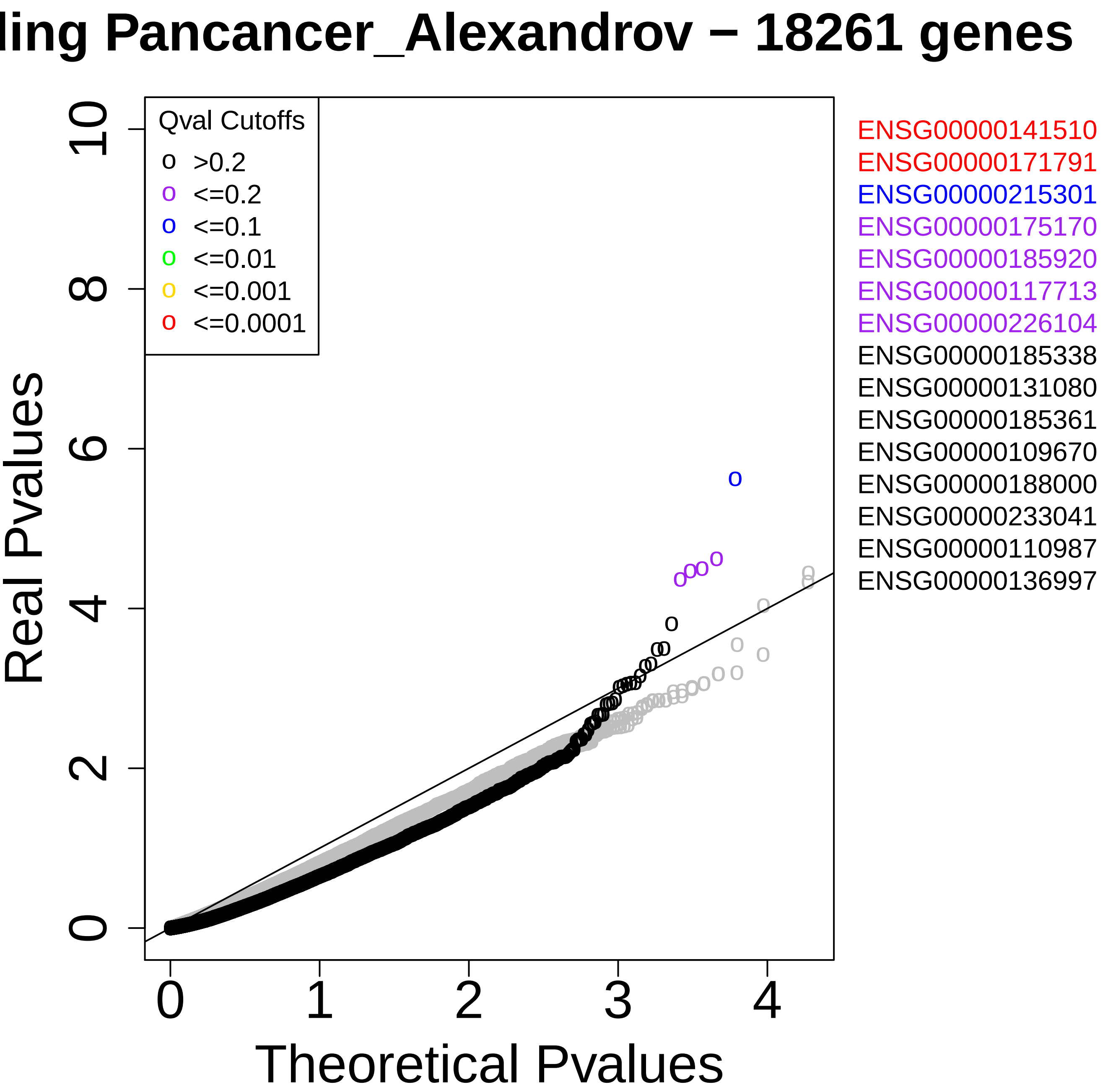
ling Pancancer_Alexandrov − 18261 genes
Qval Cutoffs
o >0.2
o <=0.2
o <=0.1
o <=0.01
o <=0.001
o <=0.0001
Real Pvalues
Theoretical Pvalues
0
2
4
6
8
10
0
1
2
3
4
ENSG00000141510
ENSG00000171791
ENSG00000215301
ENSG00000175170
ENSG00000185920
ENSG00000117713
ENSG00000226104
ENSG00000185338
ENSG00000131080
ENSG00000185361
ENSG00000109670
ENSG00000188000
ENSG00000233041
ENSG00000110987
ENSG00000136997

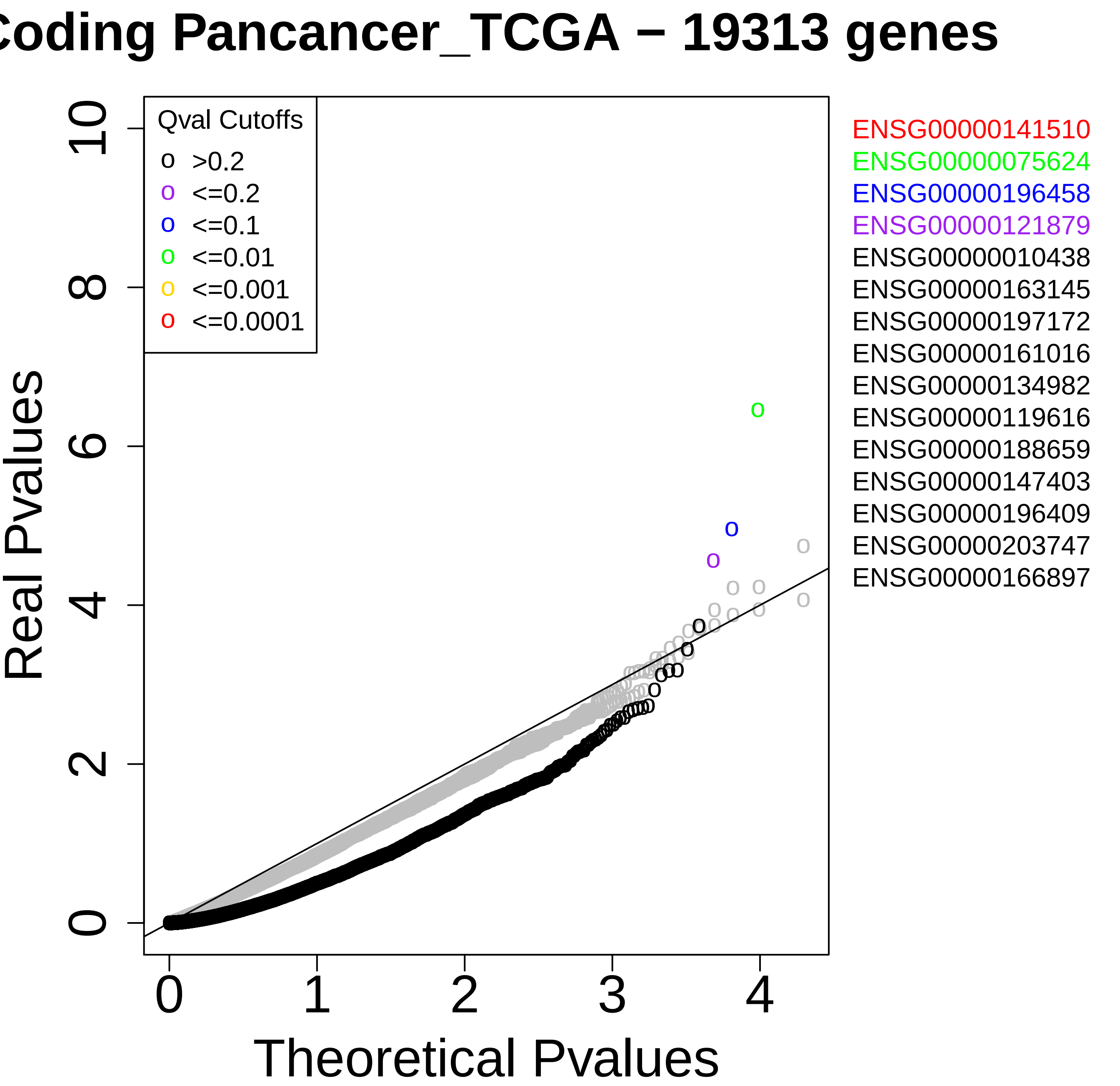
Coding Pancancer_TCGA – 19313 genes
Qval Cutoffs
o >0.2
o <=0.2
o <=0.1
o <=0.01
o <=0.001
o <=0.0001
Real Pvalues
Theoretical Pvalues
0
2
4
6
8
10
0
1
2
3
4
ENSG00000141510
ENSG00000075624
ENSG00000196458
ENSG00000121879
ENSG00000010438
ENSG00000163145
ENSG00000197172
ENSG00000161016
ENSG00000134982
ENSG00000119616
ENSG00000188659
ENSG00000147403
ENSG00000196409
ENSG00000203747
ENSG00000166897

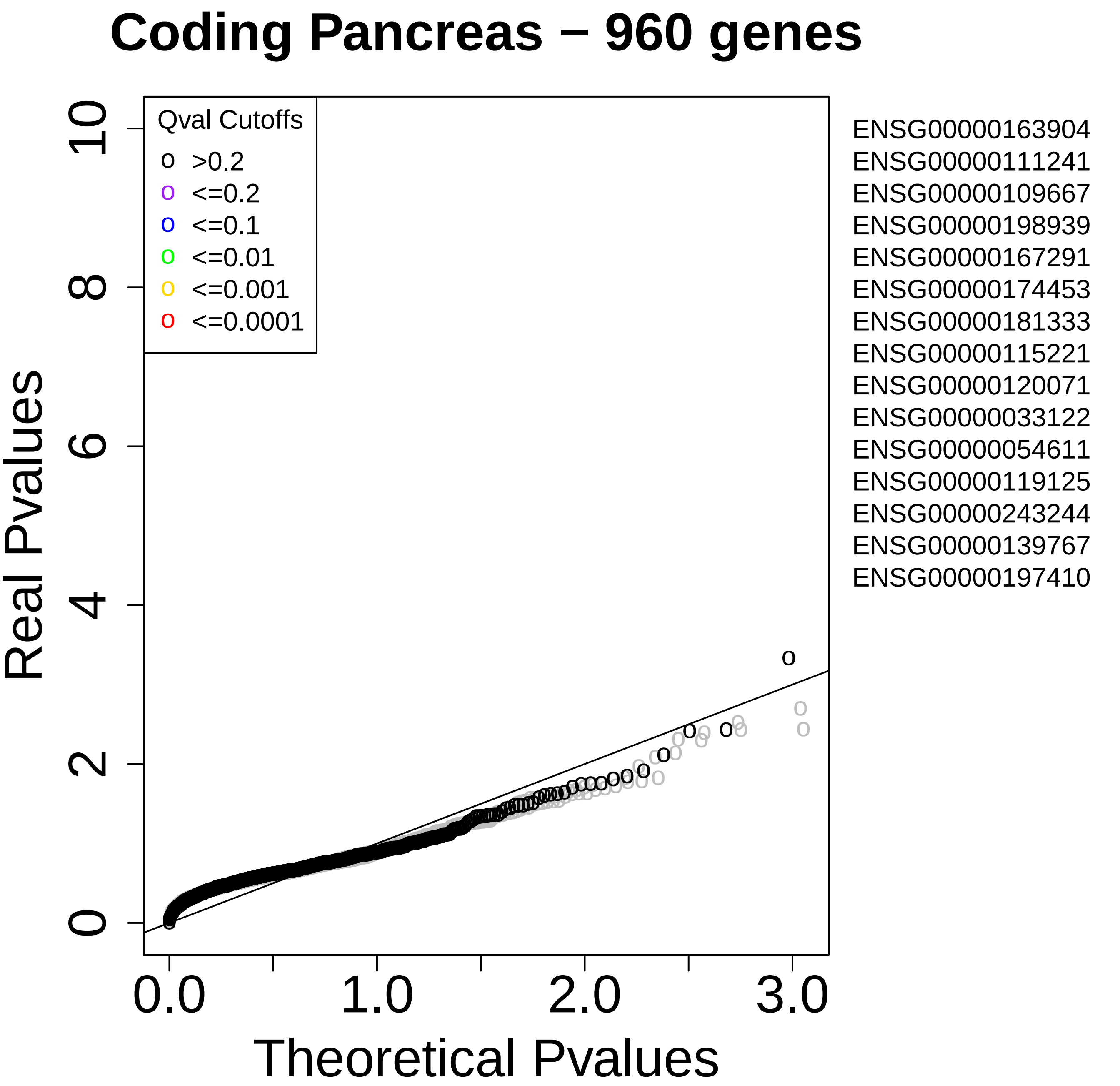
Coding Pancreas − 960 genes
Qval Cutoffs
o >0.2
o <=0.2
o <=0.1
o <=0.01
o <=0.001
o <=0.0001
Real Pvalues
Theoretical Pvalues
0
2
4
6
8
10
0.0
1.0
2.0
3.0
ENSG00000163904
ENSG00000111241
ENSG00000109667
ENSG00000198939
ENSG00000167291
ENSG00000174453
ENSG00000181333
ENSG00000115221
ENSG00000120071
ENSG00000033122
ENSG00000054611
ENSG00000119125
ENSG00000243244
ENSG00000139767
ENSG00000197410

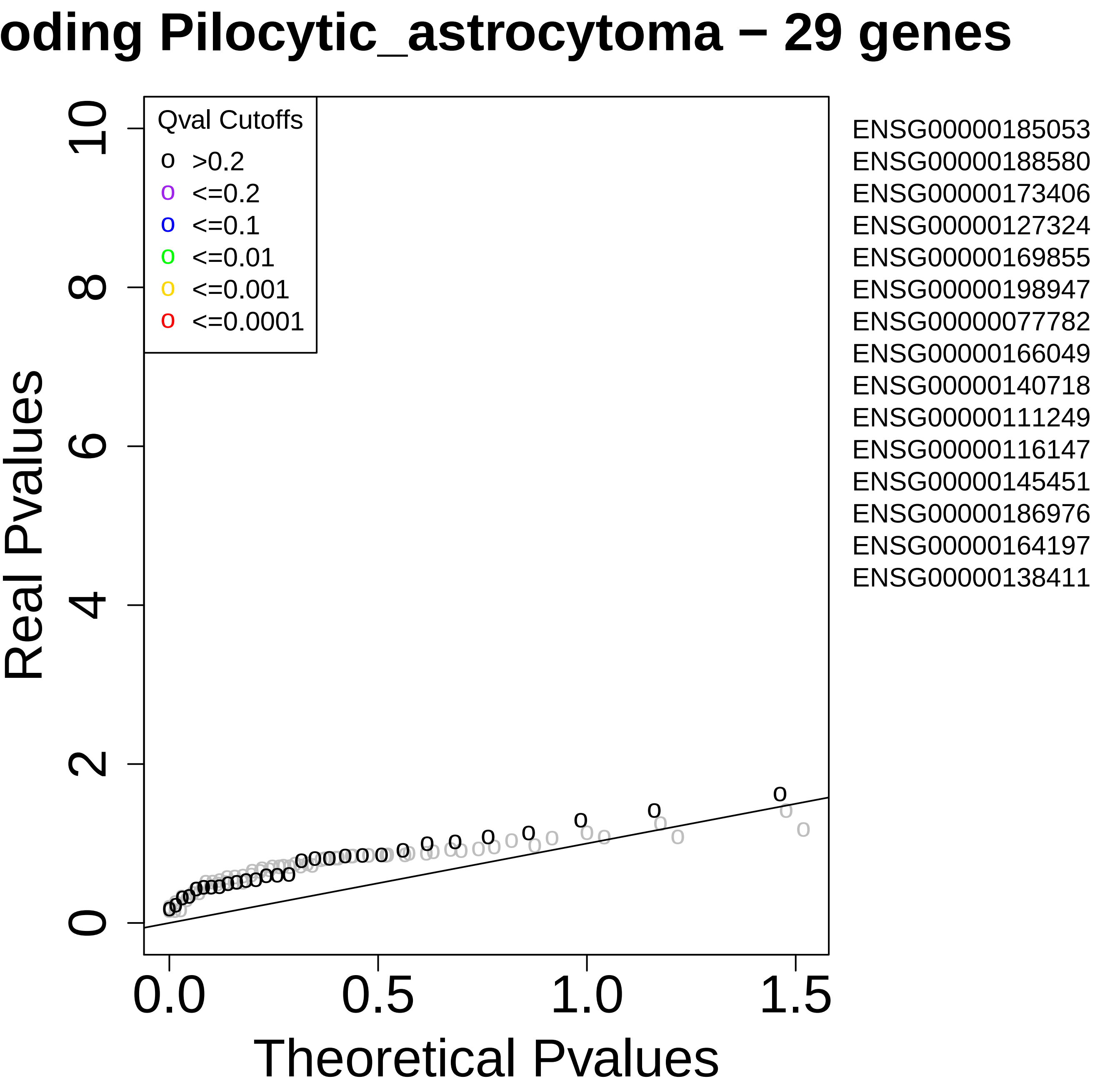

oding Pilocytic_astrocytoma − 29 genes
Qval Cutoffs
o >0.2
o <=0.2
o <=0.1
o <=0.01
o <=0.001
o <=0.0001
Real Pvalues
Theoretical Pvalues
0
2
4
6
8
10
0.0
0.5
1.0
1.5
ENSG00000185053
ENSG00000188580
ENSG00000173406
ENSG00000127324
ENSG00000169855
ENSG00000198947
ENSG00000077782
ENSG00000166049
ENSG00000140718
ENSG00000111249
ENSG00000116147
ENSG00000145451
ENSG00000186976
ENSG00000164197
ENSG00000138411

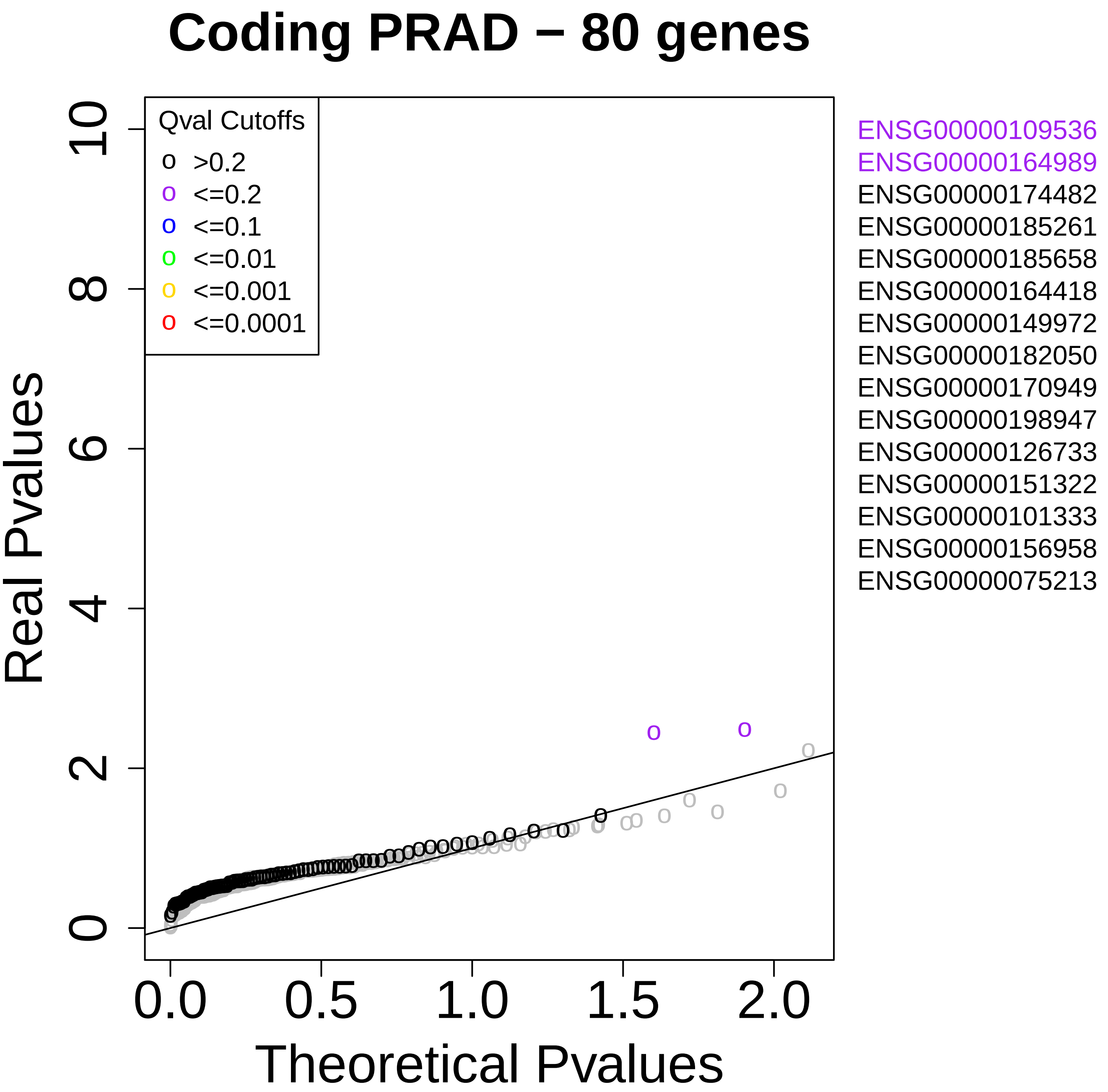

Coding PRAD − 80 genes
Qval Cutoffs
o >0.2
o <=0.2
o <=0.1
o <=0.01
o <=0.001
o <=0.0001
Real Pvalues
0
2
4
6
8
10
Theoretical Pvalues
0.0
0.5
1.0
1.5
2.0
ENSG00000109536
ENSG00000164989
ENSG00000174482
ENSG00000185261
ENSG00000185658
ENSG00000164418
ENSG00000149972
ENSG00000182050
ENSG00000170949
ENSG00000198947
ENSG00000126733
ENSG00000151322
ENSG00000101333
ENSG00000156958
ENSG00000075213

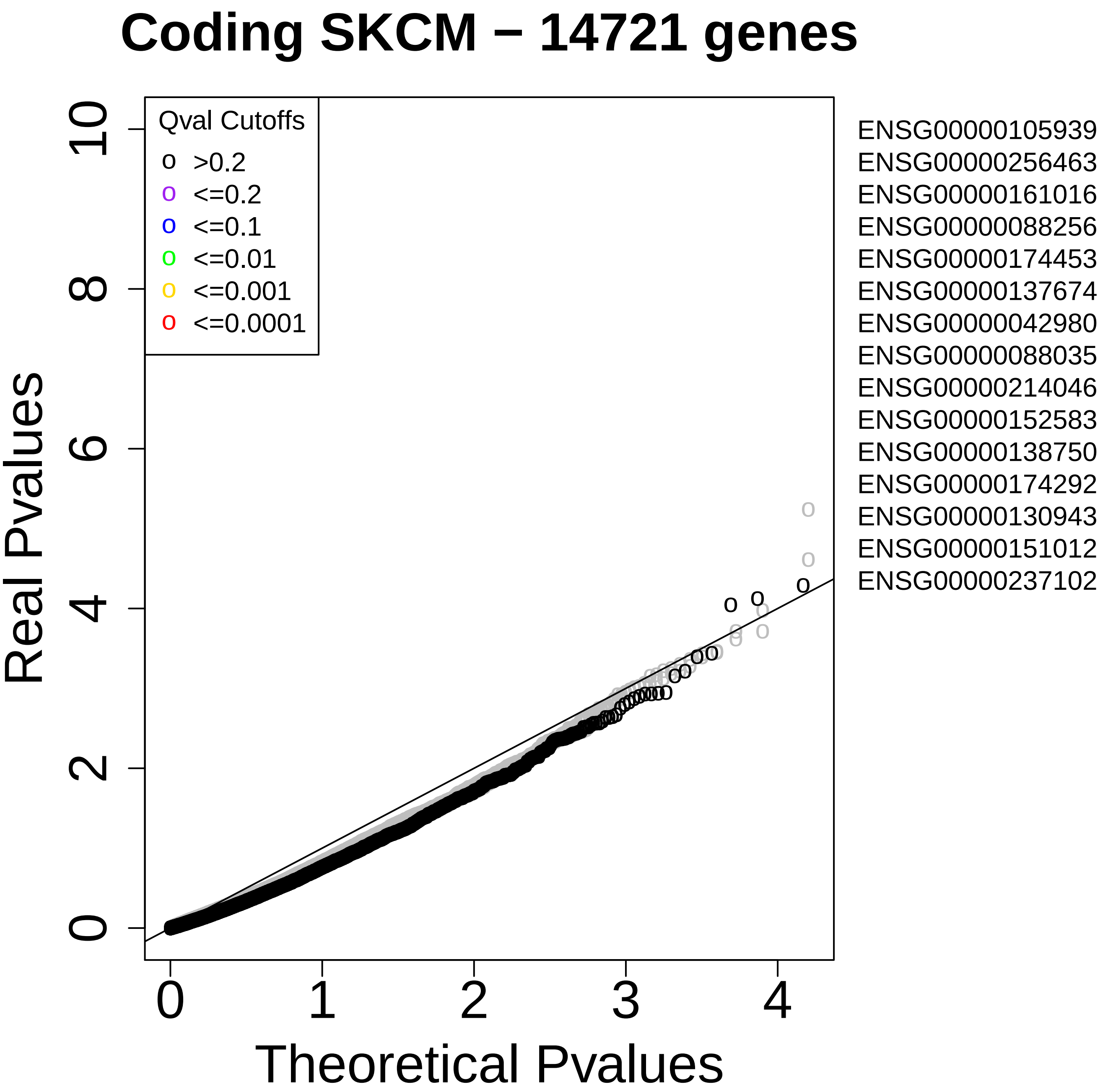

Coding SKCM – 14721 genes
Qval Cutoffs
o >0.2
o <=0.2
o <=0.1
o <=0.01
o <=0.001
o <=0.0001
Real Pvalues
Theoretical Pvalues
0
2
4
6
8
10
0
1
2
3
4
ENSG00000105939
ENSG00000256463
ENSG00000161016
ENSG00000088256
ENSG00000174453
ENSG00000137674
ENSG00000042980
ENSG00000088035
ENSG00000214046
ENSG00000152583
ENSG00000138750
ENSG00000174292
ENSG00000130943
ENSG00000151012
ENSG00000237102

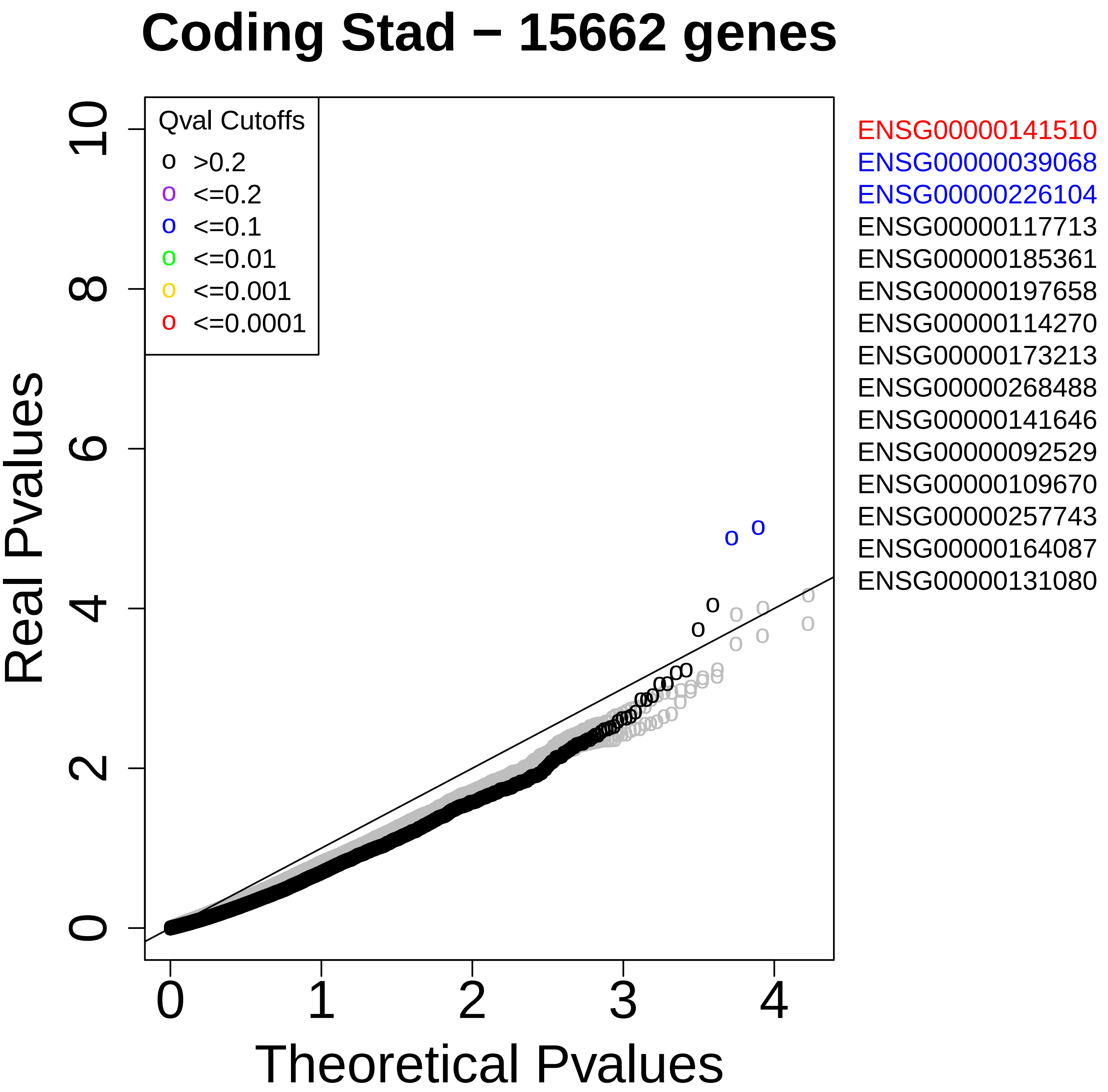

Coding Stad – 15662 genes
Qval Cutoffs
o >0.2
o <=0.2
o <=0.1
o <=0.01
o <=0.001
o <=0.0001
Real Pvalues
Theoretical Pvalues
0
2
4
6
8
10
0
1
2
3
4
ENSG00000141510
ENSG00000039068
ENSG00000226104
ENSG00000117713
ENSG00000185361
ENSG00000197658
ENSG00000114270
ENSG00000173213
ENSG00000268488
ENSG00000141646
ENSG00000092529
ENSG00000109670
ENSG00000257743
ENSG00000164087
ENSG00000131080

Coding Superpancancer − 19594 genes

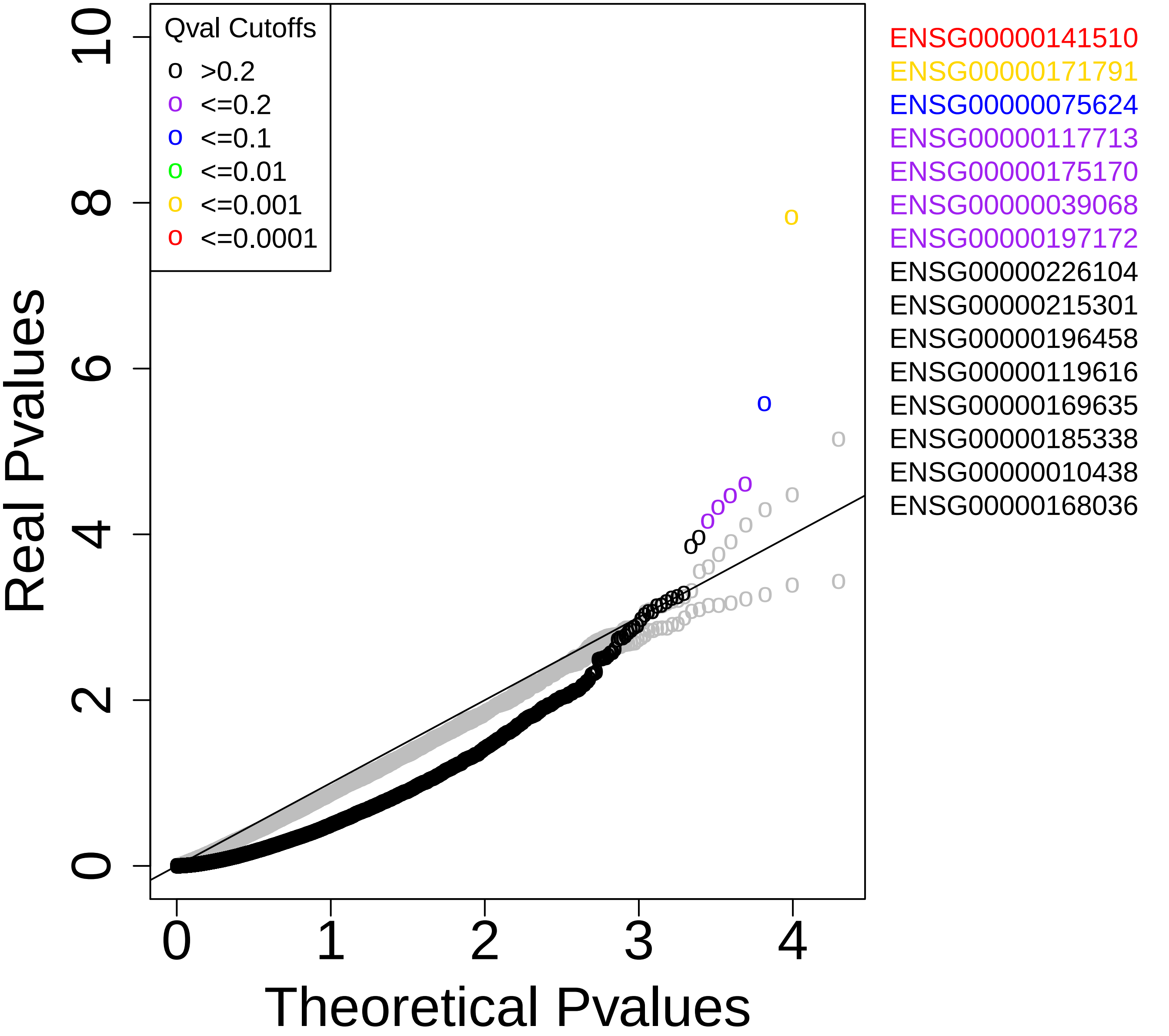

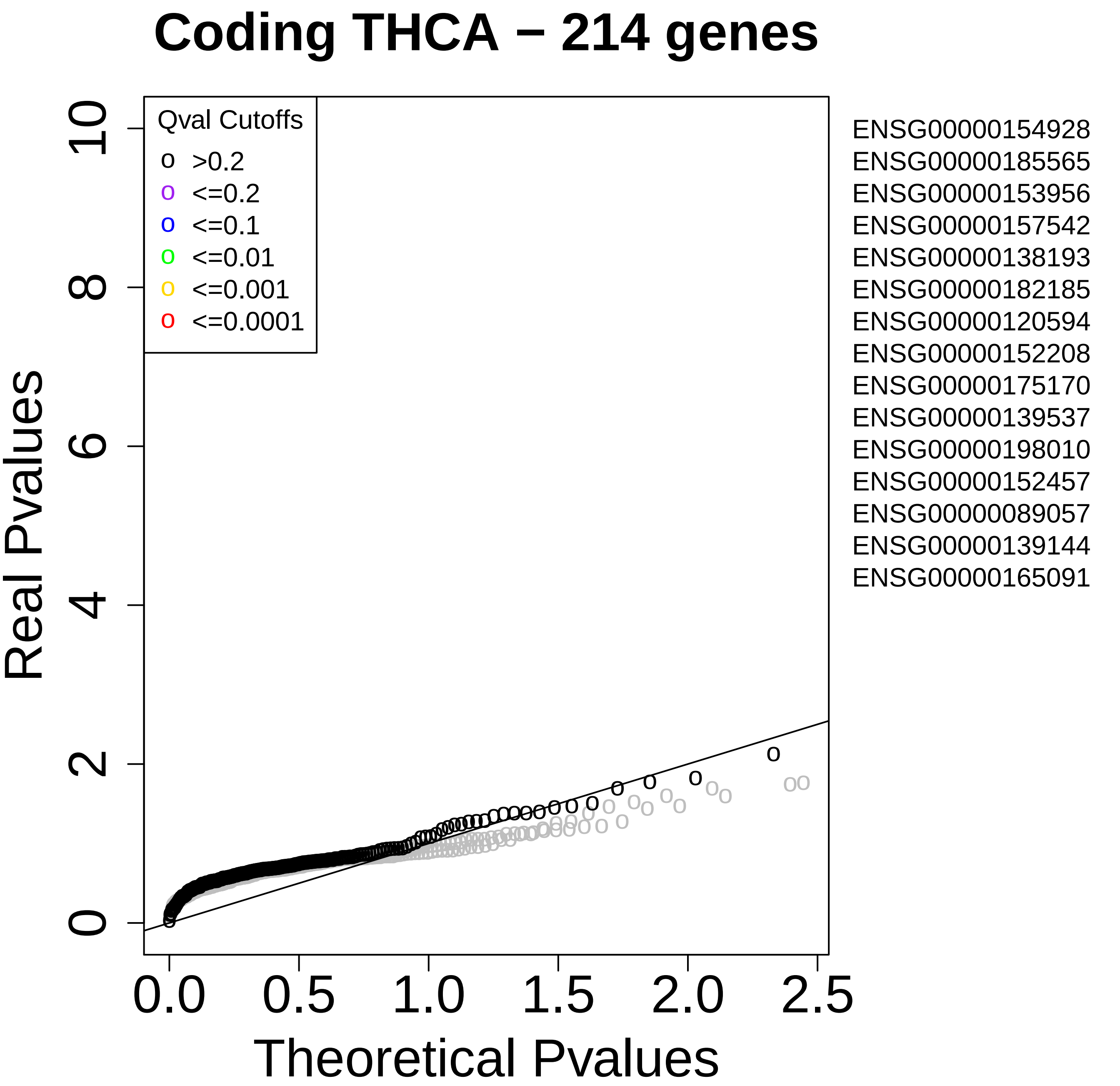
Coding THCA − 214 genes
Qval Cutoffs
o >0.2
o <=0.2
o <=0.1
o <=0.01
o <=0.001
o <=0.0001
Real Pvalues
0
2
4
6
8
10
Theoretical Pvalues
0.0
0.5
1.0
1.5
2.0
2.5
ENSG00000154928
ENSG00000185565
ENSG00000153956
ENSG00000157542
ENSG00000138193
ENSG00000182185
ENSG00000120594
ENSG00000152208
ENSG00000175170
ENSG00000139537
ENSG00000198010
ENSG00000152457
ENSG00000089057
ENSG00000139144
ENSG00000165091

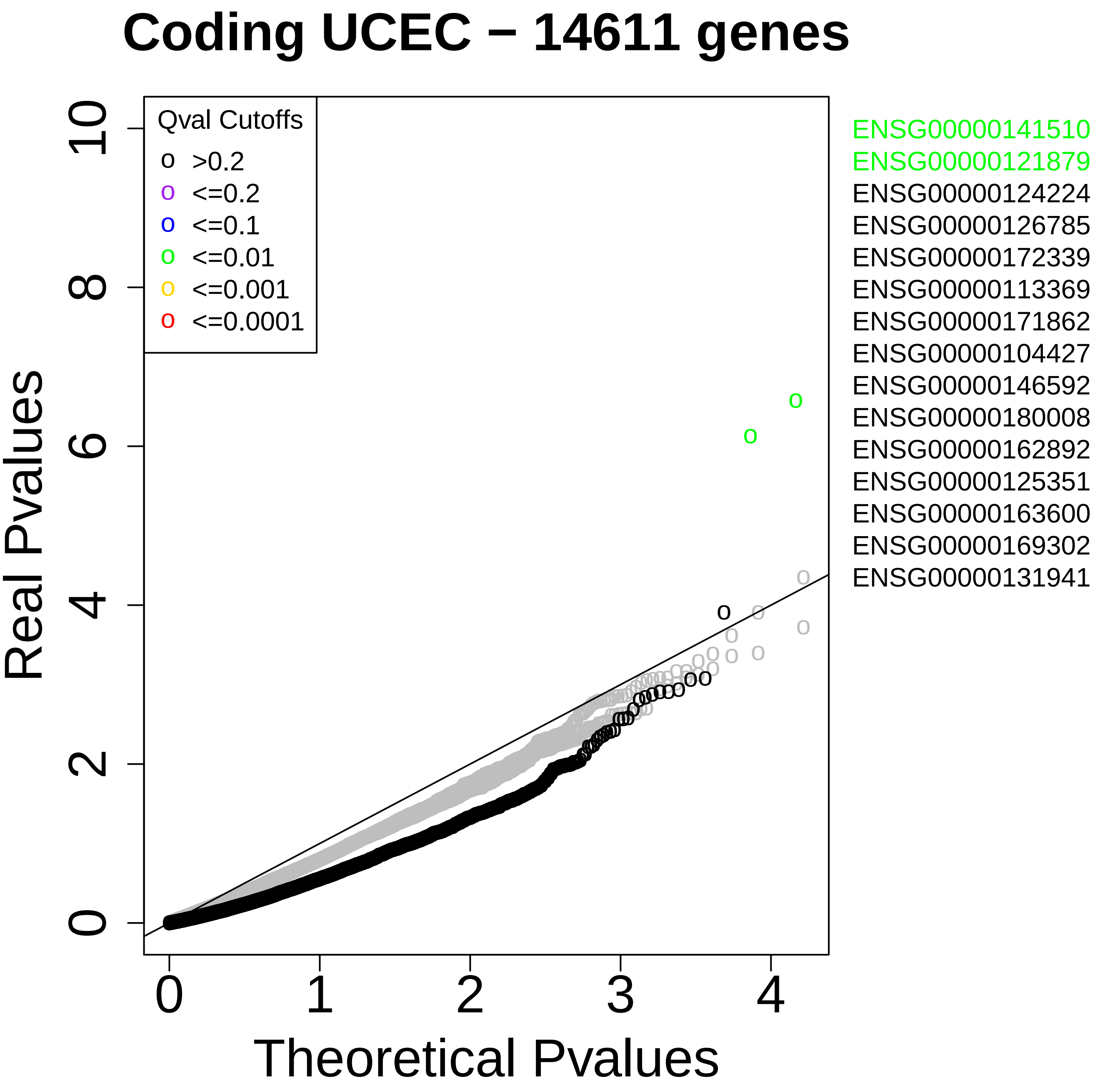
Coding UCEC − 14611 genes
Qval Cutoffs
o >0.2
o <=0.2
o <=0.1
o <=0.01
o <=0.001
o <=0.0001
Real Pvalues
Theoretical Pvalues
0
2
4
6
8
10
0
1
2
3
4
ENSG00000141510
ENSG00000121879
ENSG00000124224
ENSG00000126785
ENSG00000172339
ENSG00000113369
ENSG00000171862
ENSG00000104427
ENSG00000146592
ENSG00000180008
ENSG00000162892
ENSG00000125351
ENSG00000163600
ENSG00000169302
ENSG00000131941

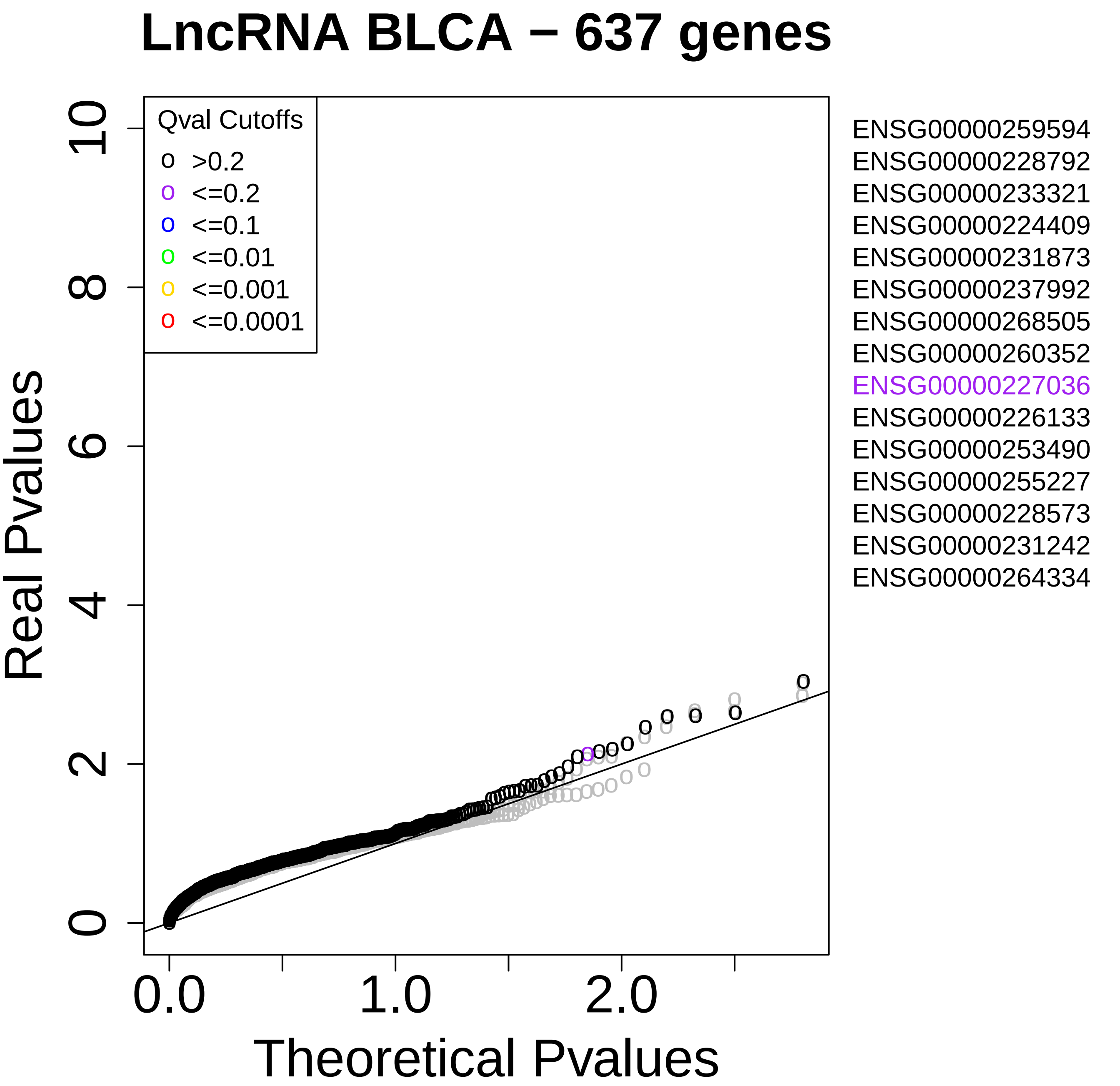
LncRNA BLCA − 637 genes
Qval Cutoffs
o >0.2
o <=0.2
o <=0.1
o <=0.01
o <=0.001
o <=0.0001
ENSG00000259594
ENSG00000228792
ENSG00000233321
ENSG00000224409
ENSG00000231873
ENSG00000237992
ENSG00000268505
ENSG00000260352
ENSG00000227036
ENSG00000226133
ENSG00000253490
ENSG00000255227
ENSG00000228573
ENSG00000231242
ENSG00000264334
Real Pvalues
Theoretical Pvalues
0
2
4
6
8
10
0.0
1.0
2.0

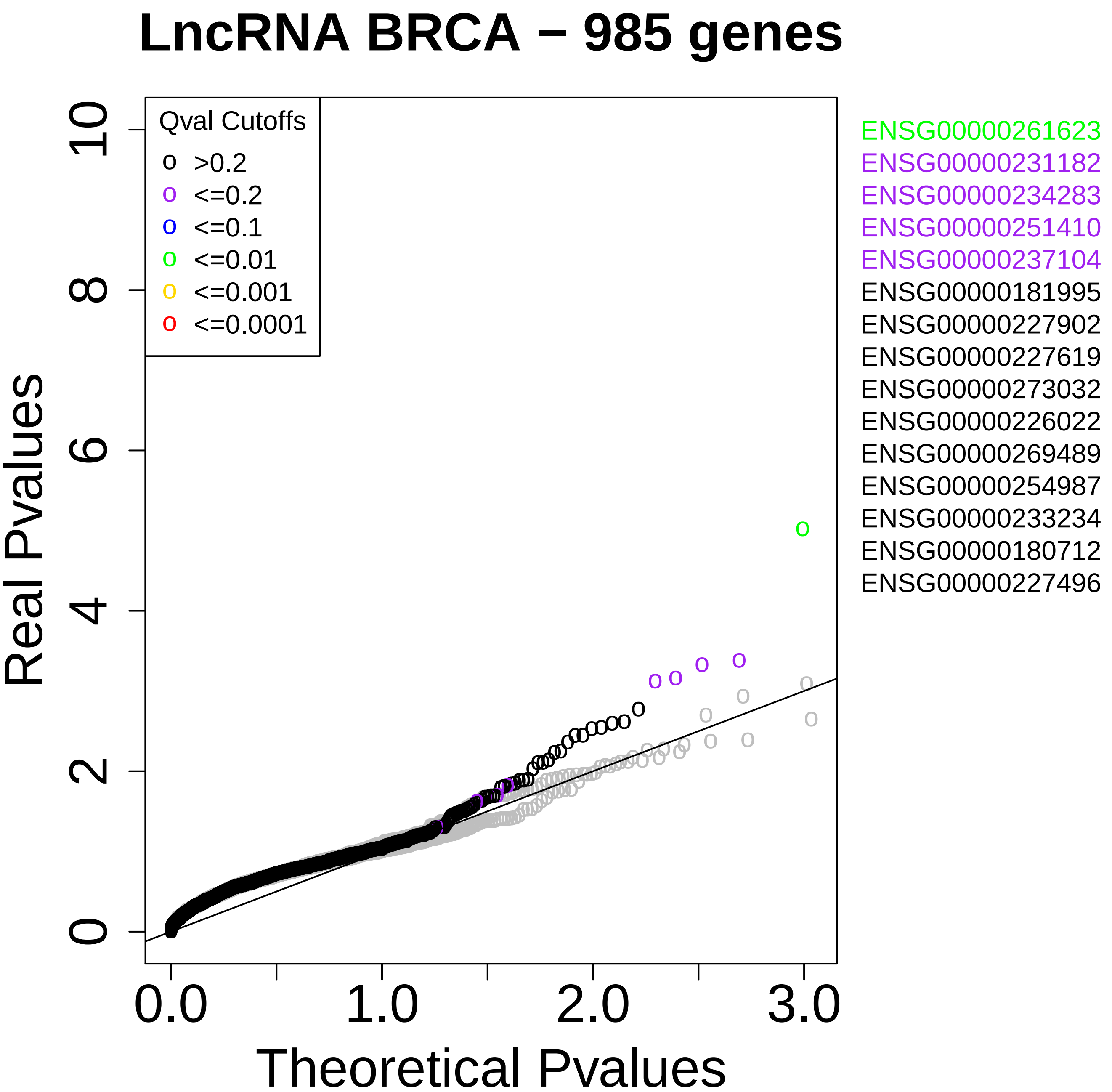

LncRNA BRCA − 985 genes
Qval Cutoffs
o >0.2
o <=0.2
o <=0.1
o <=0.01
o <=0.001
o <=0.0001
Real Pvalues
Theoretical Pvalues
0
2
4
6
8
10
0.0
1.0
2.0
3.0
ENSG00000261623
ENSG00000231182
ENSG00000234283
ENSG00000251410
ENSG00000237104
ENSG00000181995
ENSG00000227902
ENSG00000227619
ENSG00000273032
ENSG00000226022
ENSG00000269489
ENSG00000254987
ENSG00000233234
ENSG00000180712
ENSG00000227496

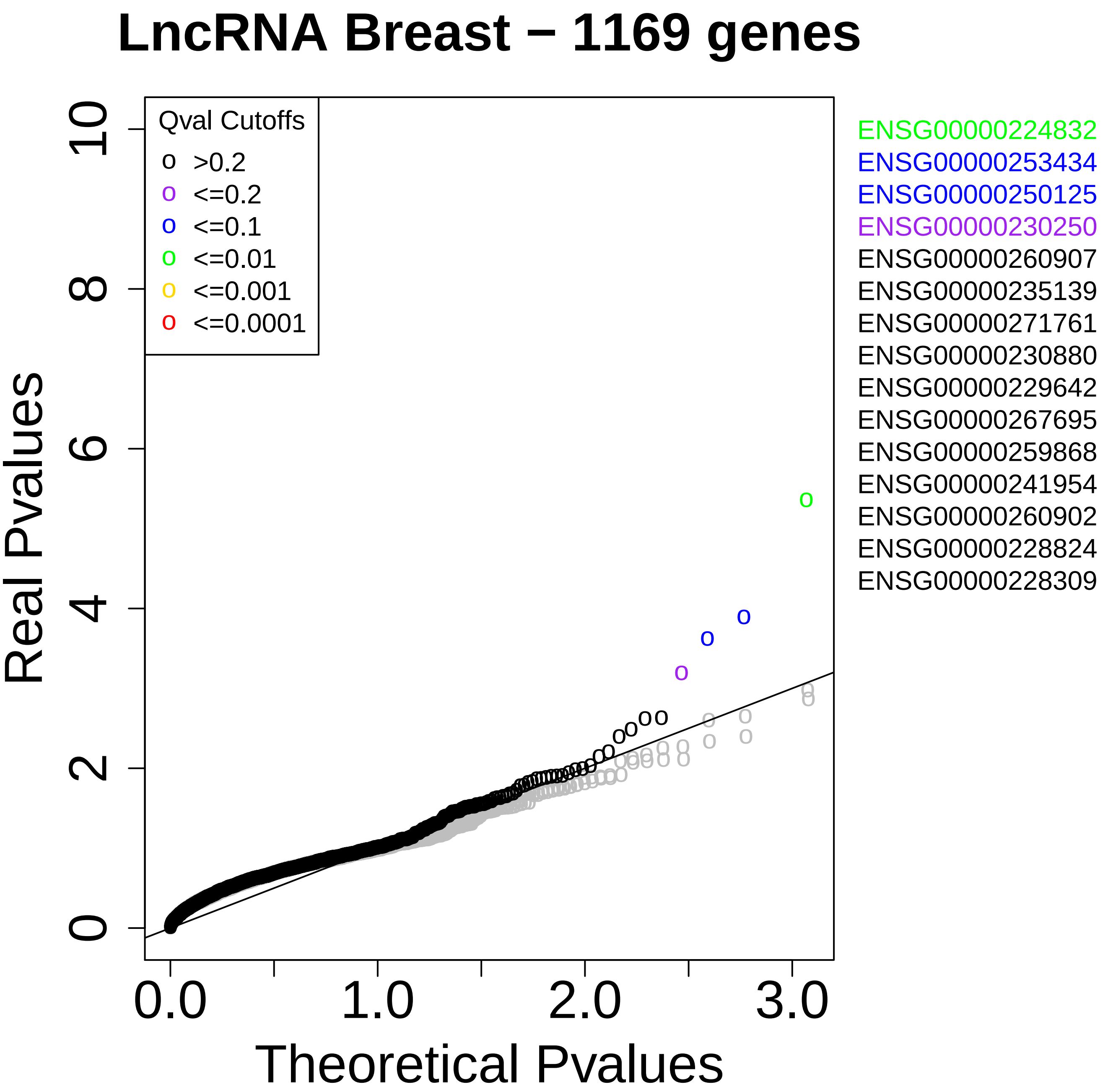
LncRNA Breast − 1169 genes
Qval Cutoffs
o >0.2
o <=0.2
o <=0.1
o <=0.01
o <=0.001
o <=0.0001
Real Pvalues
0
2
4
6
8
10
Theoretical Pvalues
0.0
1.0
2.0
3.0
ENSG00000224832
ENSG00000253434
ENSG00000250125
ENSG00000230250
ENSG00000260907
ENSG00000235139
ENSG00000271761
ENSG00000230880
ENSG00000229642
ENSG00000267695
ENSG00000259868
ENSG00000241954
ENSG00000260902
ENSG00000228824
ENSG00000228309

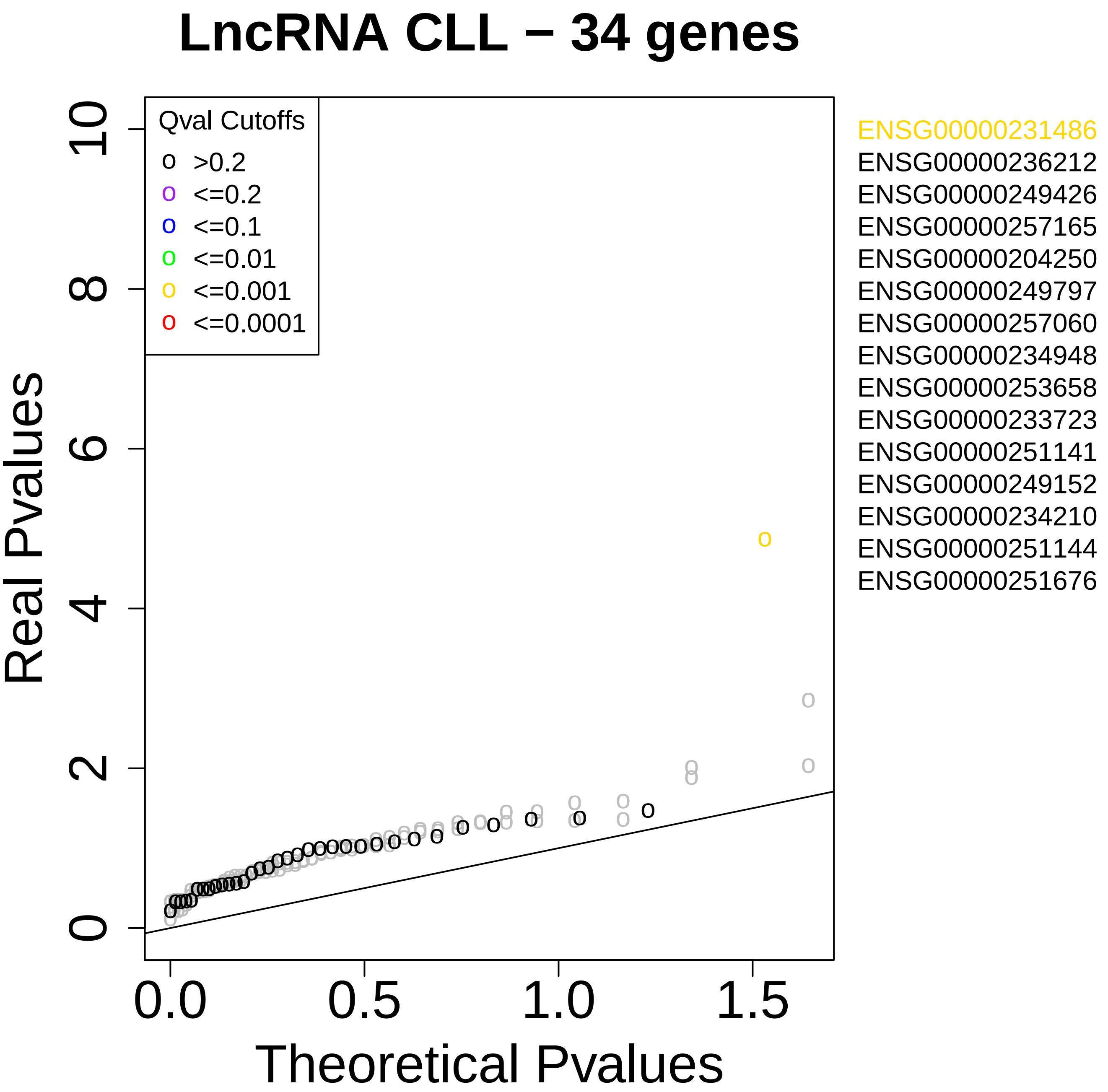
LncRNA CLL − 34 genes
Qval Cutoffs
o >0.2
o <=0.2
o <=0.1
o <=0.01
o <=0.001
o <=0.0001
Real Pvalues
Theoretical Pvalues
0
2
4
6
8
10
0.0
0.5
1.0
1.5
ENSG00000231486
ENSG00000236212
ENSG00000249426
ENSG00000257165
ENSG00000204250
ENSG00000249797
ENSG00000257060
ENSG00000234948
ENSG00000253658
ENSG00000233723
ENSG00000251141
ENSG00000249152
ENSG00000234210
ENSG00000251144
ENSG00000251676

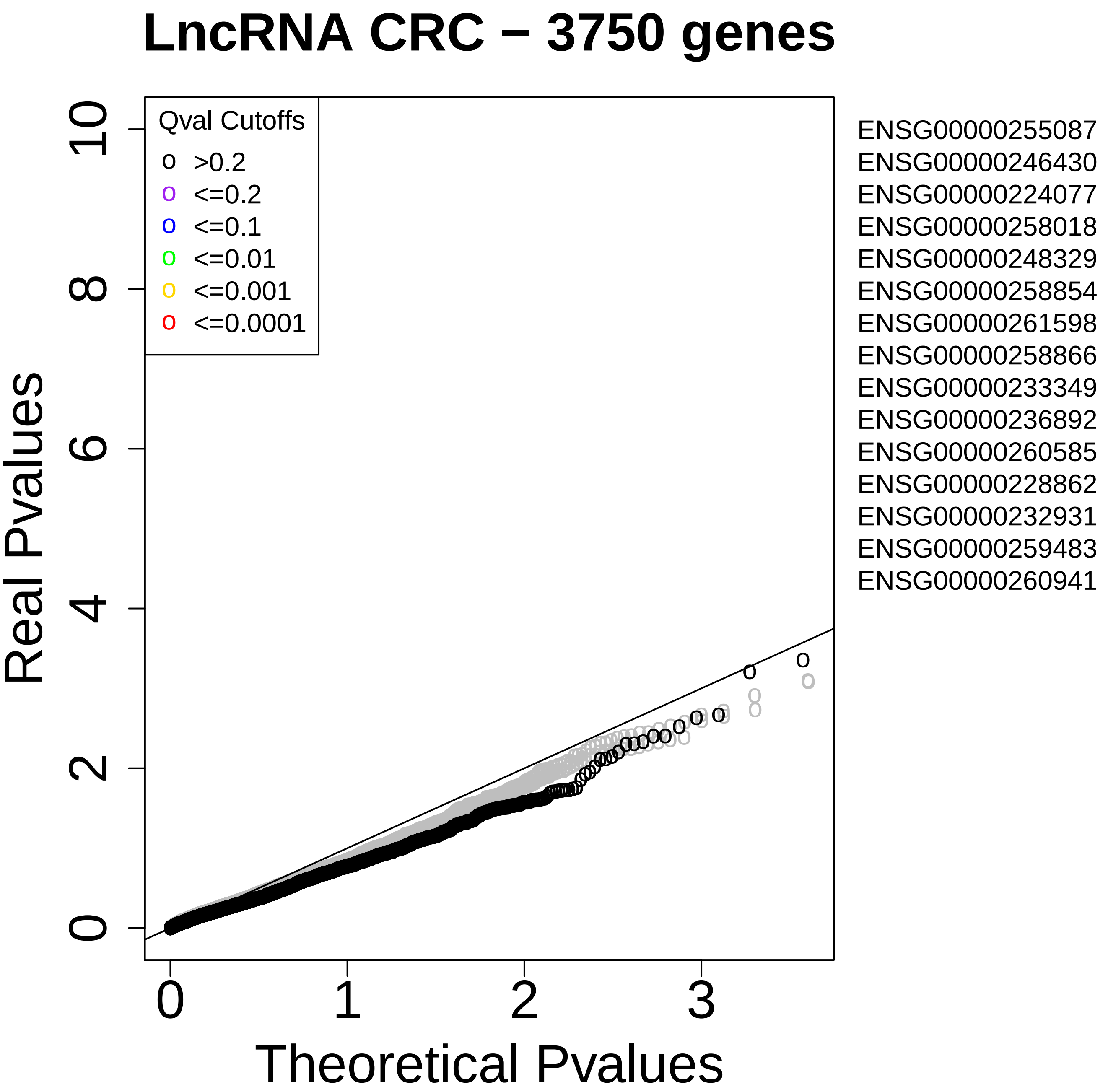
LncRNA CRC – 3750 genes
Qval Cutoffs
o >0.2
o <=0.2
o <=0.1
o <=0.01
o <=0.001
o <=0.0001
Real Pvalues
Theoretical Pvalues
0
2
4
6
8
10
0
1
2
3
ENSG00000255087
ENSG00000246430
ENSG00000224077
ENSG00000258018
ENSG00000248329
ENSG00000258854
ENSG00000261598
ENSG00000258866
ENSG00000233349
ENSG00000236892
ENSG00000260585
ENSG00000228862
ENSG00000232931
ENSG00000259483
ENSG00000260941

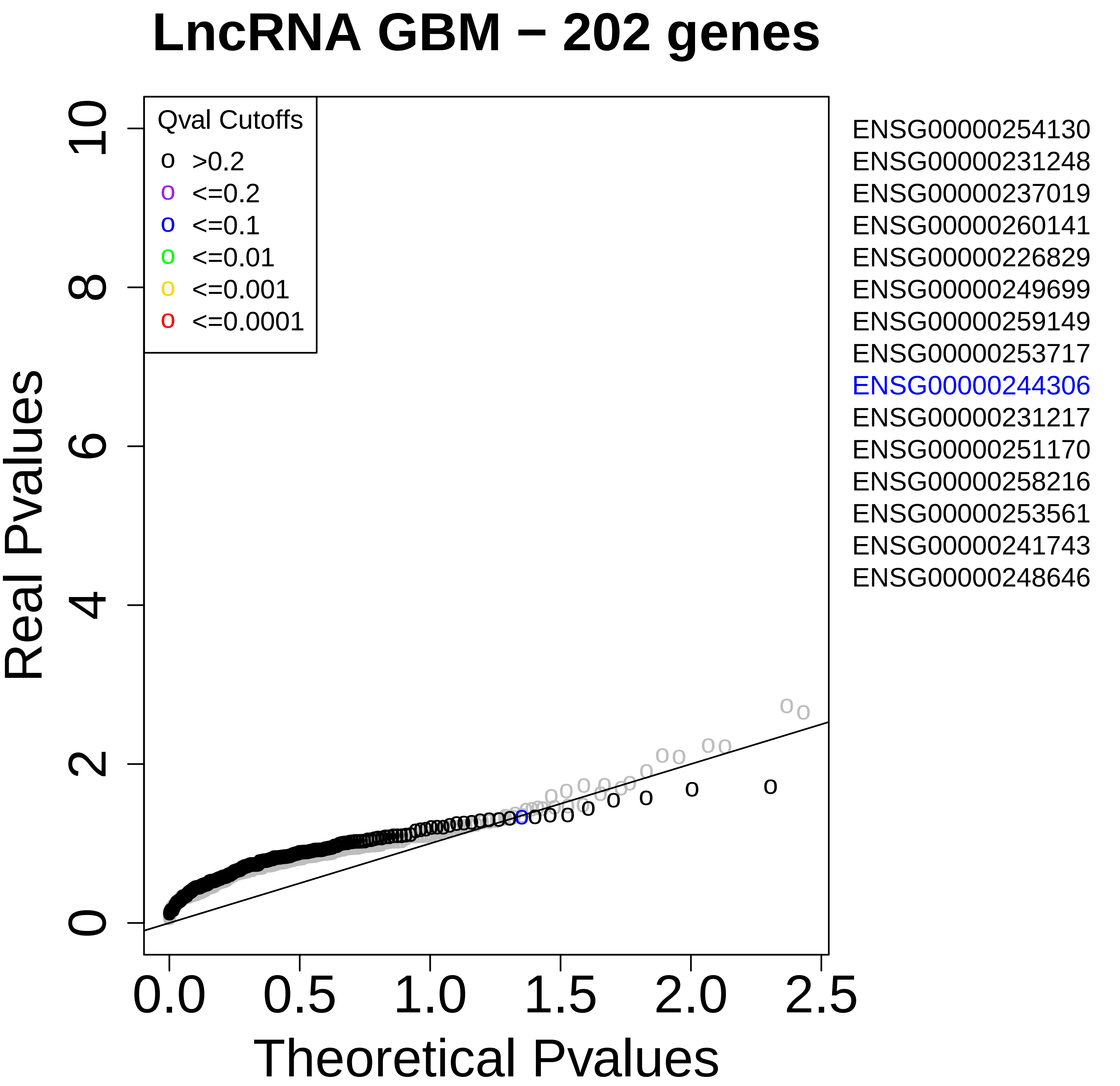
LncRNA GBM − 202 genes
Qval Cutoffs
o >0.2
o <=0.2
o <=0.1
o <=0.01
o <=0.001
o <=0.0001
Real Pvalues
Theoretical Pvalues
0
2
4
6
8
10
0.0
0.5
1.0
1.5
2.0
2.5
ENSG00000254130
ENSG00000231248
ENSG00000237019
ENSG00000260141
ENSG00000226829
ENSG00000249699
ENSG00000259149
ENSG00000253717
ENSG00000244306
ENSG00000231217
ENSG00000251170
ENSG00000258216
ENSG00000253561
ENSG00000241743
ENSG00000248646

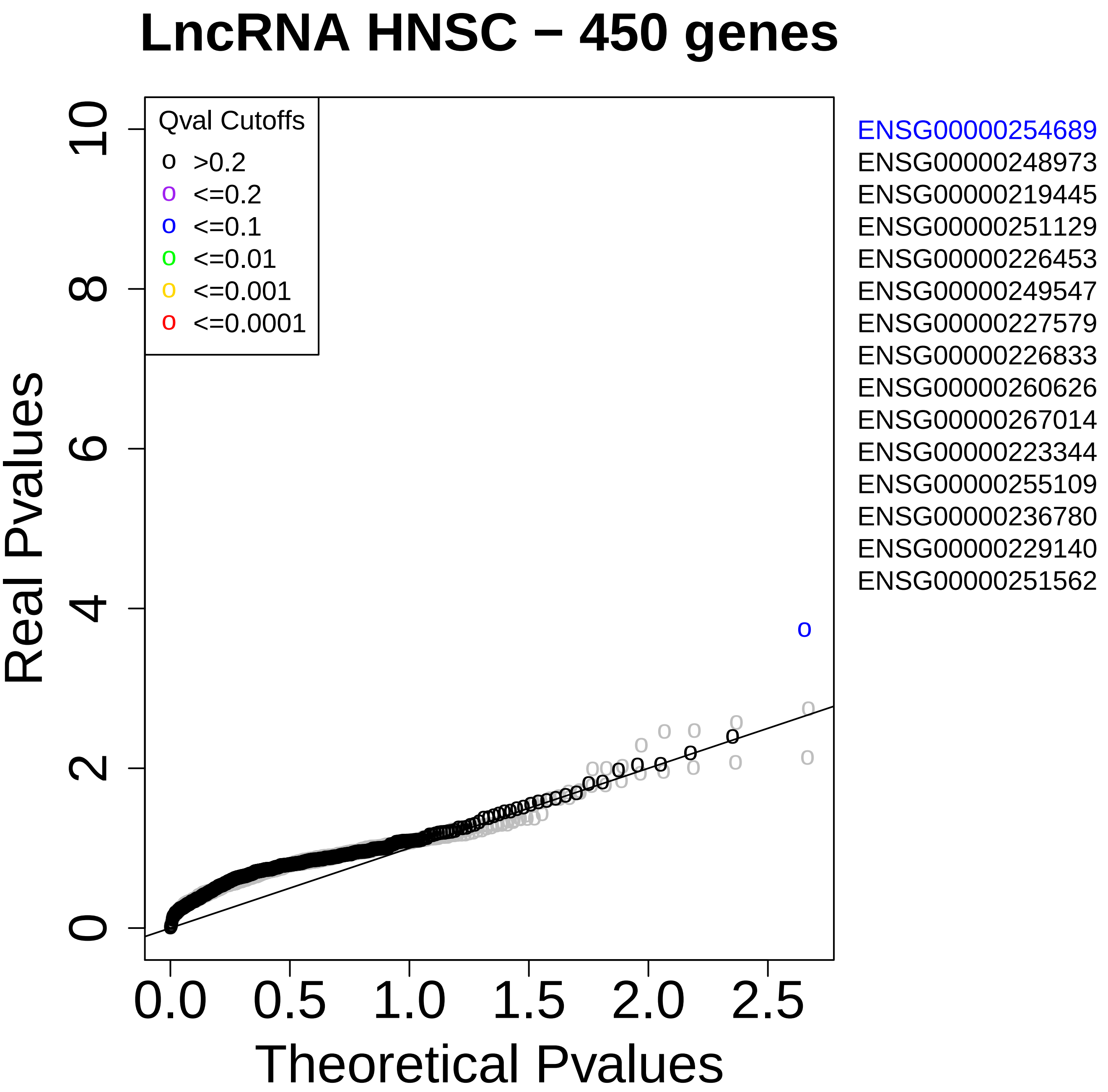

LncRNA HNSC − 450 genes
Qval Cutoffs
o >0.2
o <=0.2
o <=0.1
o <=0.01
o <=0.001
o <=0.0001
Real Pvalues
0
2
4
6
8
10
Theoretical Pvalues
0.0
0.5
1.0
1.5
2.0
2.5
ENSG00000254689
ENSG00000248973
ENSG00000219445
ENSG00000251129
ENSG00000226453
ENSG00000249547
ENSG00000227579
ENSG00000226833
ENSG00000260626
ENSG00000267014
ENSG00000223344
ENSG00000255109
ENSG00000236780
ENSG00000229140
ENSG00000251562

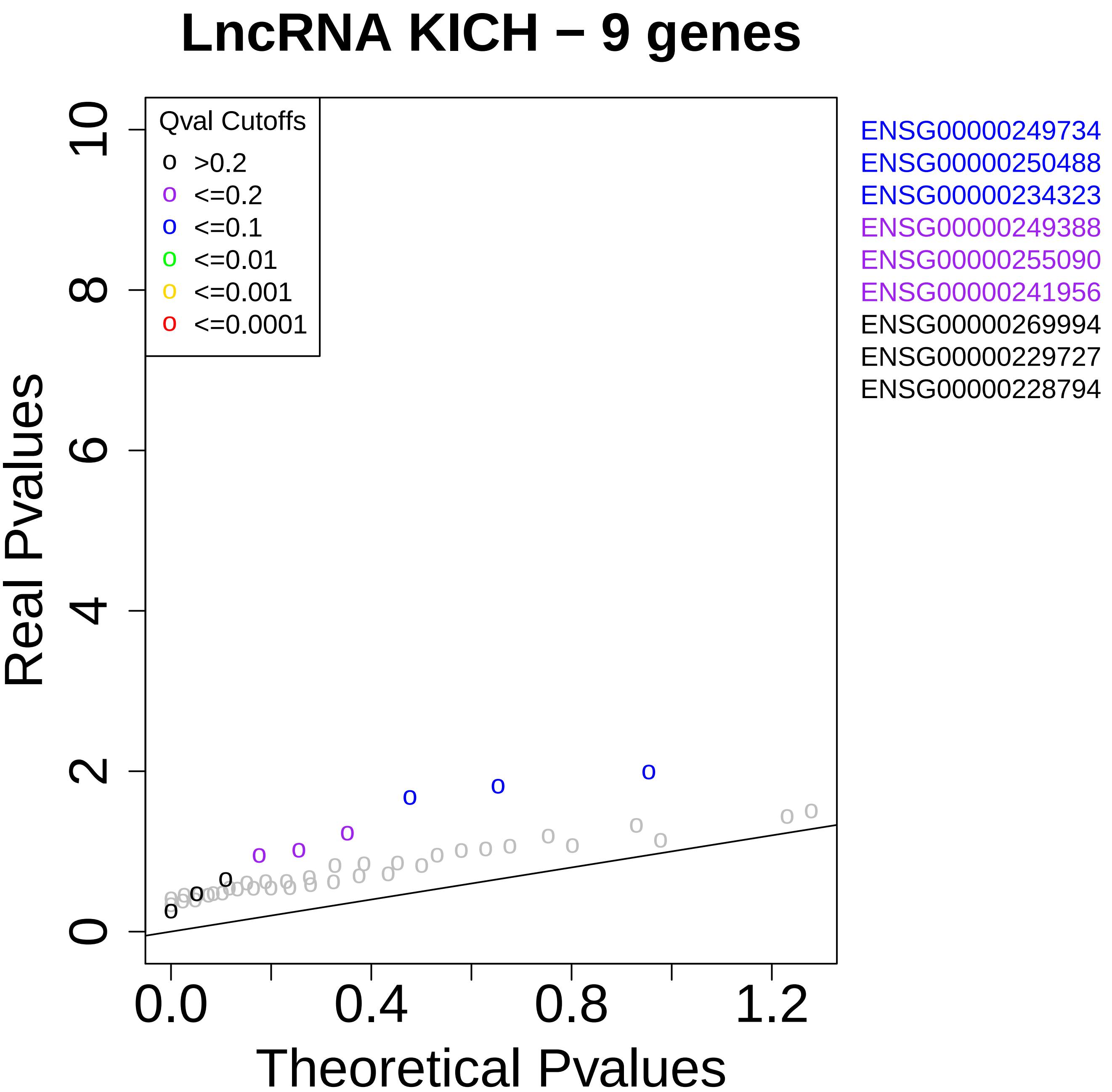
LncRNA KICH – 9 genes
Qval Cutoffs
o >0.2
o <=0.2
o <=0.1
o <=0.01
o <=0.001
o <=0.0001
Real Pvalues
Theoretical Pvalues
0
2
4
6
8
10
0.0
0.4
0.8
1.2
ENSG00000249734
ENSG00000250488
ENSG00000234323
ENSG00000249388
ENSG00000255090
ENSG00000241956
ENSG00000269994
ENSG00000229727
ENSG00000228794

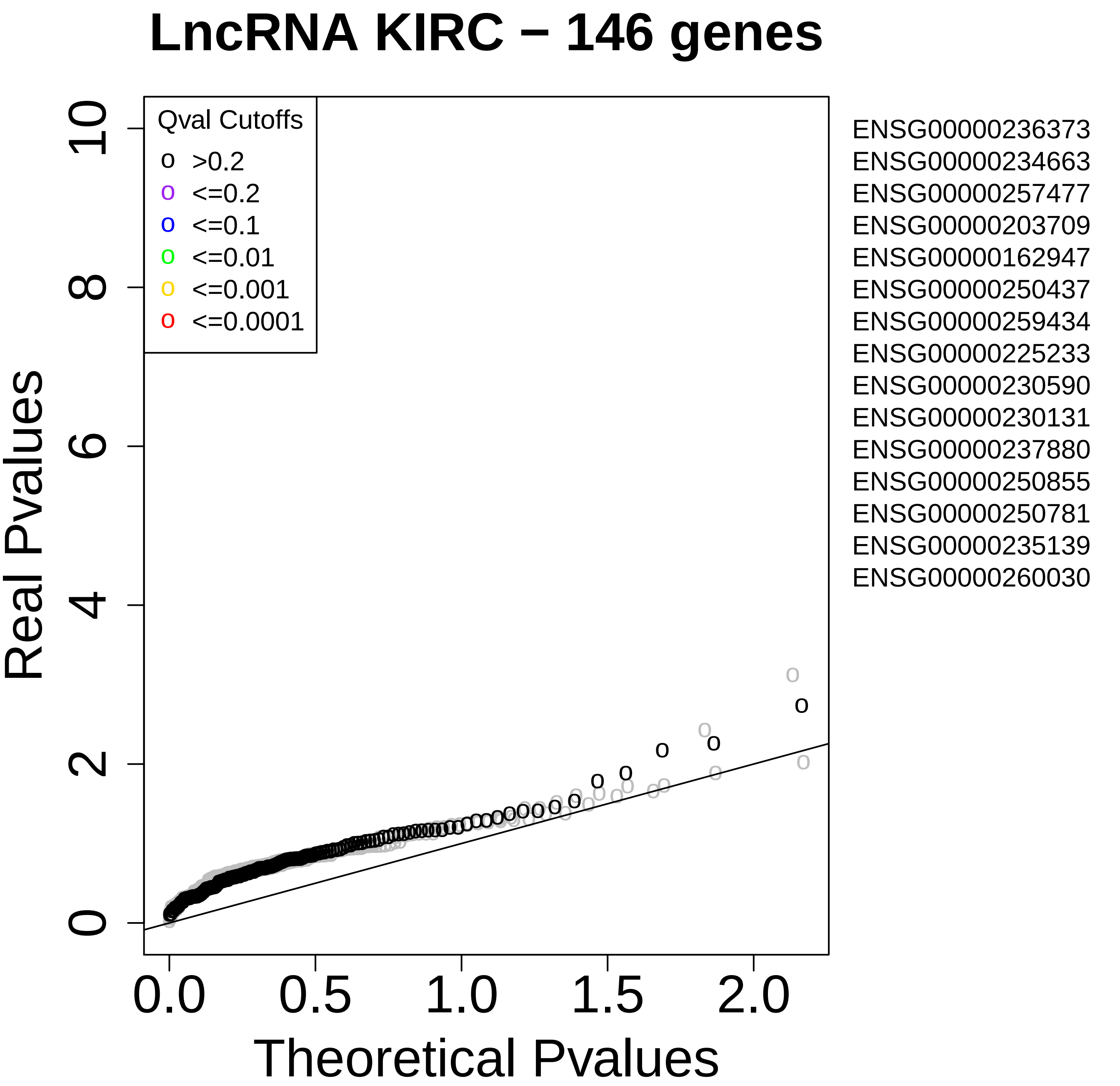

LncRNA KIRC − 146 genes
Qval Cutoffs
o >0.2
o <=0.2
o <=0.1
o <=0.01
o <=0.001
o <=0.0001
Real Pvalues
Theoretical Pvalues
0
2
4
6
8
10
0.0
0.5
1.0
1.5
2.0
ENSG00000236373
ENSG00000234663
ENSG00000257477
ENSG00000203709
ENSG00000162947
ENSG00000250437
ENSG00000259434
ENSG00000225233
ENSG00000230590
ENSG00000230131
ENSG00000237880
ENSG00000250855
ENSG00000250781
ENSG00000235139
ENSG00000260030

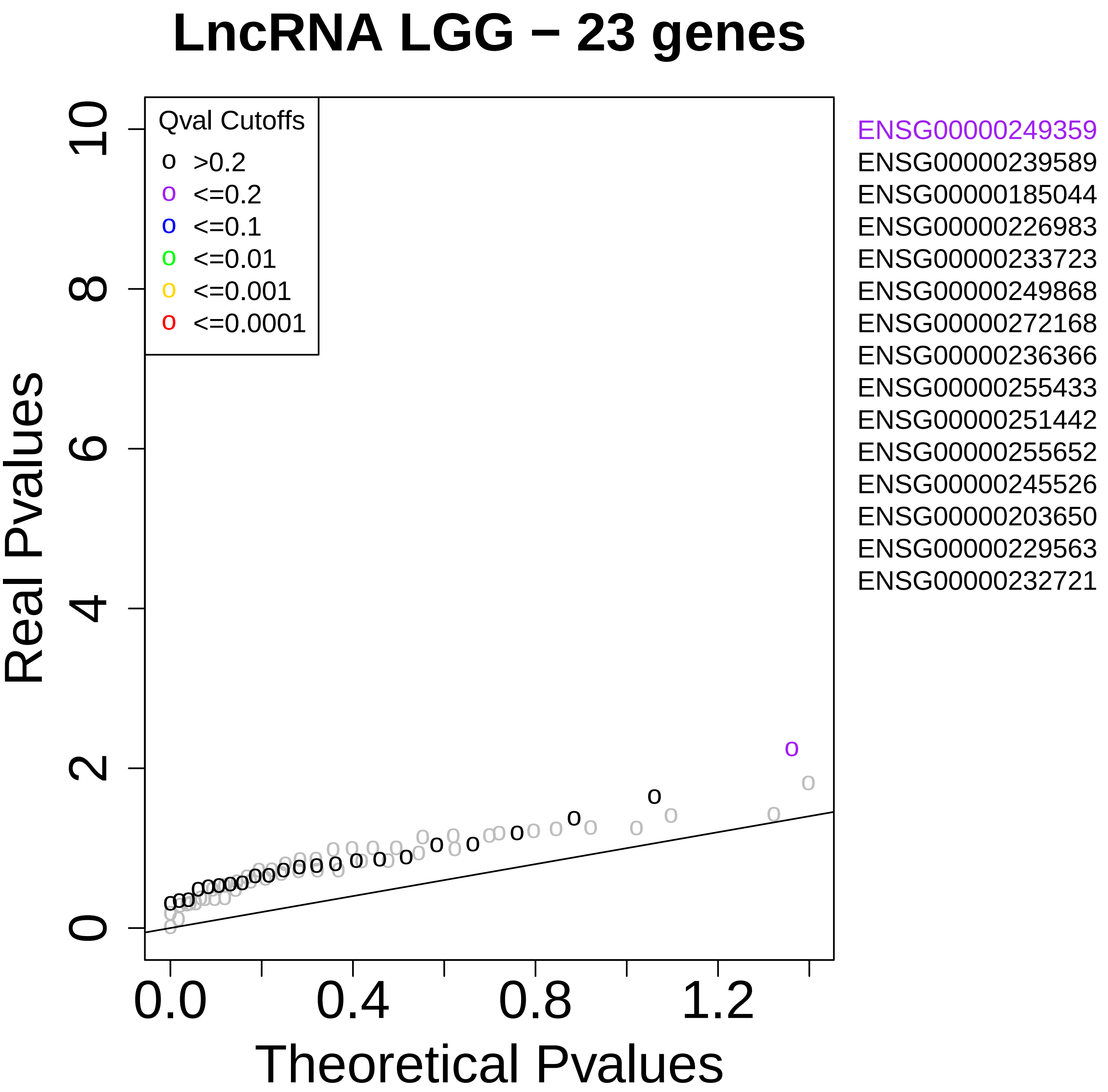
LncRNA LGG − 23 genes
Qval Cutoffs
o >0.2
o <=0.2
o <=0.1
o <=0.01
o <=0.001
o <=0.0001
Real Pvalues
Theoretical Pvalues
0
2
4
6
8
10
0.0
0.4
0.8
1.2
ENSG00000249359
ENSG00000239589
ENSG00000185044
ENSG00000226983
ENSG00000233723
ENSG00000249868
ENSG00000272168
ENSG00000236366
ENSG00000255433
ENSG00000251442
ENSG00000255652
ENSG00000245526
ENSG00000203650
ENSG00000229563
ENSG00000232721

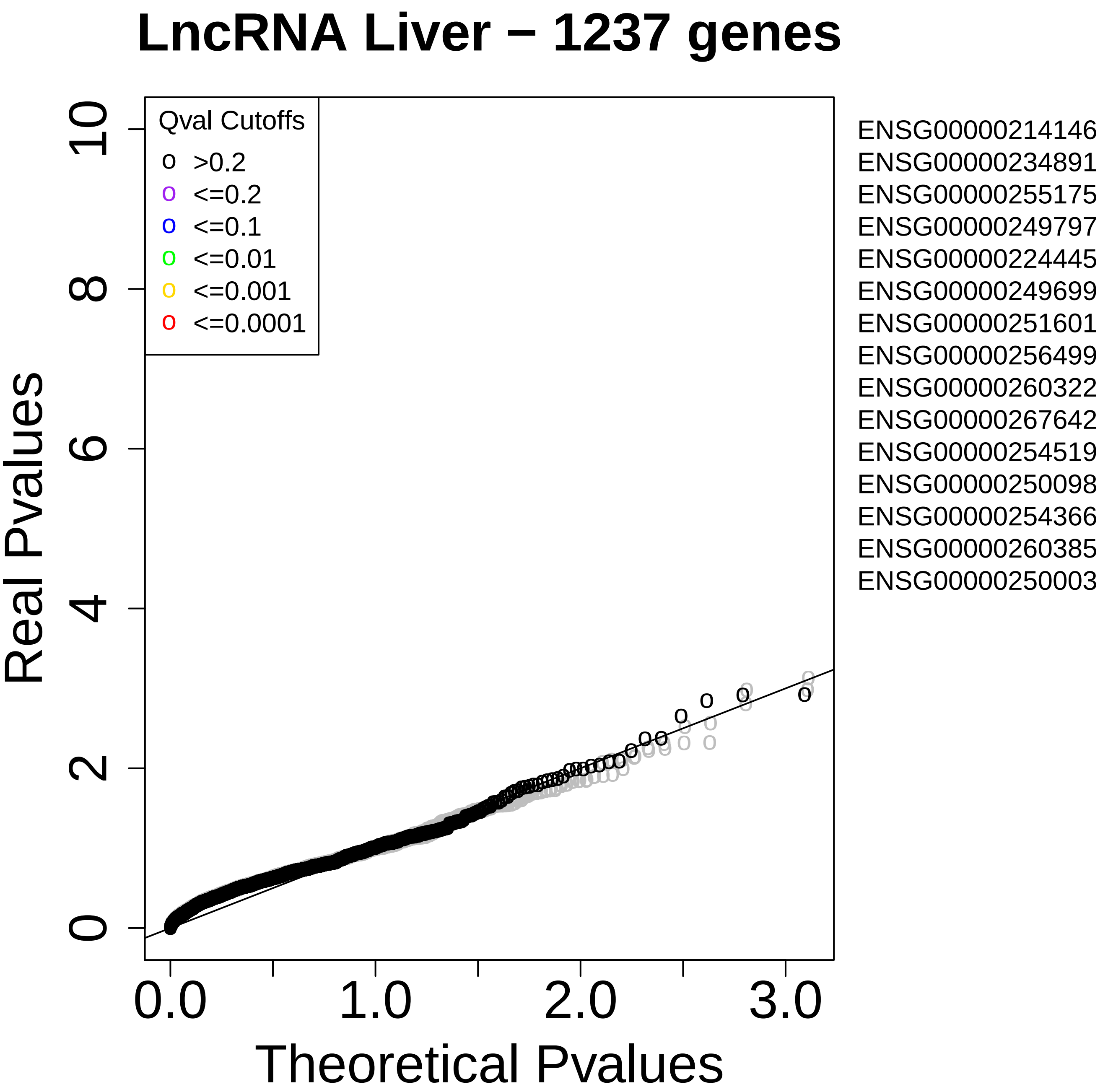
LncRNA Liver − 1237 genes
Qval Cutoffs
o >0.2
o <=0.2
o <=0.1
o <=0.01
o <=0.001
o <=0.0001
ENSG00000214146
ENSG00000234891
ENSG00000255175
ENSG00000249797
ENSG00000224445
ENSG00000249699
ENSG00000251601
ENSG00000256499
ENSG00000260322
ENSG00000267642
ENSG00000254519
ENSG00000250098
ENSG00000254366
ENSG00000260385
ENSG00000250003
Real Pvalues
Theoretical Pvalues
0
2
4
6
8
10
0.0
1.0
2.0
3.0

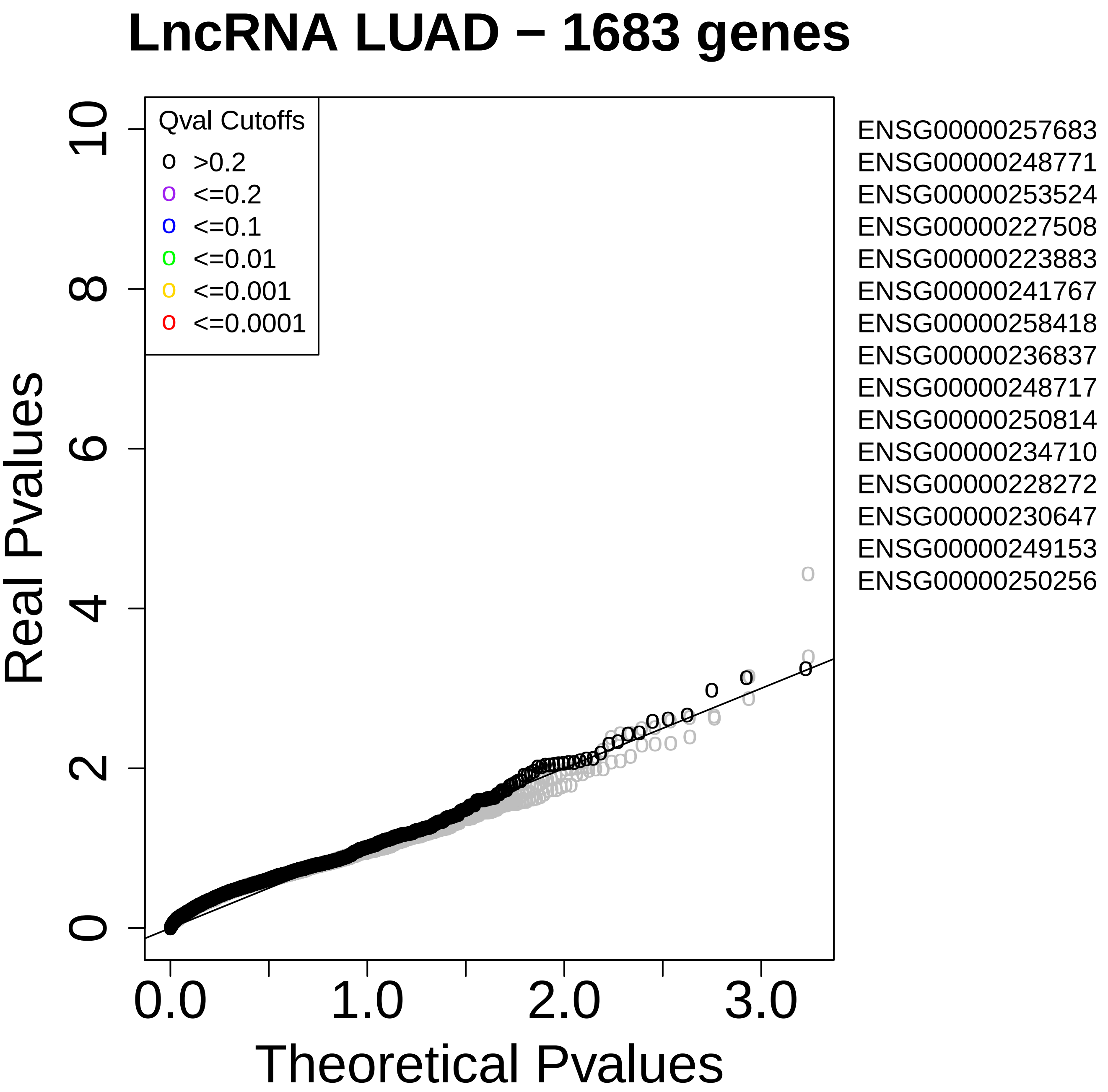

LncRNA LUAD − 1683 genes
Qval Cutoffs
o >0.2
o <=0.2
o <=0.1
o <=0.01
o <=0.001
o <=0.0001
Real Pvalues
Theoretical Pvalues
0
2
4
6
8
10
0.0
1.0
2.0
3.0
ENSG00000257683
ENSG00000248771
ENSG00000253524
ENSG00000227508
ENSG00000223883
ENSG00000241767
ENSG00000258418
ENSG00000236837
ENSG00000248717
ENSG00000250814
ENSG00000234710
ENSG00000228272
ENSG00000230647
ENSG00000249153
ENSG00000250256

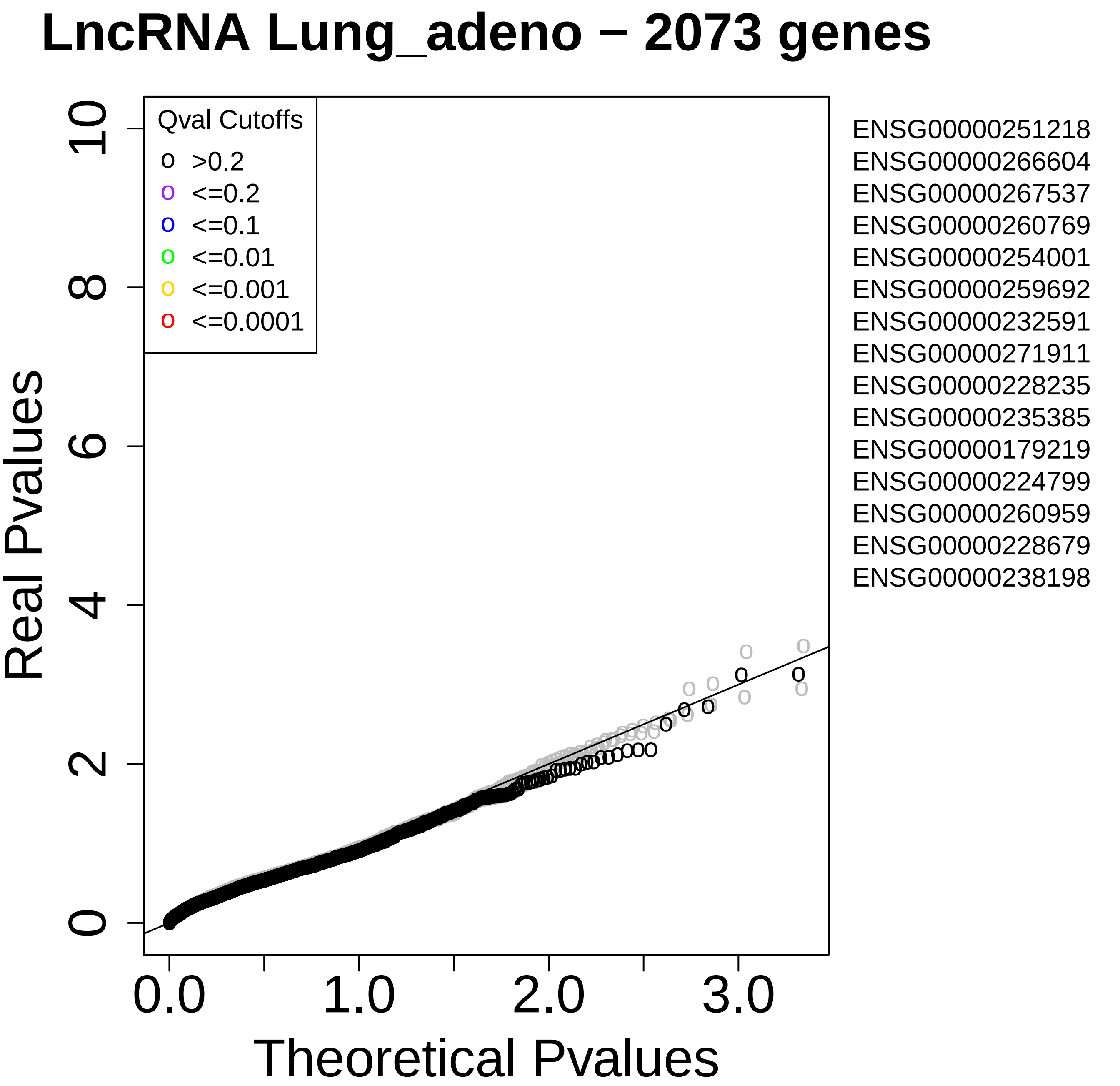
LncRNA Lung_adeno − 2073 genes
Qval Cutoffs
o >0.2
o <=0.2
o <=0.1
o <=0.01
o <=0.001
o <=0.0001
Real Pvalues
Theoretical Pvalues
0
2
4
6
8
10
0.0
1.0
2.0
3.0
ENSG00000251218
ENSG00000266604
ENSG00000267537
ENSG00000260769
ENSG00000254001
ENSG00000259692
ENSG00000232591
ENSG00000271911
ENSG00000228235
ENSG00000235385
ENSG00000179219
ENSG00000224799
ENSG00000260959
ENSG00000228679
ENSG00000238198

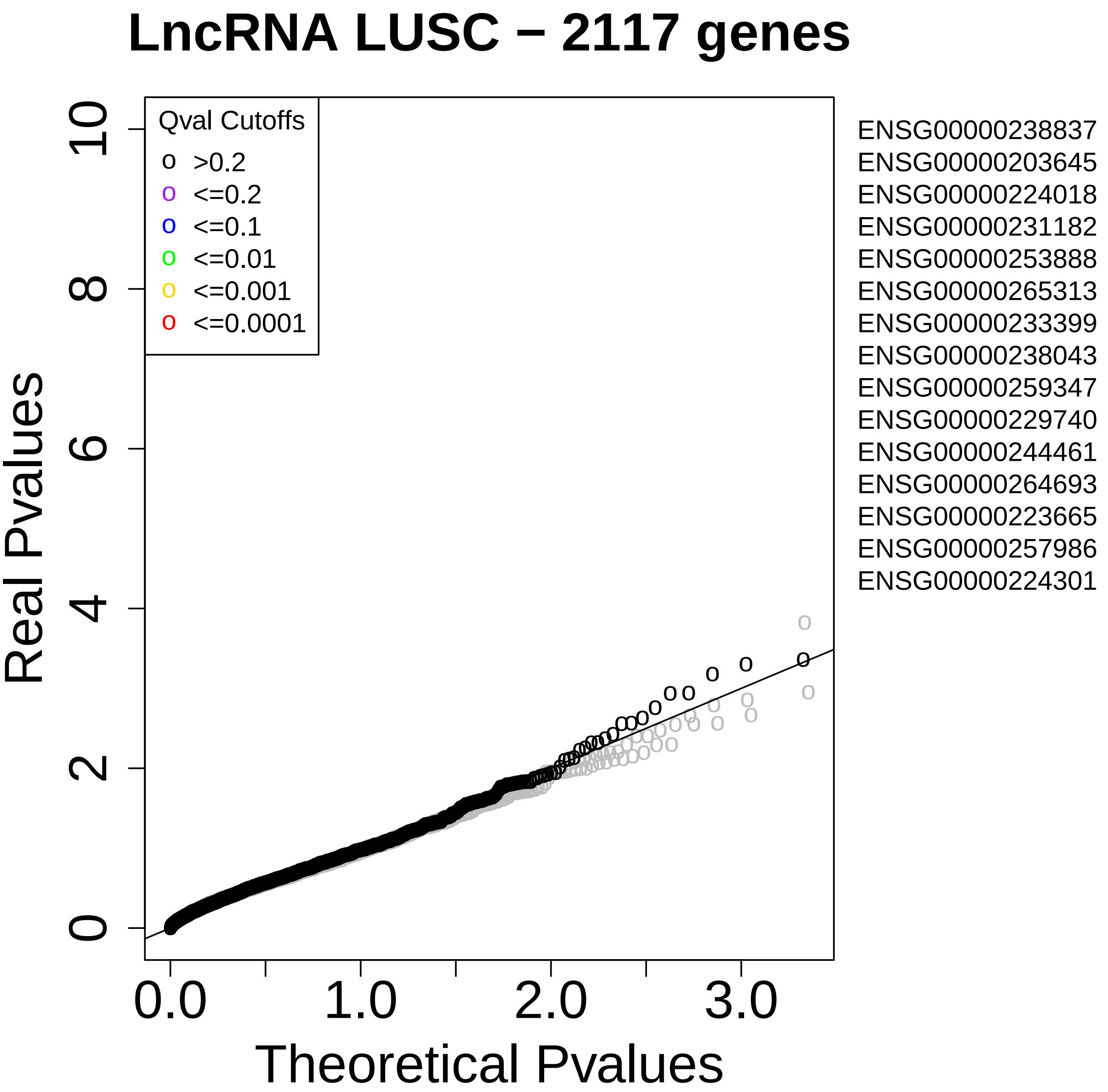
LncRNA LUSC – 2117 genes
Qval Cutoffs
o >0.2
o <=0.2
o <=0.1
o <=0.01
o <=0.001
o <=0.0001
Real Pvalues
Theoretical Pvalues
0
2
4
6
8
10
0.0
1.0
2.0
3.0
ENSG00000238837
ENSG00000203645
ENSG00000224018
ENSG00000231182
ENSG00000253888
ENSG00000265313
ENSG00000233399
ENSG00000238043
ENSG00000259347
ENSG00000229740
ENSG00000244461
ENSG00000264693
ENSG00000223665
ENSG00000257986
ENSG00000224301

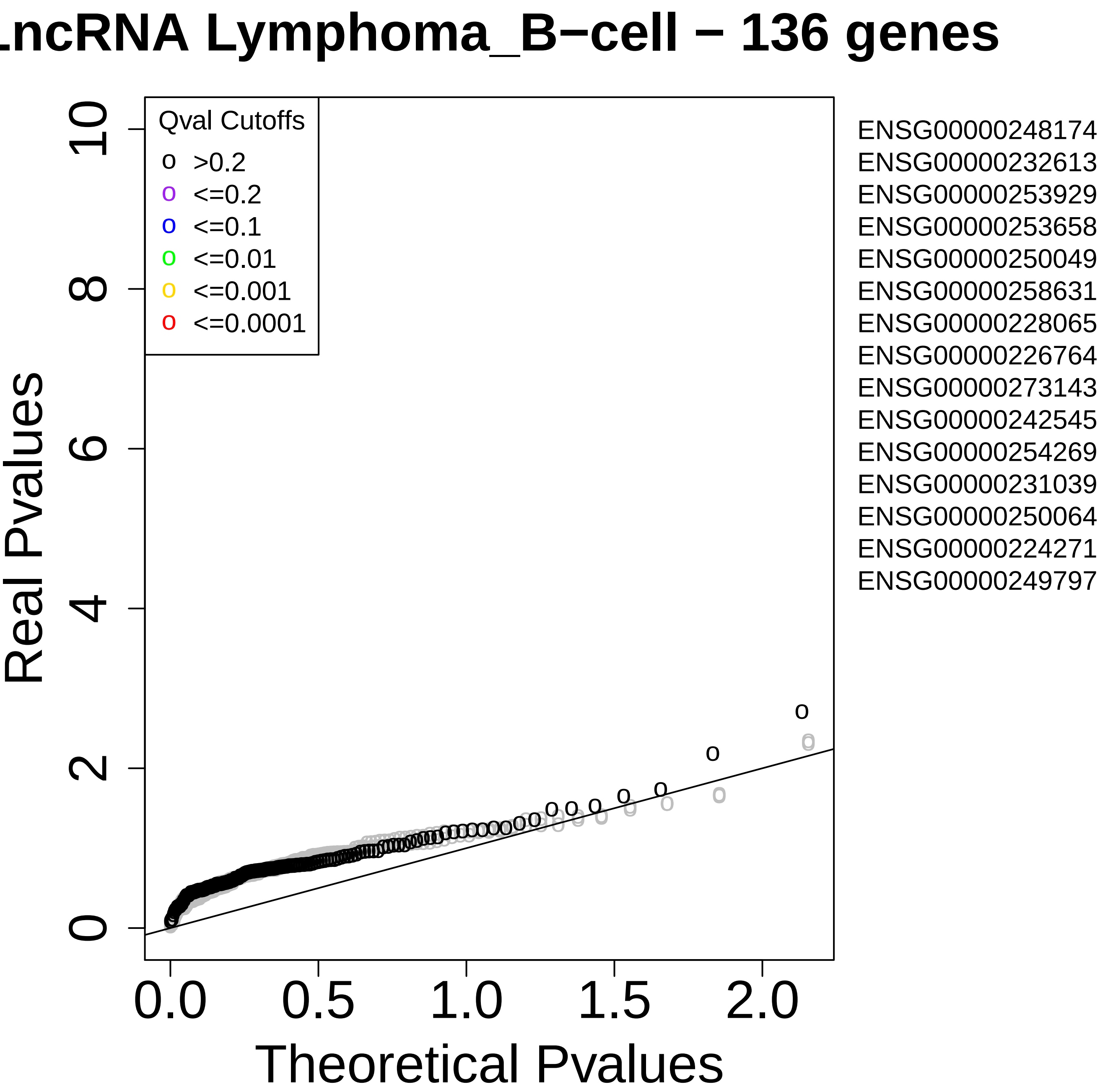
LncRNA Lymphoma_B−cell − 136 genes
Qval Cutoffs
o >0.2
o <=0.2
o <=0.1
o <=0.01
o <=0.001
o <=0.0001
Real Pvalues
Theoretical Pvalues
0
2
4
6
8
10
0.0
0.5
1.0
1.5
2.0
ENSG00000248174
ENSG00000232613
ENSG00000253929
ENSG00000253658
ENSG00000250049
ENSG00000258631
ENSG00000228065
ENSG00000226764
ENSG00000273143
ENSG00000242545
ENSG00000254269
ENSG00000231039
ENSG00000250064
ENSG00000224271
ENSG00000249797

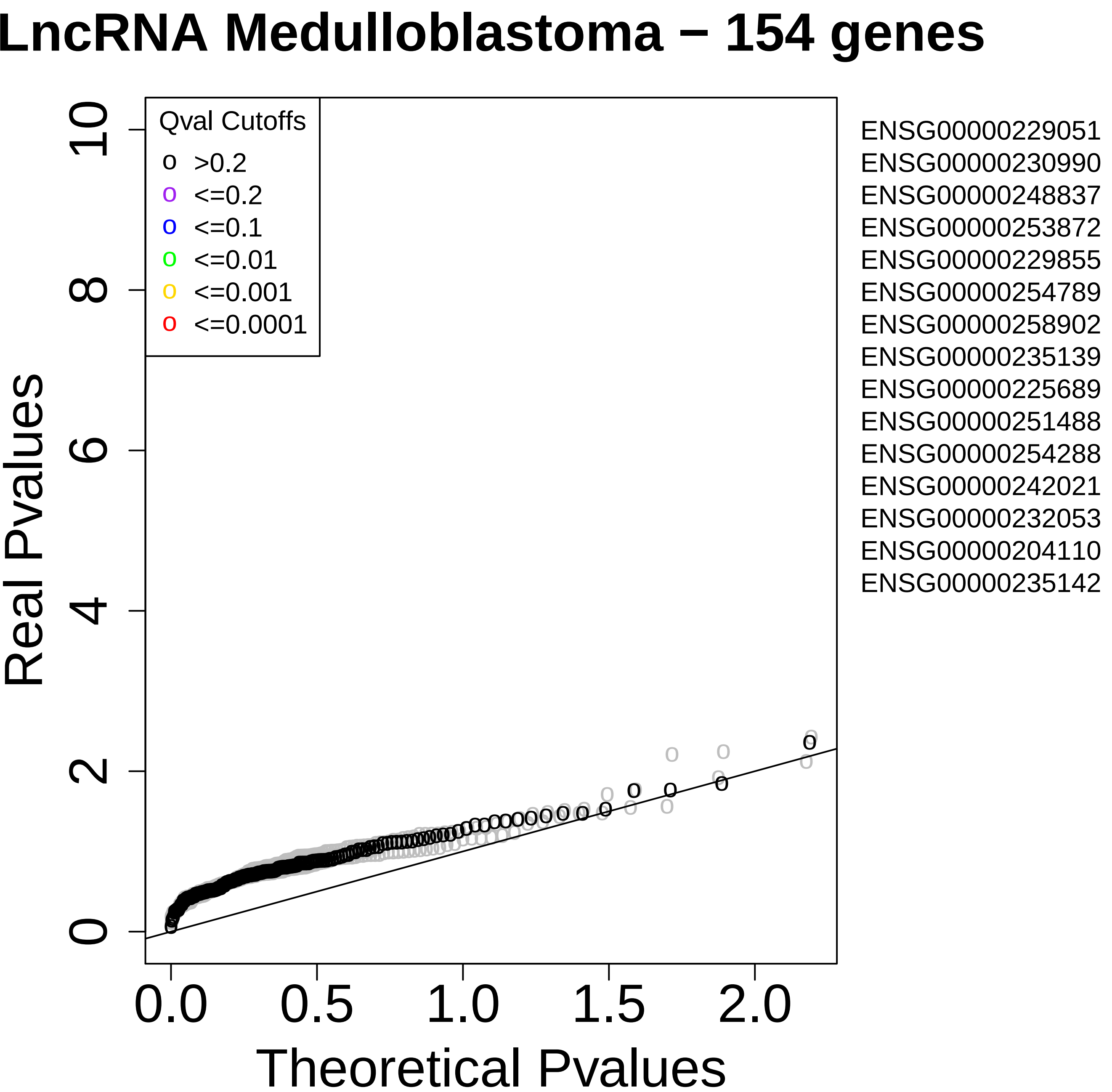
LncRNA Medulloblastoma − 154 genes
Qval Cutoffs
o >0.2
o <=0.2
o <=0.1
o <=0.01
o <=0.001
o <=0.0001
Real Pvalues
Theoretical Pvalues
0
2
4
6
8
10
0.0
0.5
1.0
1.5
2.0
ENSG00000229051
ENSG00000230990
ENSG00000248837
ENSG00000253872
ENSG00000229855
ENSG00000254789
ENSG00000258902
ENSG00000235139
ENSG00000225689
ENSG00000251488
ENSG00000254288
ENSG00000242021
ENSG00000232053
ENSG00000204110
ENSG00000235142

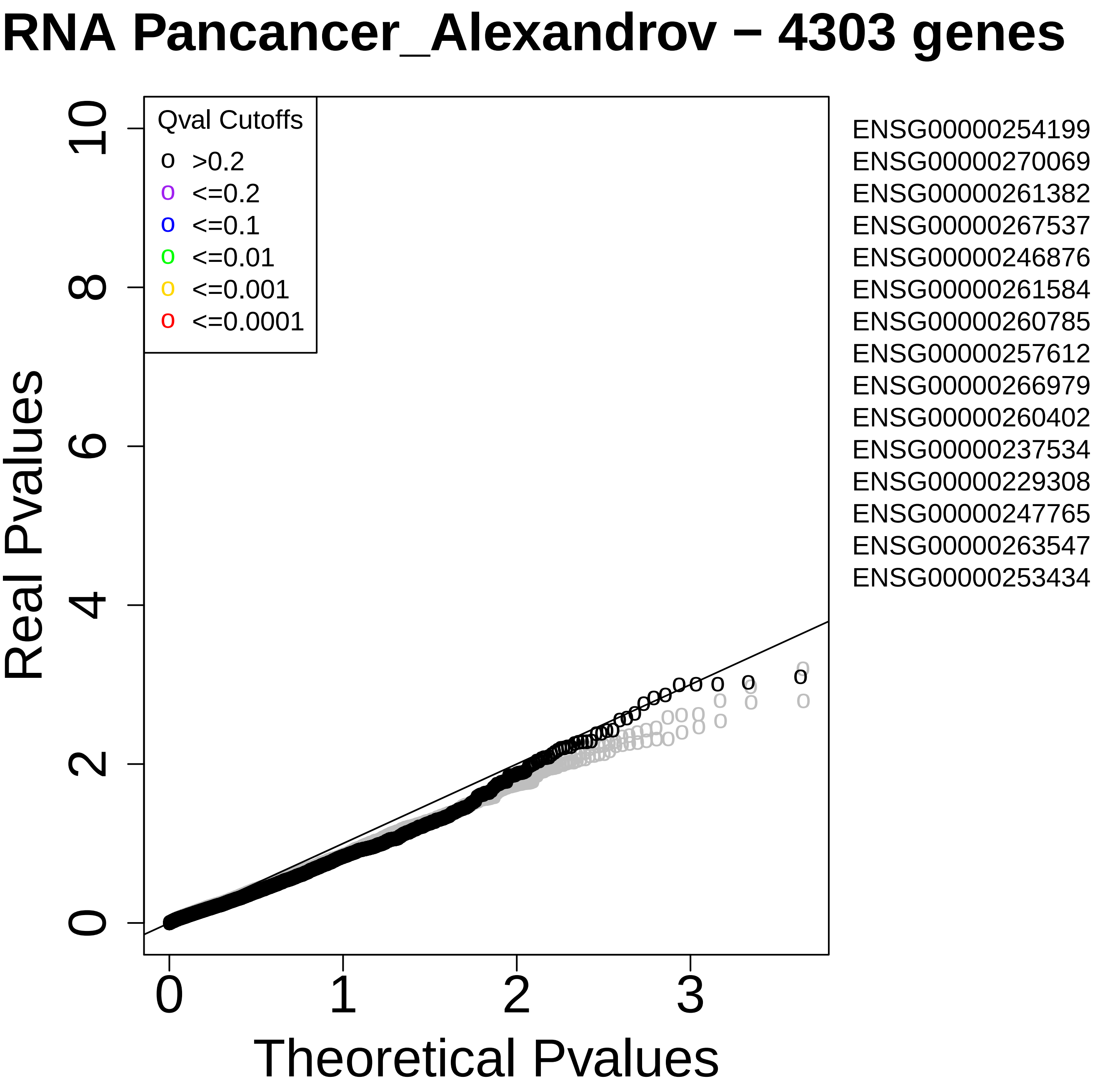

RNA Pancancer_Alexandrov − 4303 genes
Qval Cutoffs
o >0.2
o <=0.2
o <=0.1
o <=0.01
o <=0.001
o <=0.0001
Real Pvalues
Theoretical Pvalues
0
2
4
6
8
10
0
1
2
3
ENSG00000254199
ENSG00000270069
ENSG00000261382
ENSG00000267537
ENSG00000246876
ENSG00000261584
ENSG00000260785
ENSG00000257612
ENSG00000266979
ENSG00000260402
ENSG00000237534
ENSG00000229308
ENSG00000247765
ENSG00000263547
ENSG00000253434

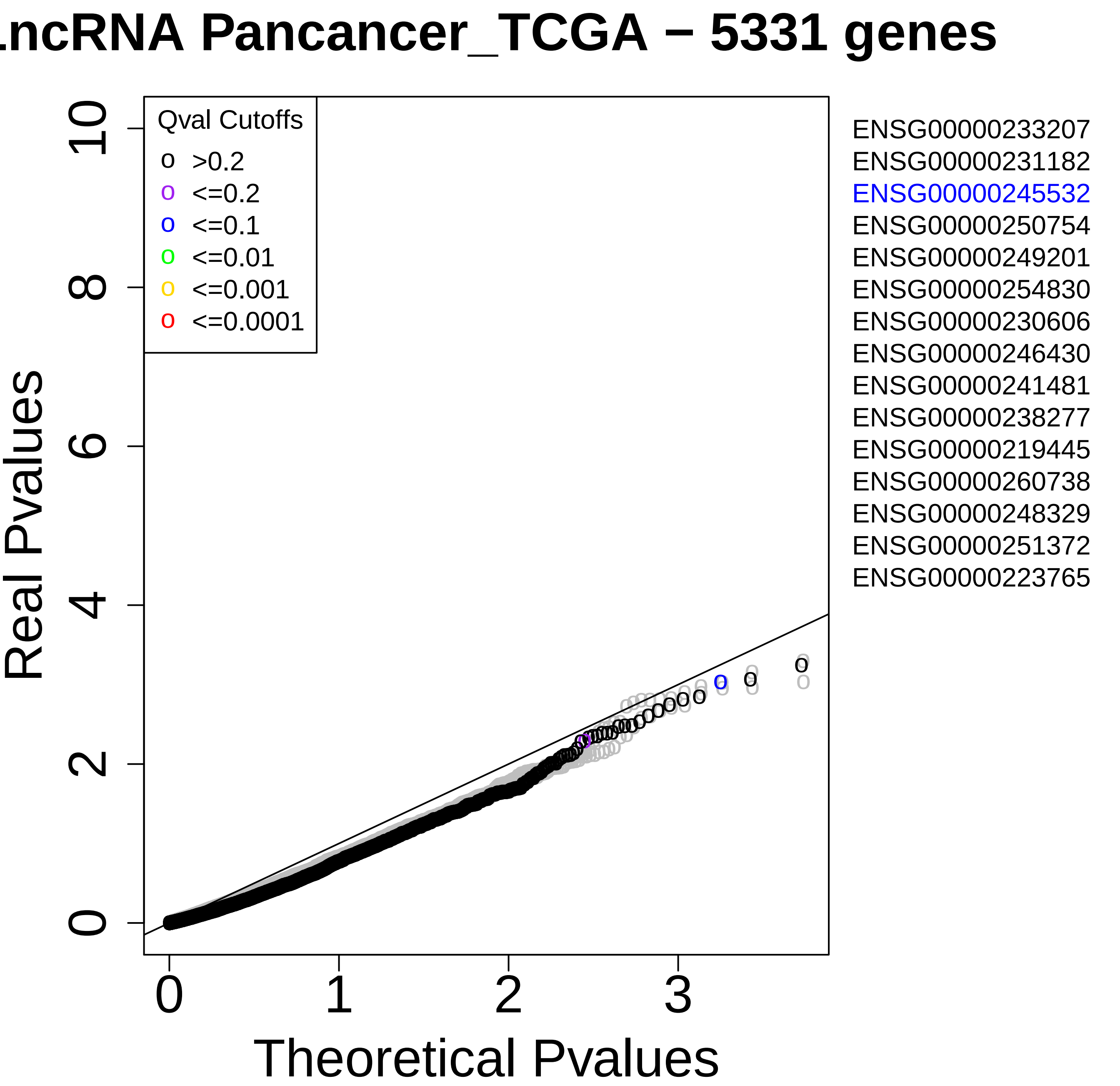

LncRNA Pancancer_TCGA – 5331 genes
Qval Cutoffs
o >0.2
o <=0.2
o <=0.1
o <=0.01
o <=0.001
o <=0.0001
Real Pvalues
Theoretical Pvalues
0
2
4
6
8
10
0
1
2
3
ENSG00000233207
ENSG00000231182
ENSG00000245532
ENSG00000250754
ENSG00000249201
ENSG00000254830
ENSG00000230606
ENSG00000246430
ENSG00000241481
ENSG00000238277
ENSG00000219445
ENSG00000260738
ENSG00000248329
ENSG00000251372
ENSG00000223765

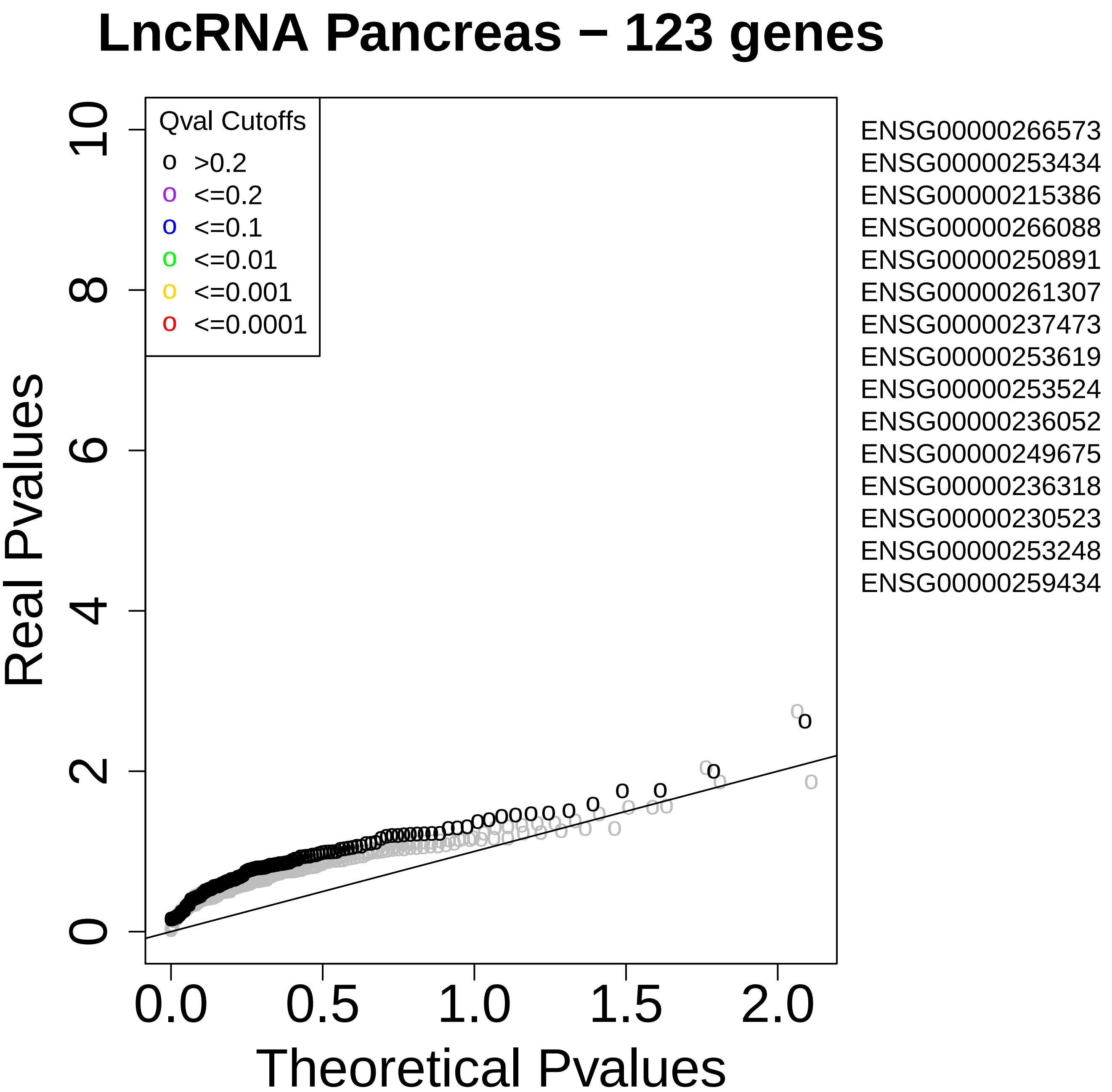

LncRNA Pancreas – 123 genes
Qval Cutoffs
o >0.2
o <=0.2
o <=0.1
o <=0.01
o <=0.001
o <=0.0001
Real Pvalues
Theoretical Pvalues
0
2
4
6
8
10
0.0
0.5
1.0
1.5
2.0
ENSG00000266573
ENSG00000253434
ENSG00000215386
ENSG00000266088
ENSG00000250891
ENSG00000261307
ENSG00000237473
ENSG00000253619
ENSG00000253524
ENSG00000236052
ENSG00000249675
ENSG00000236318
ENSG00000230523
ENSG00000253248
ENSG00000259434

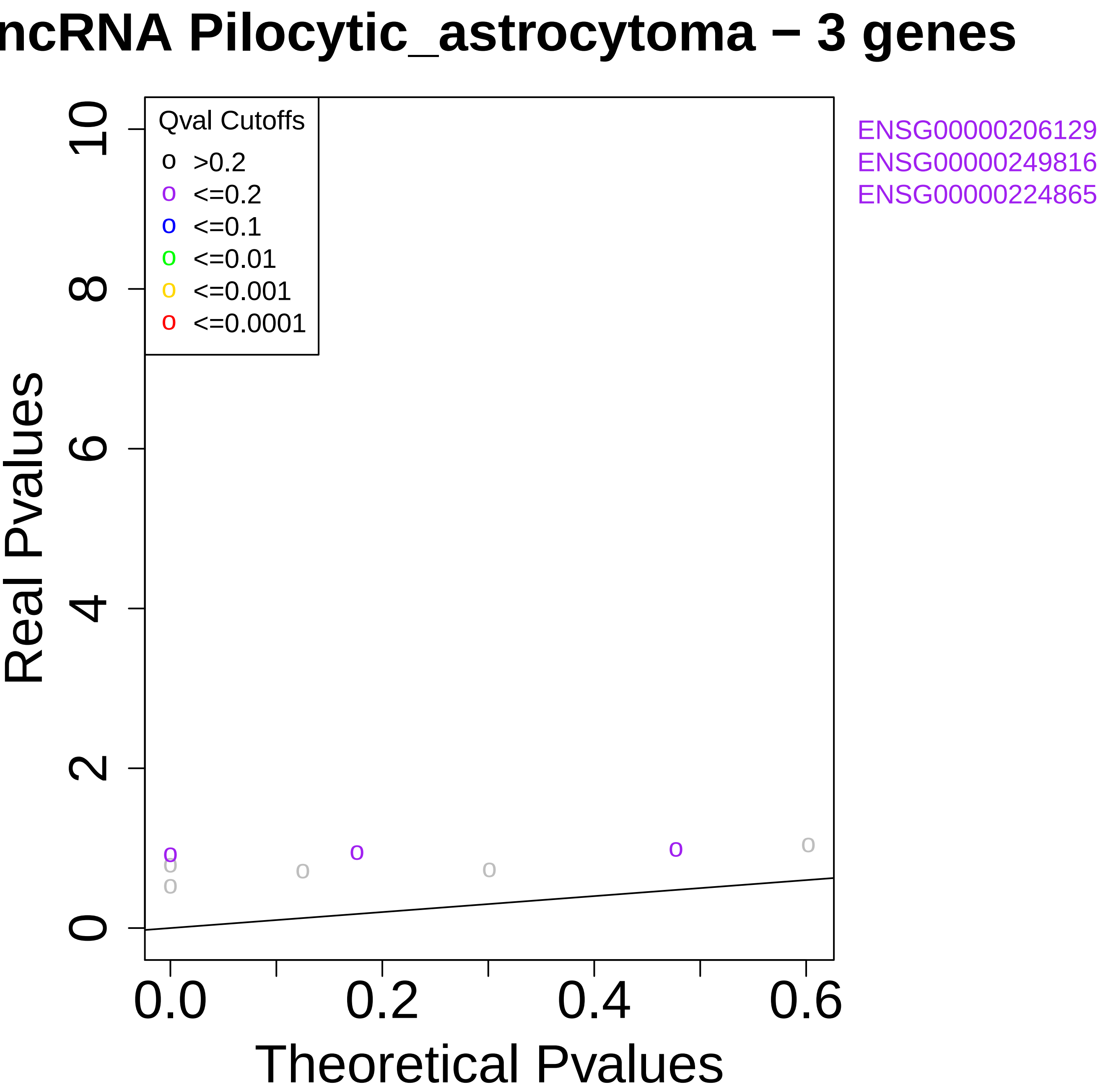
ncRNA Pilocytic_astrocytoma – 3 genes
Qval Cutoffs
o >0.2
o <=0.2
o <=0.1
o <=0.01
o <=0.001
o <=0.0001
ENSG00000206129
ENSG00000249816
ENSG00000224865
Real Pvalues
Theoretical Pvalues
0
2
4
6
8
10
0.0
0.2
0.4
0.6

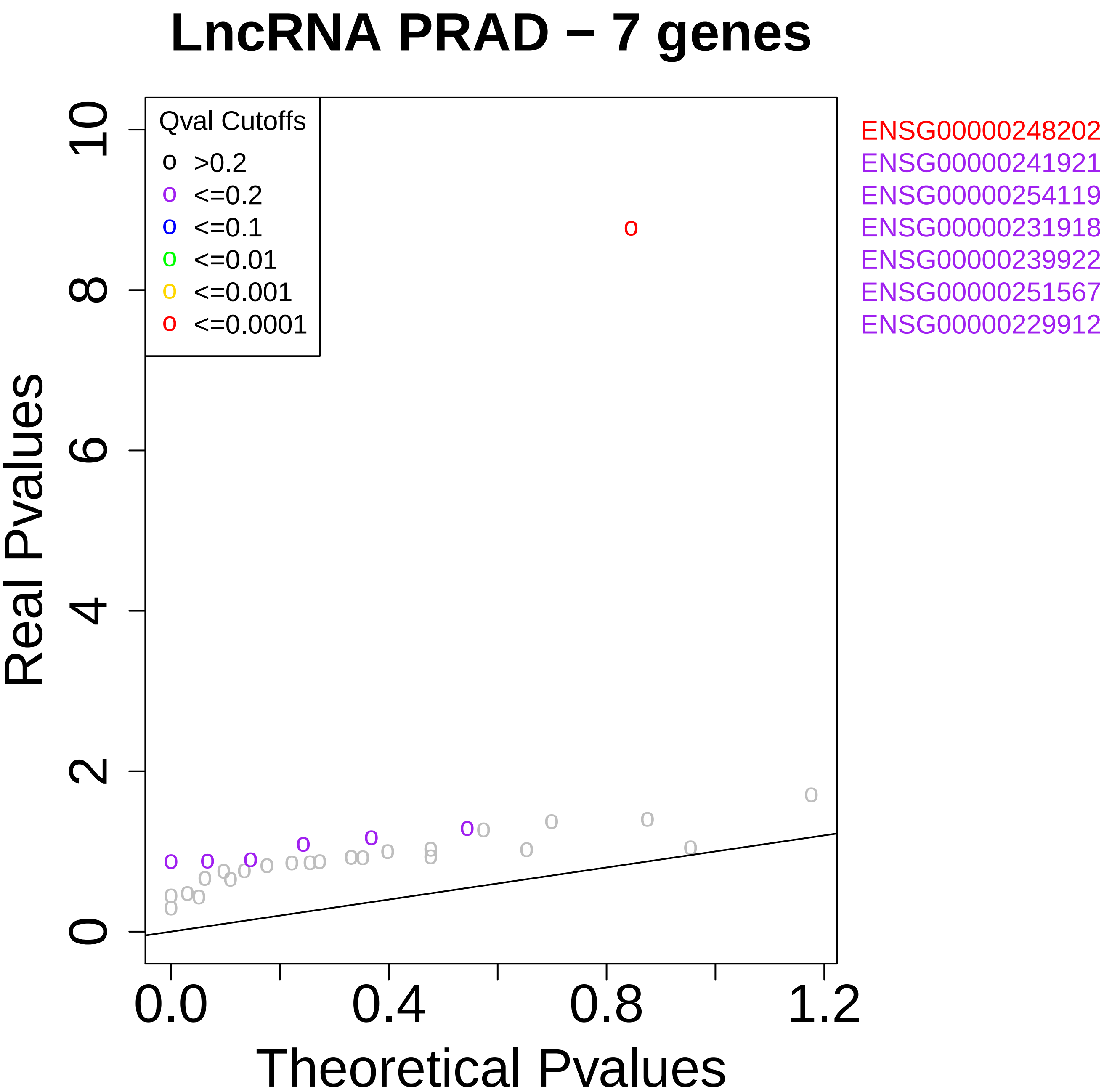
LncRNA PRAD − 7 genes
Qval Cutoffs
o >0.2
o <=0.2
o <=0.1
o <=0.01
o <=0.001
o <=0.0001
ENSG00000248202
ENSG00000241921
ENSG00000254119
ENSG00000231918
ENSG00000239922
ENSG00000251567
ENSG00000229912
Real Pvalues
Theoretical Pvalues
0
2
4
6
8
10
0.0
0.4
0.8
1.2

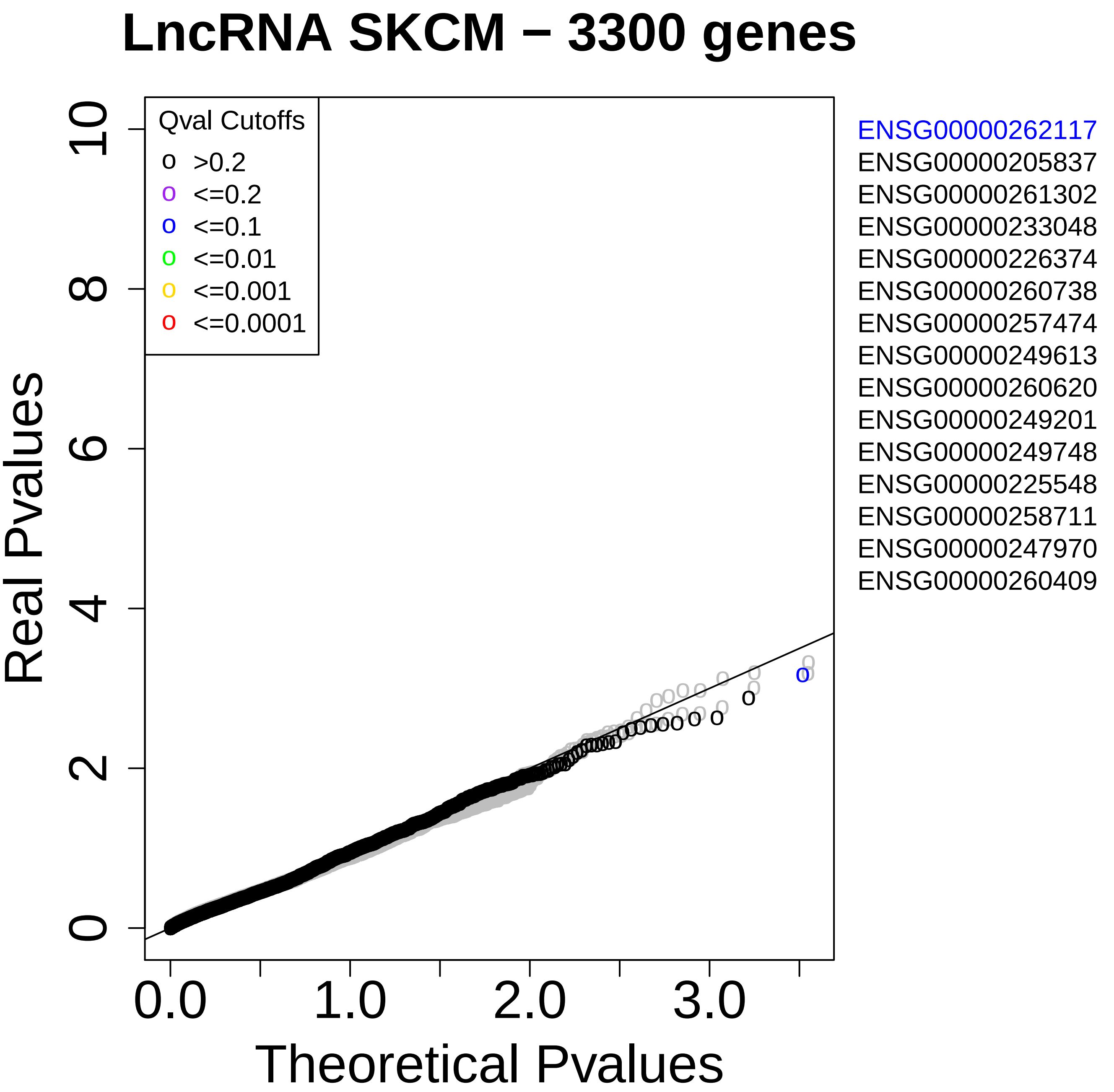

LncRNA SKCM − 3300 genes
Qval Cutoffs
o >0.2
o <=0.2
o <=0.1
o <=0.01
o <=0.001
o <=0.0001
Real Pvalues
0
2
4
6
8
10
0.0
1.0
2.0
3.0
Theoretical Pvalues
ENSG00000262117
ENSG00000205837
ENSG00000261302
ENSG00000233048
ENSG00000226374
ENSG00000260738
ENSG00000257474
ENSG00000249613
ENSG00000260620
ENSG00000249201
ENSG00000249748
ENSG00000225548
ENSG00000258711
ENSG00000247970
ENSG00000260409

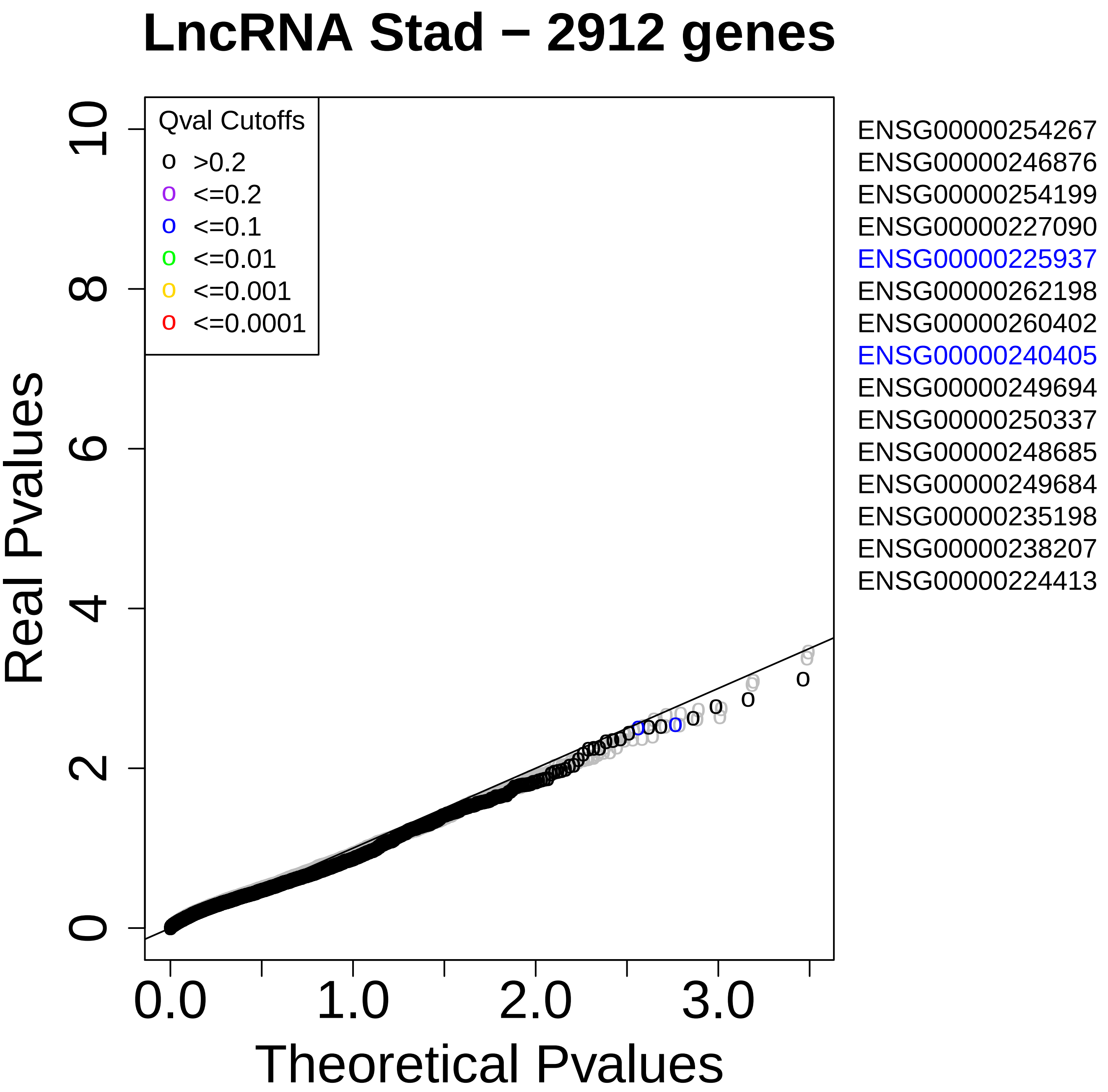

LncRNA Stad − 2912 genes
Qval Cutoffs
o >0.2
o <=0.2
o <=0.1
o <=0.01
o <=0.001
o <=0.0001
Real Pvalues
Theoretical Pvalues
0
2
4
6
8
10
0.0
1.0
2.0
3.0
ENSG00000254267
ENSG00000246876
ENSG00000254199
ENSG00000227090
ENSG00000225937
ENSG00000262198
ENSG00000260402
ENSG00000240405
ENSG00000249694
ENSG00000250337
ENSG00000248685
ENSG00000249684
ENSG00000235198
ENSG00000238207
ENSG00000224413

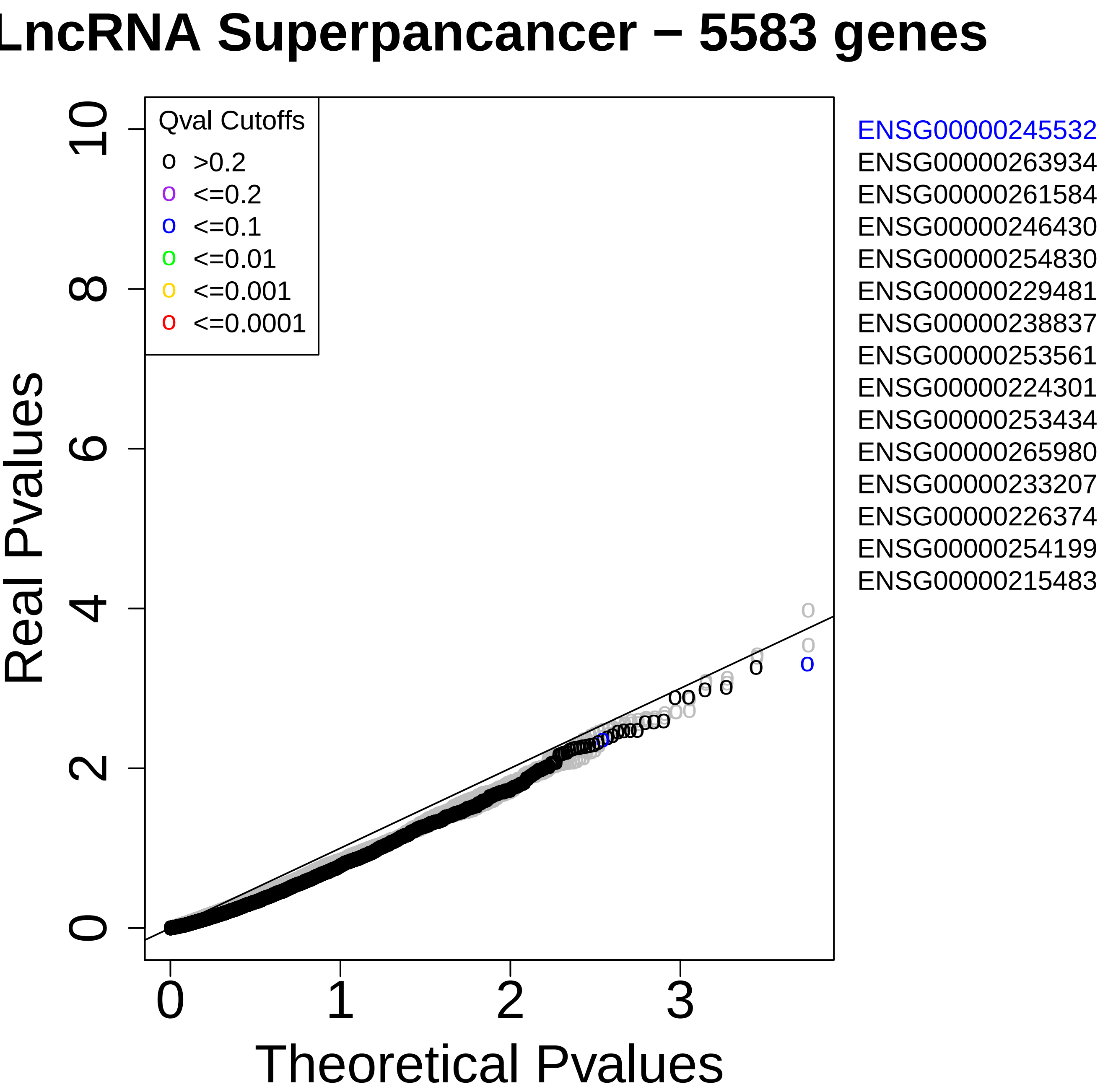

LncRNA Superpancancer – 5583 genes
Qval Cutoffs
o >0.2
o <=0.2
o <=0.1
o <=0.01
o <=0.001
o <=0.0001
Real Pvalues
Theoretical Pvalues
0
2
4
6
8
10
0
1
2
3
ENSG00000245532
ENSG00000263934
ENSG00000261584
ENSG00000246430
ENSG00000254830
ENSG00000229481
ENSG00000238837
ENSG00000253561
ENSG00000224301
ENSG00000253434
ENSG00000265980
ENSG00000233207
ENSG00000226374
ENSG00000254199
ENSG00000215483

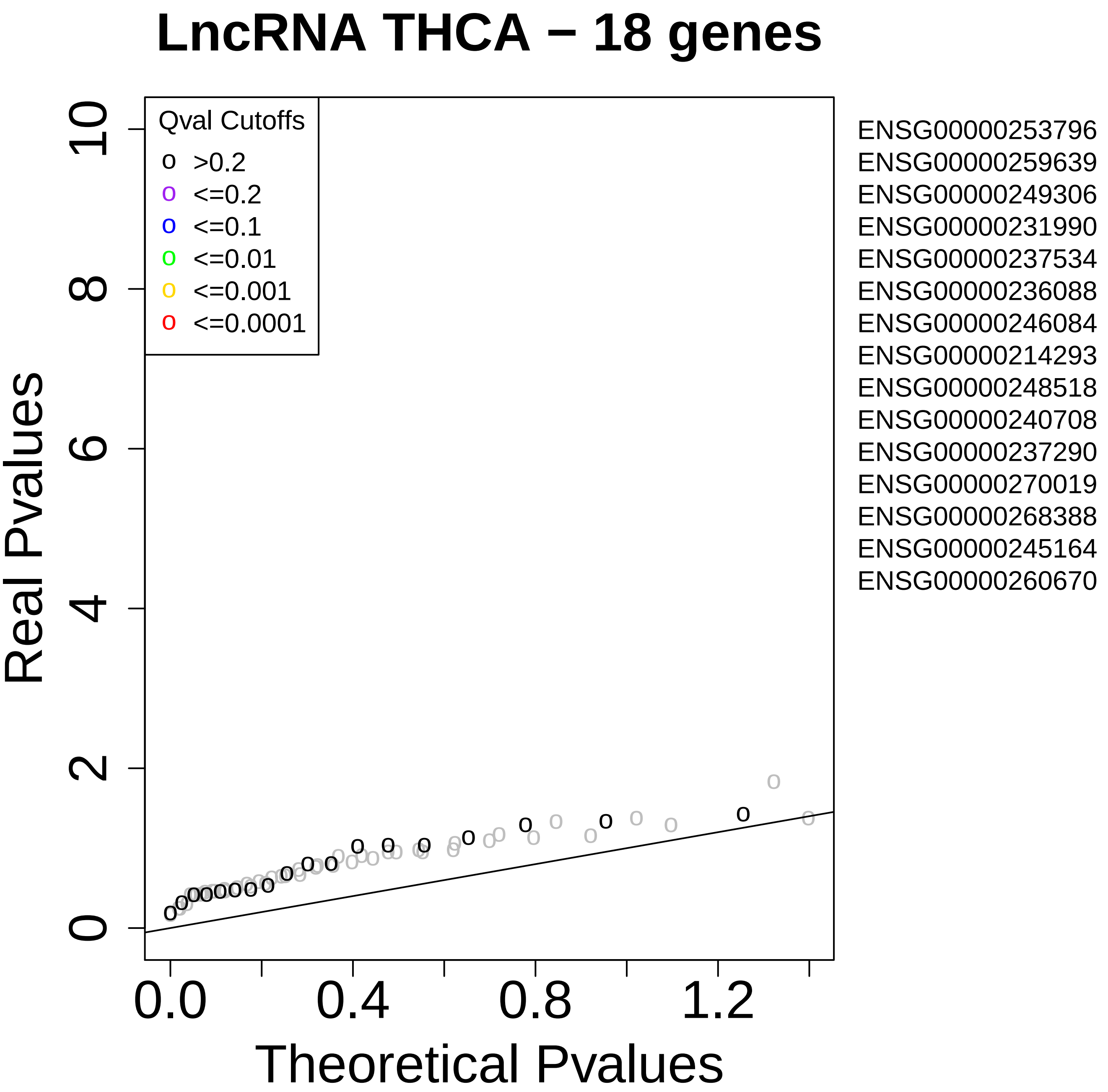
LncRNA THCA − 18 genes
Qval Cutoffs
o >0.2
o <=0.2
o <=0.1
o <=0.01
o <=0.001
o <=0.0001
Real Pvalues
0
2
4
6
8
10
Theoretical Pvalues
0.0
0.4
0.8
1.2
ENSG00000253796
ENSG00000259639
ENSG00000249306
ENSG00000231990
ENSG00000237534
ENSG00000236088
ENSG00000246084
ENSG00000214293
ENSG00000248518
ENSG00000240708
ENSG00000237290
ENSG00000270019
ENSG00000268388
ENSG00000245164
ENSG00000260670

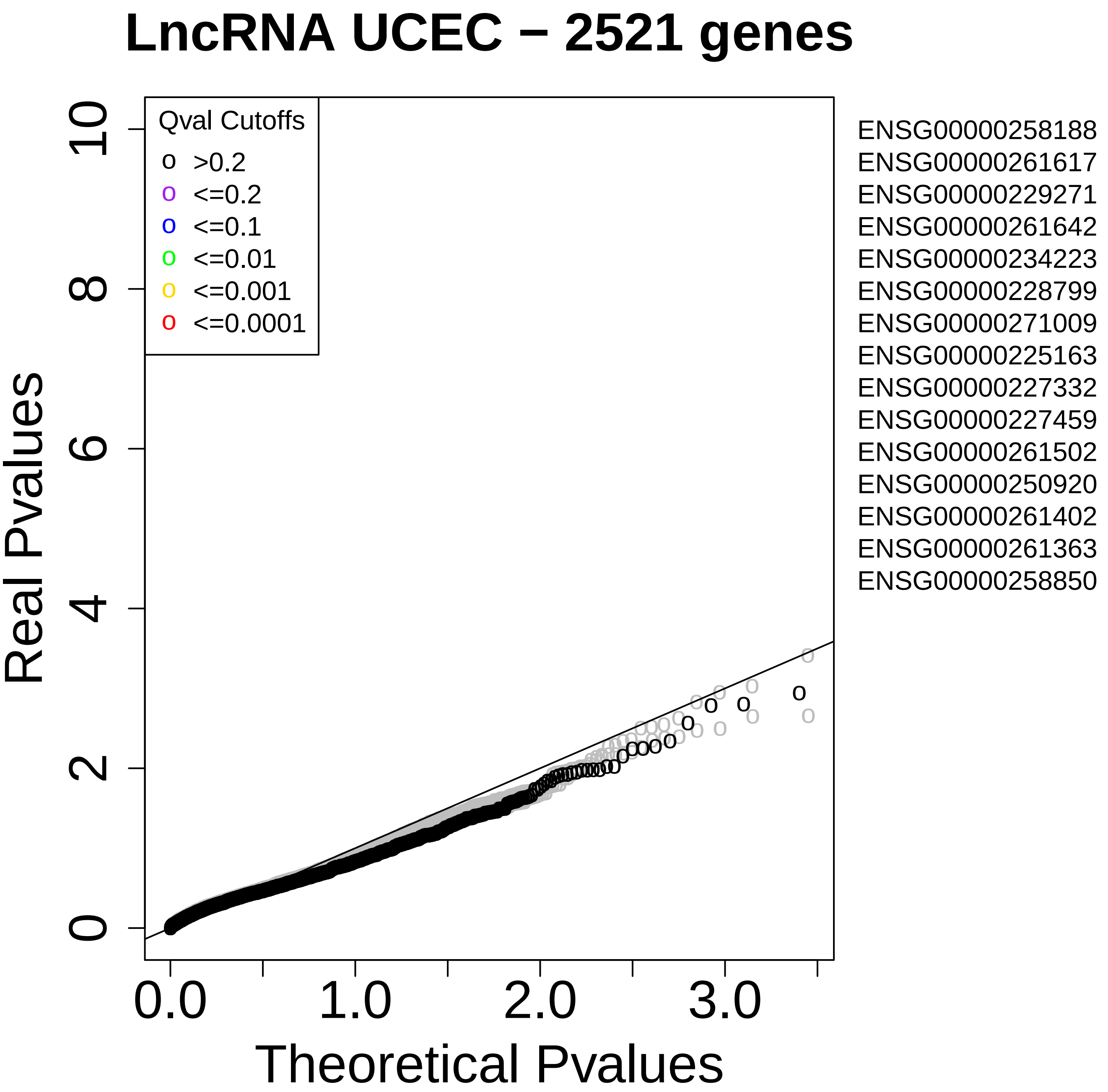
LncRNA UCEC − 2521 genes
Qval Cutoffs
o >0.2
o <=0.2
o <=0.1
o <=0.01
o <=0.001
o <=0.0001
Real Pvalues
Theoretical Pvalues
0
2
4
6
8
10
0.0
1.0
2.0
3.0
ENSG00000258188
ENSG00000261617
ENSG00000229271
ENSG00000261642
ENSG00000234223
ENSG00000228799
ENSG00000271009
ENSG00000225163
ENSG00000227332
ENSG00000227459
ENSG00000261502
ENSG00000250920
ENSG00000261402
ENSG00000261363
ENSG00000258850

Supplementary Figure S4

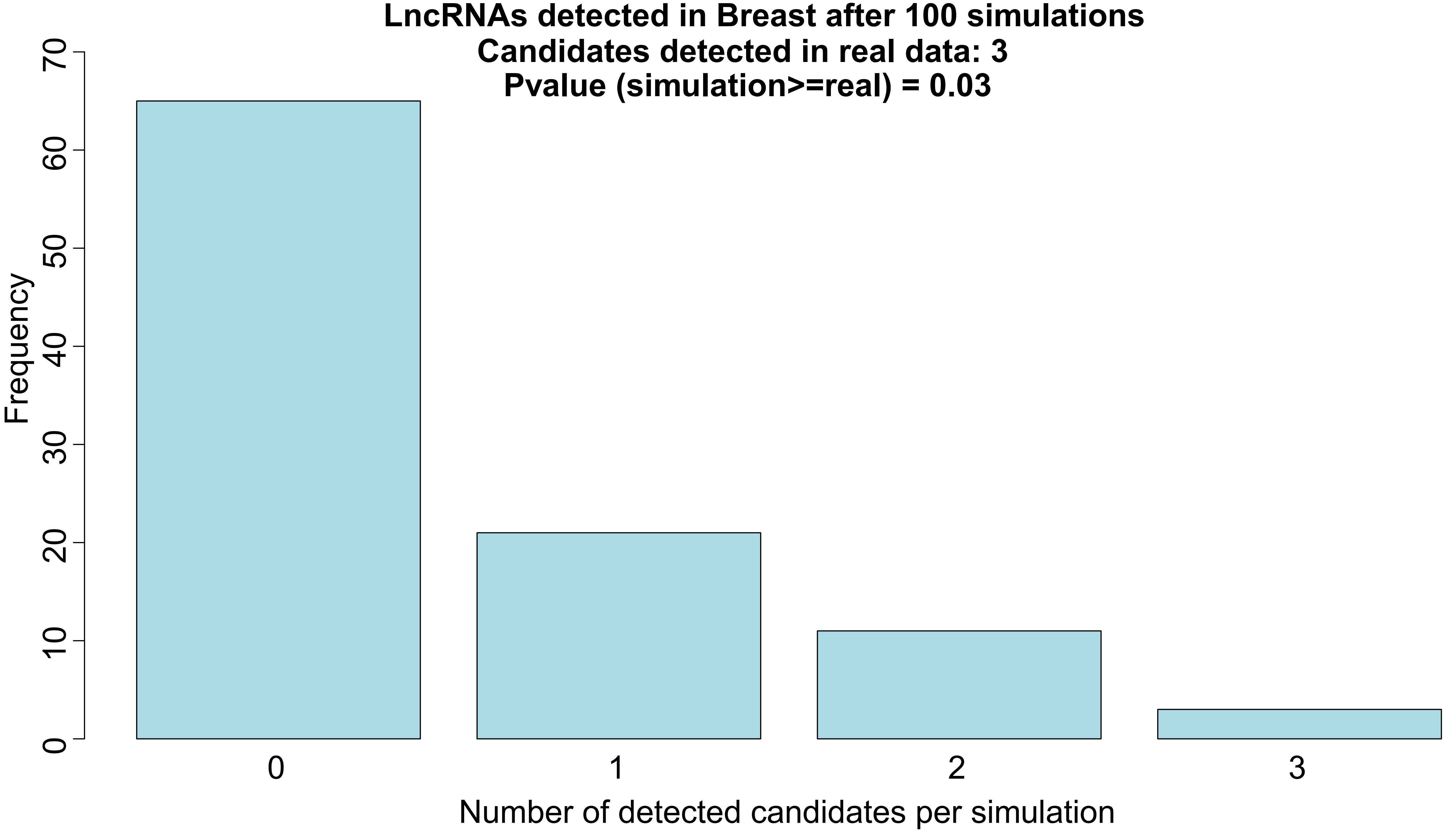

Supplementary Figure S5

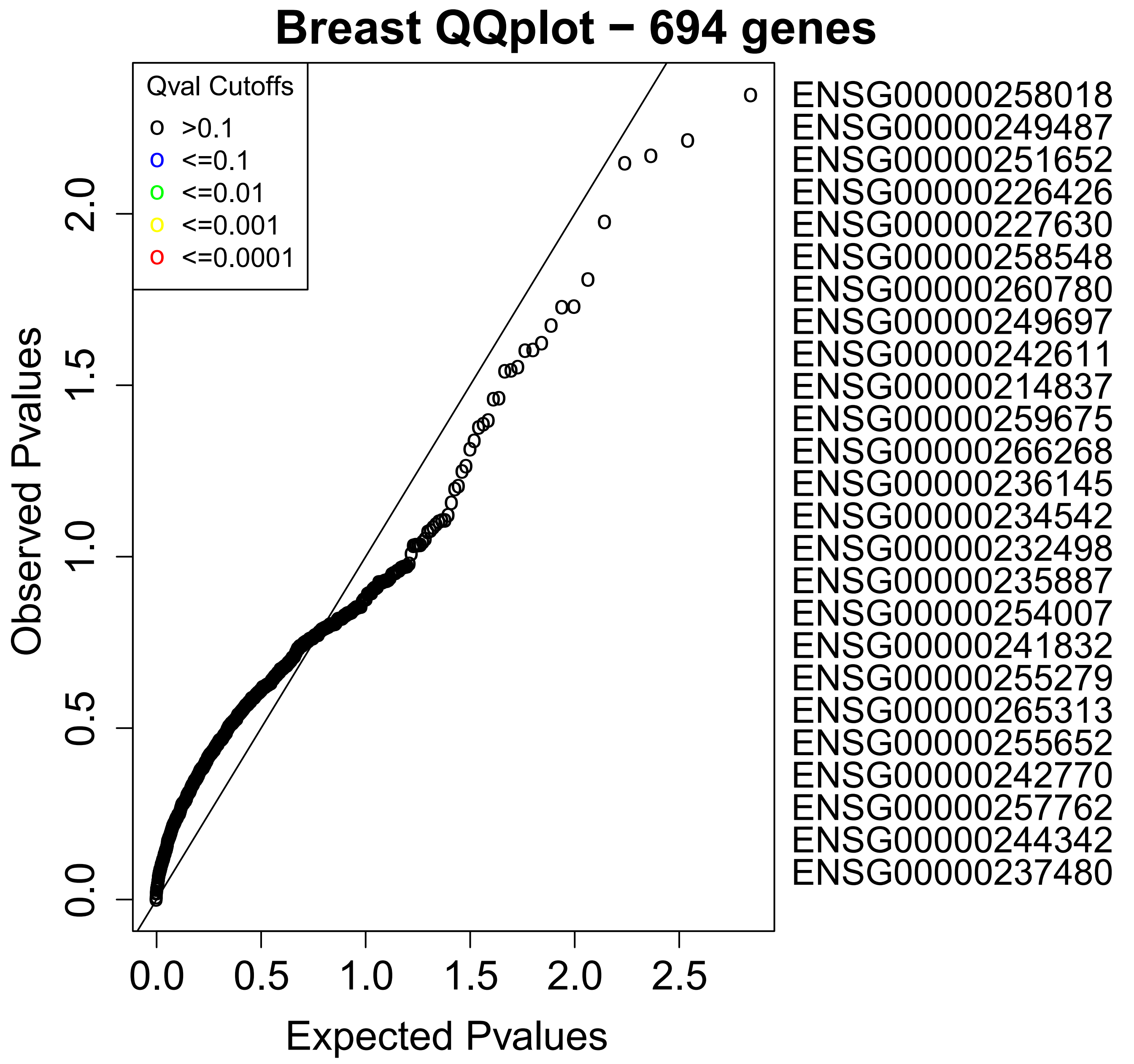

Supplementary Figure S6

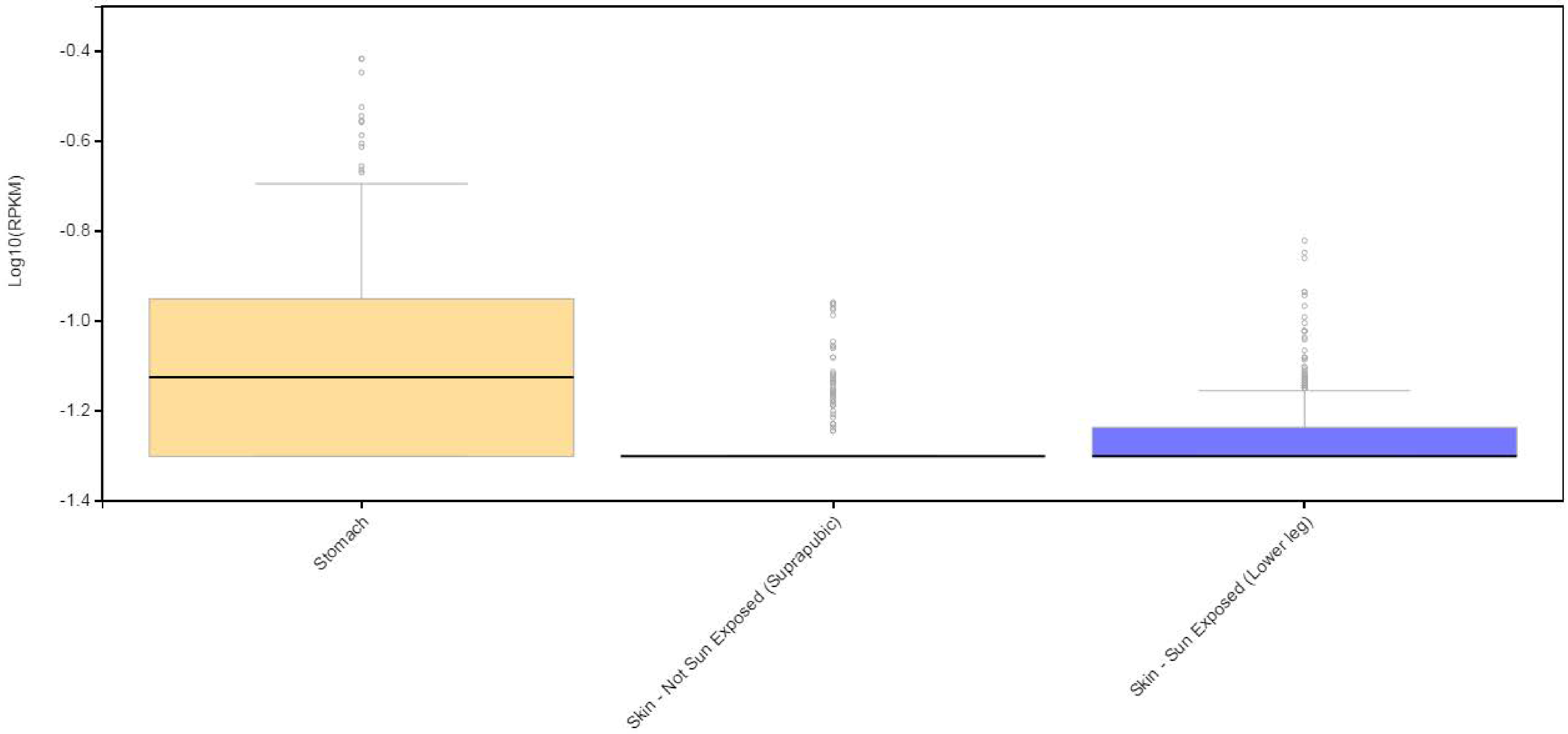

Supplementary Figure S7

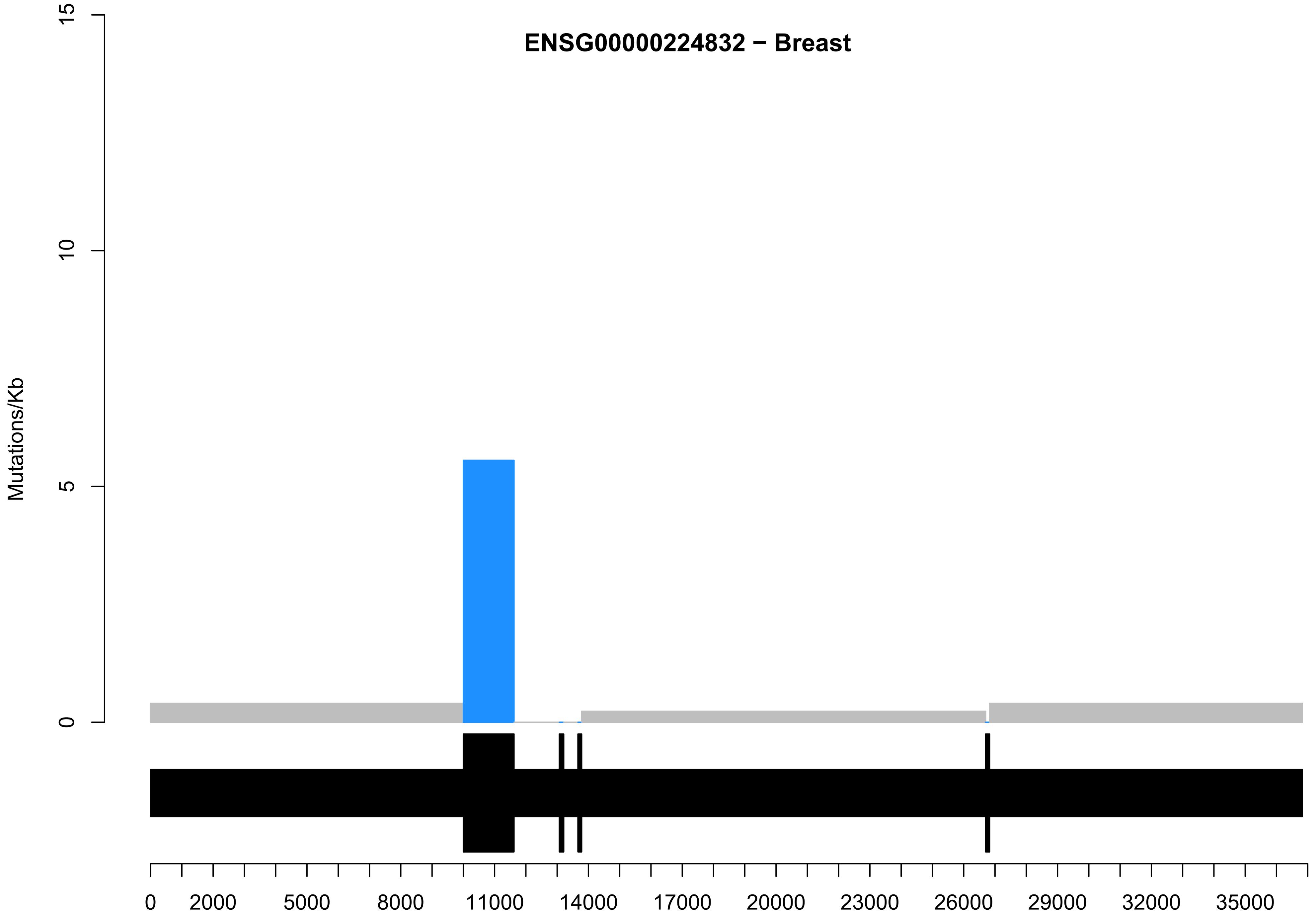

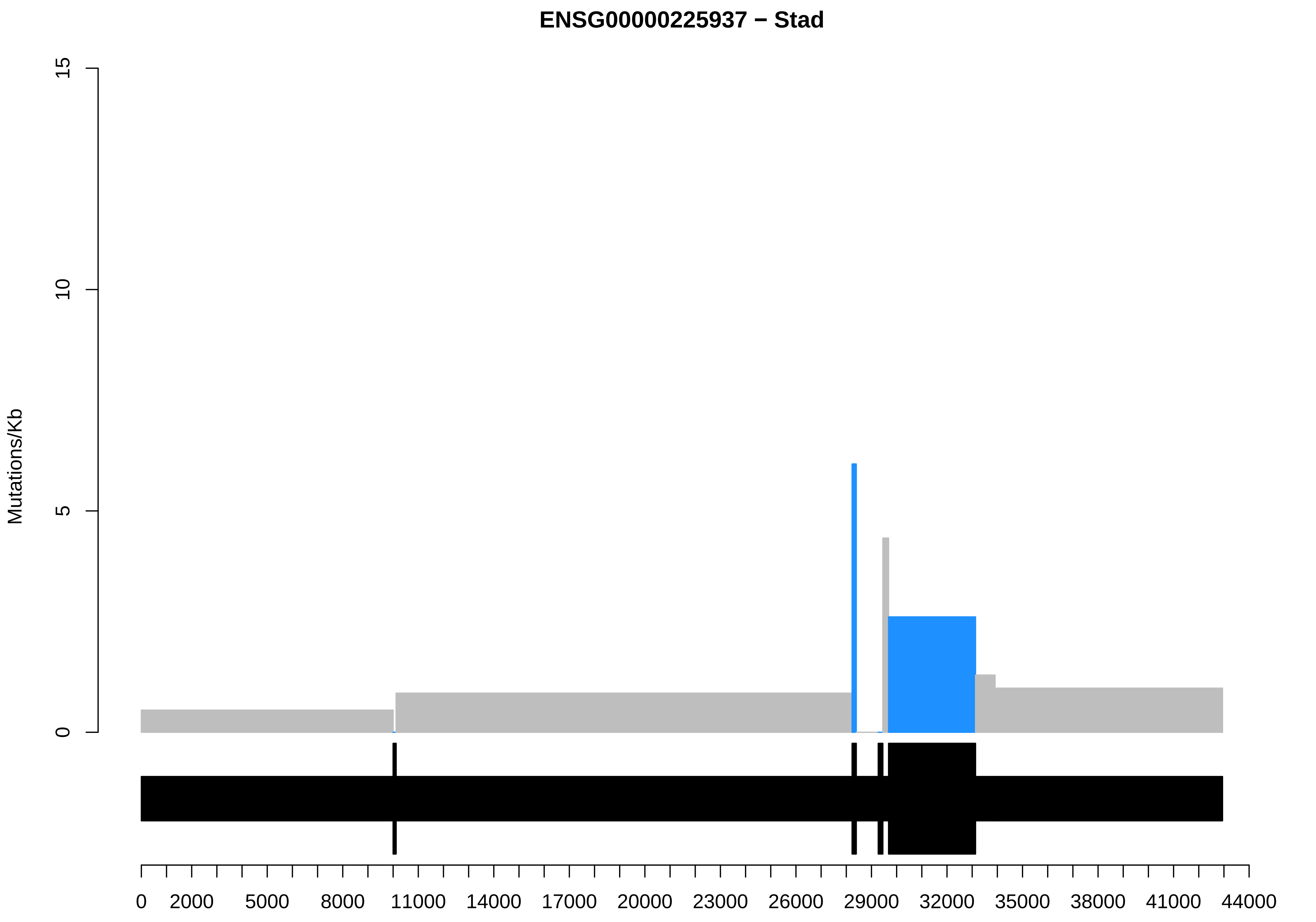

ENSG00000225937 − Stad
Mutations/Kb
0
5
10
15
0
2000
5000
8000
11000
14000
17000
20000
23000
26000
29000
32000
35000
38000
41000
44000

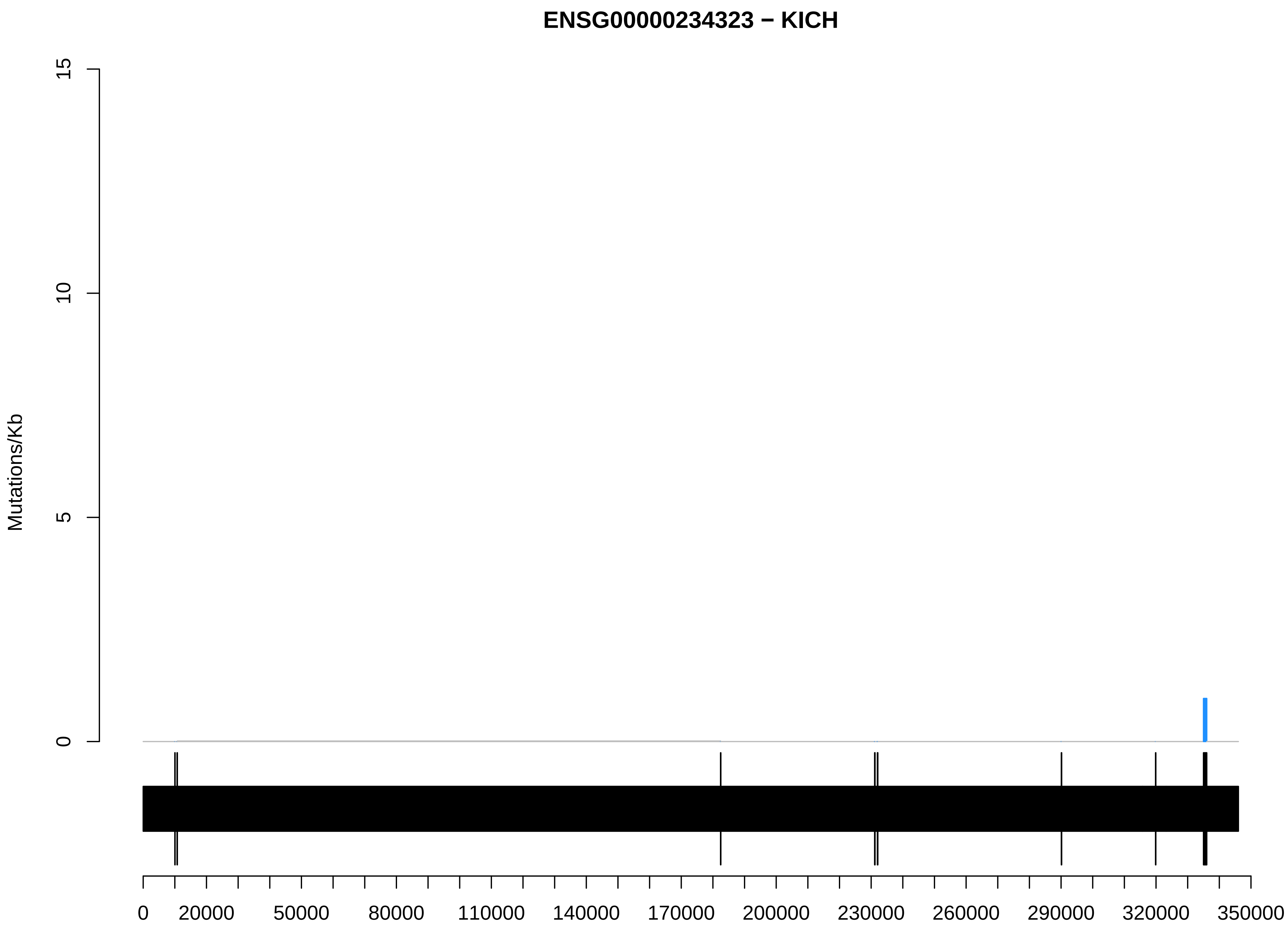
ENSG00000234323 − KICH
Mutations/Kb
0
5
10
15
0
20000
50000
80000
110000
140000
170000
200000
230000
260000
290000
320000
350000

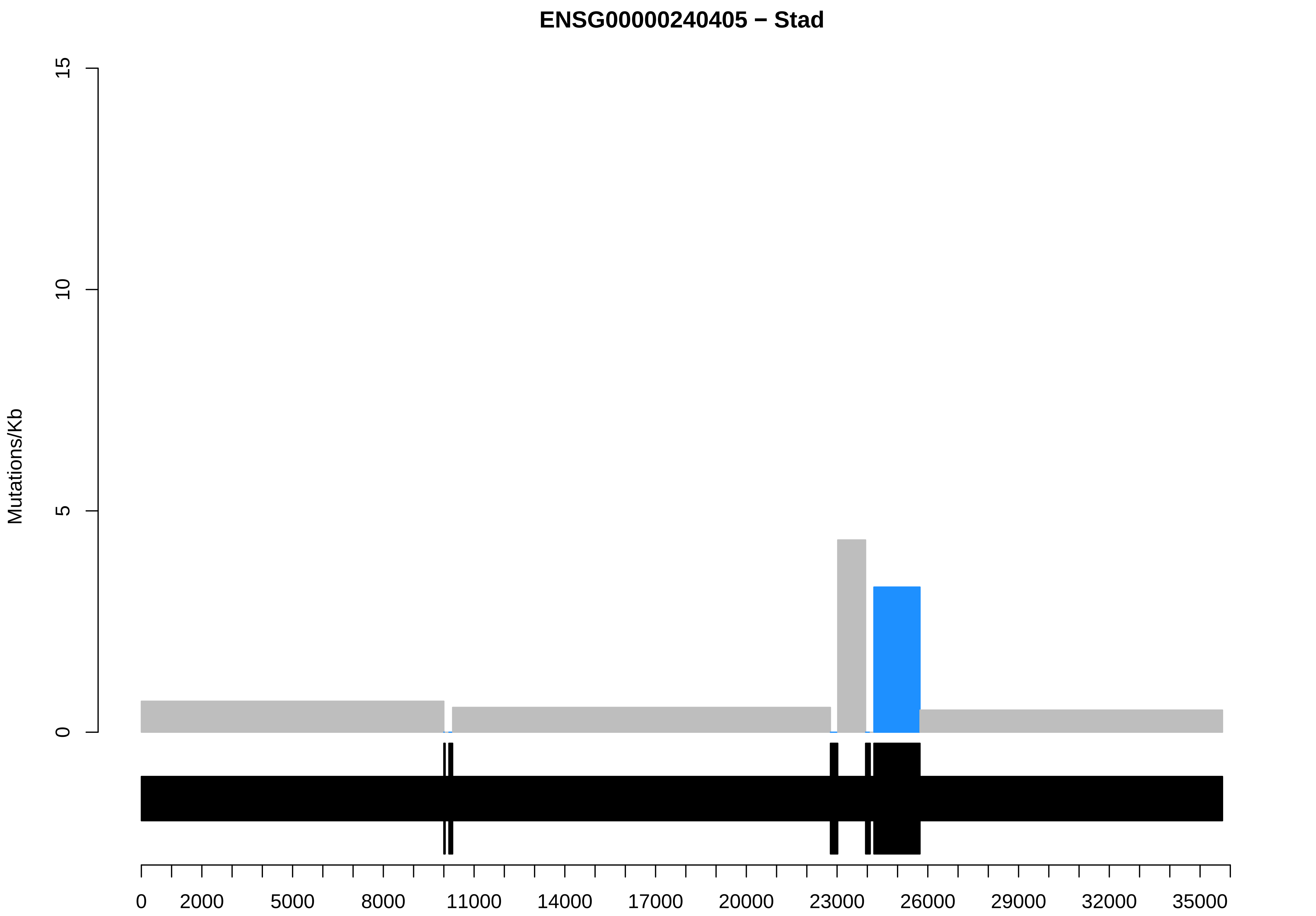
ENSG00000240405 − Stad
Mutations/Kb
0
5
10
15
0
2000
5000
8000
11000
14000
17000
20000
23000
26000
29000
32000
35000

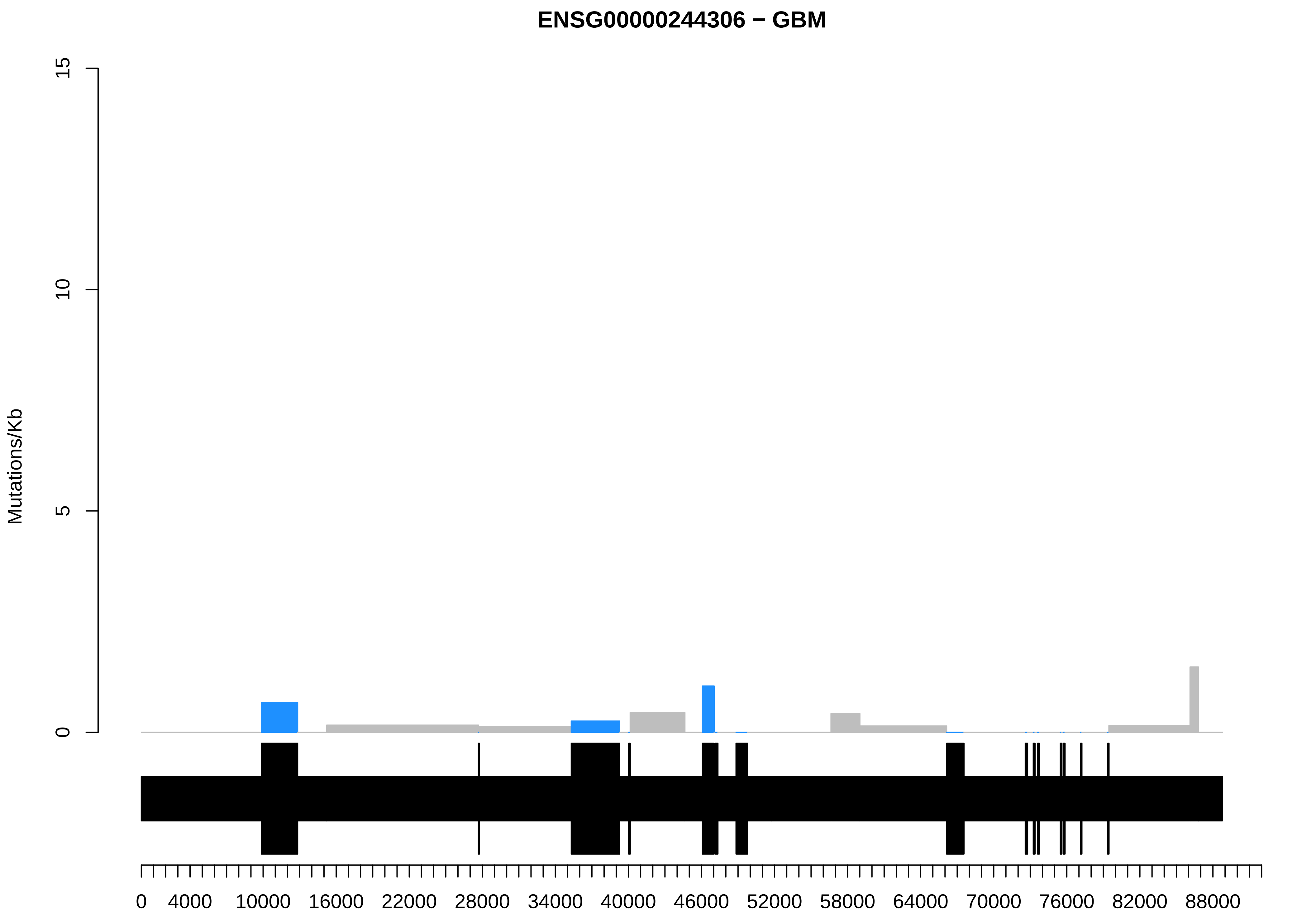

ENSG00000244306 − GBM
Mutations/Kb
0
5
10
15
0
4000
10000
16000
22000
28000
34000
40000
46000
52000
58000
64000
70000
76000
82000
88000

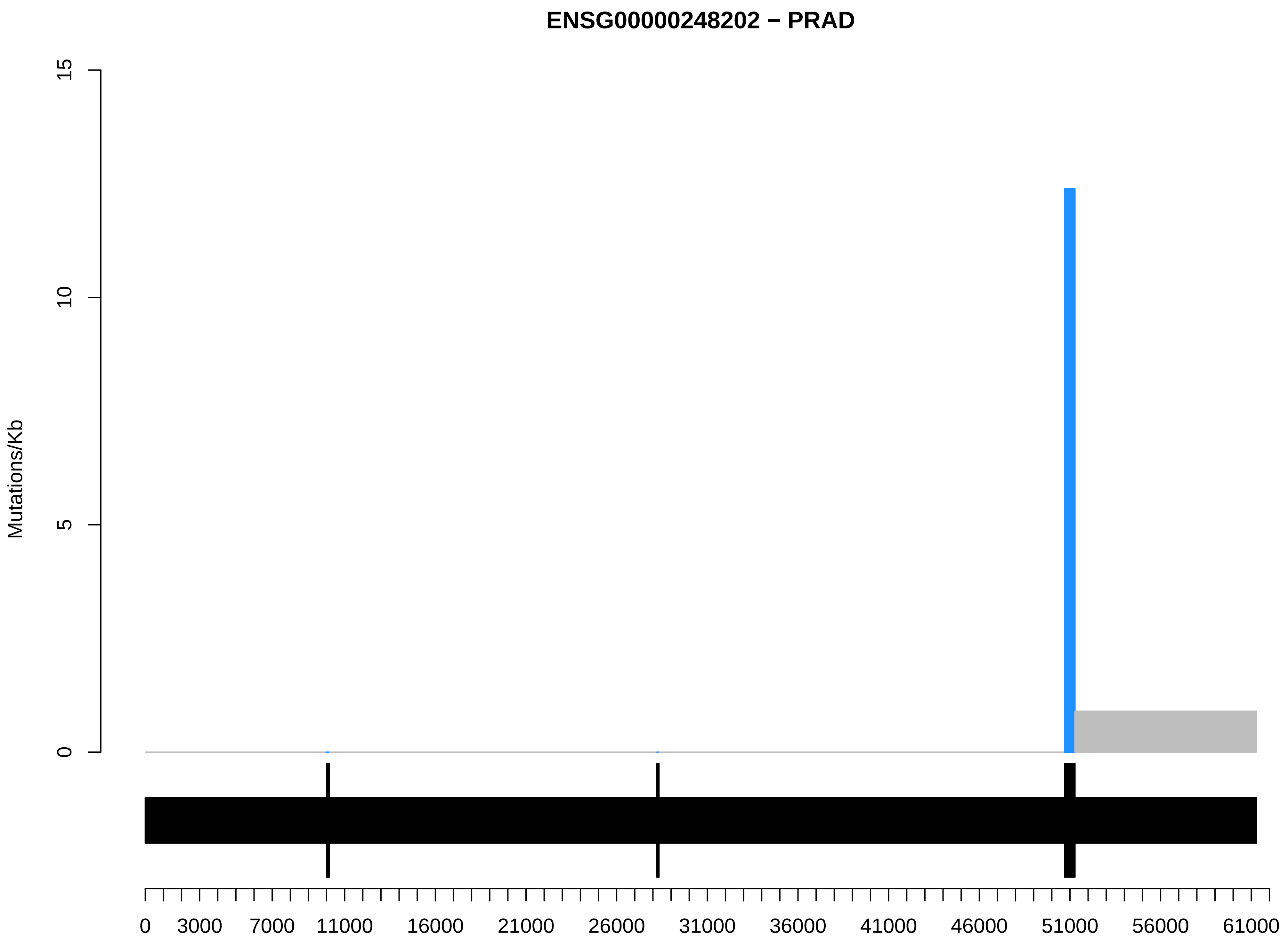

ENSG00000248202 − PRAD
Mutations/Kb
0
5
10
15
0
3000
7000
11000
16000
21000
26000
31000
36000
41000
46000
51000
56000
61000

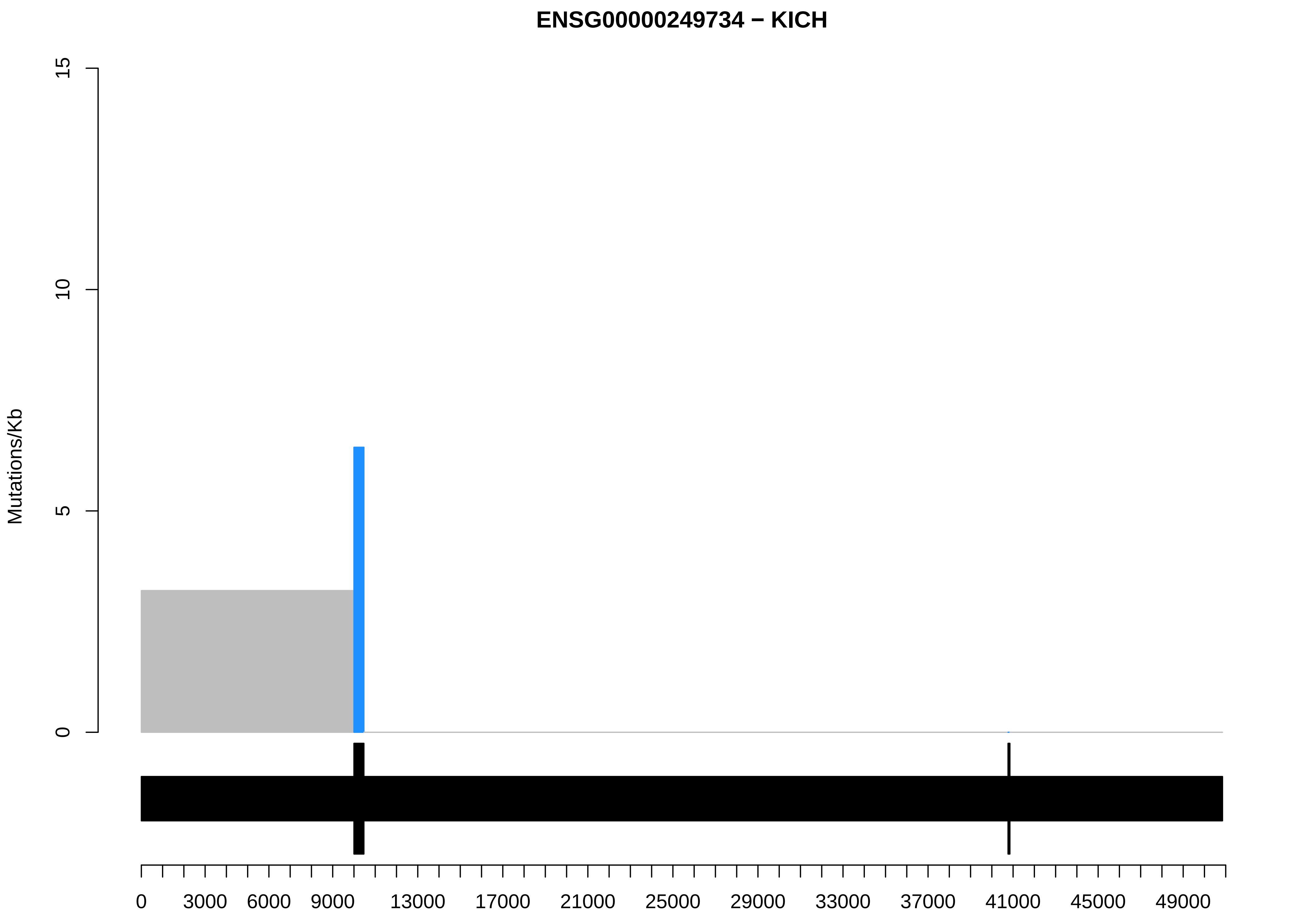
ENSG00000249734 − KICH
Mutations/Kb
0
5
10
15
0
3000
6000
9000
13000
17000
21000
25000
29000
33000
37000
41000
45000
49000

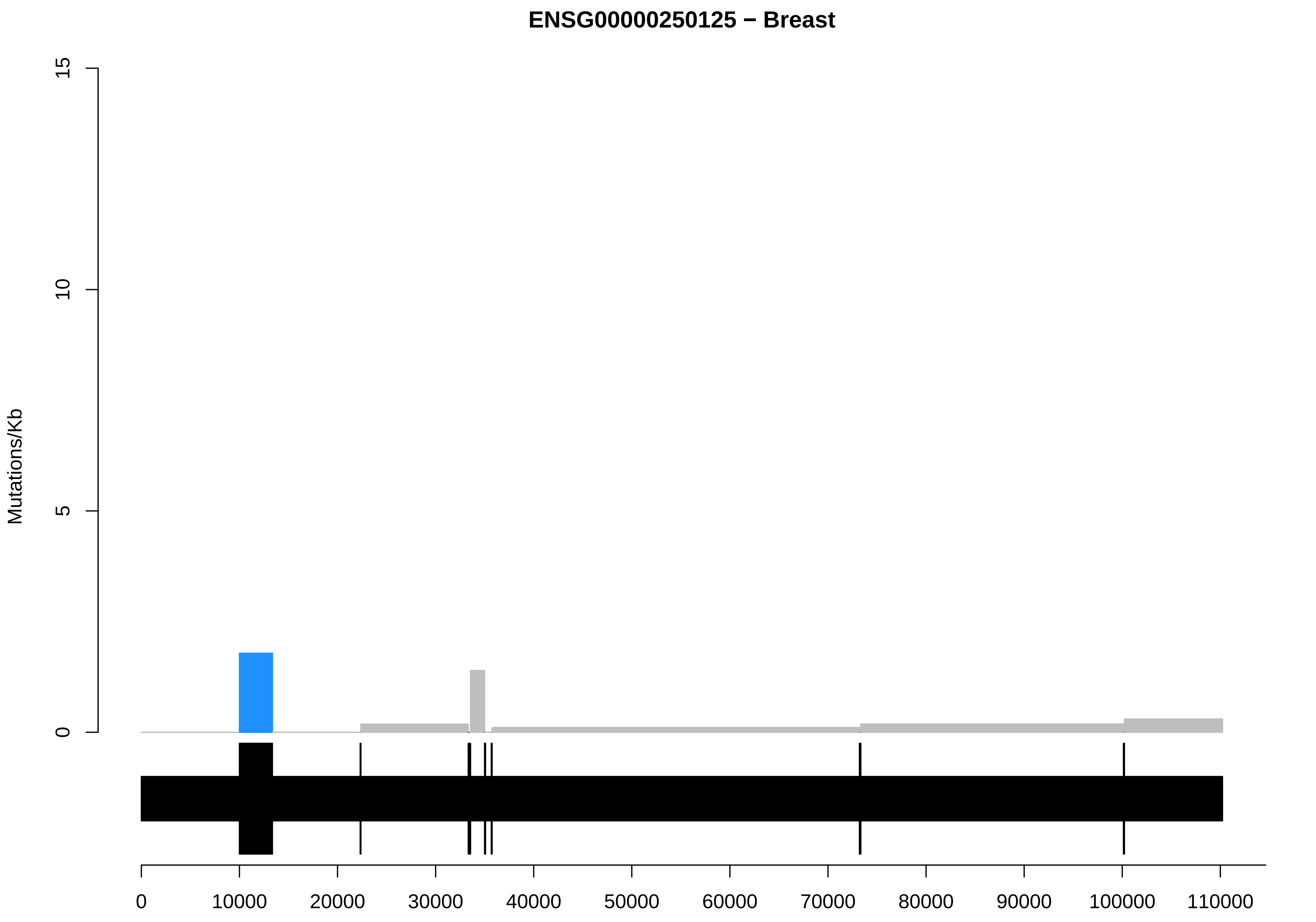

ENSG00000250125 − Breast
Mutations/Kb
0
5
10
15
0
10000
20000
30000
40000
50000
60000
70000
80000
90000
100000
110000

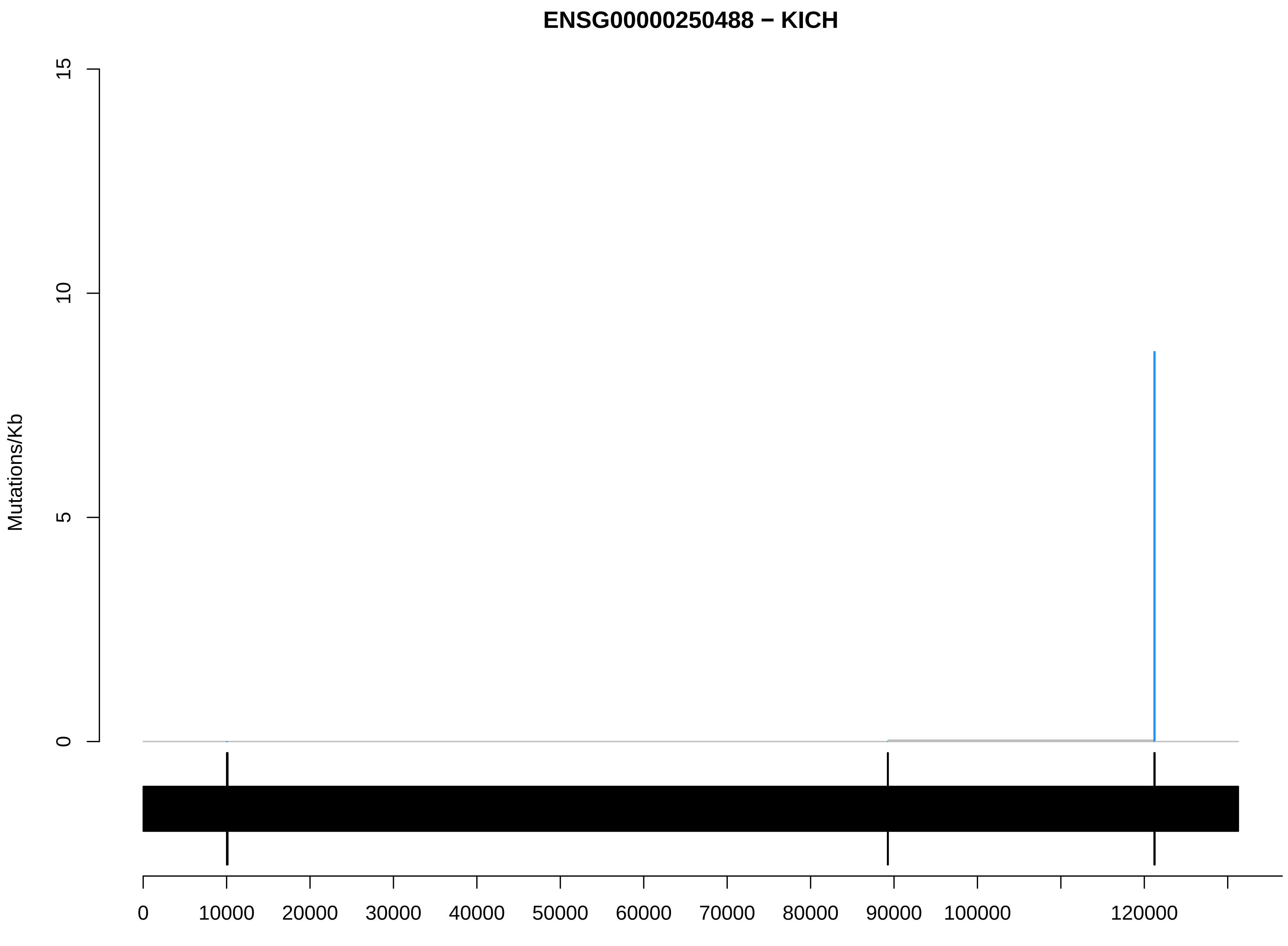

ENSG00000250488 − KICH
Mutations/Kb
0
5
10
15
0
10000
20000
30000
40000
50000
60000
70000
80000
90000
100000
120000

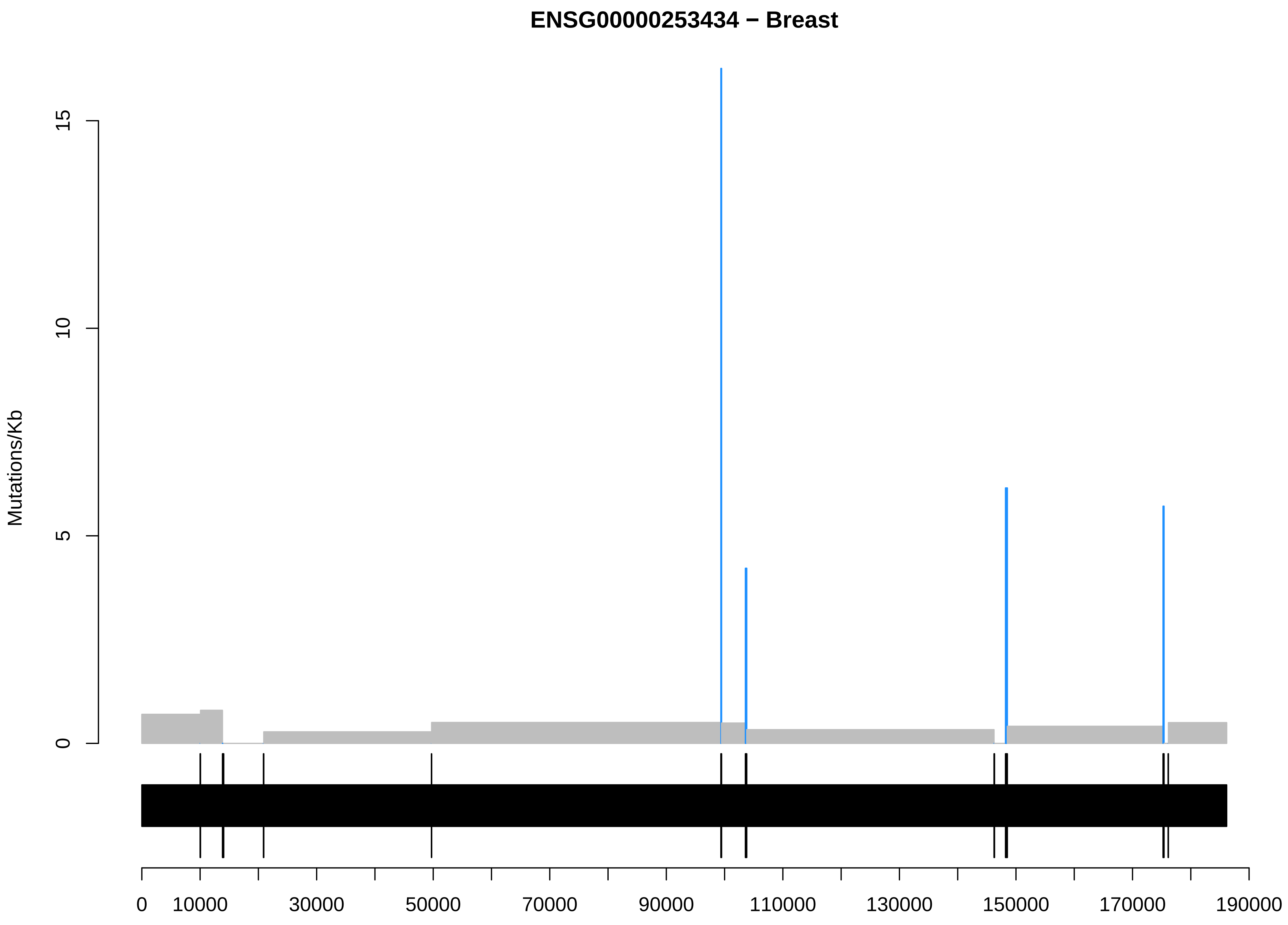

ENSG00000253434 − Breast
Mutations/Kb
0
5
10
15
0
10000
30000
50000
70000
90000
110000
130000
150000
170000
190000

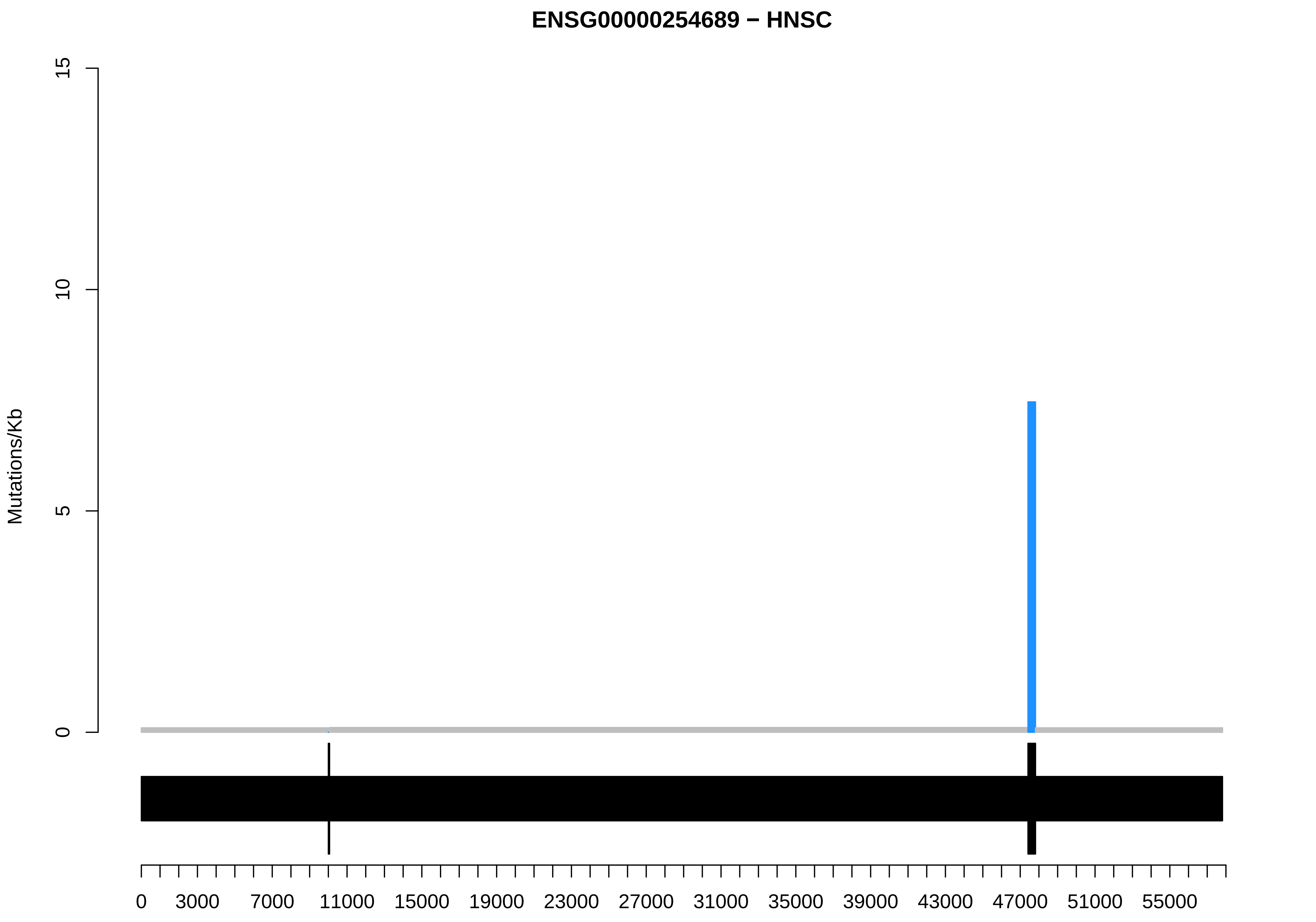
ENSG00000254689 − HNSC
Mutations/Kb
0
5
10
15
0
3000
7000
11000
15000
19000
23000
27000
31000
35000
39000
43000
47000
51000
55000

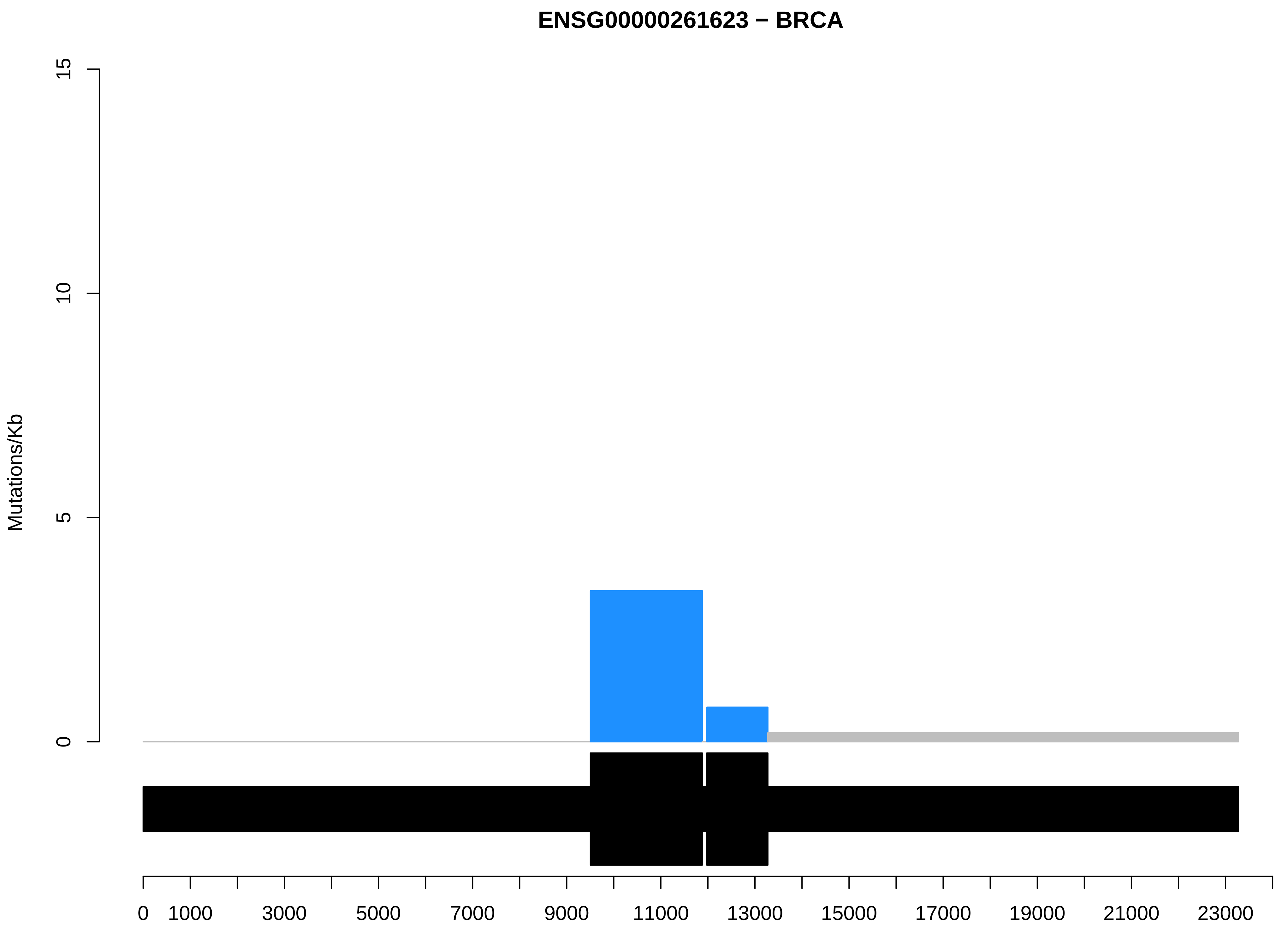
ENSG00000261623 − BRCA
Mutations/Kb
0
5
10
15
0
1000
3000
5000
7000
9000
11000
13000
15000
17000
19000
21000
23000

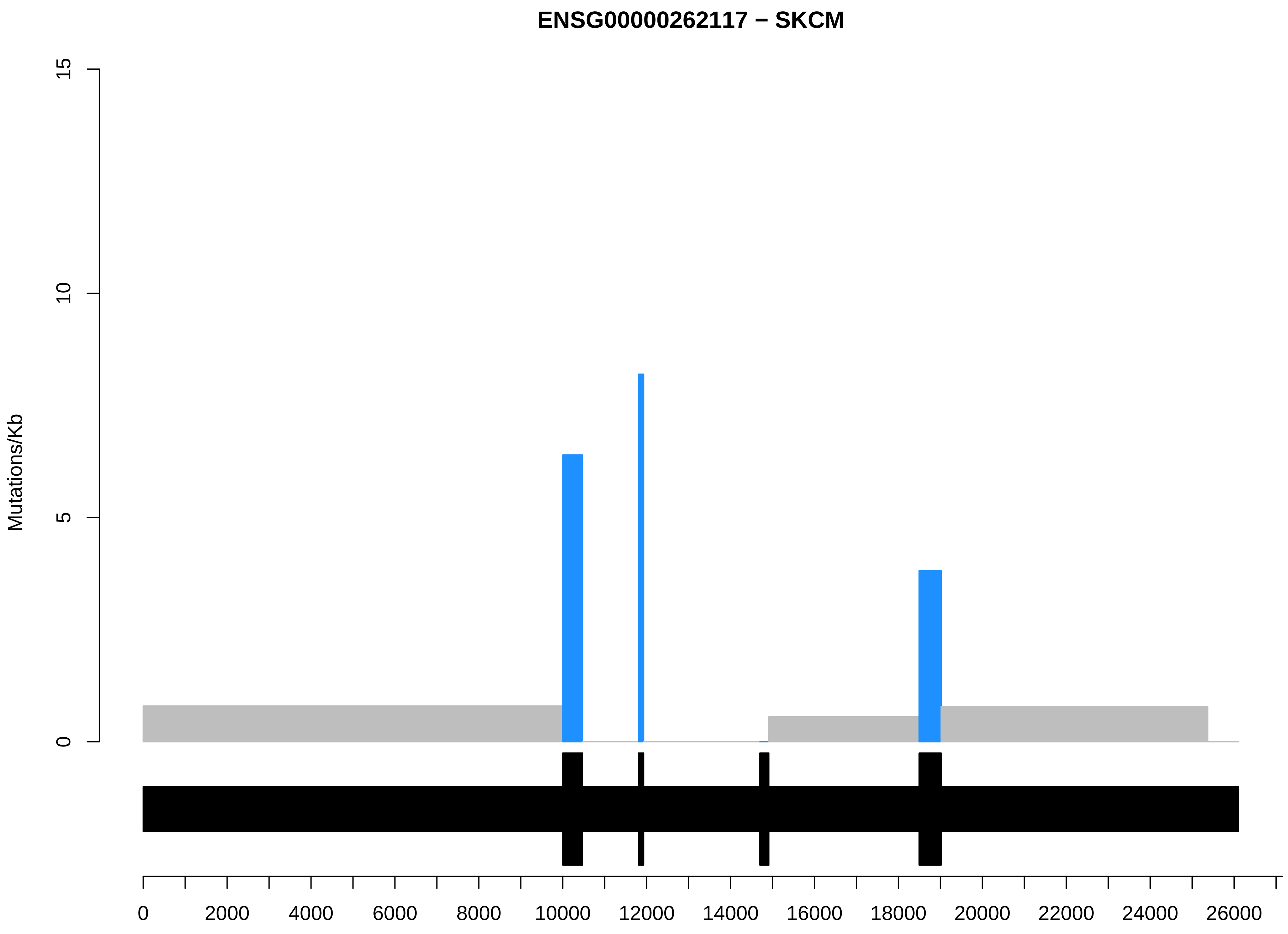

ENSG00000262117 − SKCM
Mutations/Kb
0
5
10
15
0
2000
4000
6000
8000
10000
12000
14000
16000
18000
20000
22000
24000
26000

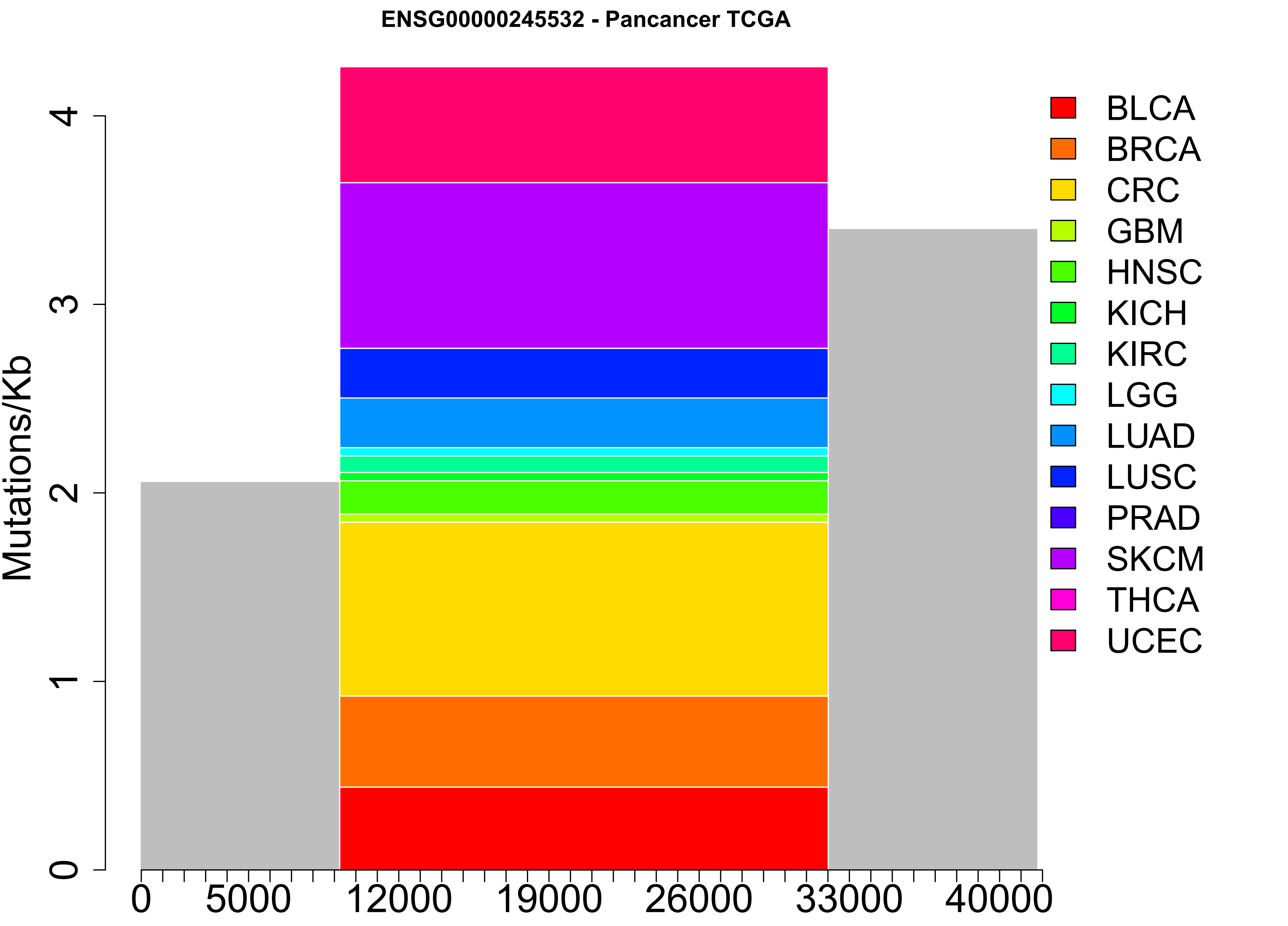
ENSG00000245532 - Pancancer TCGA
Mutations/Kb
0
1
2
3
4
0
5000
12000
19000
26000
33000
40000
BLCA
BRCA
CRC
GBM
HNSC
KICH
KIRC
LGG
LUAD
LUSC
PRAD
SKCM
THCA
UCEC

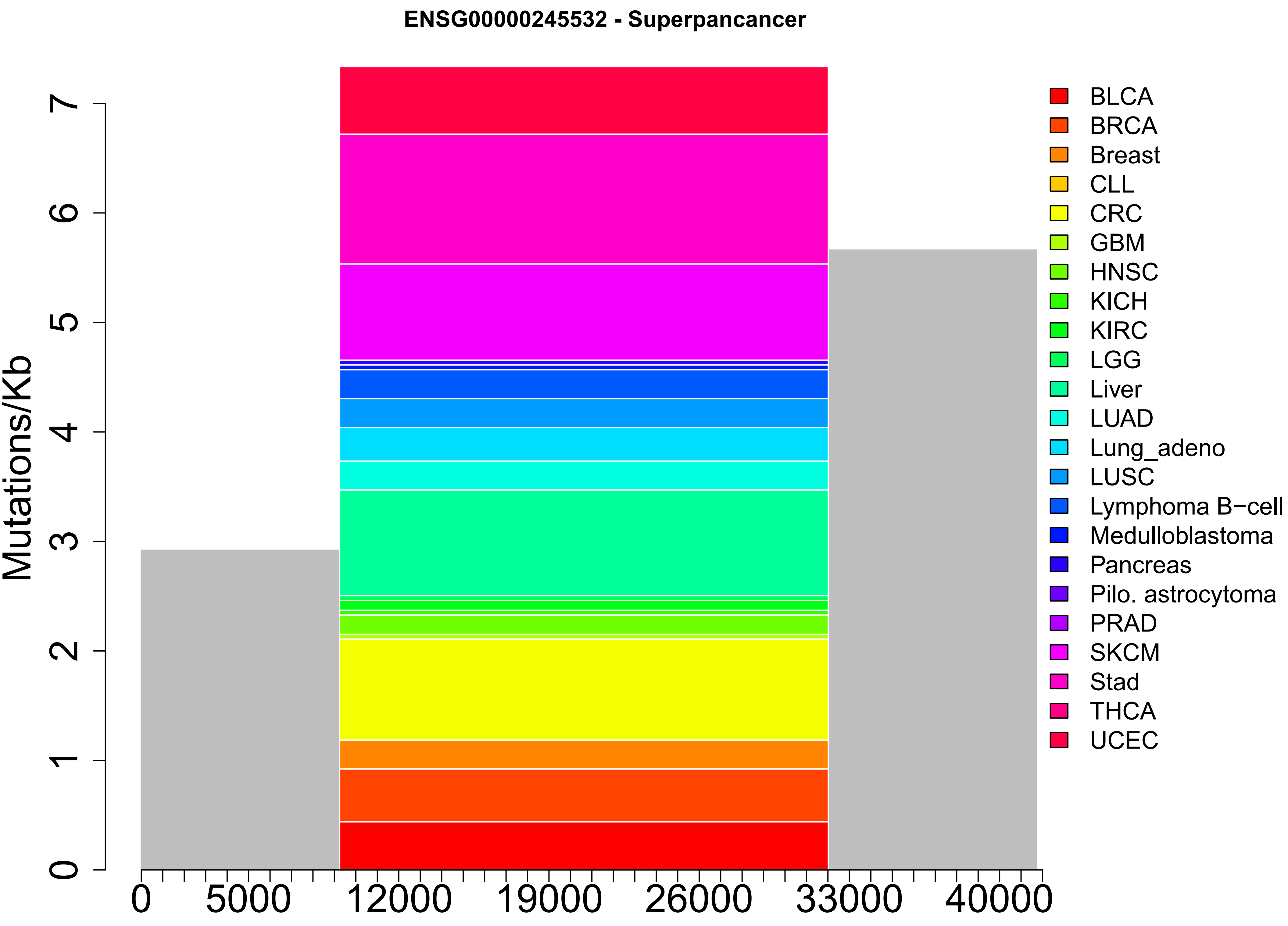

ENSG00000245532 - Superpancancer
Mutations/Kb
0
1
2
3
4
5
6
7
0
5000
12000
19000
26000
33000
40000
BLCA
BRCA
Breast
CLL
CRC
GBM
HNSC
KICH
KIRC
LGG
Liver
LUAD
Lung_adeno
LUSC
Lymphoma B−cell
Medulloblastoma
Pancreas
Pilo. astrocytoma
PRAD
SKCM
Stad
THCA
UCEC

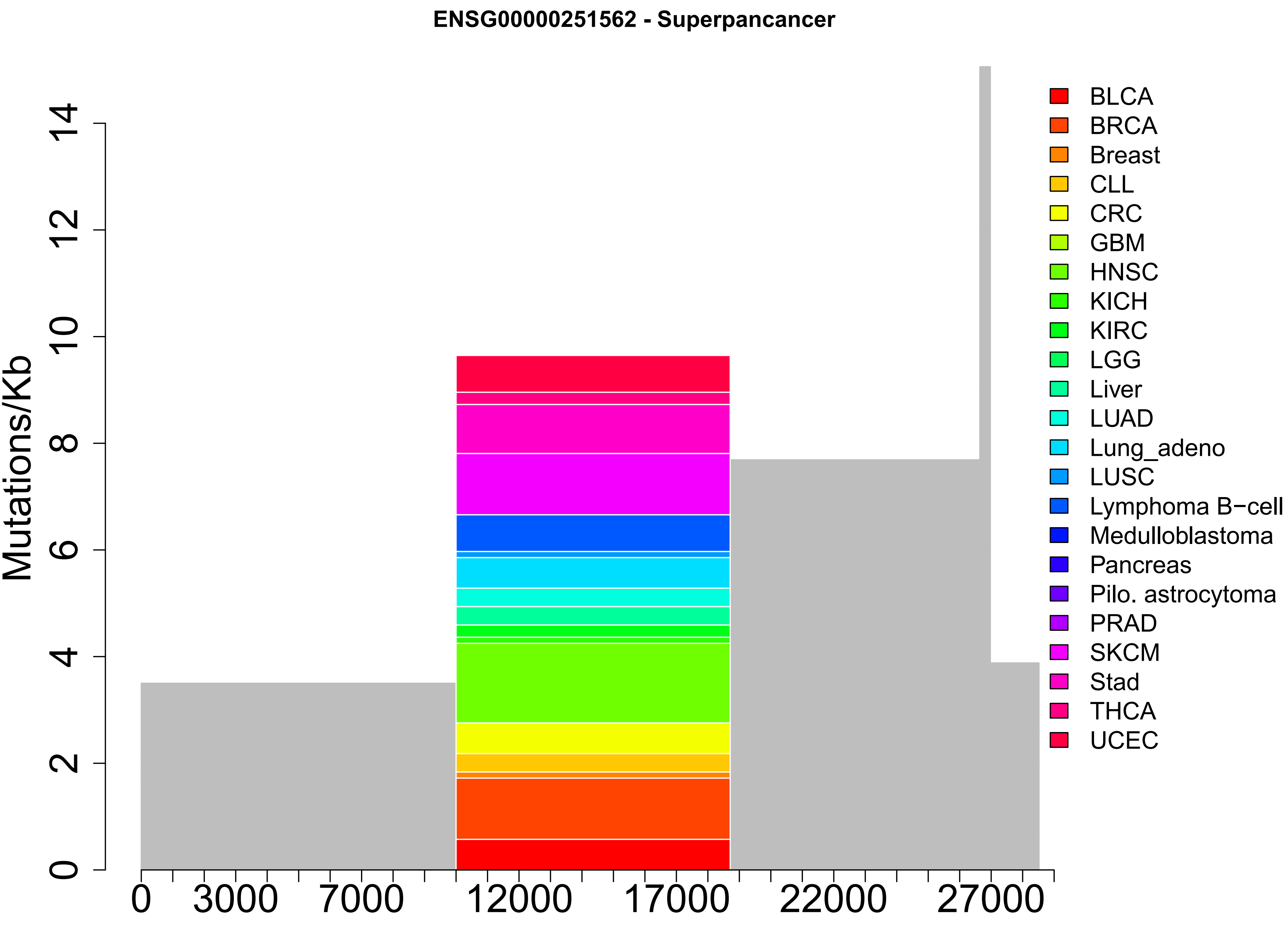

ENSG00000251562 - Superpancancer
Mutations/Kb
0
2
4
6
8
10
12
14
0
3000
7000
12000
17000
22000
27000
BLCA
BRCA
Breast
CLL
CRC
GBM
HNSC
KICH
KIRC
LGG
Liver
LUAD
Lung_adeno
LUSC
Lymphoma B−cell
Medulloblastoma
Pancreas
Pilo. astrocytoma
PRAD
SKCM
Stad
THCA
UCEC

Supplementary Figure S8

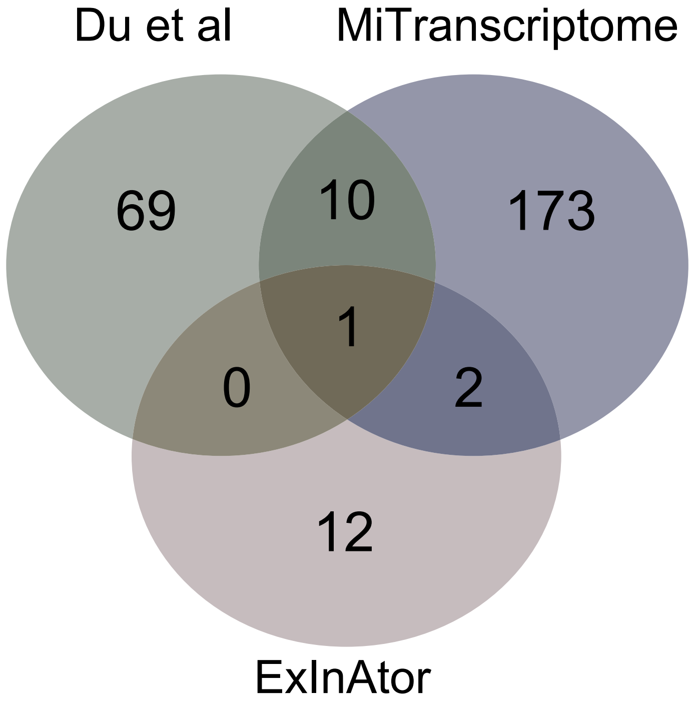

Supplementary Figure S9

A

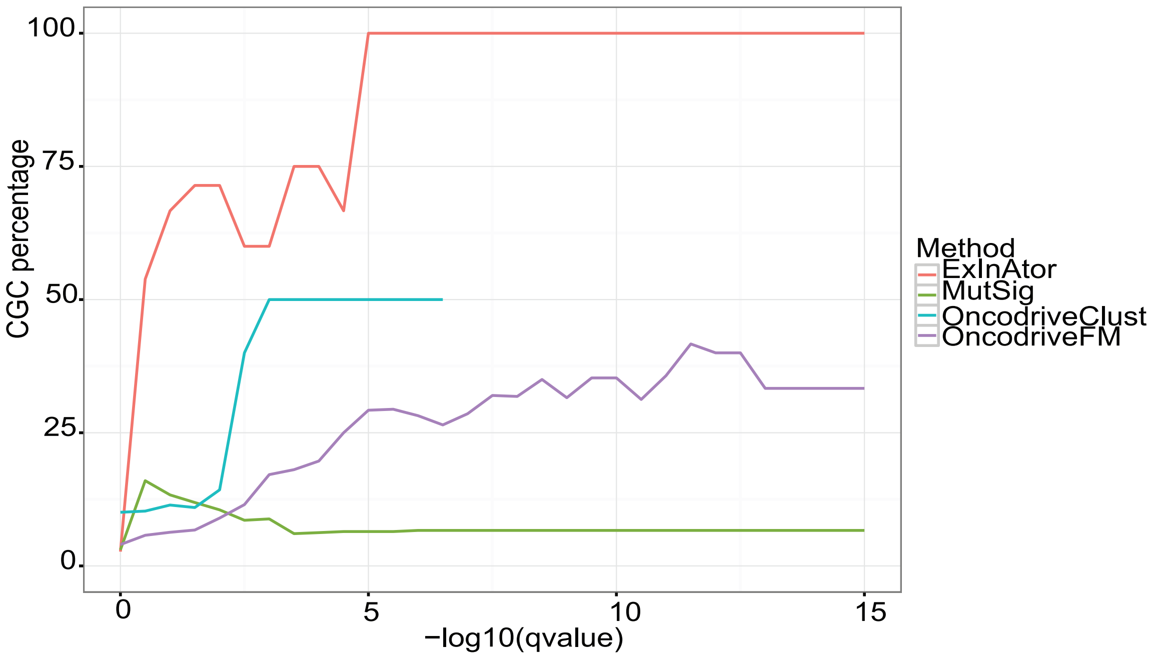

B

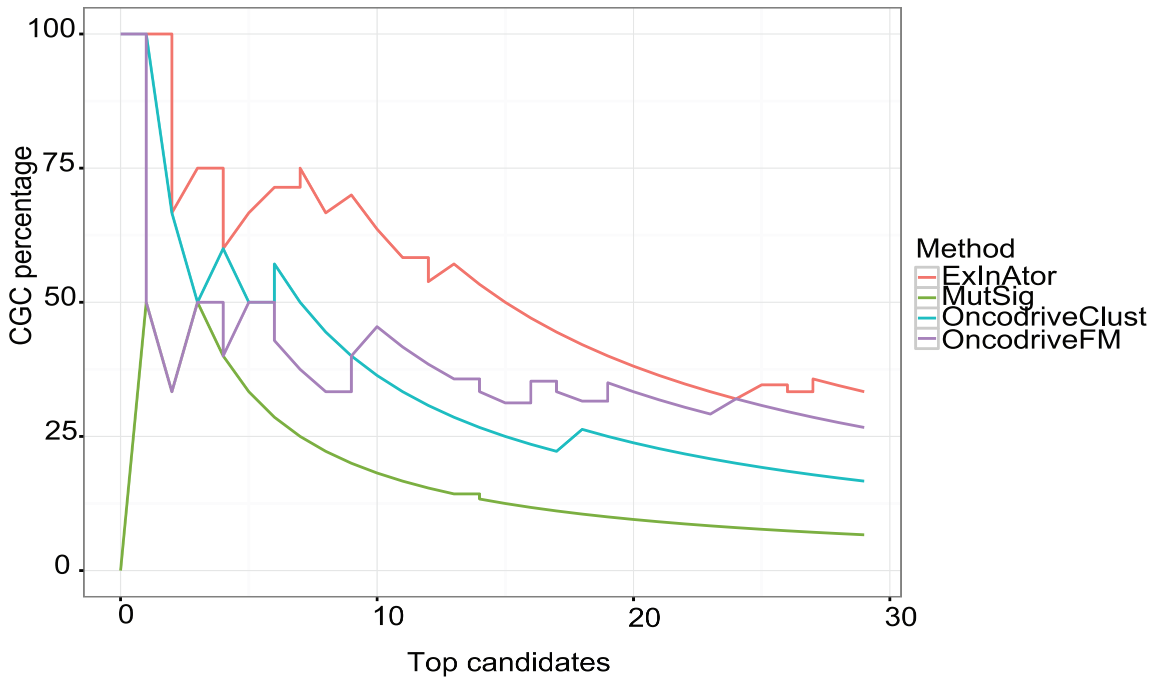

Supplementary Figure S10

A

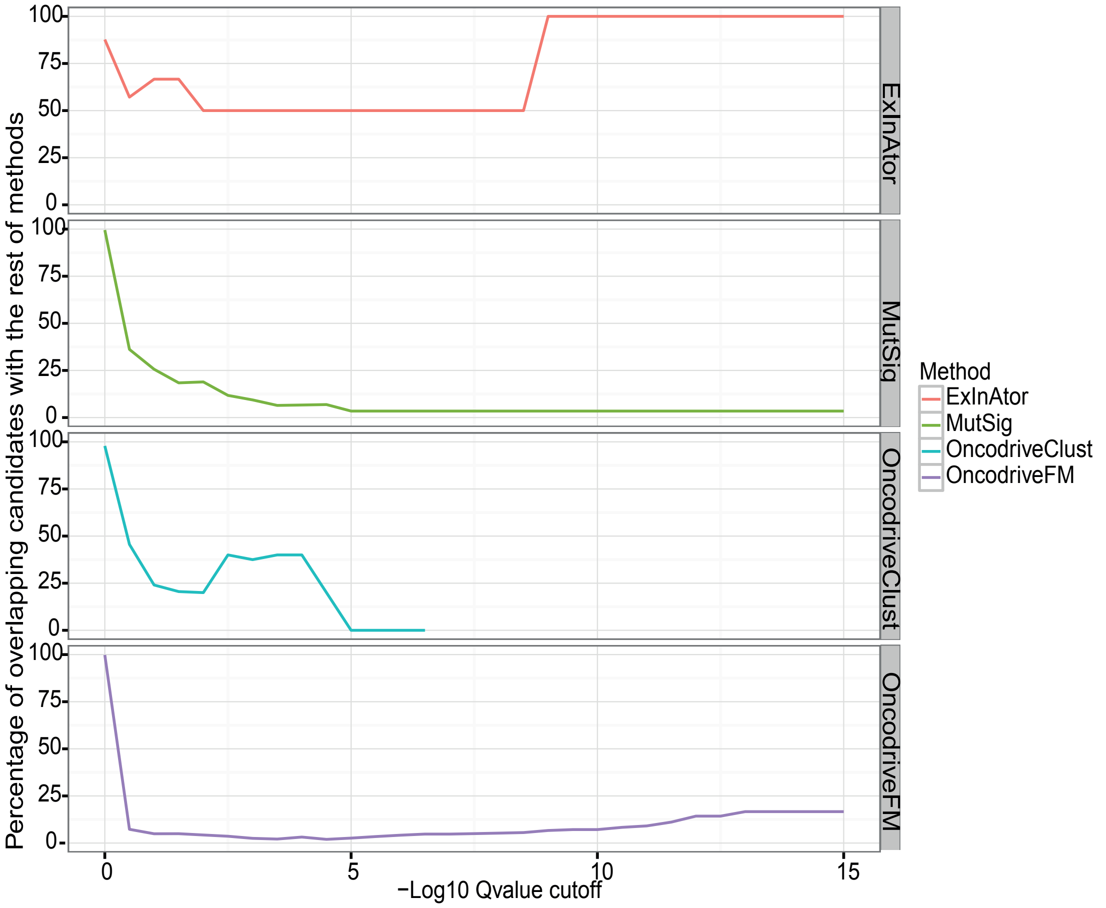

B

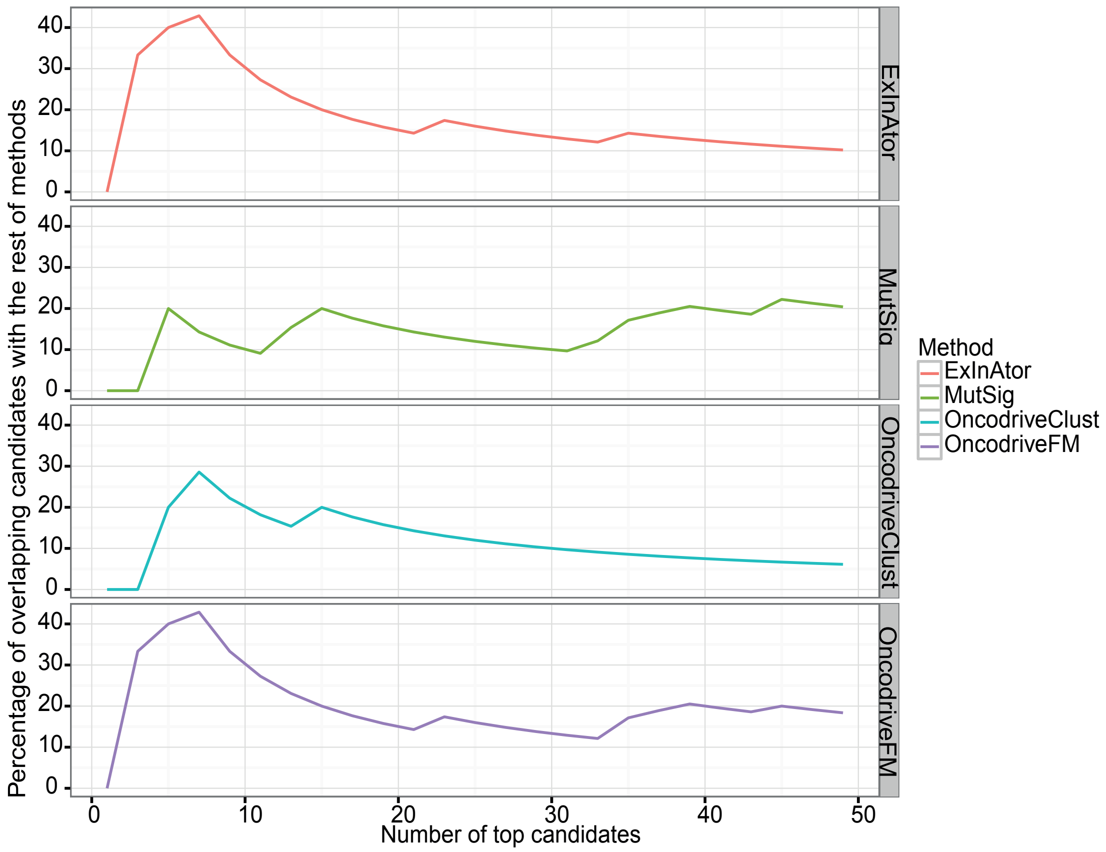

Supplementary Figure S11

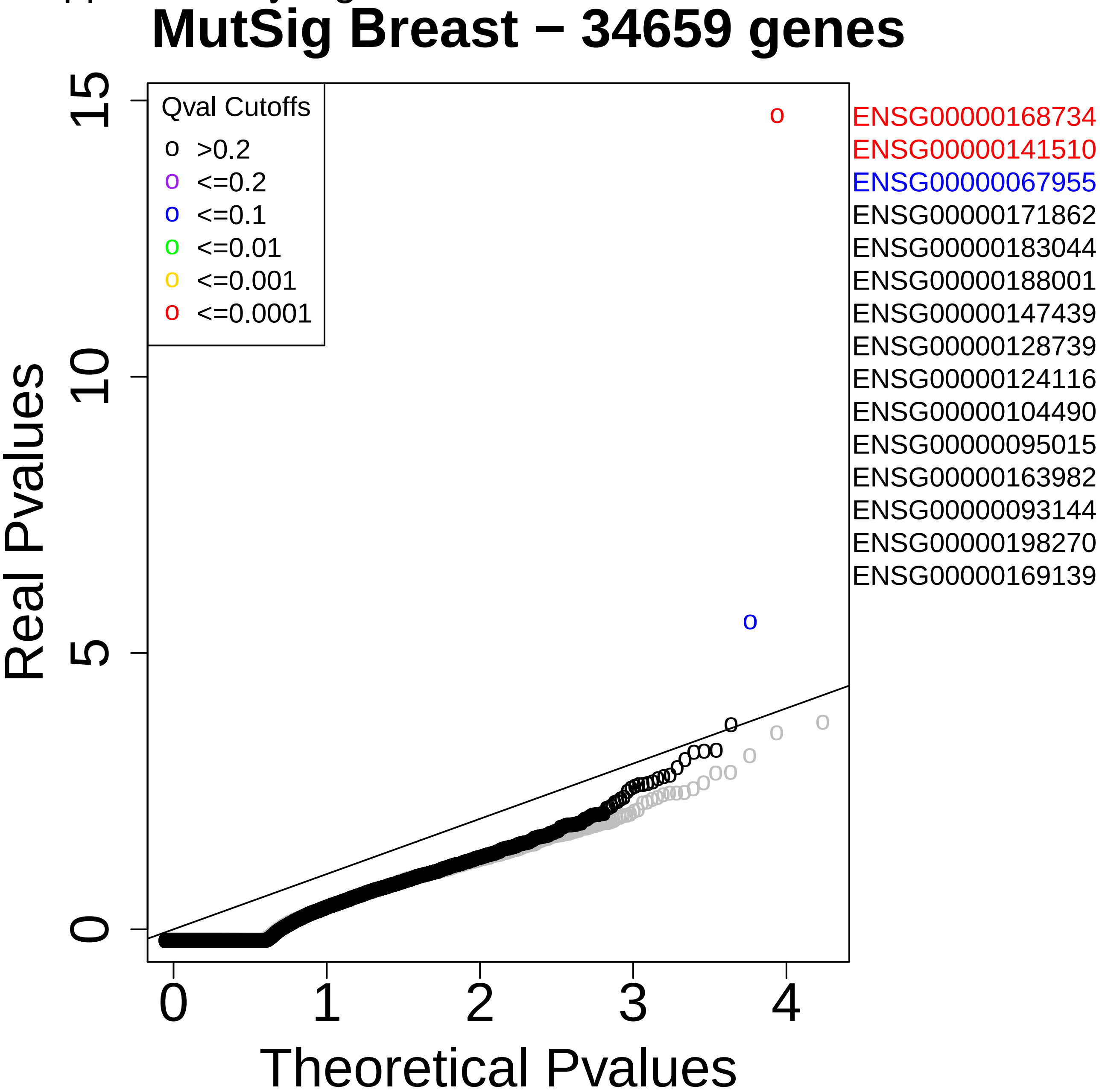

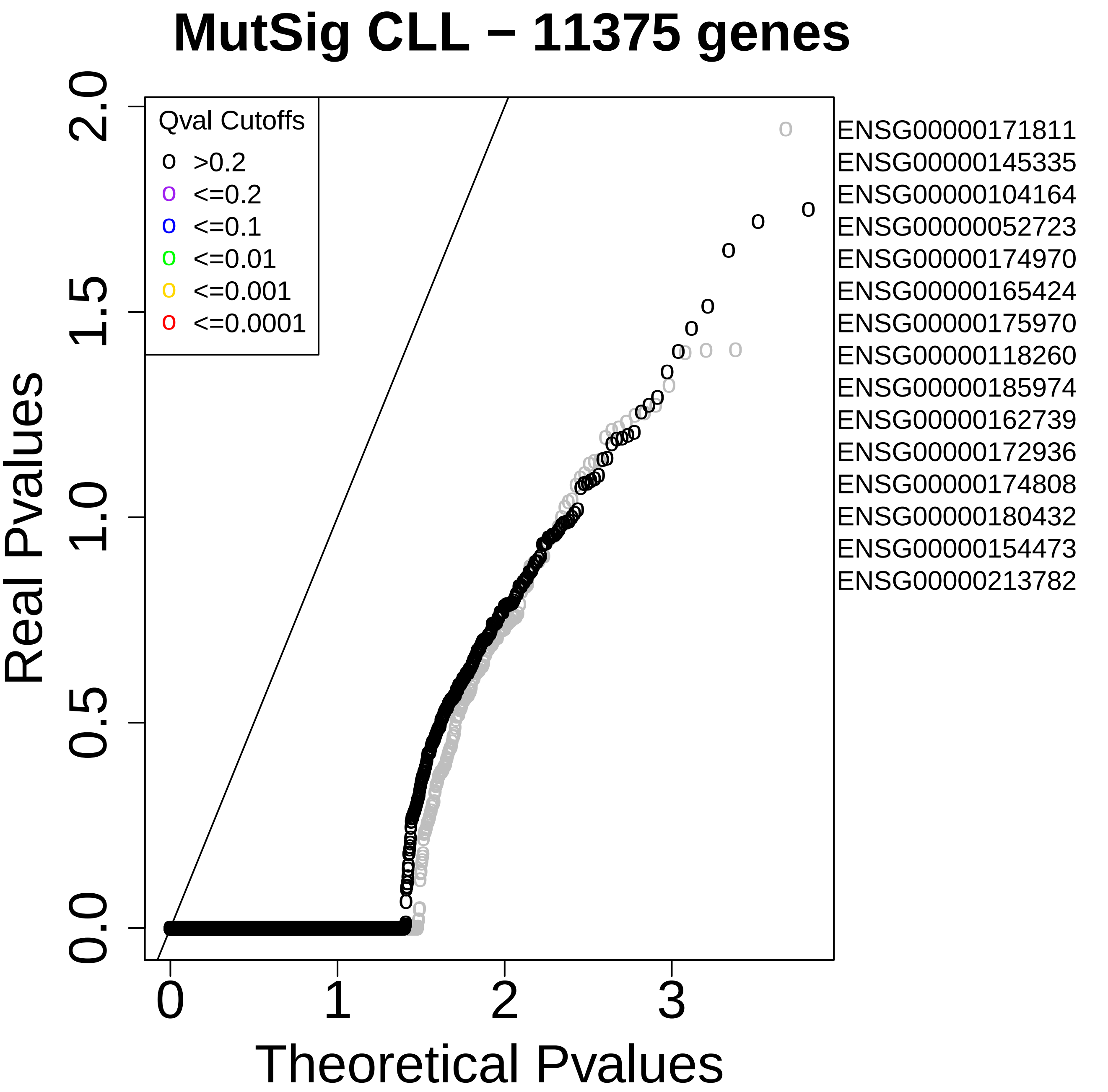
MutSig CLL – 11375 genes
Qval Cutoffs
o >0.2
o <=0.2
o <=0.1
o <=0.01
o <=0.001
o <=0.0001
Real Pvalues
Theoretical Pvalues
0.0
0.5
1.0
1.5
2.0
0
1
2
3
ENSG00000171811
ENSG00000145335
ENSG00000104164
ENSG00000052723
ENSG00000174970
ENSG00000165424
ENSG00000175970
ENSG00000118260
ENSG00000185974
ENSG00000162739
ENSG00000172936
ENSG00000174808
ENSG00000180432
ENSG00000154473
ENSG00000213782

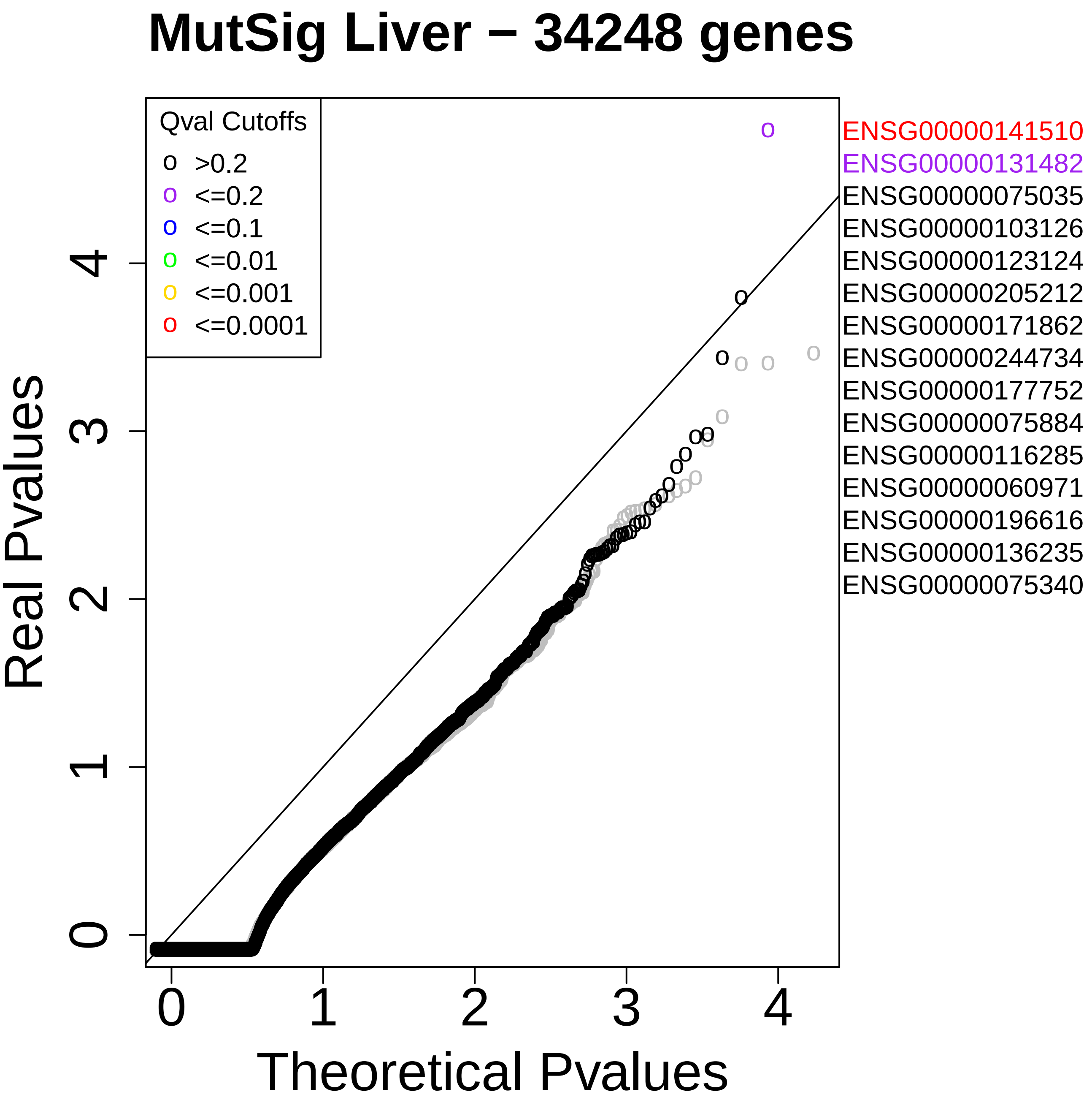
MutSig Liver − 34248 genes
Qval Cutoffs
o >0.2
o <=0.2
o <=0.1
o <=0.01
o <=0.001
o <=0.0001
ENSG00000141510
ENSG00000131482
ENSG00000075035
ENSG00000103126
ENSG00000123124
ENSG00000205212
ENSG00000171862
ENSG00000244734
ENSG00000177752
ENSG00000075884
ENSG00000116285
ENSG00000060971
ENSG00000196616
ENSG00000136235
ENSG00000075340
Real Pvalues
Theoretical Pvalues
0
1
2
3
4

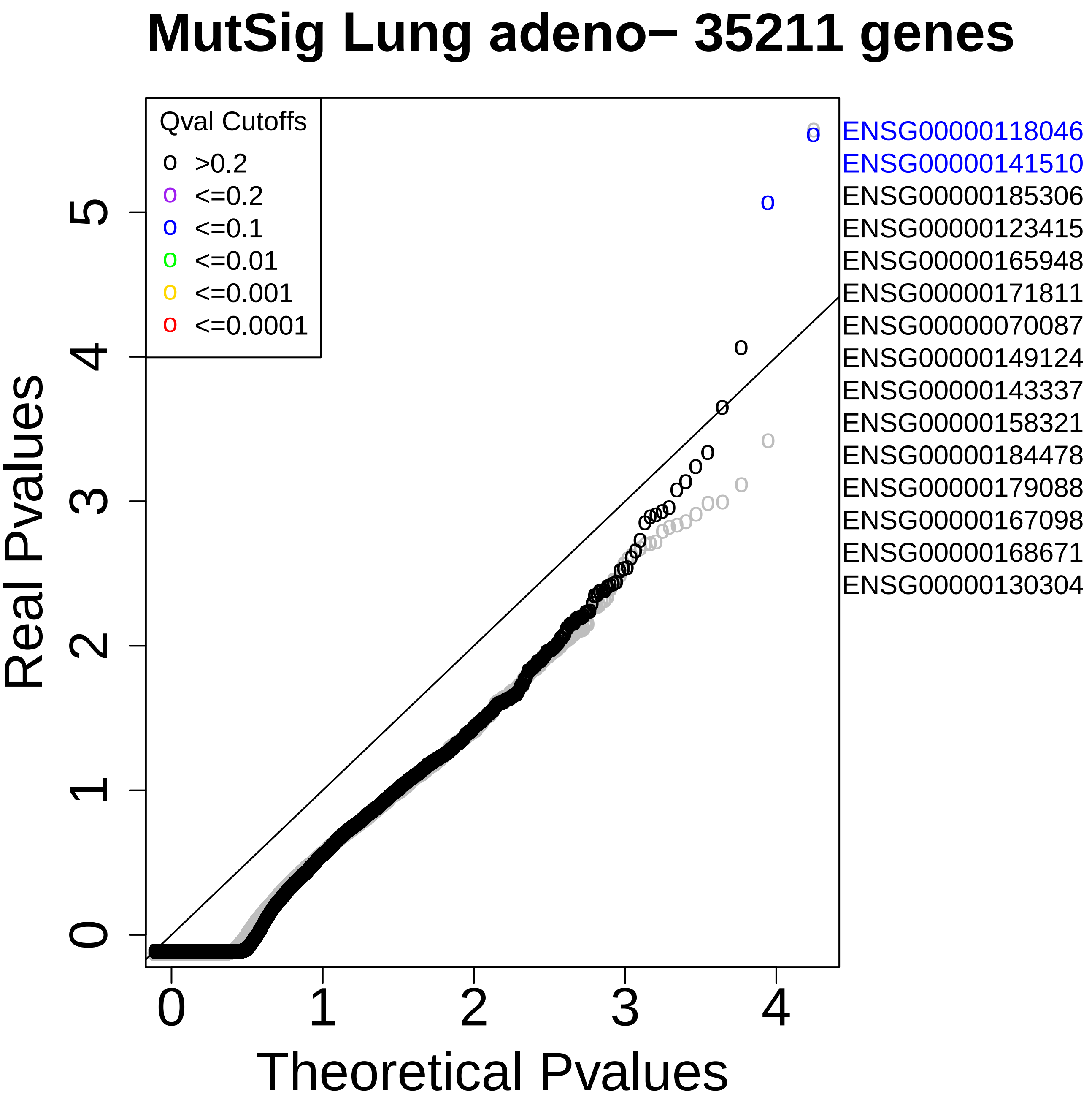

MutSig Lung adeno– 35211 genes
Qval Cutoffs
o >0.2
o <=0.2
o <=0.1
o <=0.01
o <=0.001
o <=0.0001
Real Pvalues
0
1
2
3
4
5
Theoretical Pvalues
0
1
2
3
4
ENSG00000118046
ENSG00000141510
ENSG00000185306
ENSG00000123415
ENSG00000165948
ENSG00000171811
ENSG00000070087
ENSG00000149124
ENSG00000143337
ENSG00000158321
ENSG00000184478
ENSG00000179088
ENSG00000167098
ENSG00000168671
ENSG00000130304

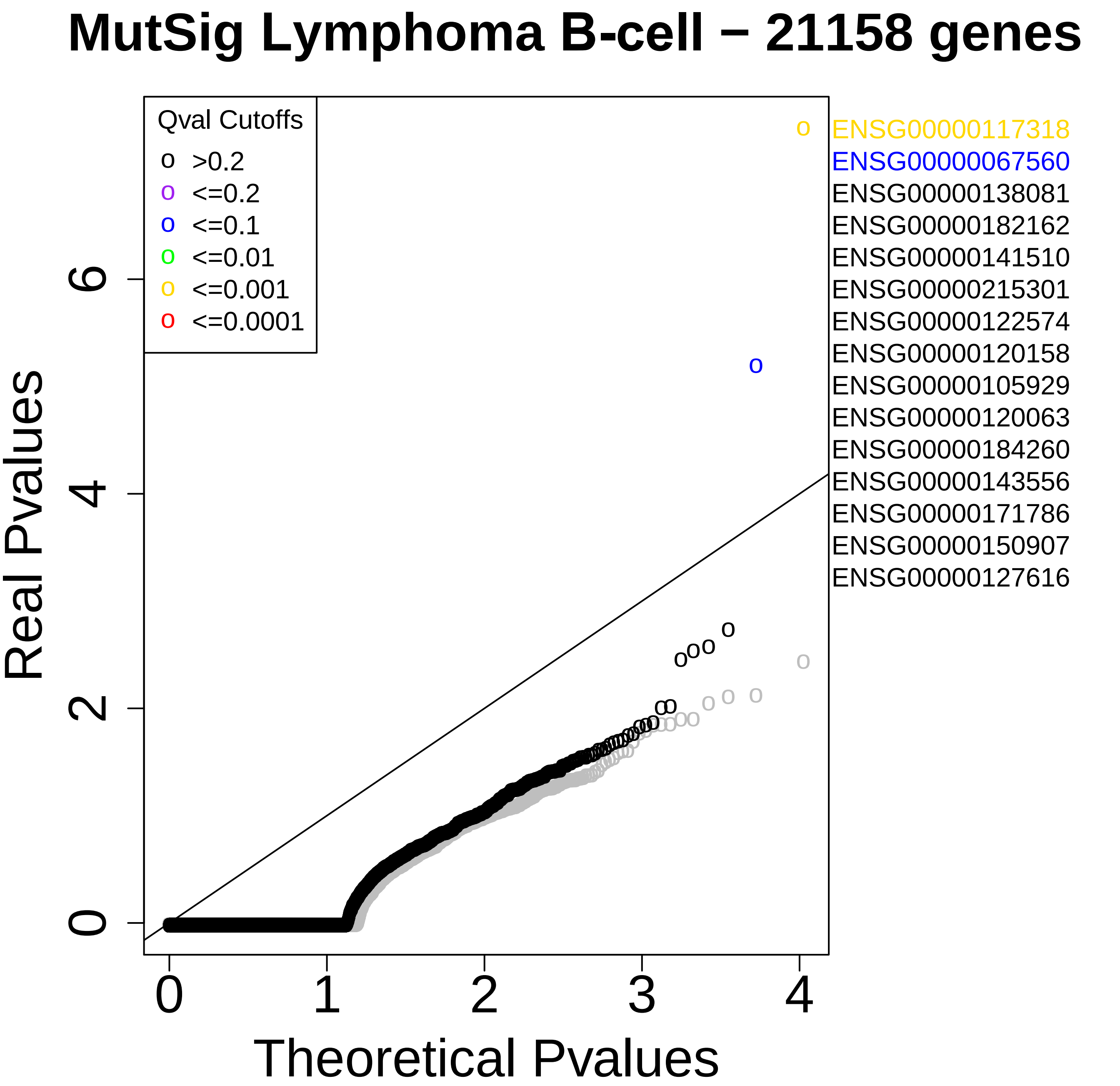
MutSig Lymphoma B-cell − 21158 genes
Qval Cutoffs
>0.2
<=0.2
<=0.1
<=0.01
<=0.001
<=0.0001
ENSG00000117318
ENSG00000067560
ENSG00000138081
ENSG00000182162
ENSG00000141510
ENSG00000215301
ENSG00000122574
ENSG00000120158
ENSG00000105929
ENSG00000120063
ENSG00000184260
ENSG00000143556
ENSG00000171786
ENSG00000150907
ENSG00000127616
Real Pvalues
Theoretical Pvalues
0
2
4
6
0
1
2
3
4

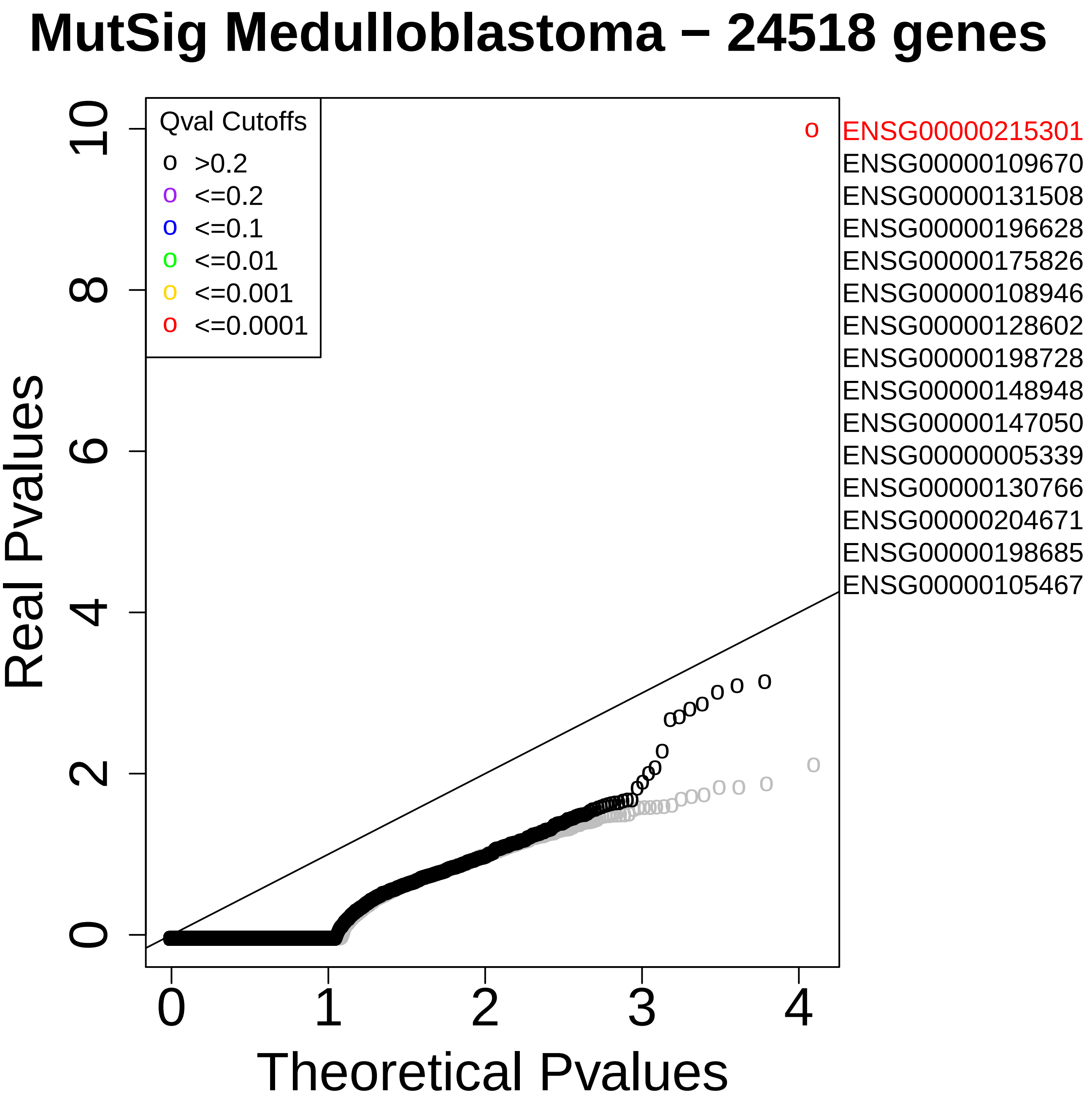
MutSig Medulloblastoma − 24518 genes
Qval Cutoffs
o >0.2
o <=0.2
o <=0.1
o <=0.01
o <=0.001
o <=0.0001
Real Pvalues
Theoretical Pvalues
0
2
4
6
8
10
0
1
2
3
4
ENSG00000215301
ENSG00000109670
ENSG00000131508
ENSG00000196628
ENSG00000175826
ENSG00000108946
ENSG00000128602
ENSG00000198728
ENSG00000148948
ENSG00000147050
ENSG00000005339
ENSG00000130766
ENSG00000204671
ENSG00000198685
ENSG00000105467

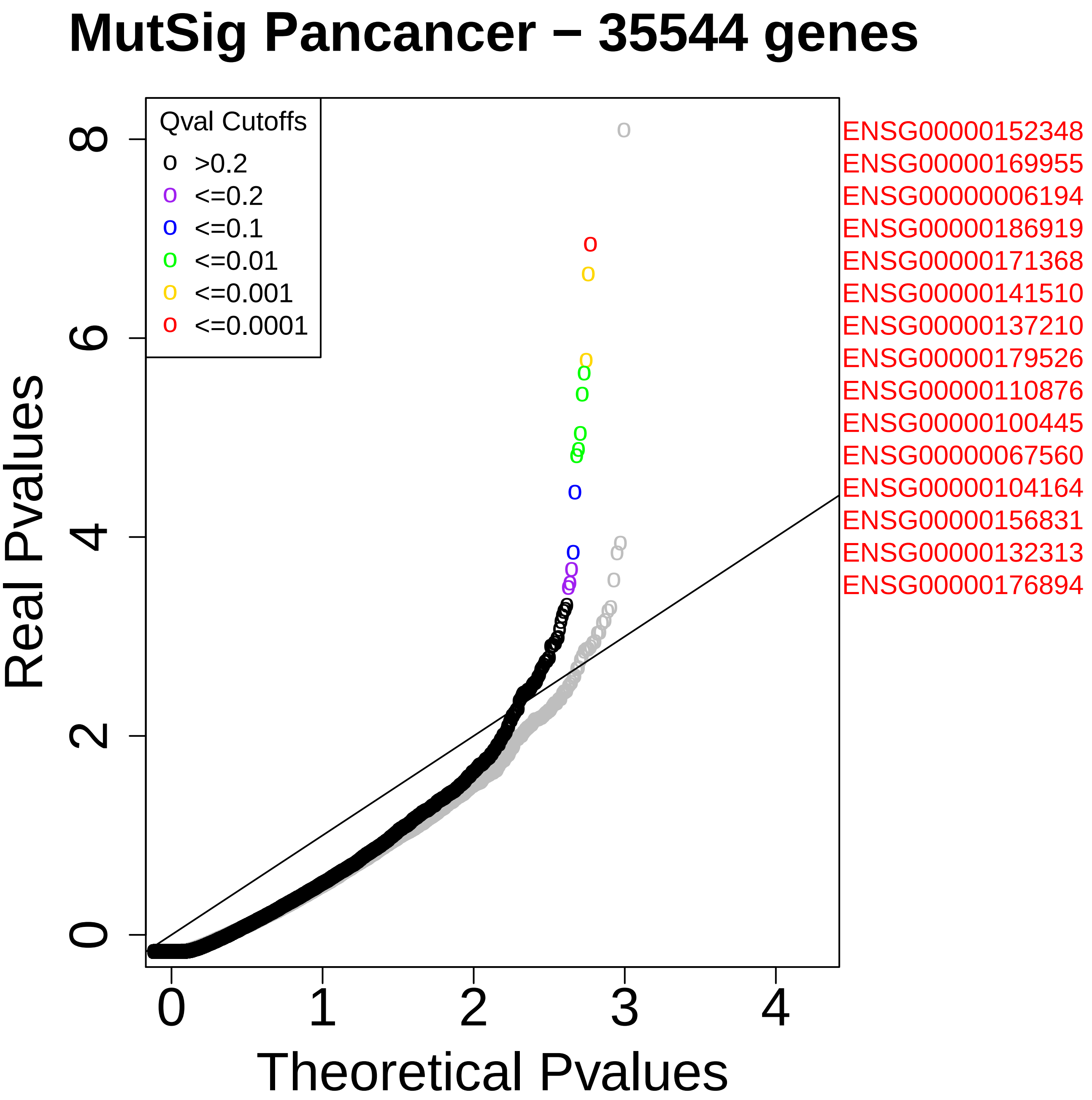
MutSig Pancancer – 35544 genes
Qval Cutoffs
o >0.2
o <=0.2
o <=0.1
o <=0.01
o <=0.001
o <=0.0001
Real Pvalues
Theoretical Pvalues
0
2
4
6
8
0
1
2
3
4
ENSG00000152348
ENSG00000169955
ENSG00000006194
ENSG00000186919
ENSG00000171368
ENSG00000141510
ENSG00000137210
ENSG00000179526
ENSG00000110876
ENSG00000100445
ENSG00000067560
ENSG00000104164
ENSG00000156831
ENSG00000132313
ENSG00000176894

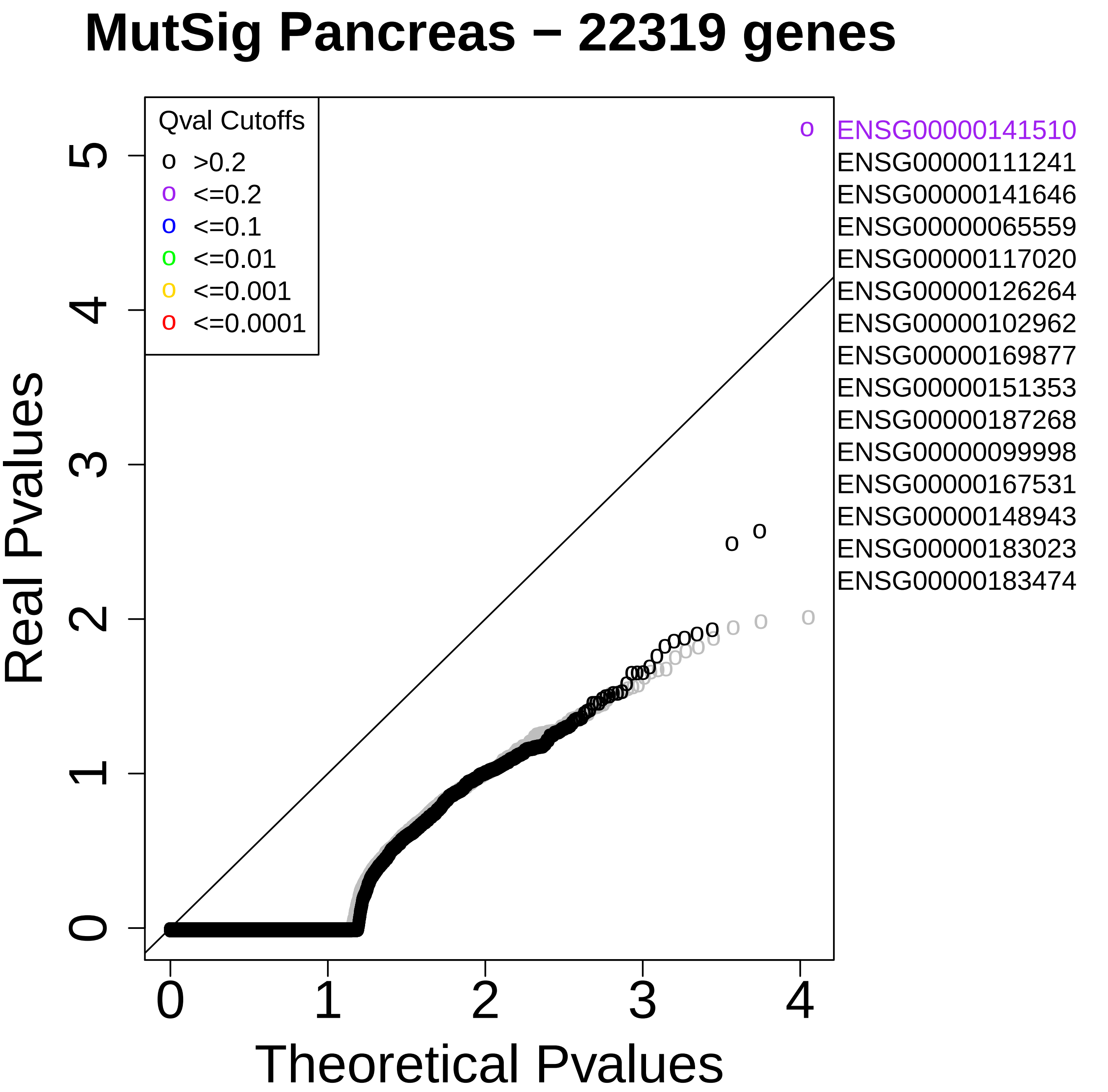

MutSig Pancreas − 22319 genes
Qval Cutoffs
o >0.2
o <=0.2
o <=0.1
o <=0.01
o <=0.001
o <=0.0001
Real Pvalues
Theoretical Pvalues
0
1
2
3
4
5
ENSG00000141510
ENSG00000111241
ENSG00000141646
ENSG00000065559
ENSG00000117020
ENSG00000126264
ENSG00000102962
ENSG00000169877
ENSG00000151353
ENSG00000187268
ENSG00000099998
ENSG00000167531
ENSG00000148943
ENSG00000183023
ENSG00000183474

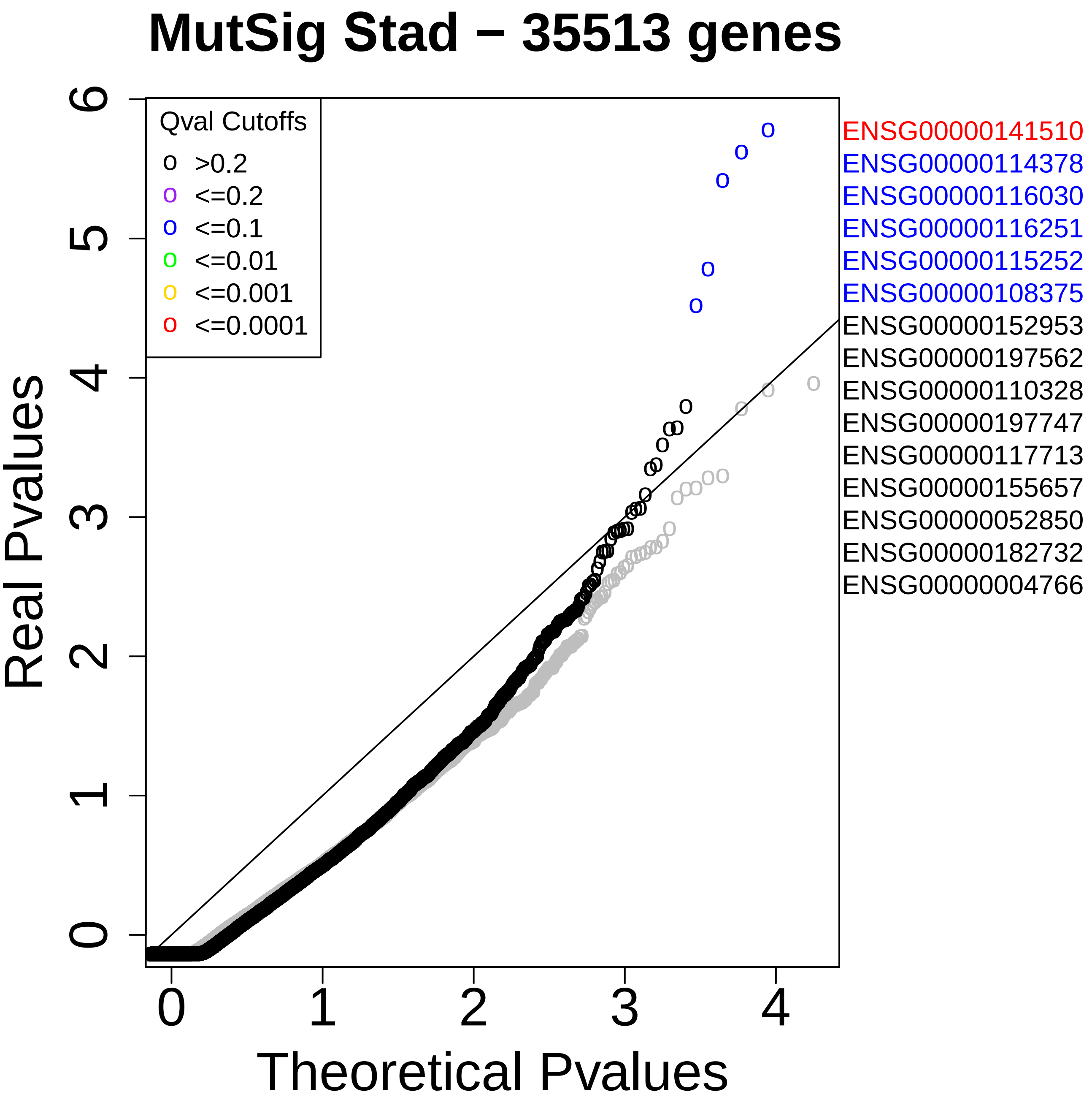
MutSig Stad − 35513 genes
Qval Cutoffs
o >0.2
o <=0.2
o <=0.1
o <=0.01
o <=0.001
o <=0.0001
ENSG00000141510
ENSG00000114378
ENSG00000116030
ENSG00000116251
ENSG00000115252
ENSG00000108375
ENSG00000152953
ENSG00000197562
ENSG00000110328
ENSG00000197747
ENSG00000117713
ENSG00000155657
ENSG00000052850
ENSG00000182732
ENSG00000004766
Real Pvalues
Theoretical Pvalues
0
1
2
3
4
5
6

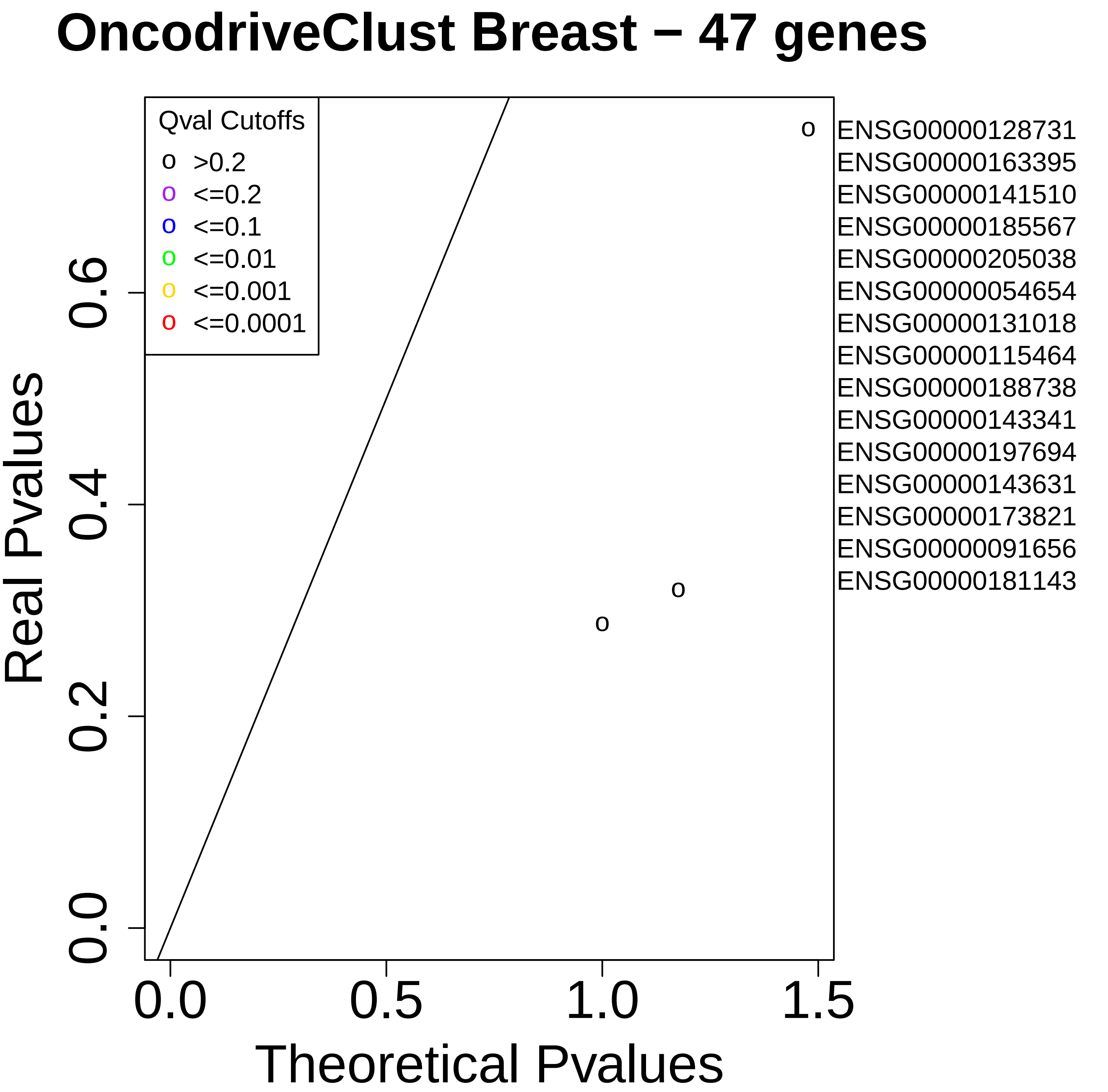

OncodriveClust Breast – 47 genes
Qval Cutoffs
o >0.2
o <=0.2
o <=0.1
o <=0.01
o <=0.001
o <=0.0001
ENSG00000128731
ENSG00000163395
ENSG00000141510
ENSG00000185567
ENSG00000205038
ENSG00000054654
ENSG00000131018
ENSG00000115464
ENSG00000188738
ENSG00000143341
ENSG00000197694
ENSG00000143631
ENSG00000173821
ENSG00000091656
ENSG00000181143
0.0
0.2
0.4
0.6
Real Pvalues
0.0
0.5
1.0
1.5
Theoretical Pvalues

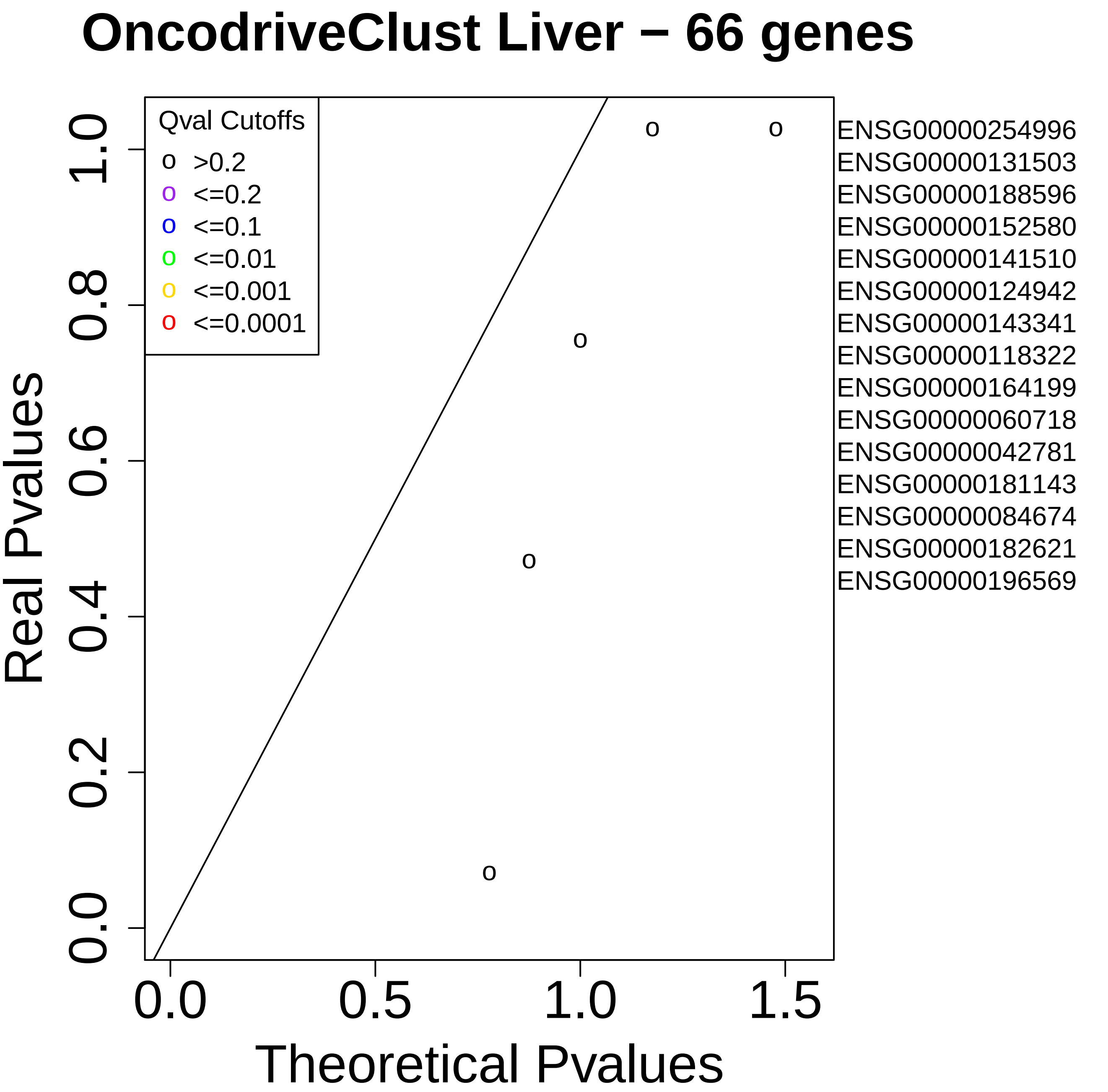

OncodriveClust Liver − 66 genes
Qval Cutoffs
o >0.2
o <=0.2
o <=0.1
o <=0.01
o <=0.001
o <=0.0001
Real Pvalues
0.0
0.2
0.4
0.6
0.8
1.0
Theoretical Pvalues
0.0
0.5
1.0
1.5
ENSG00000254996
ENSG00000131503
ENSG00000188596
ENSG00000152580
ENSG00000141510
ENSG00000124942
ENSG00000143341
ENSG00000118322
ENSG00000164199
ENSG00000060718
ENSG00000042781
ENSG00000181143
ENSG00000084674
ENSG00000182621
ENSG00000196569

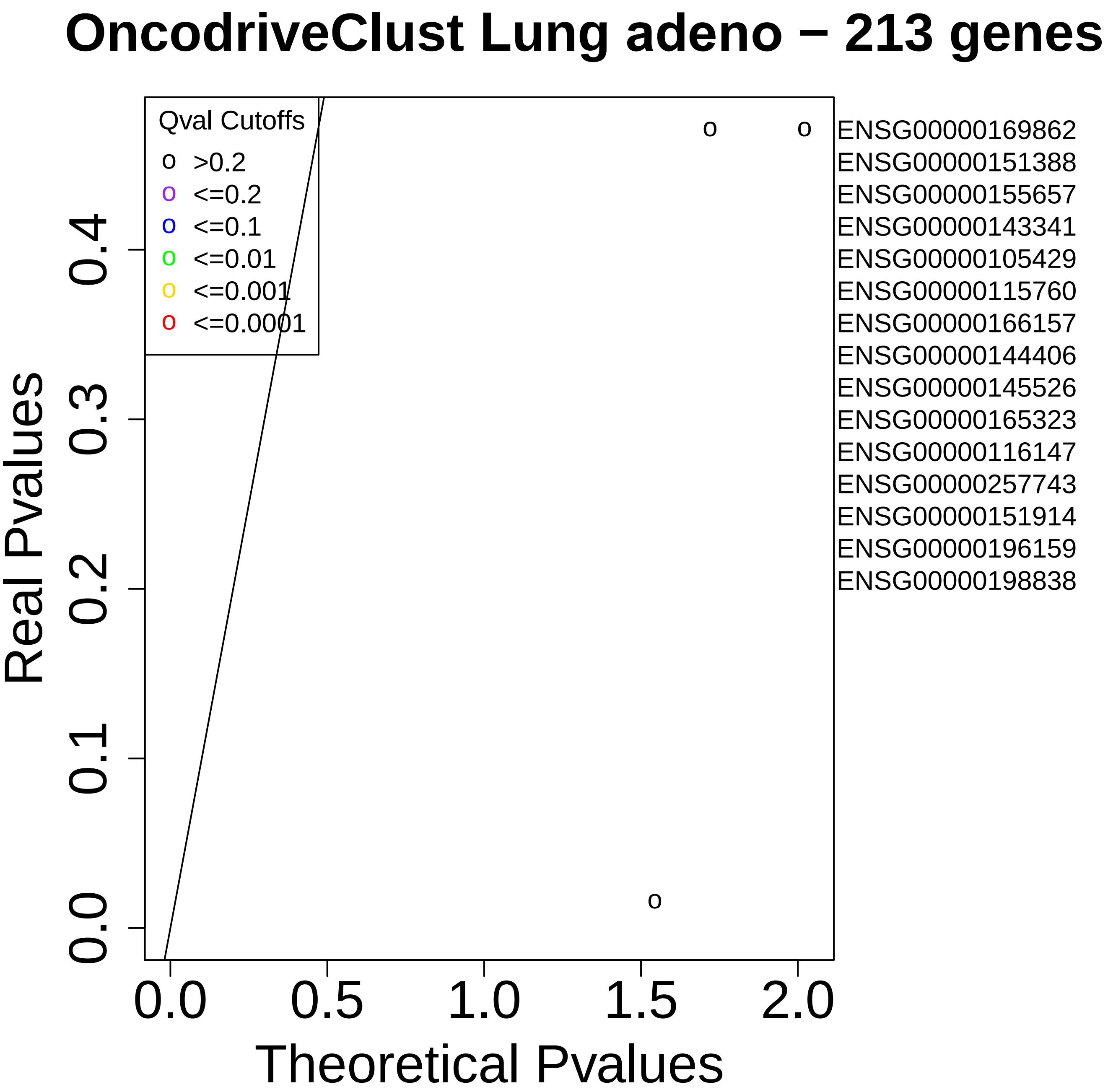
OncodriveClust Lung adeno − 213 genes
Qval Cutoffs
o >0.2
o <=0.2
o <=0.1
o <=0.01
o <=0.001
o <=0.0001
Real Pvalues
0.0
0.1
0.2
0.3
0.4
Theoretical Pvalues
0.0
0.5
1.0
1.5
2.0
ENSG00000169862
ENSG00000151388
ENSG00000155657
ENSG00000143341
ENSG00000105429
ENSG00000115760
ENSG00000166157
ENSG00000144406
ENSG00000145526
ENSG00000165323
ENSG00000116147
ENSG00000257743
ENSG00000151914
ENSG00000196159
ENSG00000198838

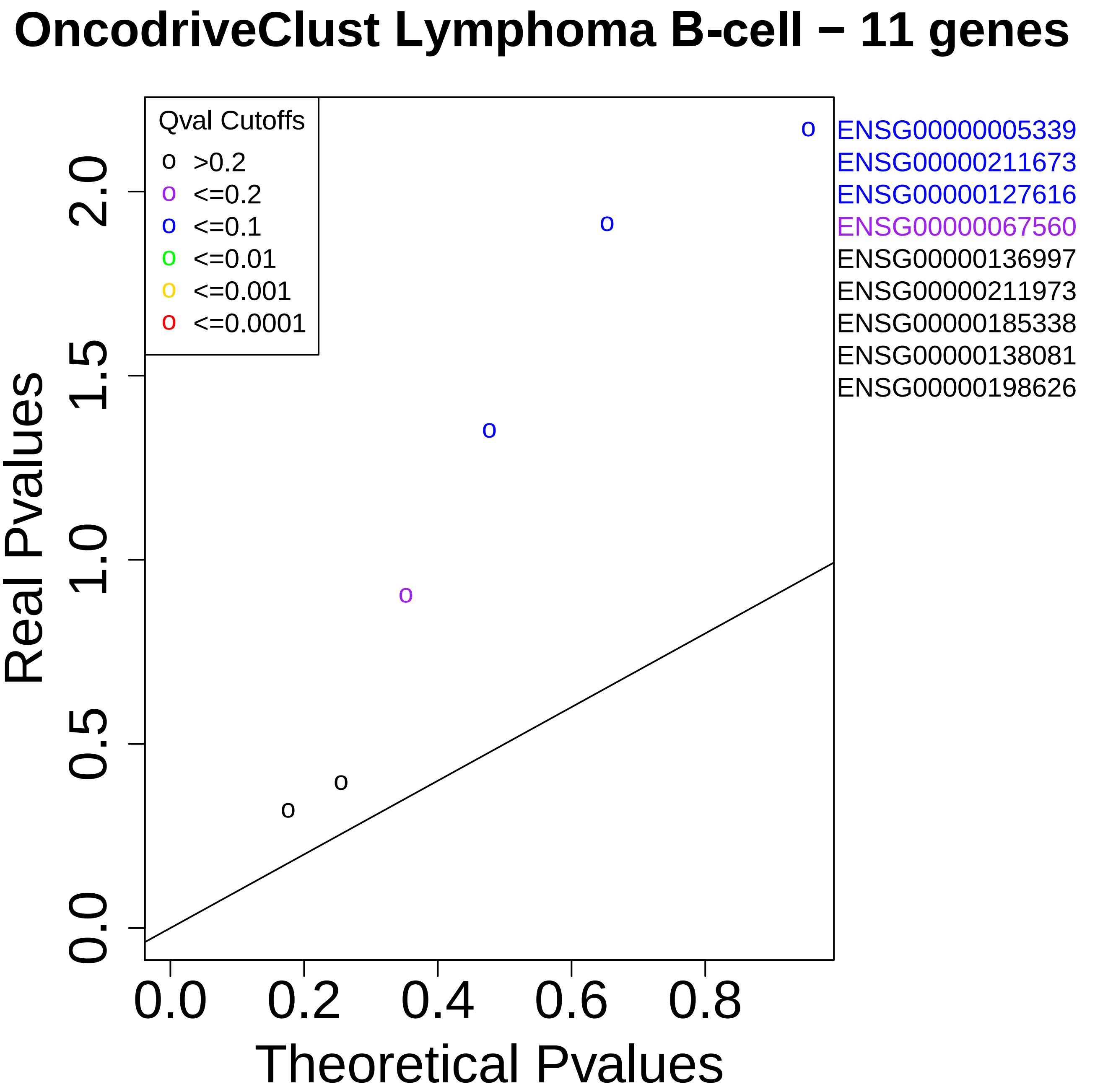
OncodriveClust Lymphoma B-cell − 11 genes
Qval Cutoffs
o >0.2
o <=0.2
o <=0.1
o <=0.01
o <=0.001
o <=0.0001
ENSG00000005339
ENSG00000211673
ENSG00000127616
ENSG00000067560
ENSG00000136997
ENSG00000211973
ENSG00000185338
ENSG00000138081
ENSG00000198626
Real Pvalues
0.0
0.5
1.0
1.5
2.0
0.0
0.2
0.4
0.6
0.8
Theoretical Pvalues

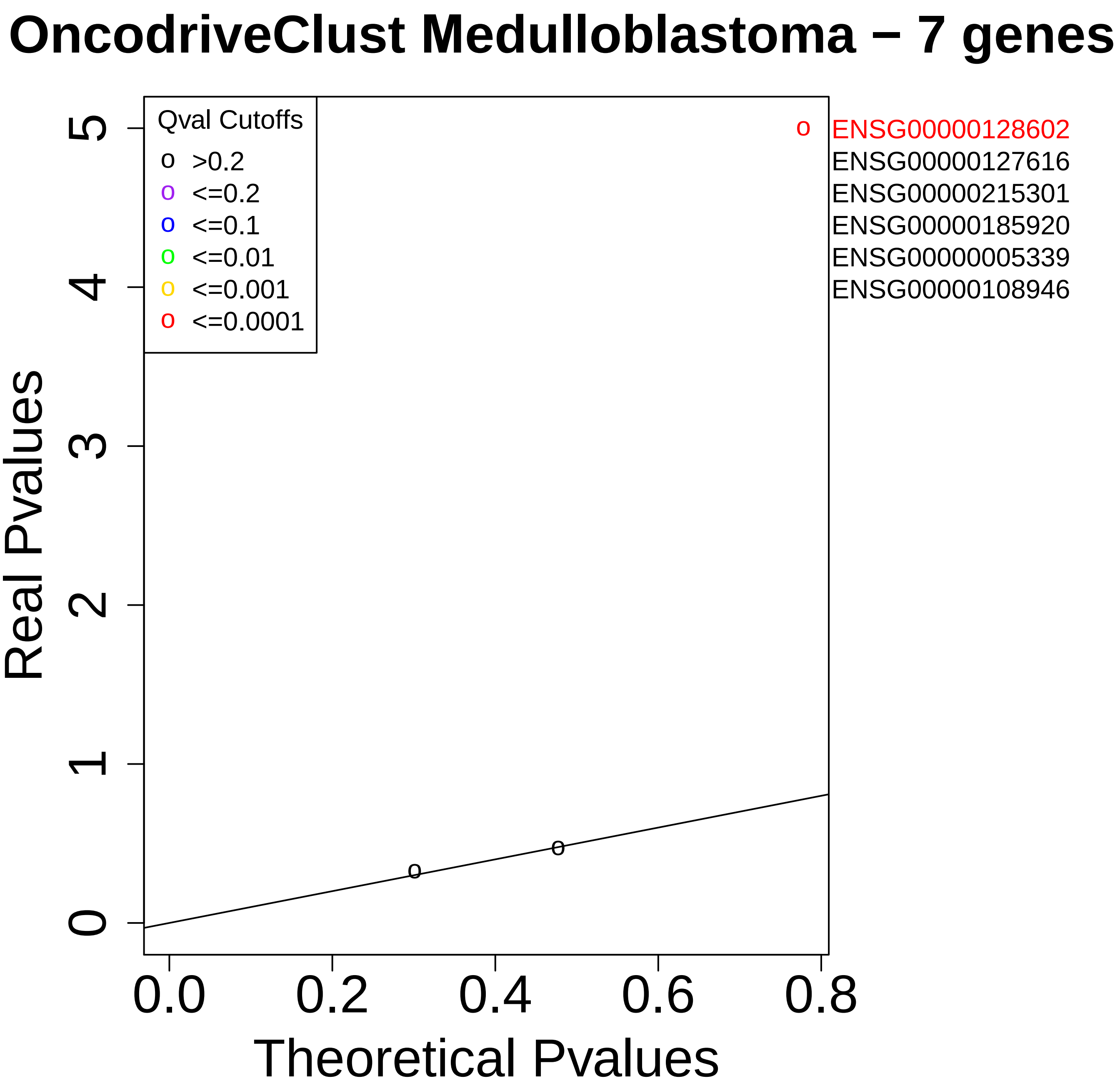
OncodriveClust Medulloblastoma − 7 genes
Qval Cutoffs
>0.2
<=0.2
<=0.1
<=0.01
<=0.001
<=0.0001
ENSG00000128602
ENSG00000127616
ENSG00000215301
ENSG00000185920
ENSG00000005339
ENSG00000108946
Real Pvalues
Theoretical Pvalues
0
1
2
3
4
5
0.0
0.2
0.4
0.6
0.8

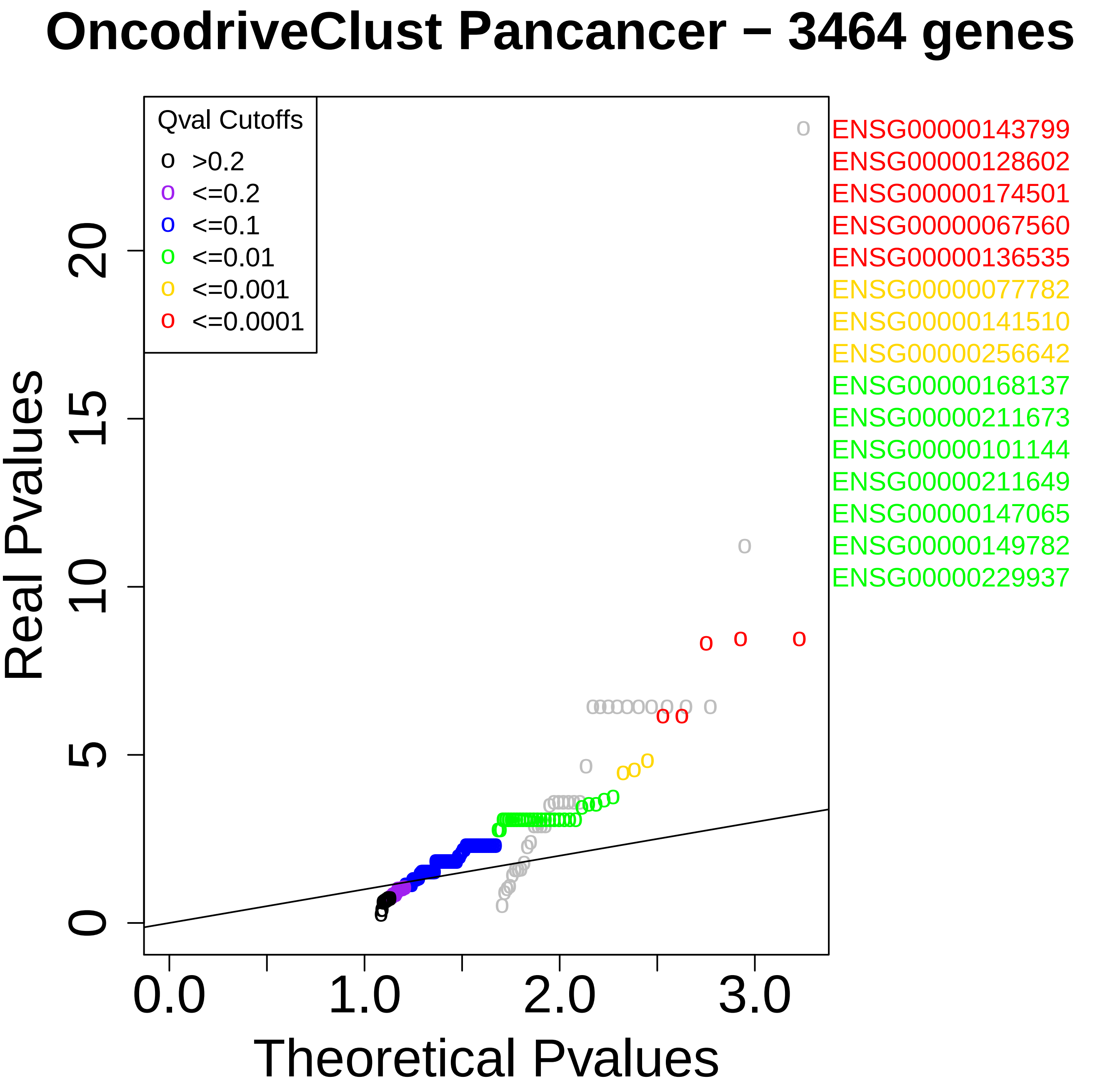
OncodriveClust Pancancer – 3464 genes
Qval Cutoffs
>0.2
<=0.2
<=0.1
<=0.01
<=0.001
<=0.0001
Real Pvalues
Theoretical Pvalues
0
5
10
15
20
0.0
1.0
2.0
3.0
ENSG00000143799
ENSG00000128602
ENSG00000174501
ENSG00000067560
ENSG00000136535
ENSG00000077782
ENSG00000141510
ENSG00000256642
ENSG00000168137
ENSG00000211673
ENSG00000101144
ENSG00000211649
ENSG00000147065
ENSG00000149782
ENSG00000229937

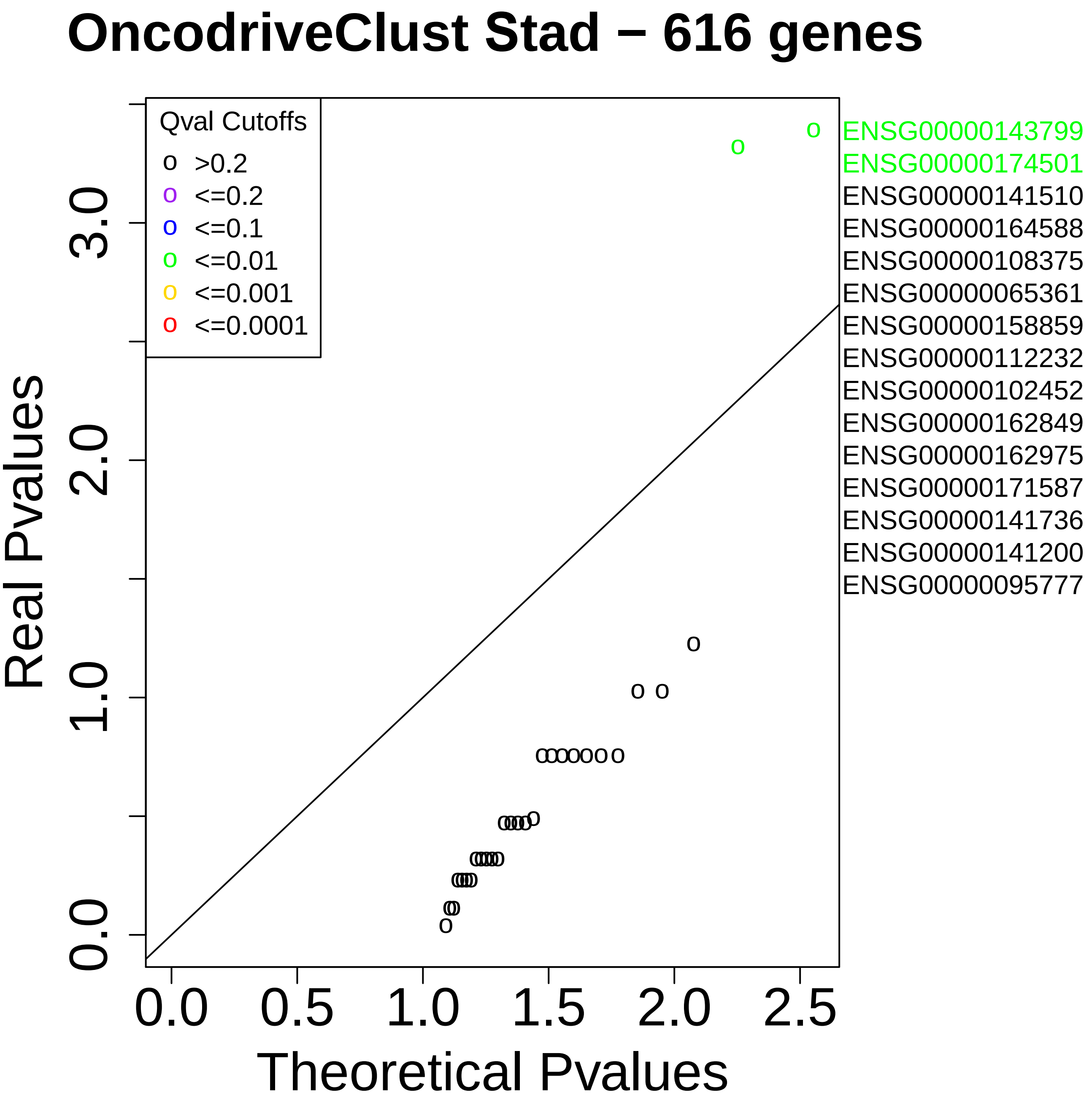
OncodriveClust Stad − 616 genes
Qval Cutoffs
>0.2
<=0.2
<=0.1
<=0.01
<=0.001
<=0.0001
ENSG00000143799
ENSG00000174501
ENSG00000141510
ENSG00000164588
ENSG00000108375
ENSG00000065361
ENSG00000158859
ENSG00000112232
ENSG00000102452
ENSG00000162849
ENSG00000162975
ENSG00000171587
ENSG00000141736
ENSG00000141200
ENSG00000095777
Real Pvalues
0.0
1.0
2.0
3.0
Theoretical Pvalues
0.0
0.5
1.0
1.5
2.0
2.5

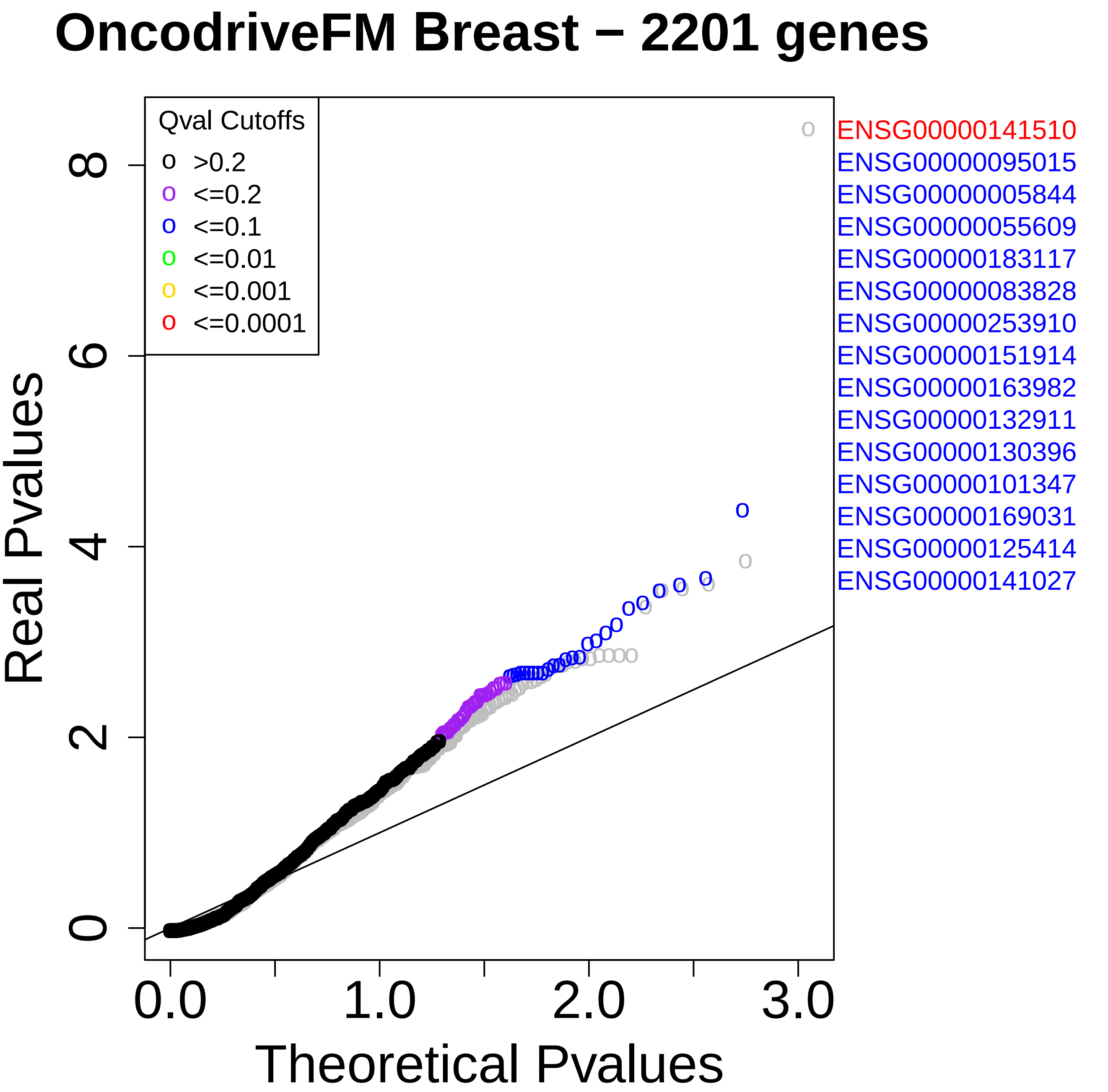

OncodriveFM Breast – 2201 genes
Qval Cutoffs
o >0.2
o <=0.2
o <=0.1
o <=0.01
o <=0.001
o <=0.0001
ENSG00000141510
ENSG00000095015
ENSG00000005844
ENSG00000055609
ENSG00000183117
ENSG00000083828
ENSG00000253910
ENSG00000151914
ENSG00000163982
ENSG00000132911
ENSG00000130396
ENSG00000101347
ENSG00000169031
ENSG00000125414
ENSG00000141027
Real Pvalues
0
2
4
6
8
0.0
1.0
2.0
3.0
Theoretical Pvalues

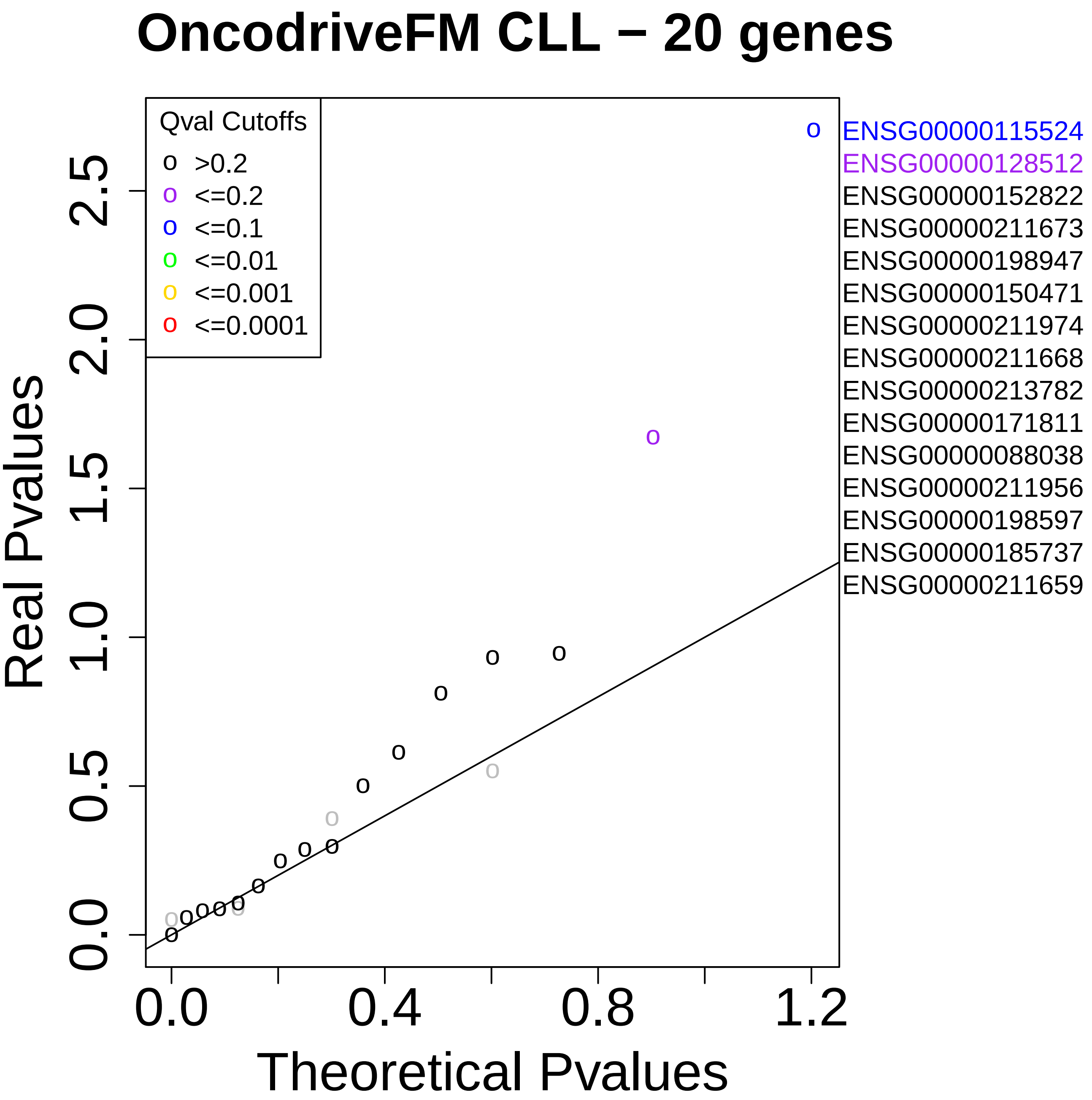

OncodriveFM CLL – 20 genes
Qval Cutoffs
>0.2
<=0.2
<=0.1
<=0.01
<=0.001
<=0.0001
Real Pvalues
0.0
0.5
1.0
1.5
2.0
2.5
Theoretical Pvalues
0.0
0.4
0.8
1.2
ENSG00000115524
ENSG00000128512
ENSG00000152822
ENSG00000211673
ENSG00000198947
ENSG00000150471
ENSG00000211974
ENSG00000211668
ENSG00000213782
ENSG00000171811
ENSG00000088038
ENSG00000211956
ENSG00000198597
ENSG00000185737
ENSG00000211659

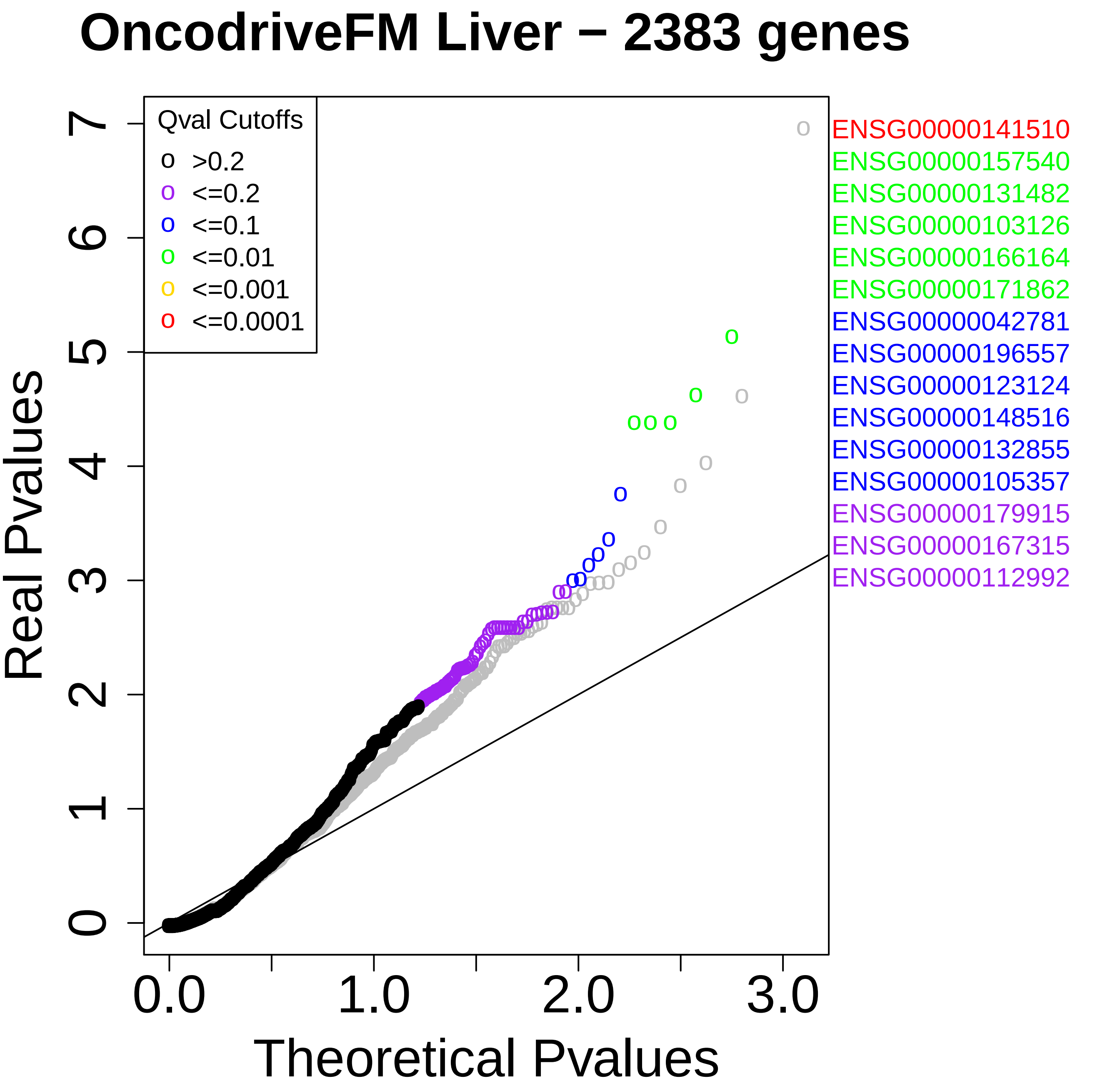
OncodriveFM Liver – 2383 genes
Qval Cutoffs
o >0.2
o <=0.2
o <=0.1
o <=0.01
o <=0.001
o <=0.0001
Real Pvalues
Theoretical Pvalues
0
1
2
3
4
5
6
7
0.0
1.0
2.0
3.0
ENSG00000141510
ENSG00000157540
ENSG00000131482
ENSG00000103126
ENSG00000166164
ENSG00000171862
ENSG00000042781
ENSG00000196557
ENSG00000123124
ENSG00000148516
ENSG00000132855
ENSG00000105357
ENSG00000179915
ENSG00000167315
ENSG00000112992

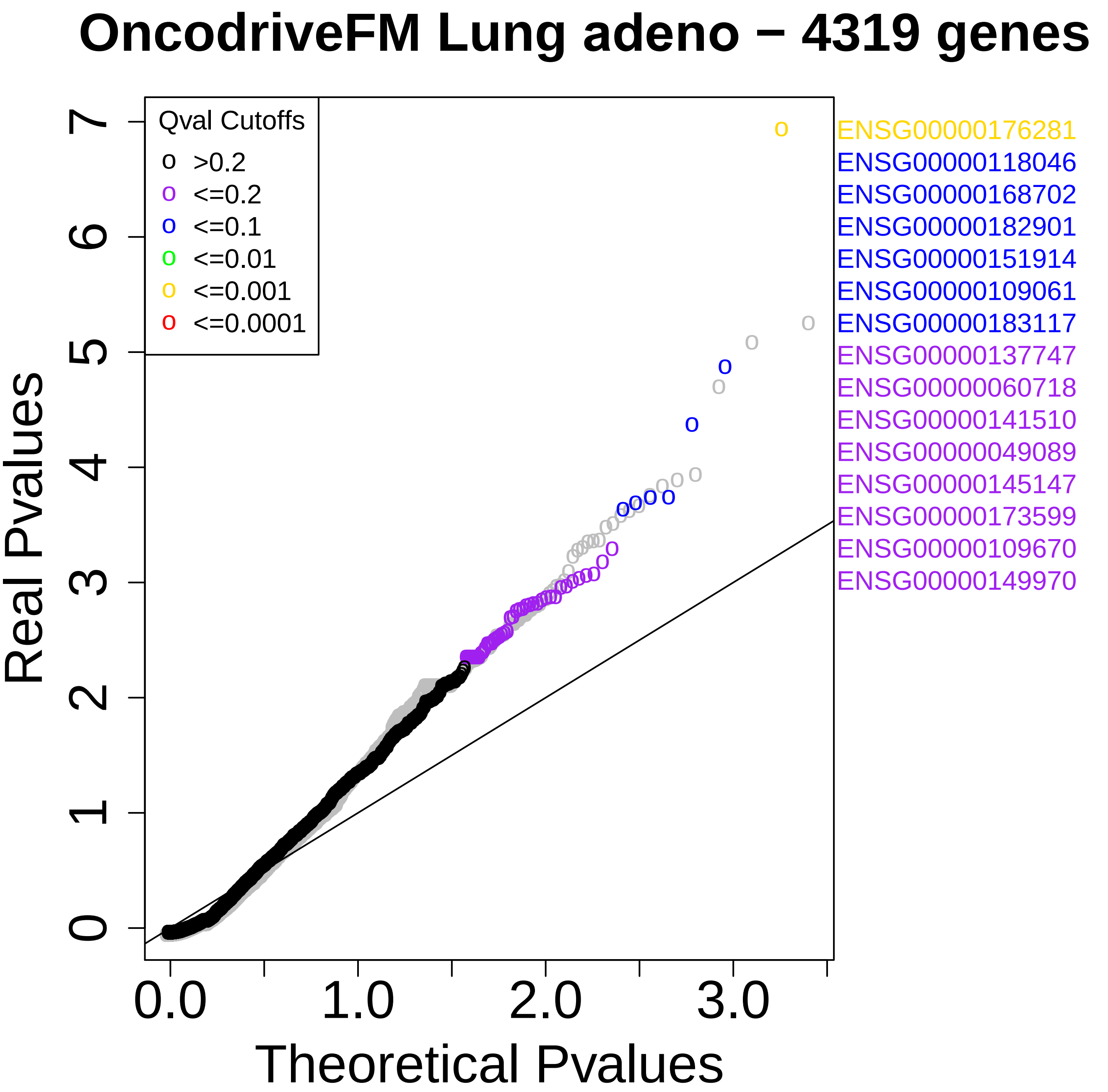

OncodriveFM Lung adeno − 4319 genes
Qval Cutoffs
o >0.2
o <=0.2
o <=0.1
o <=0.01
o <=0.001
o <=0.0001
Real Pvalues
Theoretical Pvalues
0
1
2
3
4
5
6
7
0.0
1.0
2.0
3.0
ENSG00000176281
ENSG00000118046
ENSG00000168702
ENSG00000182901
ENSG00000151914
ENSG00000109061
ENSG00000183117
ENSG00000137747
ENSG00000060718
ENSG00000141510
ENSG00000049089
ENSG00000145147
ENSG00000173599
ENSG00000109670
ENSG00000149970

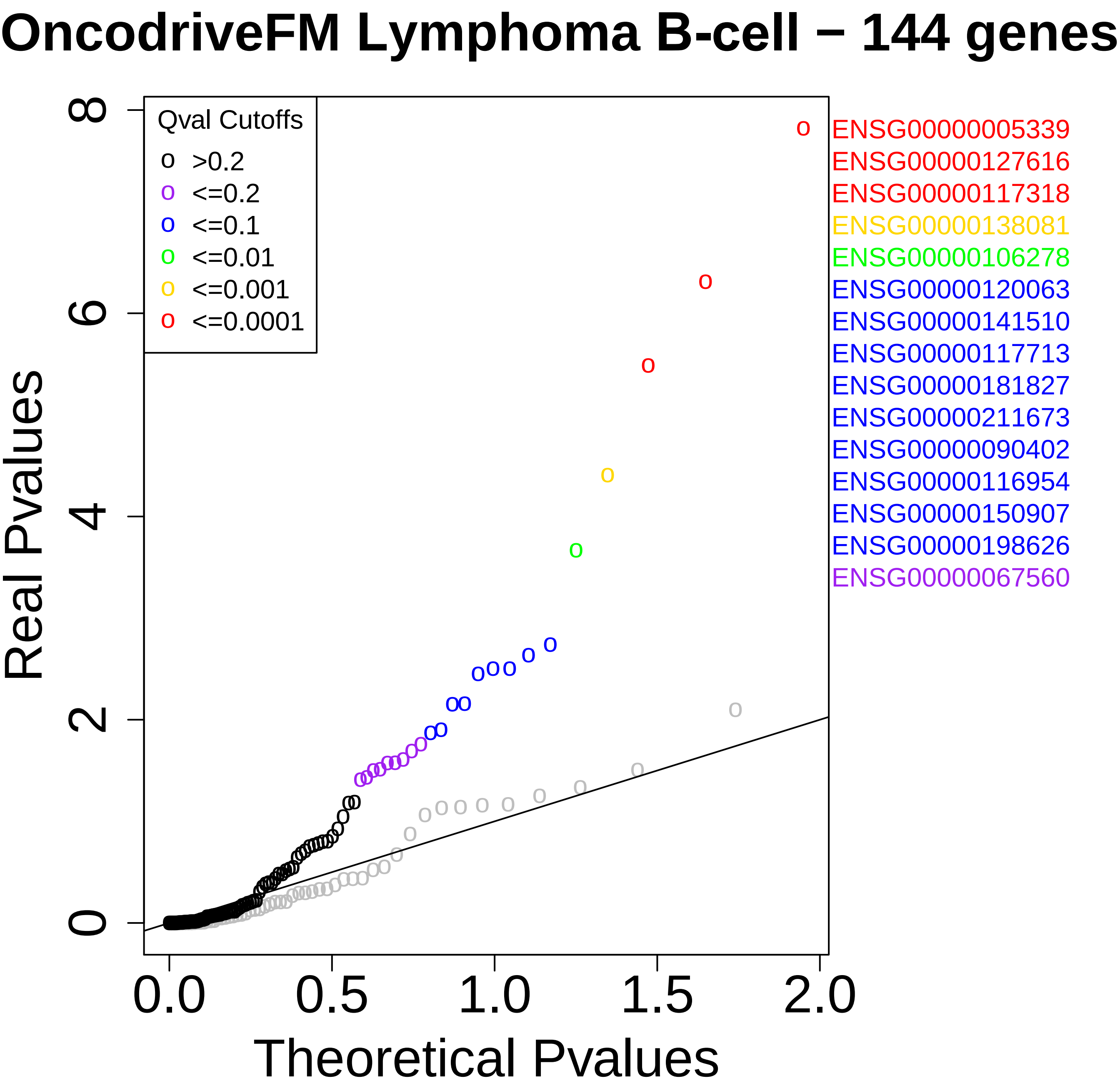
OncodriveFM Lymphoma B-cell − 144 genes
Qval Cutoffs
o >0.2
o <=0.2
o <=0.1
o <=0.01
o <=0.001
o <=0.0001
Real Pvalues
Theoretical Pvalues
0
2
4
6
8
0.0
0.5
1.0
1.5
2.0
ENSG00000005339
ENSG00000127616
ENSG00000117318
ENSG00000138081
ENSG00000106278
ENSG00000120063
ENSG00000141510
ENSG00000117713
ENSG00000181827
ENSG00000211673
ENSG00000090402
ENSG00000116954
ENSG00000150907
ENSG00000198626
ENSG00000067560

OncodriveFM Medulloblastoma − 203 genes

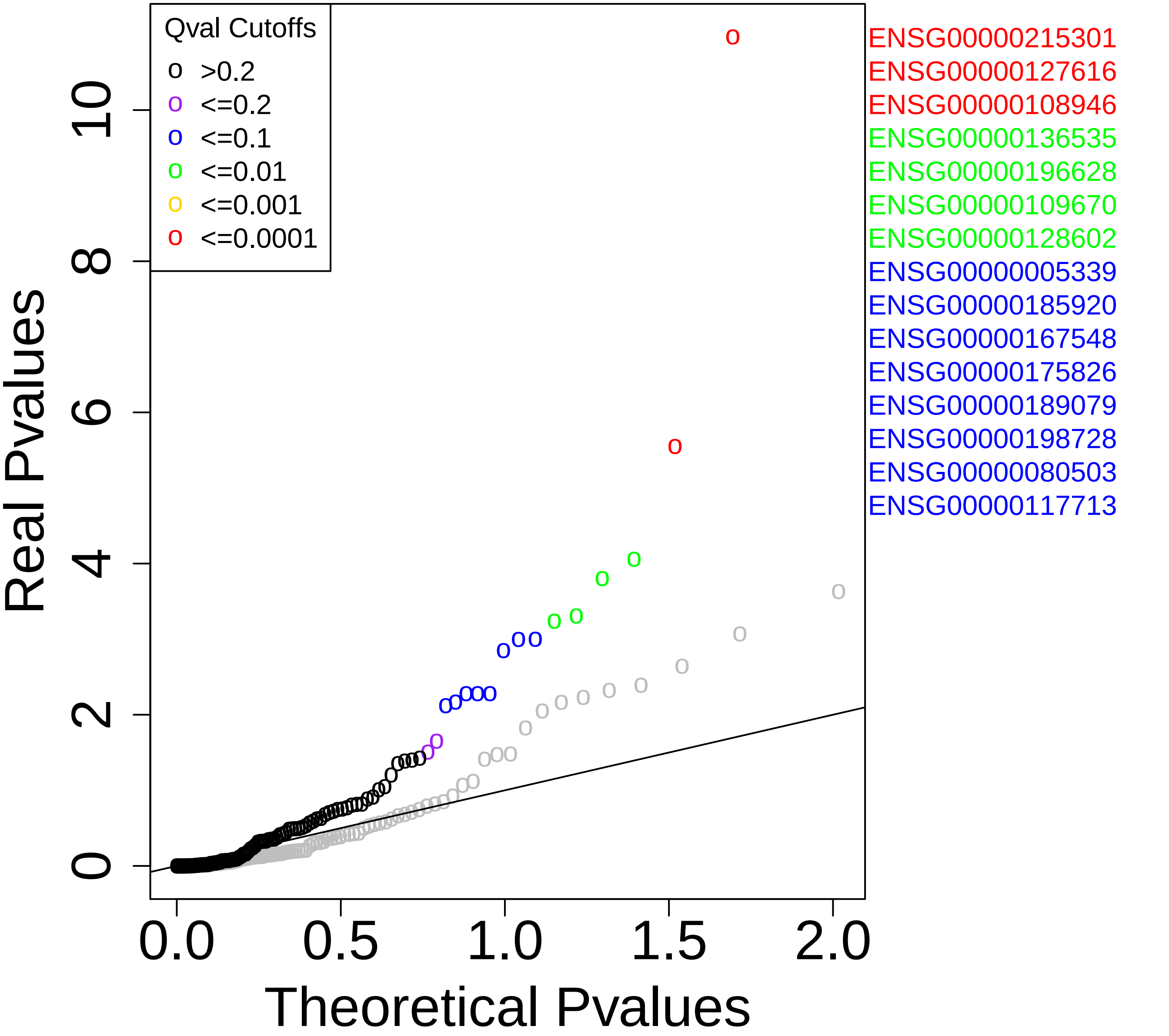

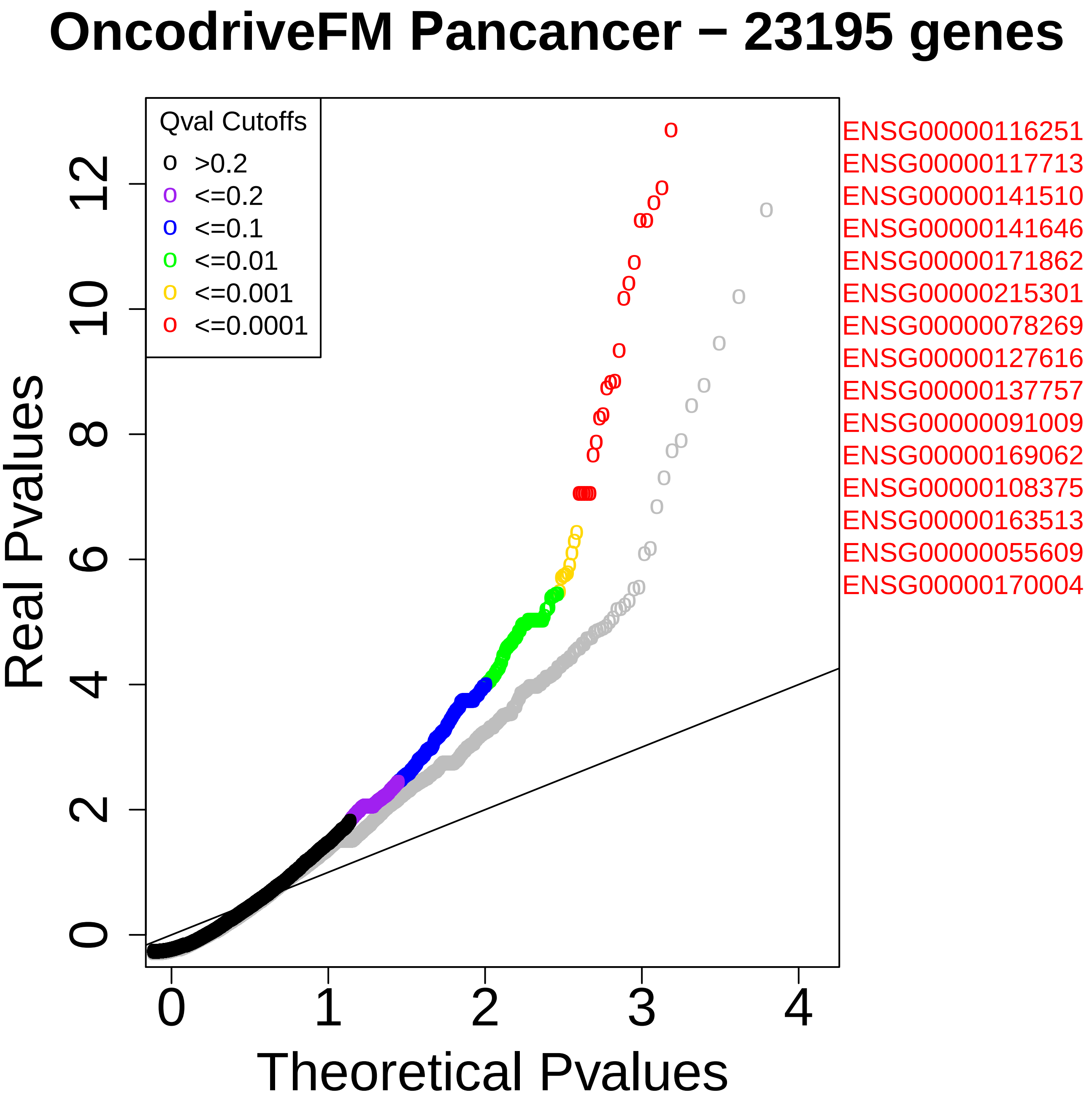

OncodriveFM Pancancer − 23195 genes
Qval Cutoffs
o >0.2
o <=0.2
o <=0.1
o <=0.01
o <=0.001
o <=0.0001
Real Pvalues
Theoretical Pvalues
0
2
4
6
8
10
12
0
1
2
3
4
ENSG00000116251
ENSG00000117713
ENSG00000141510
ENSG00000141646
ENSG00000171862
ENSG00000215301
ENSG00000078269
ENSG00000127616
ENSG00000137757
ENSG00000091009
ENSG00000169062
ENSG00000108375
ENSG00000163513
ENSG00000055609
ENSG00000170004

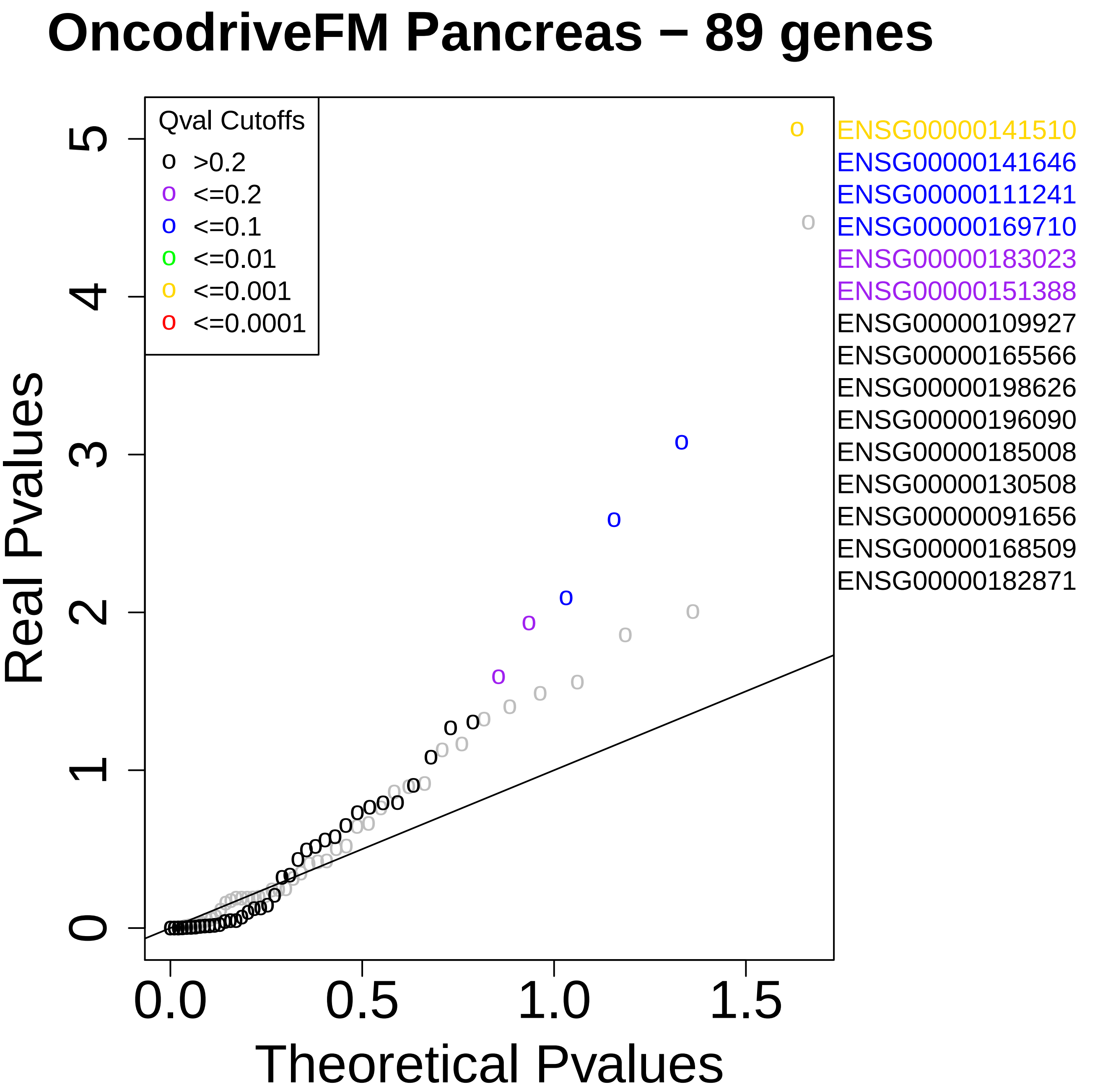
OncodriveFM Pancreas – 89 genes
Qval Cutoffs
>0.2
<=0.2
<=0.1
<=0.01
<=0.001
<=0.0001
Real Pvalues
Theoretical Pvalues
0
1
2
3
4
5
0.0
0.5
1.0
1.5
ENSG00000141510
ENSG00000141646
ENSG00000111241
ENSG00000169710
ENSG00000183023
ENSG00000151388
ENSG00000109927
ENSG00000165566
ENSG00000198626
ENSG00000196090
ENSG00000185008
ENSG00000130508
ENSG00000091656
ENSG00000168509
ENSG00000182871

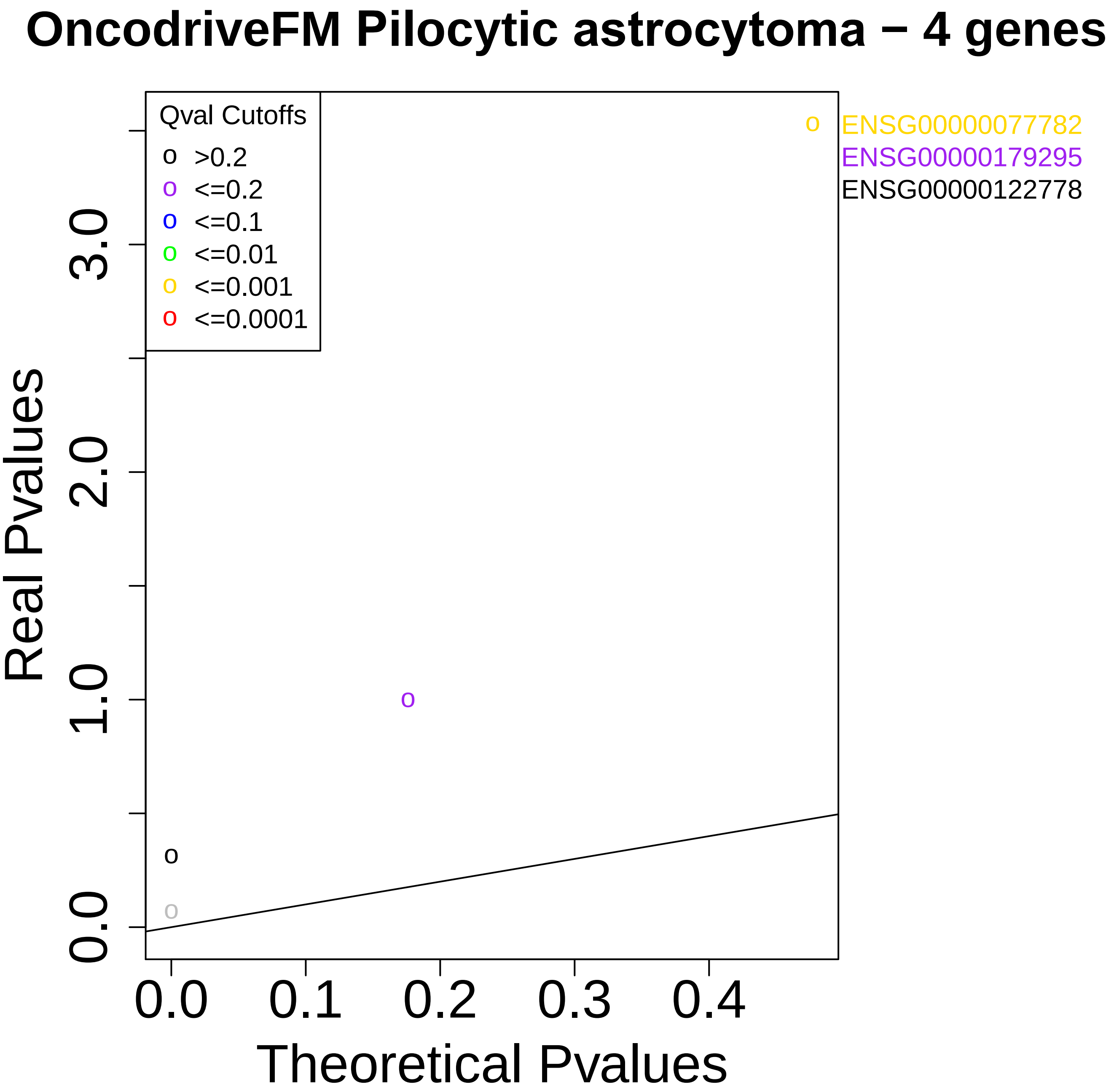
OncodriveFM Pilocytic astrocytoma − 4 genes
Qval Cutoffs
>0.2
<=0.2
<=0.1
<=0.01
<=0.001
<=0.0001
ENSG00000077782
ENSG00000179295
ENSG00000122778
Real Pvalues
0.0
1.0
2.0
3.0
Theoretical Pvalues
0.0
0.1
0.2
0.3
0.4

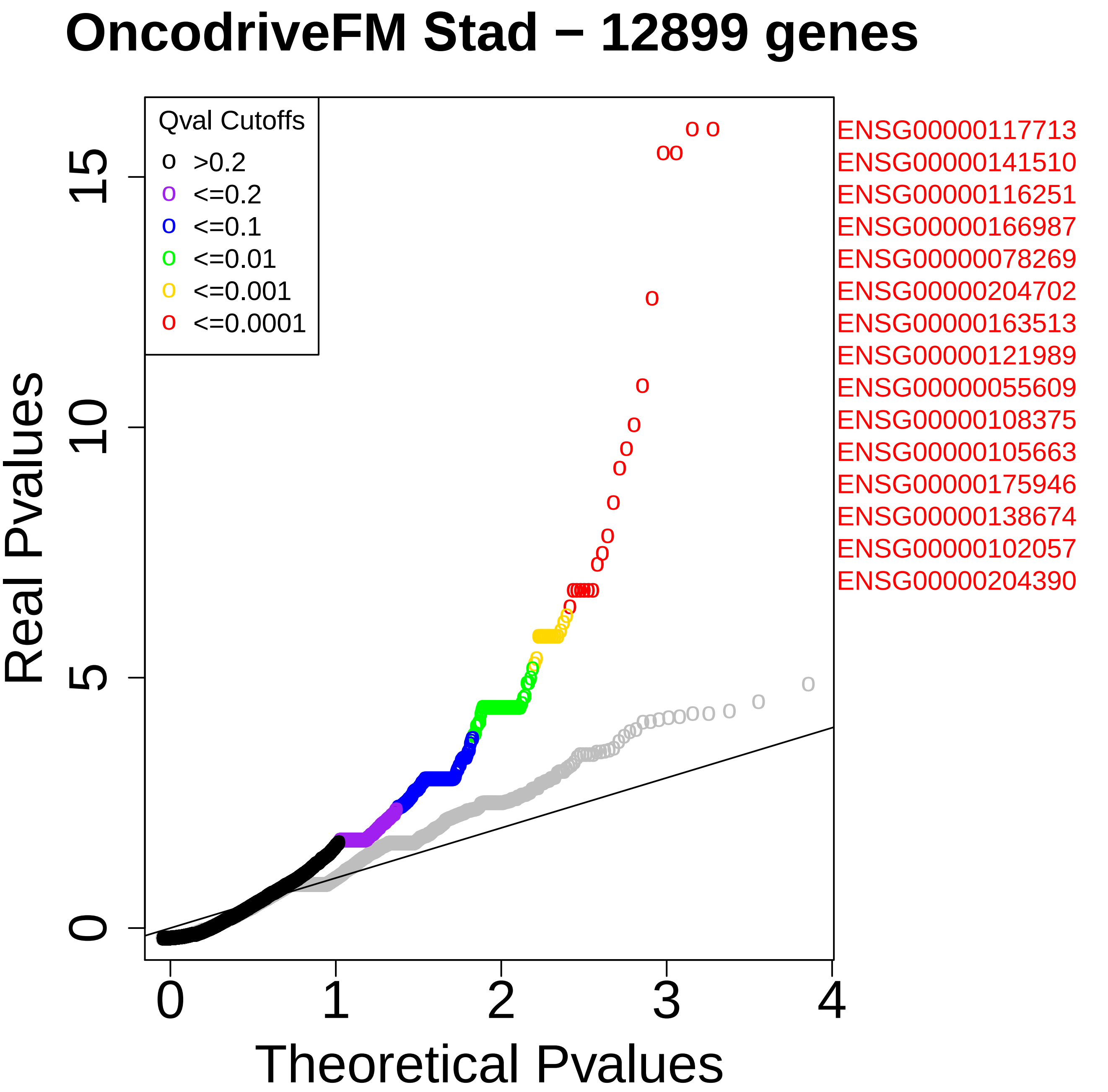
OncodriveFM Stad − 12899 genes
Qval Cutoffs
o >0.2
o <=0.2
o <=0.1
o <=0.01
o <=0.001
o <=0.0001
Real Pvalues
Theoretical Pvalues
0
5
10
15
0
1
2
3
4
ENSG00000117713
ENSG00000141510
ENSG00000116251
ENSG00000166987
ENSG00000078269
ENSG00000204702
ENSG00000163513
ENSG00000121989
ENSG00000055609
ENSG00000108375
ENSG00000105663
ENSG00000175946
ENSG00000138674
ENSG00000102057
ENSG00000204390

Supplementary Figure S12

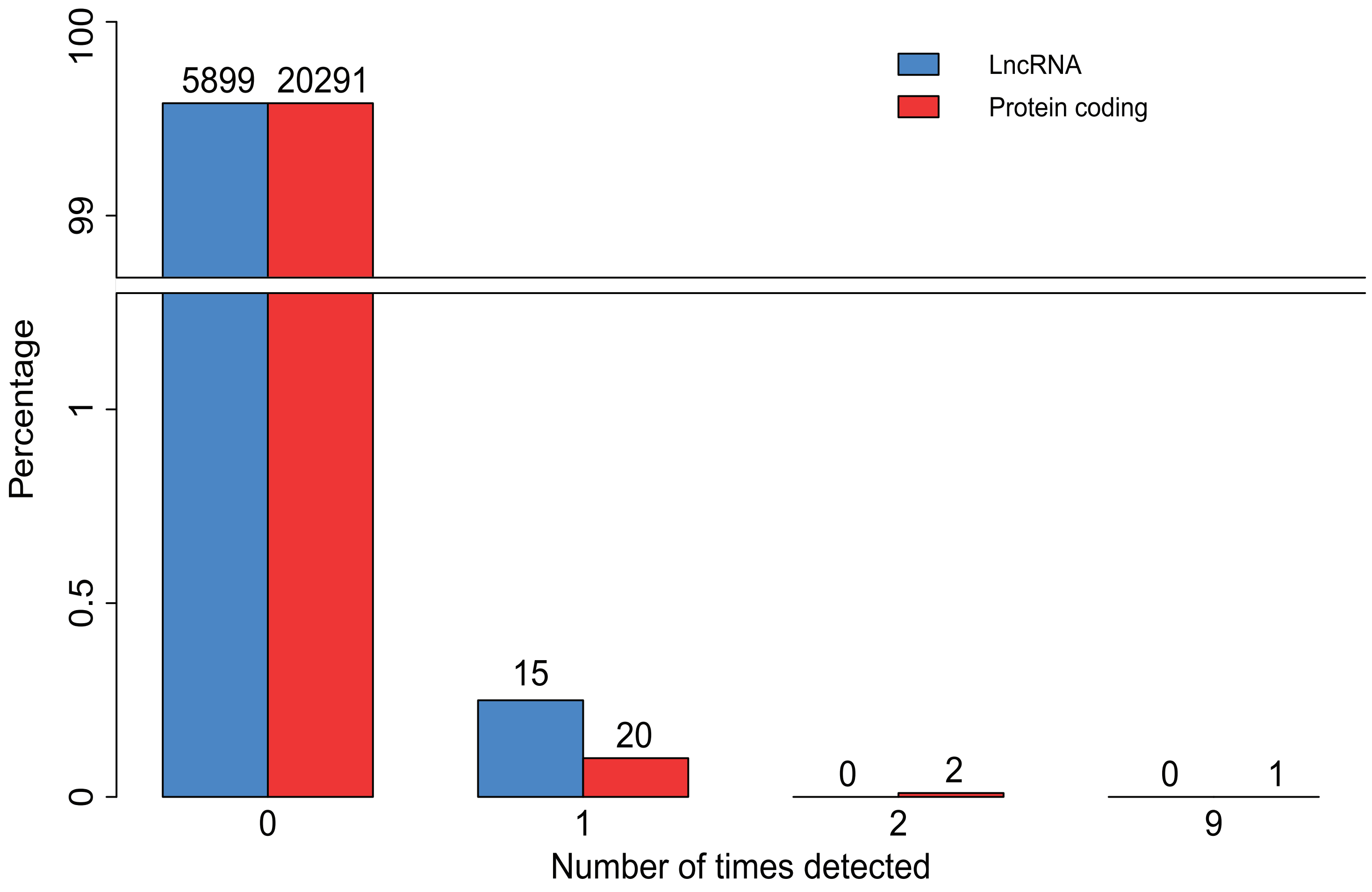

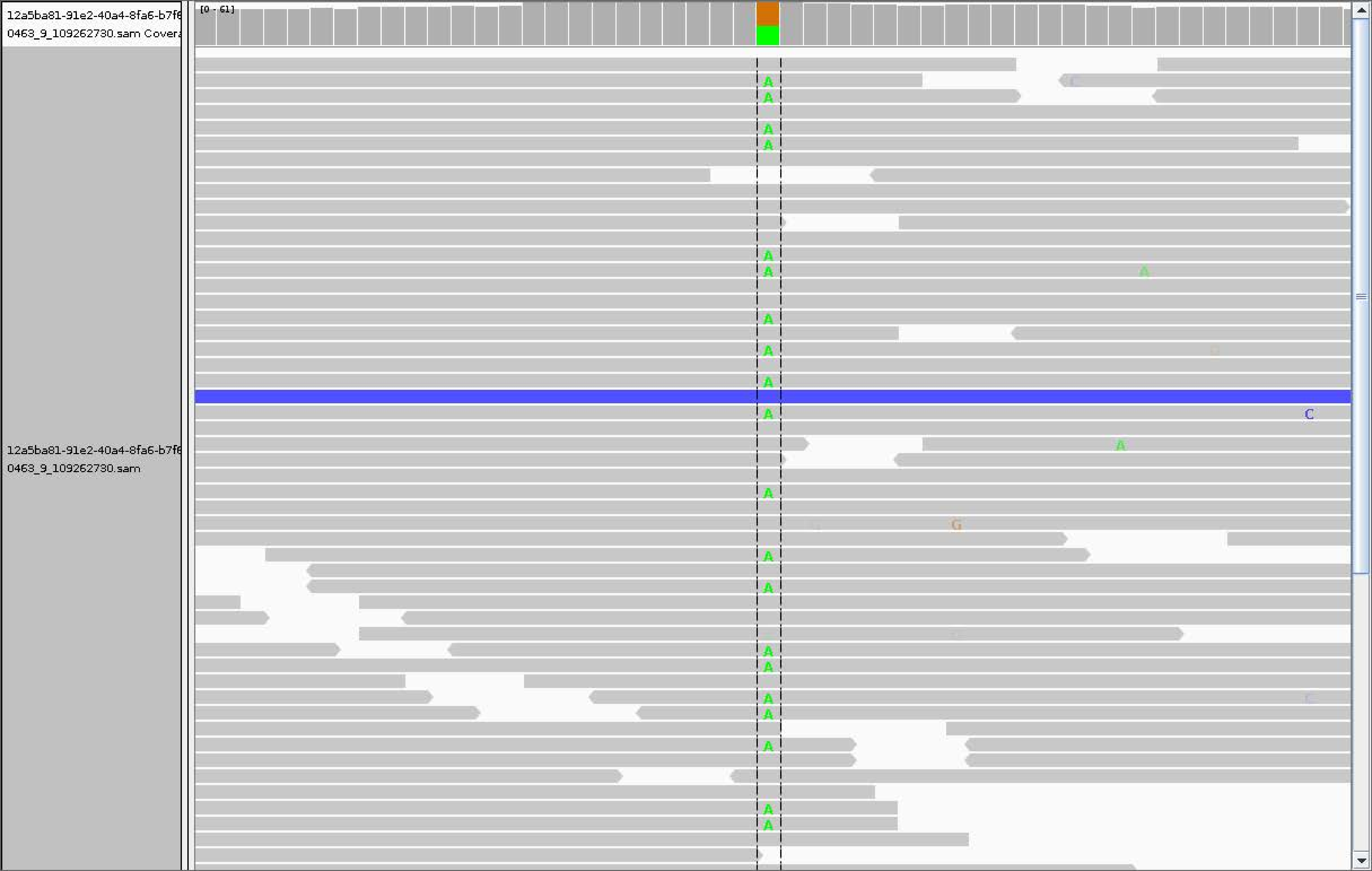
12a5ba81-91e2-40a4-8fa6-b7f6
0463_9_109262730.sam Covera
[0 - 61]
12a5ba81-91e2-40a4-8fa6-b7f6
0463_9_109262730.sam

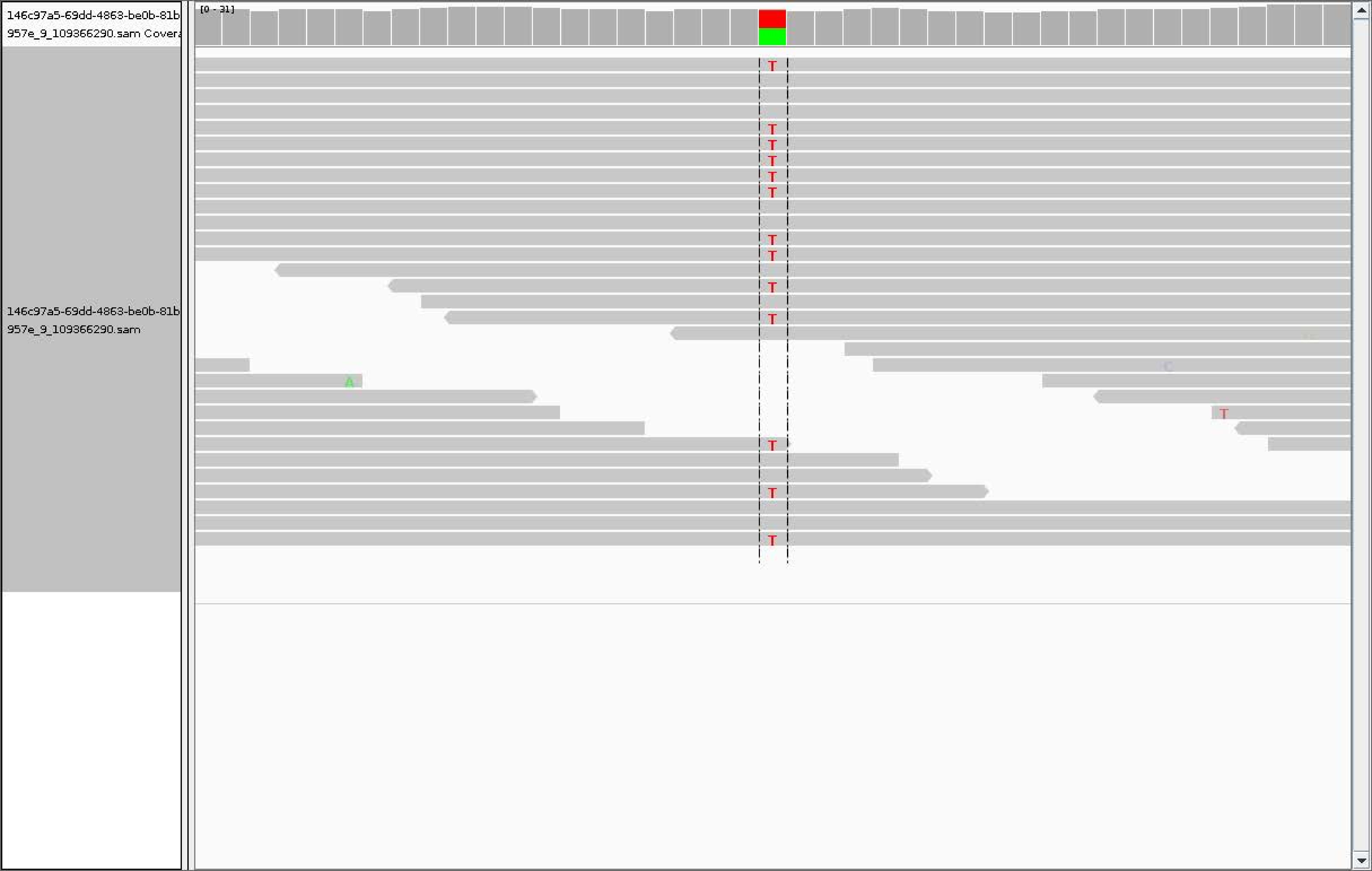
146c97a5-69dd-4863-be0b-81b
957e_9_109366290.sam Covera
[0 - 31]
146c97a5-69dd-4863-be0b-81b
957e_9_109366290.sam

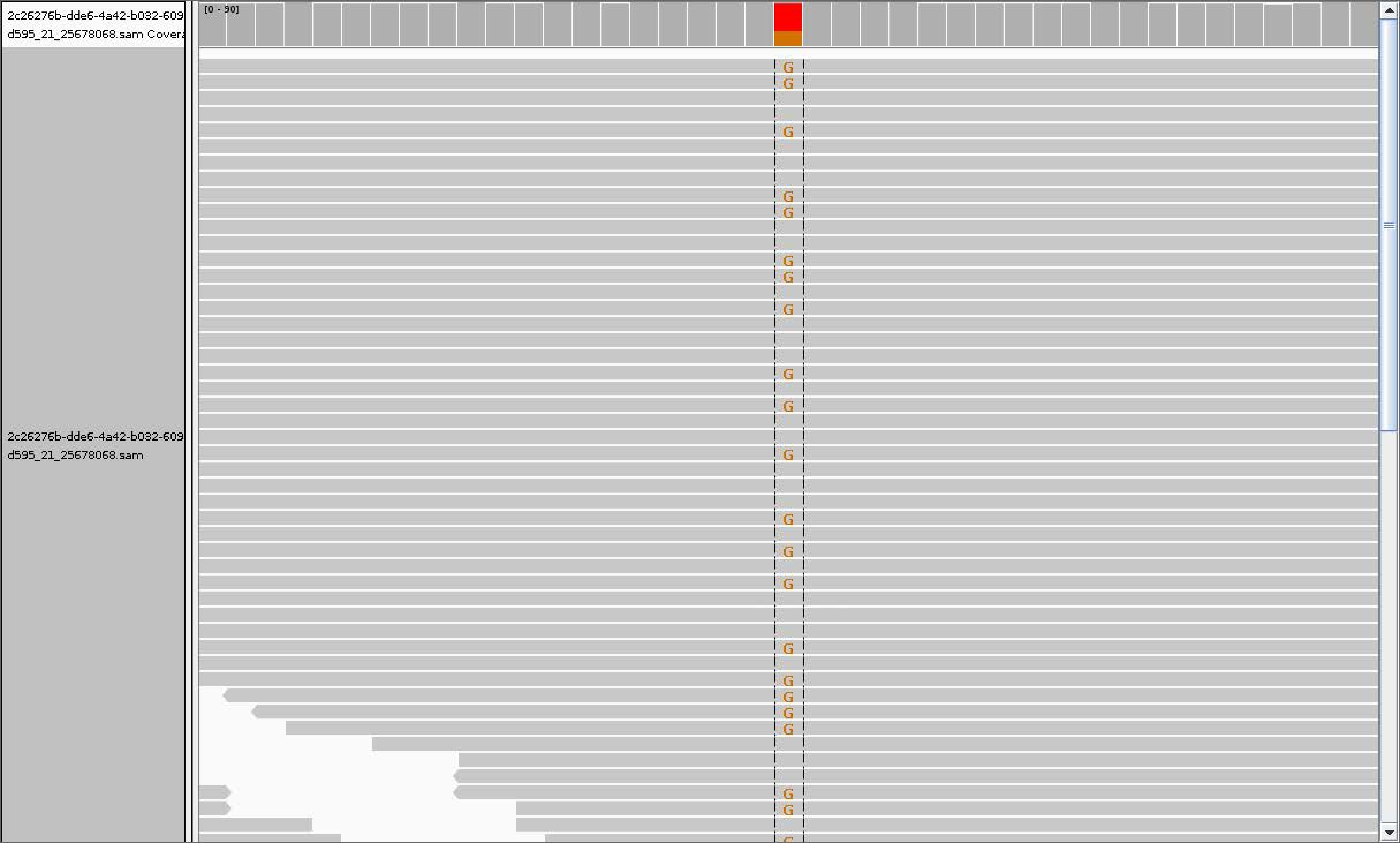
2c26276b-dde6-4a42-b032-609
d595_21_25678068.sam Covera
[0 - 90]
2c26276b-dde6-4a42-b032-609
d595_21_25678068.sam

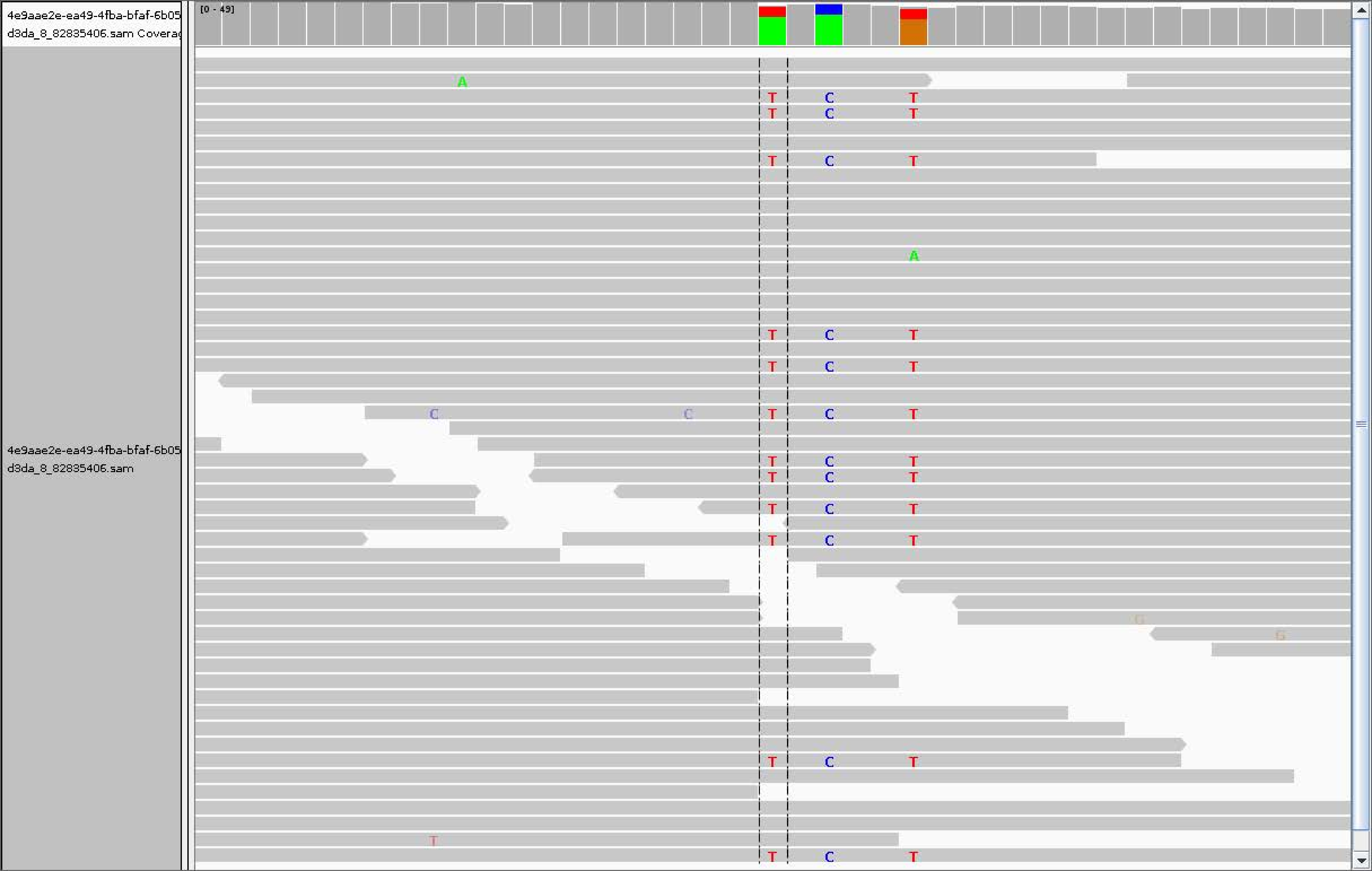
4e9aae2e-ea49-4fba-bfaf-6b05
d3da_8_82835406.sam Coverag
[0 - 49]
4e9aae2e-ea49-4fba-bfaf-6b05
d3da_8_82835406.sam

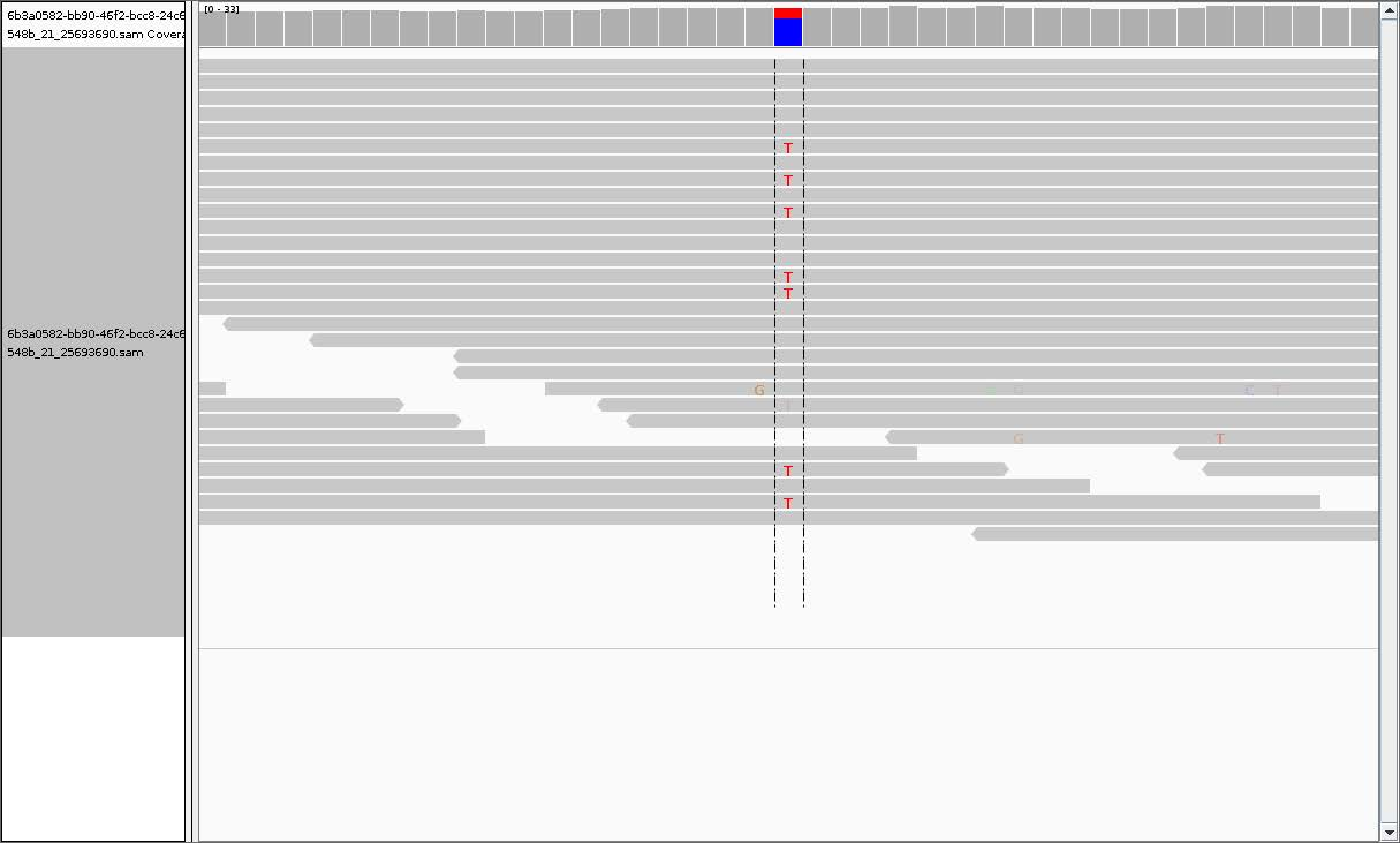
6b3a0582-bb90-46f2-bcc8-24c6
548b_21_25693690.sam Covera
[0 - 33]
6b3a0582-bb90-46f2-bcc8-24c6
548b_21_25693690.sam

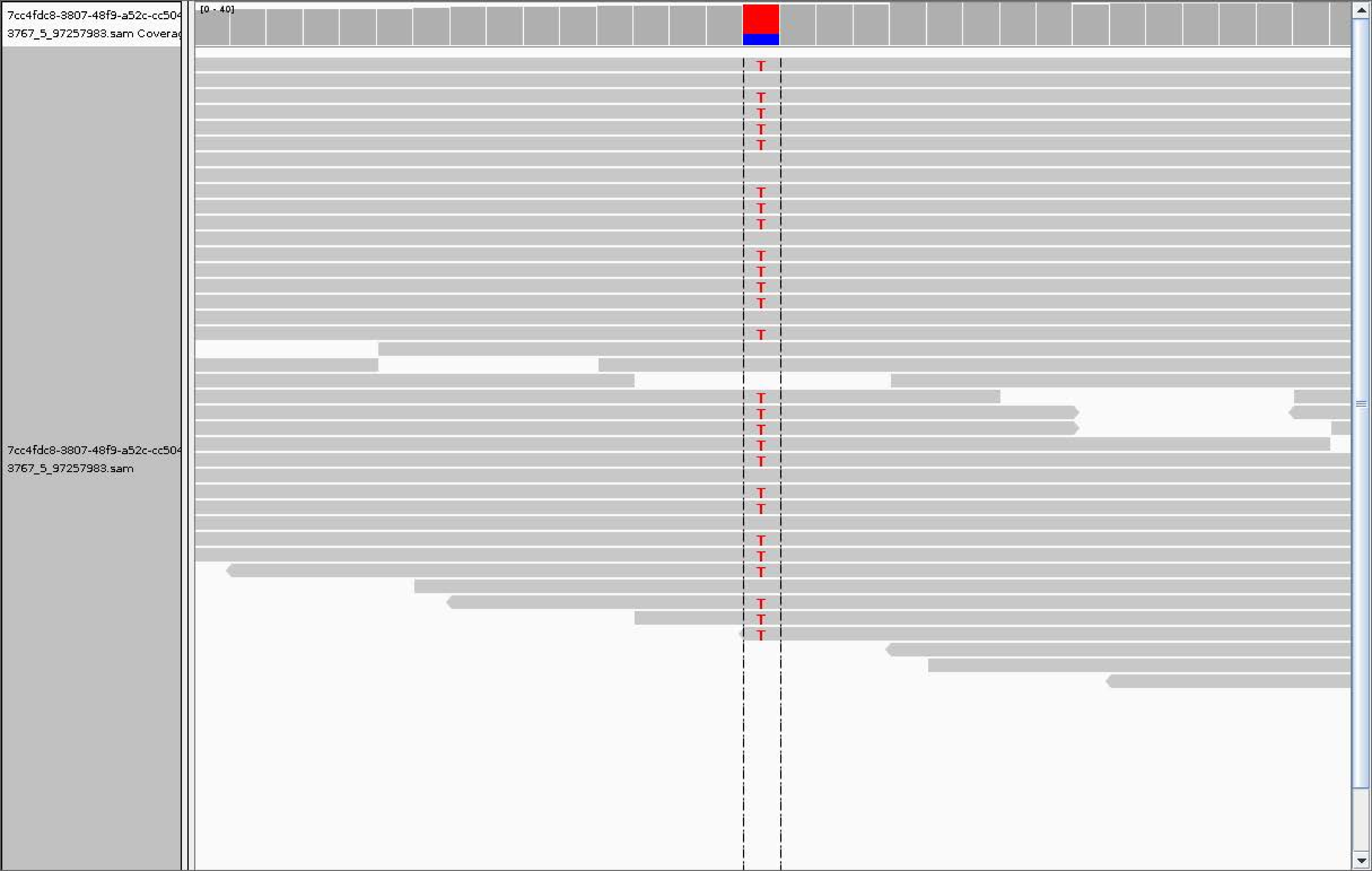
7cc4fdc8-3807-48f9-a52c-cc504
3767_5_97257983.sam Coverag
[0 - 40]
7cc4fdc8-3807-48f9-a52c-cc504
3767_5_97257983.sam

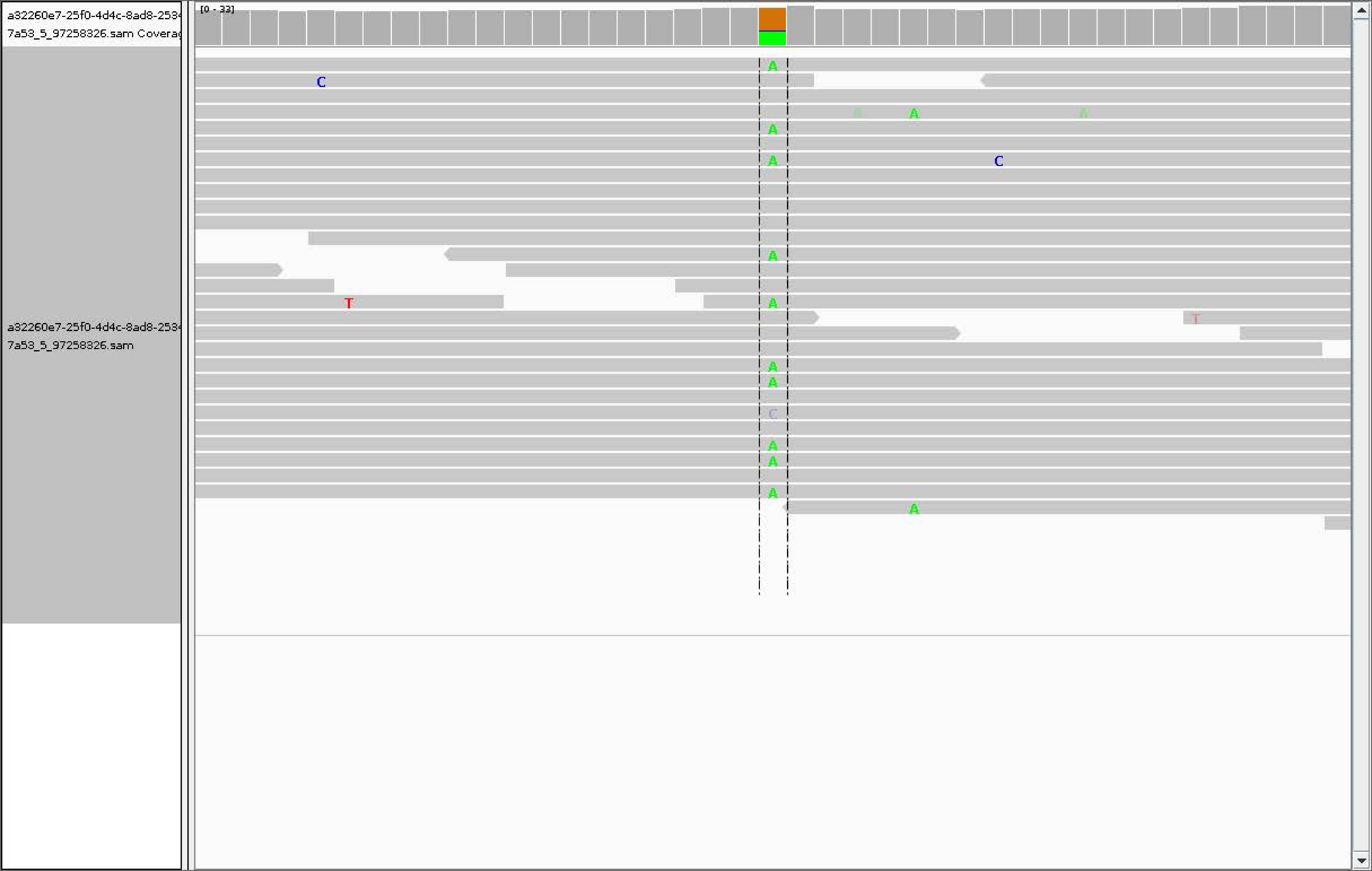
a32260e7-25f0-4d4c-8ad8-253
7a53_5_97258326.sam Coverag
[0 - 33]
a32260e7-25f0-4d4c-8ad8-253
7a53_5_97258326.sam

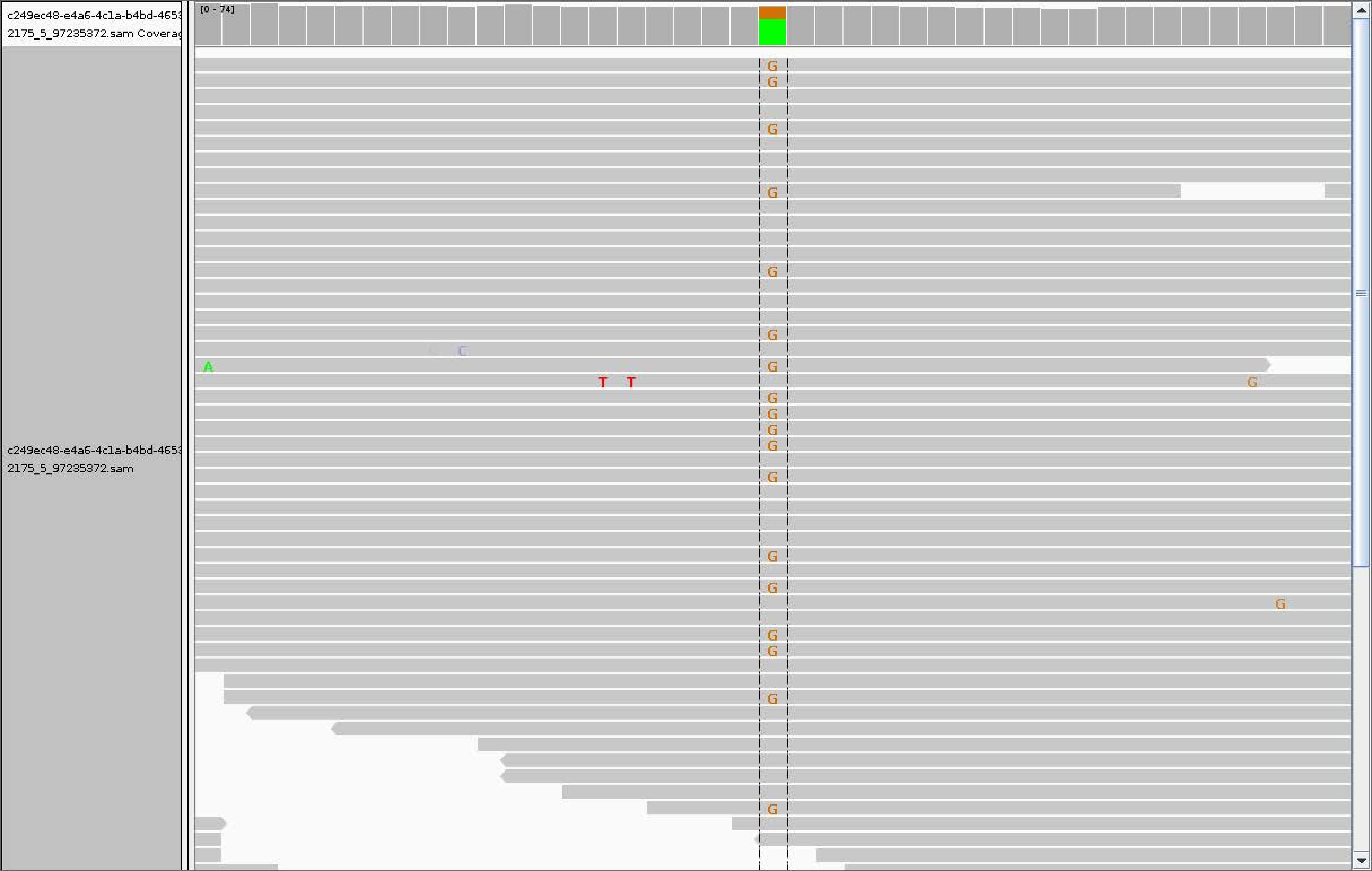
c249ec48-e4a6-4c1a-b4bd-4653
2175_5_97235372.sam Coverag
[0 - 74]
c249ec48-e4a6-4c1a-b4bd-4653
2175_5_97235372.sam

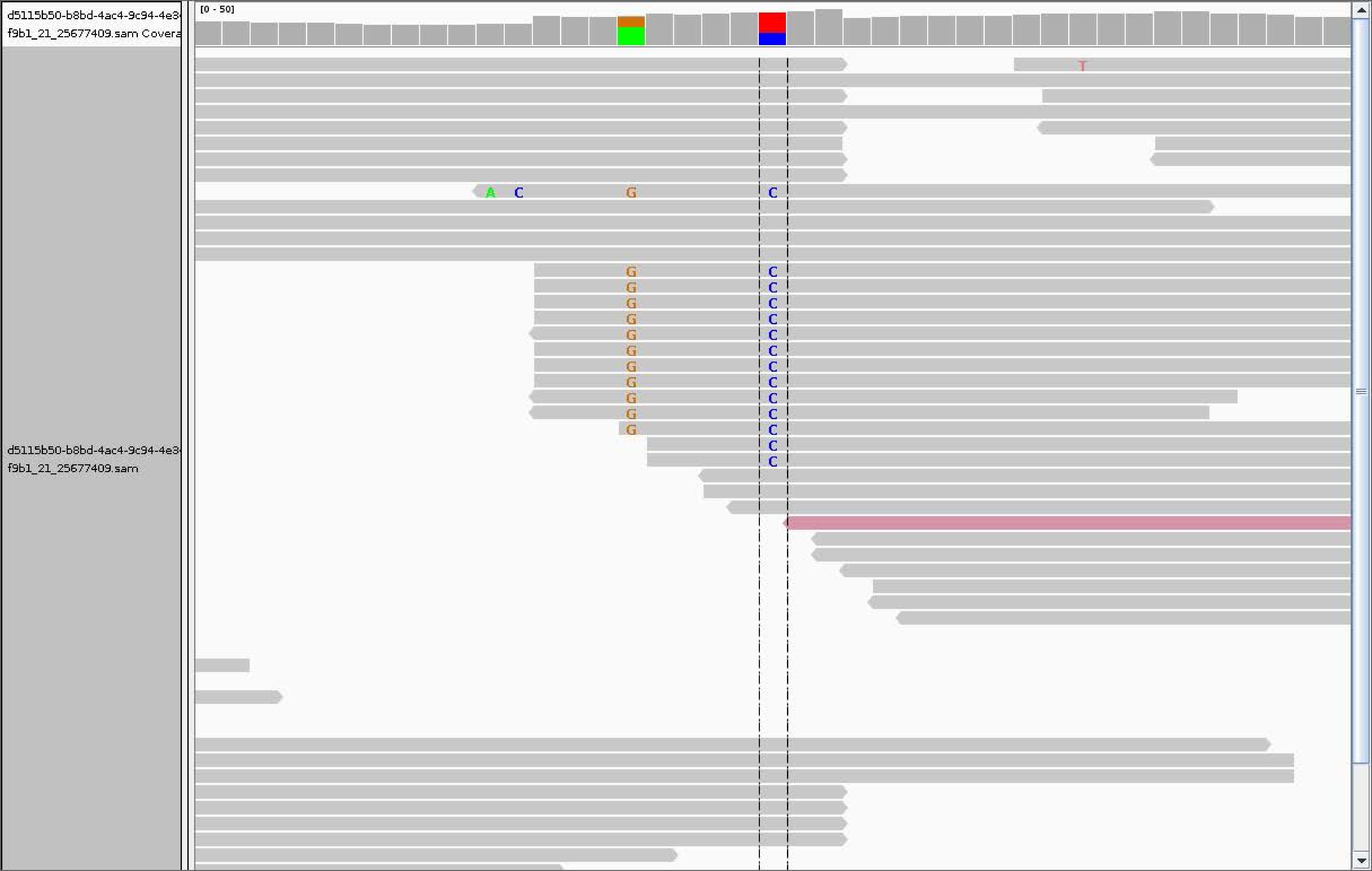
d5115b50-b8bd-4ac4-9c94-4e3
f9b1_21_25677409.sam Covera
[0 - 50]
d5115b50-b8bd-4ac4-9c94-4e3
f9b1_21_25677409.sam

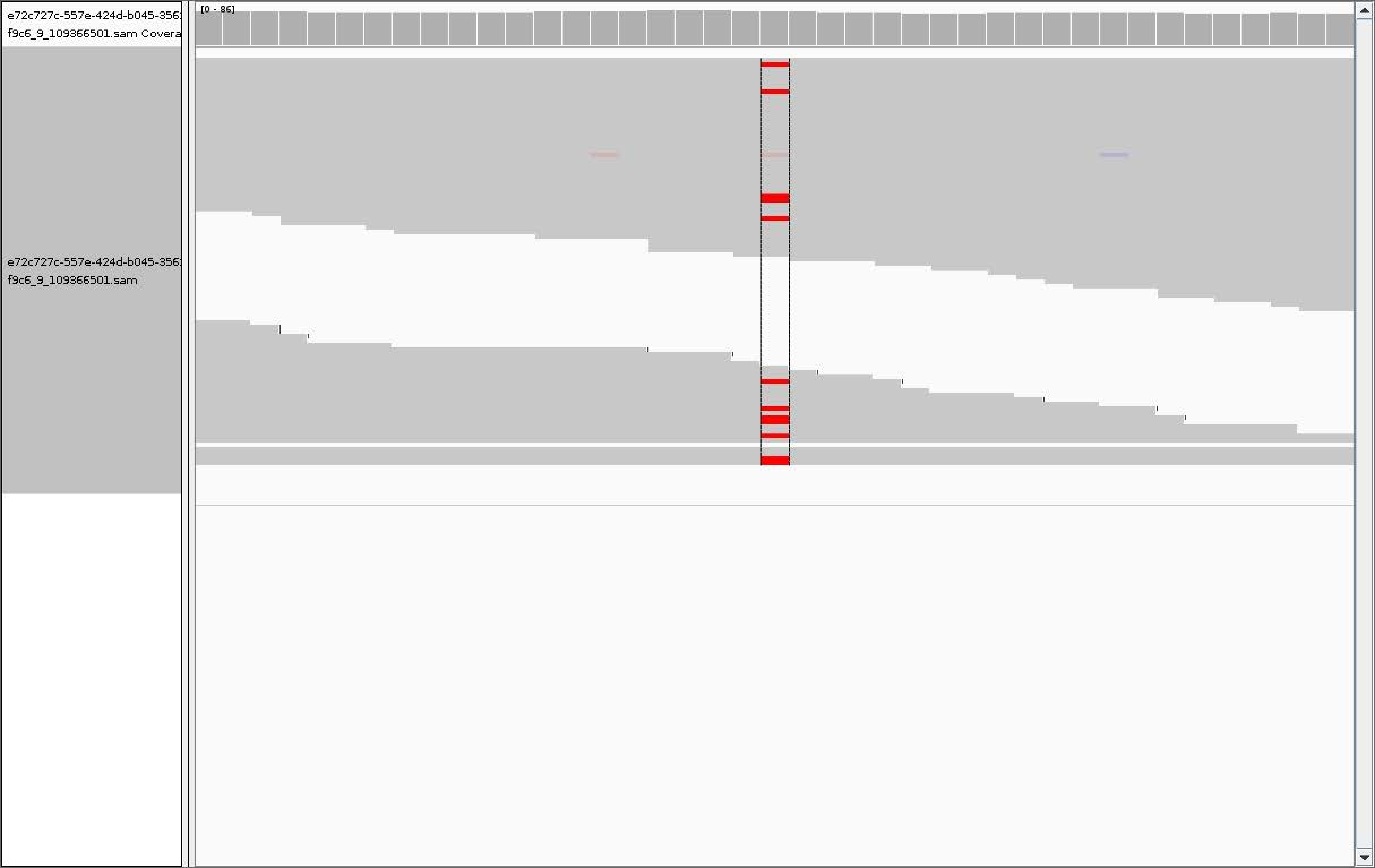
e72c727c-557e-424d-b045-356
f9c6_9_109366501.sam Covera
[0 - 86]
e72c727c-557e-424d-b045-356
f9c6_9_109366501.sam

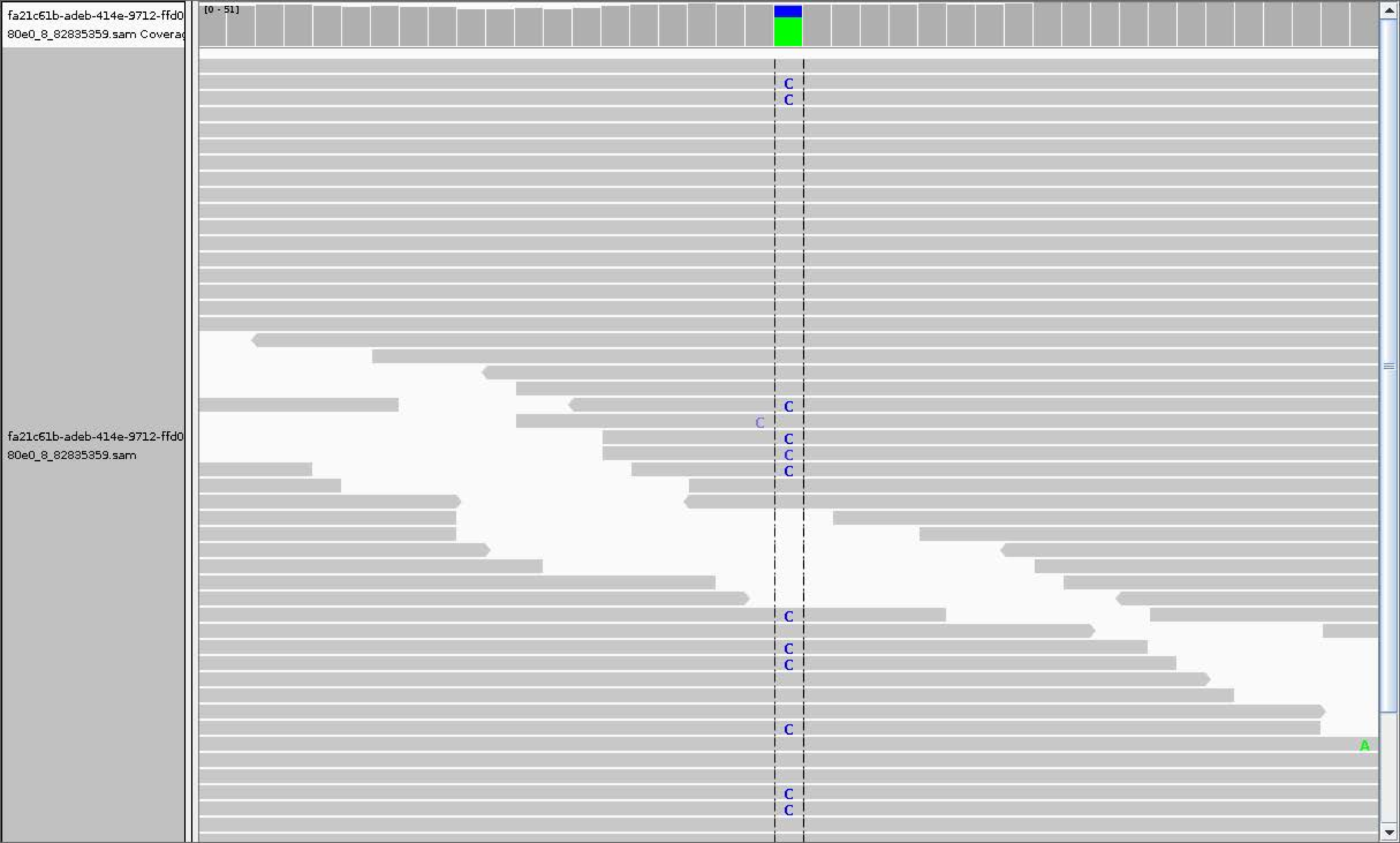
fa21c61b-adeb-414e-9712-ffd0
80e0_8_82835359.sam Coverag
[0 - 51]
fa21c61b-adeb-414e-9712-ffd0
80e0_8_82835359.sam

Supplementary Figure S14

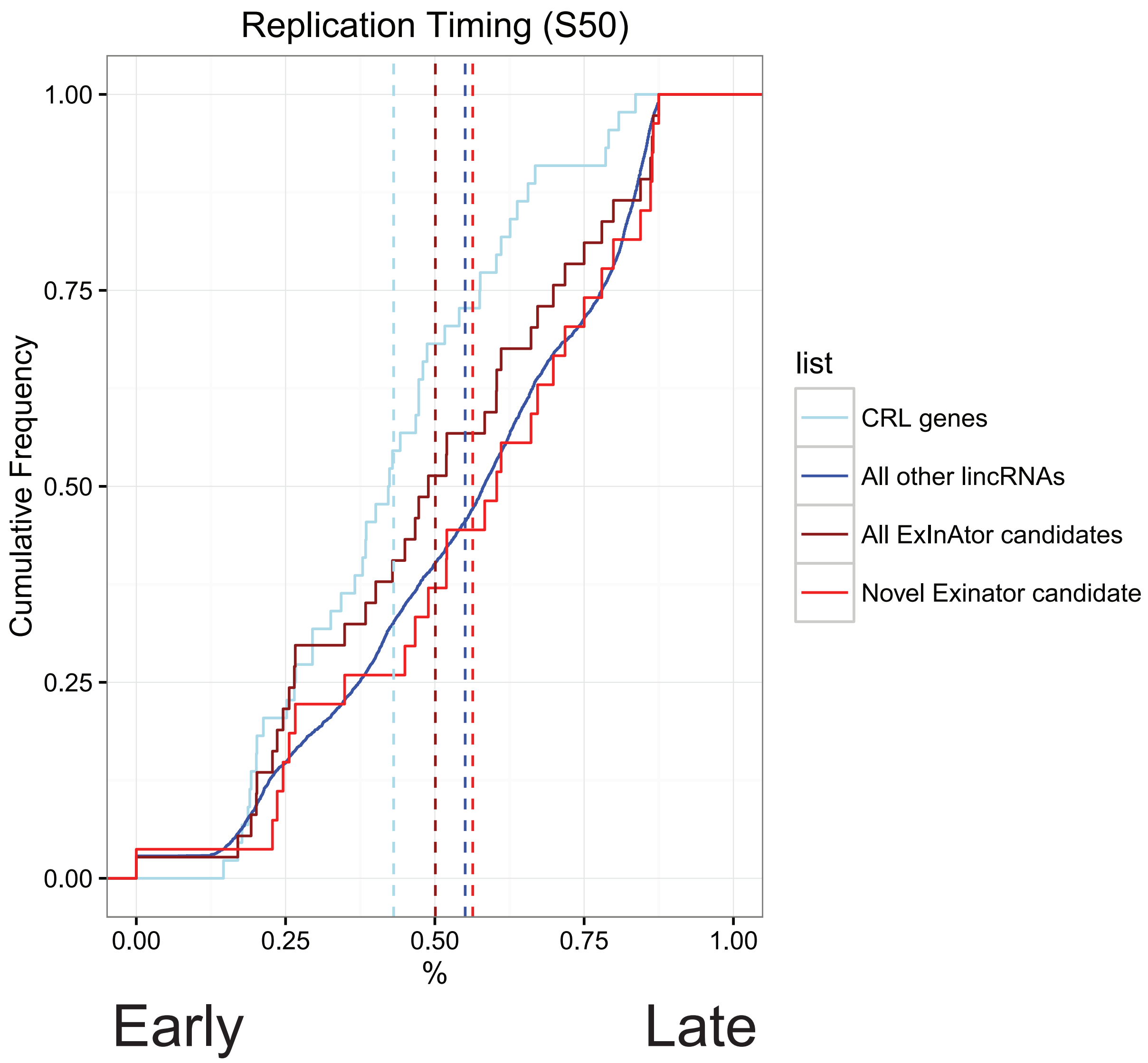

Supplement: Supplementary Figures and Legends [file srep41544-s1.pdf]
