# Supplementary material for: Discovery of Cancer Driver Long Noncoding RNAs across 1112 Tumour Genomes: New Candidates and Distinguishing Features
Source: Sci Rep. 2017 Jan 27;7:41544. doi: 10.1038/srep41544 (PMC5269722; doi:10.1038/srep41544)
Supplement: Supplementary Dataset 12 [file srep41544-s13.doc]

**Supplementary File S12**

**Effects on ExInAtor predictions of various filtering schemes.**

1. Effect of germline variant filters

Here we test the effect on driver gene prediction using stringent filters to remove germline variants.

By default throughout the study, two filters were implemented on SNP calls:

1. Multiple overlapping identical mutations were counted as one mutation – “merged”.
2. Any mutation call overlapping and having the same base change as an entry from dbSNP common list was removed.

Below we test two other filters, where any mutations are removed that overlap:

1. the entire 1000 Genomes variant set (Phase 3), or
2. all dbSNP145 entries

The tables show the total number of drivers predicted across all cancers and pancancer combinations exactly as in the main manuscript. Predictions using settings as in the main manuscript are highlighted in grey.

| **Protein coding** | | | | | |
| --- | --- | --- | --- | --- | --- |
| Dataset | Merged? | CGC | notCGC | Sensitivity (%) | Precision (%) |
| 1000 genomes | no | 14 | 26 | 2.57 | 35.00 |
| all dbSNP | no | 2 | 44 | 0.37 | 4.35 |
| common dbSNP | yes | 9 | 15 | 1.65 | 37.50 |

| **LncRNA** | | | | | |
| --- | --- | --- | --- | --- | --- |
| Dataset | Merged? | CRL | notCRL | Sensitivity (%) | Precision (%) |
| 1000 genomes | no | 4 | 34 | 8.89 | 10.53 |
| all dbSNP | no | 6 | 36 | 13.33 | 14.29 |
| common dbSNP | yes | 6 | 9 | 13.33 | 40.00 |

In summary, filtering using germline catalogues results in a loss of sensitivity and precision for lncRNA predictions.

2. Effect of removing hypermutated samples

Extrapolating from Cosmic’s definition of hypermutation in exomes (15,000, <http://cancer.sanger.ac.uk/cosmic/analyses>), we define 140,000 genome-wide as the threshold for our whole-genome data. Applying this to the datasets used in this study leads to the following reduction in samples:

|  | Mutations | | | Samples | | |
| --- | --- | --- | --- | --- | --- | --- |
|  | All | Remaining after filter | Remaining (%) | All | Remaining | Remaining (%) |
| Alexandrov | 6,827,622 | 5,073,225 | 74 | 607 | 601 | 99 |
| TCGA | 15,530,168 | 7,362,502 | 47 | 505 | 482 | 95 |

The below table shows ExInAtor prediction performance on the entire dataset, before and after this filter is imposed. The table shows the total number of drivers predicted across all cancers and pancancer combinations exactly as in the main manuscript. Predictions using settings as in the main manuscript are highlighted in grey.

| **Protein-coding** |  |  |  |  |
| --- | --- | --- | --- | --- |
|  | CGC | notCGC | Sensitivity (%) | Precision (%) |
| Remove hypermutated | 8 | 14 | 1.5 | 36.4 |
| All samples | 9 | 15 | 1.7 | 37.5 |
|  |  |  |  |  |
| **LncRNA** |  |  |  |  |
|  | CRL | notCRL | Sensitivity (%) | Precision (%) |
| Remove hypermutated | 2 | 15 | 4.4 | 11.8 |
| All samples | 6 | 9 | 13.3 | 40 |

In summary, removal of hypermutated samples results in a loss of sensitivity and precision for lncRNA and protein-coding genes.

3. Masking of repetitive sequence.

Here, the effect of masking (omitting) all annotated repetitive sequence was tested. The full RepeatMasker annotation was downloaded from UCSC Genome Browser for GRCh37/hg19. All nucleotides and mutations overlapping these were ignored.

Altogether, RepeatMasker regions represent 1,467,396,988 bp (47% of the genome). Subtracting these from the used GENCODE annotations resulted in a 44% and 50% reduction in the total (unspliced) length of protein-coding and lncRNA genes, respectively.

The table shows the total number of drivers predicted across all cancers and pancancer combinations exactly as in the main manuscript.

| **Protein-coding** |  |  |  |  |
| --- | --- | --- | --- | --- |
|  | CGC | notCGC | Sensitivity (%) | Precision (%) |
| Mask repeats | 3 | 4 | 0.55 | 42.9 |
| Original | 9 | 15 | 1.7 | 37.5 |
|  |  |  |  |  |
| **LncRNA** |  |  |  |  |
|  | CRL | notCRL | Sensitivity (%) | Precision (%) |
| Mask repeats | 1 | 3 | 2.2 | 25 |
| Original | 6 | 9 | 13.3 | 40 |

In summary, masking repetitive regions results in a loss of sensitivity and precision for lncRNA predictions, and a loss in sensitivity for protein-coding predictions.
